# Highly Enantioselective Synthesis of 3,3-Diarylpropyl Amines and 4-Aryl Tetrahydroquinolines via Ir-Catalyzed Asymmetric Hydrogenation

**DOI:** 10.1021/acs.orglett.4c04076

**Published:** 2024-12-05

**Authors:** Martí Sidro, Clara García-Mateos, Pep Rojo, Yisong Wen, Antoni Riera, Xavier Verdaguer

**Affiliations:** † Institute for Research in Biomedicine (IRB Barcelona), Barcelona Institute of Science and Technology (BIST), Baldiri Reixac 10, 08028 Barcelona, Spain; ‡ Departament de Química Inorgànica i Orgànica, Secció Química Orgànica, Universitat de Barcelona, Martí i Franquès 1, 08028 Barcelona, Spain

## Abstract

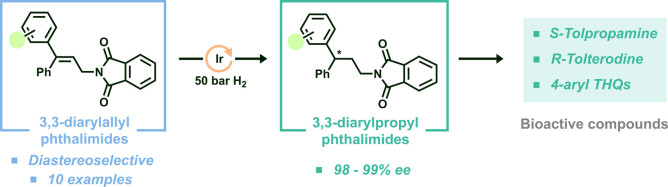

Chiral nitrogen-containing compounds are crucial for the chemical, pharmaceutical, and agrochemical industries. Nevertheless, the synthesis of certain valuable scaffolds remains underdeveloped due to the vast chemical space available. In this work, we present a diastereoselective methodology for synthesizing 3,3-diarylallyl phthalimides, which, following iridium-catalyzed asymmetric hydrogenation using Ir–UbaPHOX, yield 3,3-diarylpropyl amines with high enantioselectivity (98–99% ee). The importance of alkene purity to achieve high enantioselectivity is discussed. The synthetic utility of the chiral propylamines obtained is demonstrated through the preparation of medicinally useful bioactive compounds like the drugs tolterodine and tolpropamine and 4-aryl tetrahydroquinolines. This strategy enables the synthesis of these compounds with the highest enantioselectivity reported to date.

Chiral amines are key fragments in many biologically active compounds, including drugs, natural products, and agrochemicals.^1^ Furthermore, many chiral amines have been used for a wide variety of synthetic purposes like resolving agents, chiral auxiliaries, or building blocks of chiral complex molecules.^2^ As a result, over recent decades, synthetic chemists have been particularly focused on their asymmetric synthesis.^3^ Despite the widespread importance of chiral amines, traditional synthetic methods, such as resolution, are still being used. To overcome the drawbacks of these methodologies, innovative catalytic asymmetric approaches are being developed.^4^ Among these, the asymmetric hydrogenation (AH) of unsaturated compounds stands out as one of the most powerful tools.^5^ Unfortunately, due to the extension of the chemical space, the AH of certain types of amine substrates is still underdeveloped. In particular, the AH of allyl amines has received little attention because they lack a proper coordinating group.

On this matter, 3,3-diarylpropyl amines rise as an interesting target. They are found in several medicinally useful bioactive compounds, including the commercially available drugs tolterodine^6^ and fesoterodine (Figure 1a).^7^ Additionally, the cyclization and functionalization of these substrates grants access to 4-aryl-substituted tetrahydroquinolines (THQs), which also hold significant relevance in the pharmaceutical industry, as reflected by their presence in numerous drugs and natural products (Figure 1b).^8^

**Figure 1:**
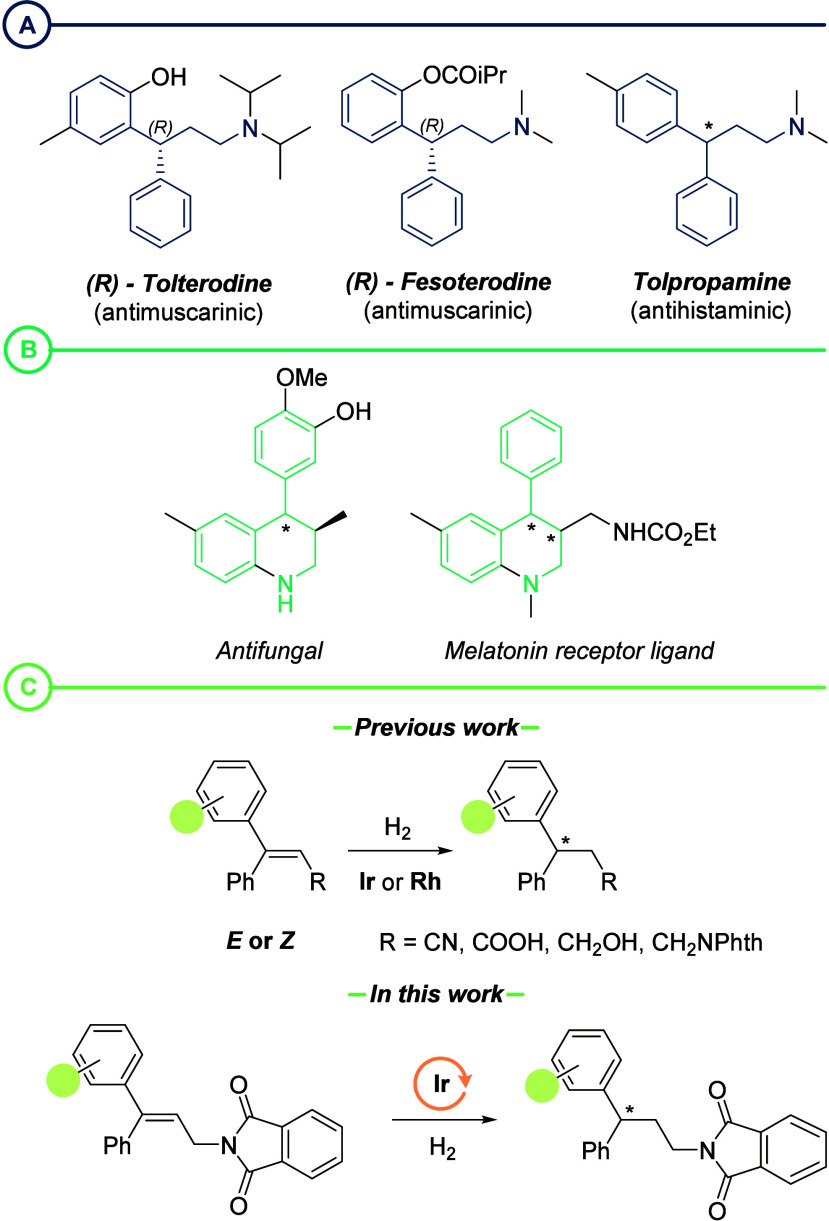
(a) Examples of commercially available drugs containing a 3,3-diarylpropyl amine core. (b) Examples of biologically active compounds with a 4-aryl-substituted THQ core. (c) Previous AH approaches to access 3,3-diarylpropyl amines and strategy envisaged in this study.

So far, catalytic asymmetric methods to synthesize 3,3-diarylpropyl compounds rely mainly on two strategies. The most widely used is the enantioselective rhodium-catalyzed 1,4-conjugate addition of arylboronic acids to β-aryl-α,β-unsaturated esters.^9^ This strategy provides good results in terms of enantioselectivity when *meta*- or *para*-substituted boronic acids are employed. However, when it comes to *ortho*-substituted compounds, the selectivity decreases. Other organometallic nucleophiles and α,β-unsaturated groups have been used without success.^10^ The alternative strategy involves metal-catalyzed AH (Figure 1c), the reaction employed herein. Currently, this approach is dominated by rhodium catalysts.^11^ Recently, the Rh-catalyzed hydrogenation of a single diarylallyl phthalimide was reported to provide 86% ee.^11e^ The unsatisfactory results obtained when synthesizing *ortho*- or *para*-substituted compounds make the use of this metal far from ideal. Iridium is another metal frequently used in AH. However, to our knowledge, only two studies on iridium-catalyzed AH of this class of compounds have been reported, both with suboptimal enantioselectivites.^12^ Therefore, a general and highly enantioselective methodology for the synthesis of 3,3-diarylpropyl amines is desirable.

Here we describe a novel approach based on the iridium-catalyzed AH of 3,3-diarylallyl phthalimides.^13^ Our strategy grants access to the desired motifs with optimal enantioselectivities regardless of the aryl substitution. We also show that the resulting substrates can be easily derivatized to obtain medicinally useful bioactive compounds like the drugs tolterodine and tolpropamine^14^ and 4-aryl THQs.

The synthesis of 3,3-diarylallyl phthalimides was envisaged from allylic alcohol **1**, which is easily accessible in a stereoselective manner from *E*-cinnamyl alcohol by Monteiro’s procedure.^15^ From compound **1**, the phthalimide and aryl fragments can be introduced in any order. Initially, we introduced the phthalimide first. This can be done either by a Mitsunobu reaction or by substitution on the corresponding mesylate. Both procedures afforded vinyl bromide **2** in excellent yield (Scheme 1). Next, the Suzuki coupling with different boronic acids provided a diverse array of 3,3-diarylallyl phthalimides **3** in good yields.

**Scheme 1:**
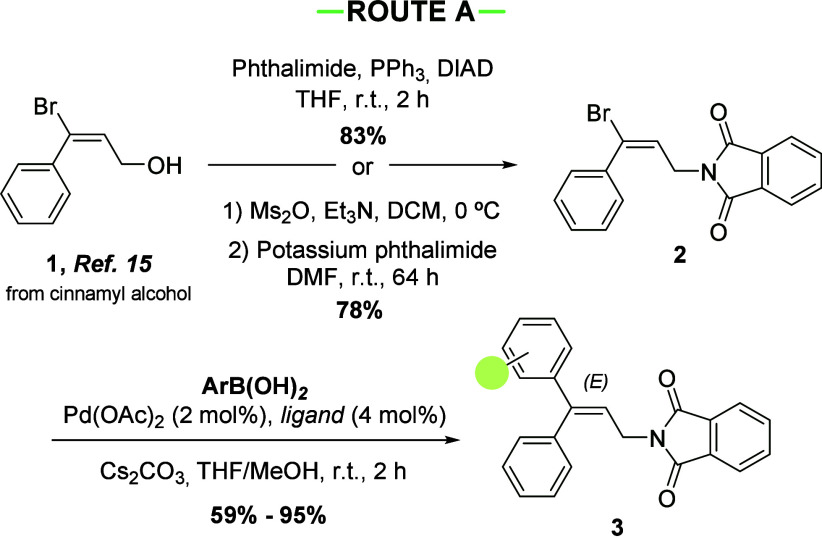
Synthesis of 3,3-Diarylallyl Phthalimides **3** by Route A

The initial catalyst screening for AH was carried out using different catalysts developed by our group, like the Ir–MaxPHOX^16^ and Ir–PepPHOX^17^ families, on substrate **3a** (Scheme 2). Although these catalysts afforded excellent results, the best enantioselectivity (99% ee) was obtained using commercially available Ir–(*S*,*S*)-UbaPHOX.^18^ For full details, see the Supporting Information (SI).

**Scheme 2:**
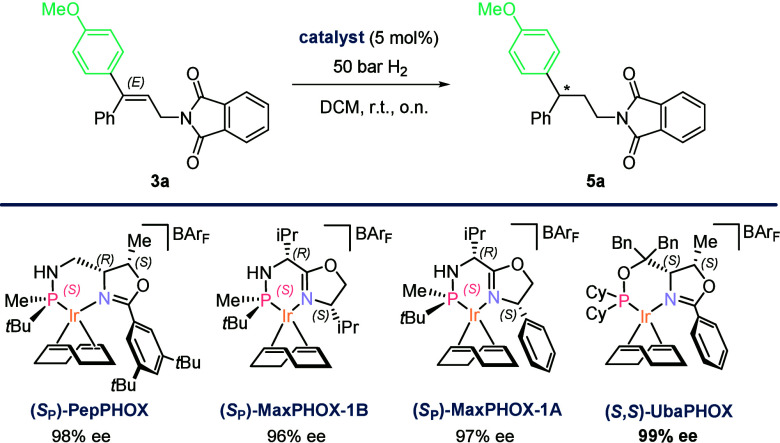
Summarized Screening of Catalysts for the AH of **3a** The catalysts shown afforded full conversion. See the SI for the complete catalyst screening results.

Next, we studied the optimization of the hydrogenation conditions with *p*-methyl-substituted substrate **3b** and Ir-UbaPHOX catalyst (Table 1). Dichloromethane (DCM), trifluorotoluene (TFT), and dichloroethane (DCE) all provided 96–97% ee (Table 1, entries 1–3). Toluene also provided comparable selectivity but with a significant loss of activity (Table 1, entry 4). The use of a weakly coordinating solvent such as ethyl acetate (EtOAc) was detrimental in terms of conversion (Table 1, entry 5). The use of greener solvents such as dimethyl carbonate (DMC) and propylene carbonate (PC) was also attempted without success due to the poor solubility of the allyl phthalimide (Table 1, entries 6 and 7). Regarding the hydrogen pressure, the best results were obtained at 50 bar (Table 1, entry 1). Decreasing the pressure to 10 bar resulted in a slight decrease in selectivity (Table 1, entry 8), and when the pressure was set at 3 bar, the reaction did not reach full conversion (Table 1, entry 9). Finally, the catalyst loading was decreased to 1 mol % with no loss of selectivity (Table 1, entry 10).

**Table 1:**
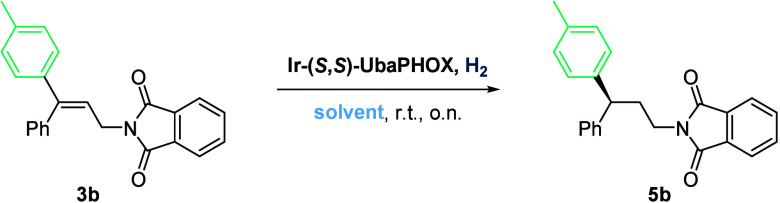
Optimization of Pressure, Solvent, and Catalyst Loading Parameters^a^

entry	catalyst loading [mol %]	*P*_H_2__ [bar]	solvent	conv. [%]^b^	ee [%]^c^
1	5	50	DCM	>99	96
2	5	50	TFT	>99	97
3	5	50	DCE	>99	96
4	5	50	toluene	62	95
5	5	50	EtOAc	3	–
6	5	50	DMC	0	–
7	5	50	PC	0	–
8	5	10	DCM	>99	91
9	5	3	DCM	53	57
10	1	50	DCM	>99	96
11^d^	1	50	DCM	>99	98

a The experiments were carried out at 0.17 M.

b Determined by ^1^H NMR analysis of the crude reaction mixtures.

c Determined by HPLC analysis on a chiral stationary phase.

d The starting material was synthesized via Route B (Scheme 3b).

While screening the reaction conditions with **3b**, we encountered a few reproducibility issues. In some instances, the hydrogenation was not complete, and the isomerized starting material *E*/*Z***-3b** was recovered. After closer inspection, we realized that this occurred with non-recrystallized samples of **3b**. HPLC-MS analysis of such batches revealed that they contained small amounts of the previous bromoalkene intermediate **2** (see the SI for more details). To confirm that the presence of the bromoalkene was responsible for the isomerization, a 1 mol % loading of **2** was added to a recrystallized batch of *E*-**3b** (Scheme 3a). Hydrogenation at 1 mol % catalyst in this case was completely suppressed, and the isomerized alkene was recovered. This observation confirmed that any bromoalkene impurity was extremely detrimental for the conversion and selectivity of the AH process. To avoid the presence of **2**, we tackled the synthesis of alkene substrates **3** via an alternative route. Reversing the order of the reactions, the arylboronic acids were introduced first via a Suzuki coupling, and the phthalimide group was incorporated later using a Mitsunobu reaction (Scheme 3b). After column chromatography and/or recrystallization, the desired *E*-3,3-diarylallyl phthalimides **3** were obtained as single diastereomers. Notably, the AH of **3a** synthesized via Route B resulted in an increase in selectivity from 96% to 98% ee (Table 1, entry 11).

**Scheme 3:**
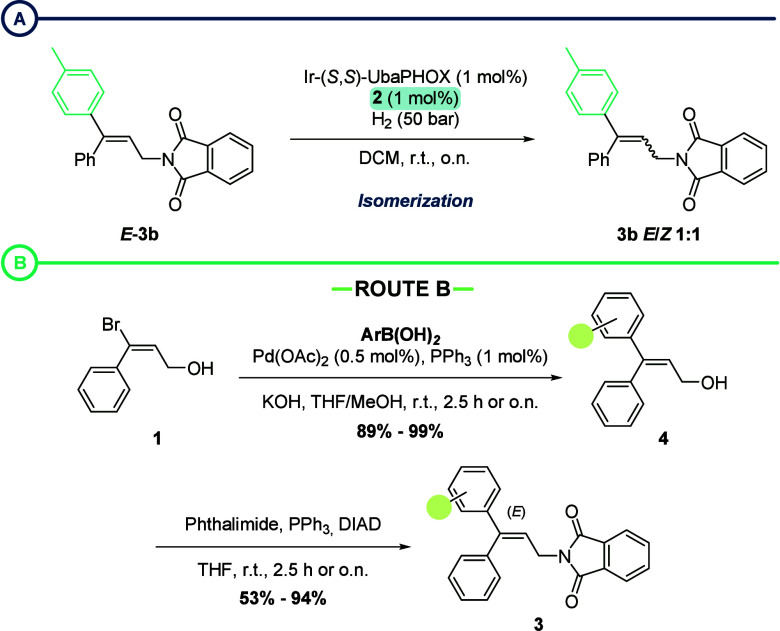
(a) Isomerization Induced by the Presence of **2**; (b) Synthesis of 3,3-Diarylallyl Phthalimides **3** by Route B

Using both routes, a set of 3,3-diarylallyl phthalimides with different substituents on one of the aryl groups (**3a**–**3j**) were prepared. These substrates were subjected to AH at 1 mol % under the optimized conditions (Scheme 4). All olefins bearing *para* substituents (**3a**–**3d**) on the aryl ring gave enantioselectivities ranging from 98% to 99% ee. When this substitution was placed at the *meta* position (**3e**), 98% ee was achieved. Example **3f** with *ortho* substitution yielded 99% ee. Similarly, **3g** also provided 99% ee but required a longer reaction time and an increase in the catalyst loading. The transformation also proved to be effective with disubstituted compounds. In this regard, **3h** and **3i** were successfully hydrogenated, achieving 99% ee. Finally, the naphthyl substituted substrate **3j** was also attempted, yielding 98% ee. It was observed that compounds synthesized through Route B consistently provided higher selectivity. This observation confirmed that trace amounts of **2** that remained on substrate **3** were responsible for partial isomerization of the substrate, thus resulting in a decrease in selectivity.^19^ Hydrogenation of substrates containing acetyl, furan, and thiophene moieties provided low conversion and selectivity (see the SI). This is most likely due to coordination of these moieties to the iridium center, resulting in catalyst deactivation.

**Scheme 4:**
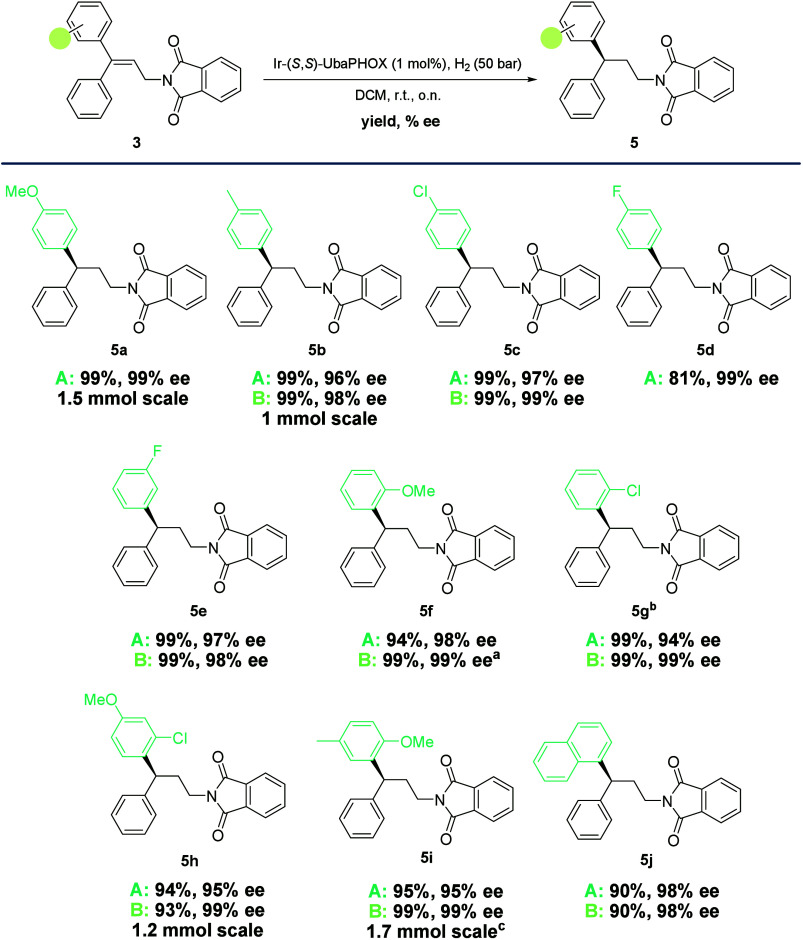
Scope of the Catalytic Hydrogenation of 3,3-Diarylallyl Phthalimides **3** 98% ee was obtained when the catalyst loading was decreased to 0.5 mol %. 2 mol % catalyst loading and 64 h reaction time were used. Large-scale hydrogenation was performed with Ir–(*R*,*R*)-UbaPHOX to yield (*R*)-**5i**. See the SI for the molarity values used in each reaction. All substrates provided complete conversion, except for **5h** (Route B; 97%). The ee values were determined by HPLC analysis on a chiral stationary phase.

Examples **3a**, **3b**, **3g**, **3h**, and **3i** were also hydrogenated on larger scales ranging from 0.5 to 1.7 mmol (150–650 mg) of starting material without loss of selectivity. Example **3f** was also hydrogenated at a 0.5 mol % catalyst loading with a minimal decrease in enantioselectivity (98% ee). The stereochemistry of all the products was predicted to be *S* using Andersson’s quadrant model (see the SI).^20^ This was later confirmed by comparison of the sign of the optical rotation of **10g** (*vide infra*) with the literature.^21^ The stereochemical outcome was assumed to be the same for all substrates.

We next proceeded to demonstrate the usefulness of the present methodology by applying it to the synthesis of biologically active compounds of pharmacological interest (Scheme 5). First, the deprotected primary amine derivatives of **5** were readily obtained in quantitative yield by phthalimide deprotection using hydrazine (Scheme 5a). (*R*)-Tolterodine is a commercially available drug that has been synthesized on numerous occasions using racemic resolution,^22^ chiral auxiliaries,^23^ rhodium-catalyzed 1,4-conjugate addition,^9^ or AH on coumarins.^24^ Nonetheless, none of these approaches contemplate iridium-catalyzed AH as the key step. Starting from (*R*)-**6i** (Scheme 5b), obtained using Ir–(*R*,*R*)-UbaPHOX, the free amine was alkylated with two isopropyl groups using acetone and Pd/C under H_2_ pressure to yield **7**. A final deprotection of the phenol group provided (*R*)-tolterodine (**8**) in optically pure form. Tolpropamine, an antihistaminic drug, has only been described as a racemate, and no asymmetric synthesis has been previously reported. Here, starting from **6b** (Scheme 5b), the free amine was dimethylated via an Eschweiler–Clarke reaction to yield (*S*)-tolpropamine (**9**).

**Scheme 5:**
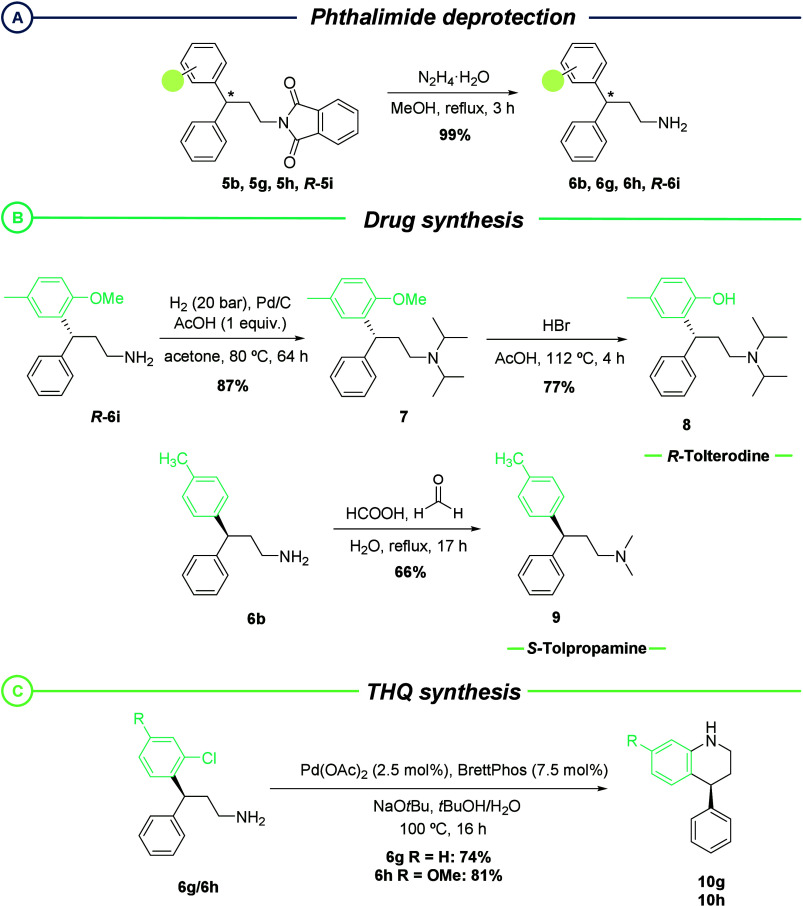
(a) Deprotection of Phthalimides **5**; (b) Asymmetric Synthesis of (*R*)-Tolterodine and (*S*)-Tolpropamine; (c) Cyclization of Hydrogenated Amines **6g**/**6h** to Provide THQs **10g**/**10h**

Ultimately, regarding the promising activity of 4-aryl THQs against biological targets, we envisioned their asymmetric synthesis from the cyclization of *o*-chloro-substituted 3,3-diarylpropyl phthalimides **5g**/**5h** (Scheme 5c). After deprotection of the phthalimides, a Buchwald–Hartwig cyclization yielded the desired 4-aryl THQs. Comparison of the optical rotation of **10g** with literature data confirmed not only the absolute configuration of the hydrogenation products but also that no racemization occurred during the deprotection and cyclization reactions.^20^ To the best of our knowledge, the approach described herein provides the best enantioselectivity in the synthesis of such compounds reported to date. All of these applications demonstrate the versatility of the chiral diarylpropyl amine intermediates obtained using our methodology.

In summary, here we describe a novel methodology to prepare 3,3-diarylallyl phthalimides **3** as single diastereomers. Iridium-catalyzed asymmetric hydrogenation of these compounds provides the corresponding 3,3-diarylpropyl amines with high enantioselectivity. During optimization of the reaction, it was found that bromoalkene impurities induced the isomerization of the alkene starting material, thus lowering the selectivity of the overall process. Using a synthetic route that minimizes the bromoalkene impurities in the starting material, the final chiral propylamines were obtained with selectivity ranging from 98 to 99% ee. The scope of the reaction has been shown to tolerate distinct functional groups and substitutions patterns. The synthetic utility of 3,3-diarylpropyl phthalimides **5** has been proven by preparing tolpropamine, tolterodine, and 4-aryl THQs, achieving the highest enantioselectivities reported to date.

## Data Availability

The data underlying this study are available in the published article and its online Supporting Information.

## Supporting Information Available

Screening of catalysts for the AH; HPLC-MS chromatogram of non-recrystallized **3b**; experimental procedures and spectroscopic data; ^1^H, ^13^C, and ^19^F NMR spectra; and HPLC chromatograms of racemic and enantioenriched compounds (PDF)

## References

[ref1] aHöhne M.; Bornscheuer U. T. Biocatalytic Routes to Optically Active Amines. ChemCatChem 2009, 1, 42–51. 10.1002/cctc.200900110.

[ref2] aHodgson D. M.; Gibbs A. R.; Lee G. P. Enantioselective Desymmetrisation of Achiral Epoxides. Tetrahedron 1996, 52, 14361–14384. 10.1016/0040-4020(96)00888-5.

[ref3] Stereoselective Formation of Amines; Wei L., Zhang X., Eds.; Topics in Current Chemistry, Vol. 343; Springer, 2014. 10.1007/128_2013_492 24233253

[ref4] aComprehensive Asymmetric Catalysis; Jacobsen E. N., Pfaltz A., Yamamoto H., Eds.; Springer, 2004.

[ref5] aKim A. N.; Stoltz M. Recent Advances in Homogeneous Catalysts for the Asymmetric Hydrogenation of Heteroarenes. ACS Catal. 2020, 10, 13834–13851. 10.1021/acscatal.0c03958. 34567830 PMC8460131

[ref6] Staskin D.; Herschorn S.; Fialkov J.; Tu L. M.; Walsh T.; Schermer C. R. A Prospective, Double-Blind, Randomized, Two-Period Crossover, Multicenter Study to Evaluate Tolerability and Patient Preference between Mirabegron and Tolterodine in Patients with Overactive Bladder (PREFER Study). Int. Urogynecol. J. 2018, 29, 273–283. 10.1007/s00192-017-3377-5. 28620791 PMC5780540

[ref7] Oishi M.; Takano Y.; Torita Y.; Malhotra B.; Chiba K. Physiological Based Pharmacokinetic Modeling to Estimate in Vivo Ki of Ketoconazole on Renal P-Gp Using Human Drug-Drug Interaction Study Result of Fesoterodine and Ketoconazole. Drug. Metab. Pharmacokinet. 2018, 33, 90–95. 10.1016/j.dmpk.2017.11.005. 29338933

[ref8] aRomero-Bohórquez A. R.; Kouznetsov V. V.; Zacchino S. A. Synthesis and in Vitro Evaluation of Antifungal Properties of Some 4-Aryl-3-Methyl-1,2,3,4-Tetrahydroquinolines Derivatives. Univ. Sci. 2014, 20, 177–189. 10.11144/Javeriana.SC20-2.siea.

[ref9] aPaquin J.-F.; Stephenson C. R. J.; Defieber C.; Carreira E. M. Catalytic Asymmetric Synthesis with Rh–Diene Complexes: 1,4-Addition of Arylboronic Acids to Unsaturated Esters. Org. Lett. 2005, 7, 3821–3824. 10.1021/ol051533l. 16092884

[ref10] aTokunaga N.; Hayashi T. Highly Enantioselective 1,4-Addition of Arylzinc Reagents to 3-Arylpropenals Catalyzed by a Rhodium–Binap Complex in the Presence of Chlorotrimethylsilane. Tetrahedron: Asymmetry 2006, 17, 607–613. 10.1016/j.tetasy.2006.01.036.

[ref11] aWang X.; Guram A.; Caille S.; Hu J.; Preston J. P.; Ronk M.; Walker S. Highly Enantioselective Hydrogenation of Styrenes Directed by 2′-Hydroxyl Groups. Org. Lett. 2011, 13, 1881–1883. 10.1021/ol200422p. 21384895

[ref12] aTolstoy P.; Engman M.; Paptchikhine A.; Bergquist J.; Church T. L.; Leung A. W.-M.; Andersson P. G. Iridium-Catalyzed Asymmetric Hydrogenation Yielding Chiral Diarylmethines with Weakly Coordinating or Noncoordinating Substituents. J. Am. Chem. Soc. 2009, 131, 8855–8860. 10.1021/ja9013375. 19552449

[ref13] Cabré A.; Romagnoli E.; Martínez-Balart P.; Verdaguer X.; Riera A. Highly Enan-tioselective Iridium-Catalyzed Hydrogena-tion of 2-Aryl Allyl Phthalimides. Org. Lett. 2019, 21, 9709–9713. 10.1021/acs.orglett.9b03865. 31702157

[ref14] Meindl W. Antimykobakterielle Antihistaminika. Arch. Pharm. 1989, 322, 493–497. 10.1002/ardp.19893220808. 2513793

[ref15] Limberger J.; Claudino T. S.; Monteiro A. L. Stereoselective Synthesis of (*E*)-3,3-Diaryl and (*E*)-3-Aryl-3-Aryloxy Allylamines and Allylalcohols from Trans-Cinnamyl Chloride and Alcohol. RSC Adv. 2014, 4, 45558–45565. 10.1039/C4RA08036J.

[ref16] aOrgué S.; Flores-Gaspar A.; Biosca M.; Pàmies O.; Diéguez M.; Riera A.; Verdaguer X. Chem. Commun. 2015, 51, 17548–17551. 10.1039/C5CC07504A. 26477668

[ref17] Rojo P.; Molinari M.; Cabré A.; García-Mateos C.; Riera A.; Verdaguer X. Iridium-Catalyzed Asymmetric Hydrogenation of 2,3-Diarylallyl Amines with a Threonine-Derived P-Stereogenic Ligand for the Synthesis of Tetrahydroquinolines and Tetrahydroisoquinolines. Angew. Chem., Int. Ed. 2022, 61, e202204300 10.1002/anie.202204300. PMC9400882 35543384

[ref18] Pfaltz A.; Blankenstein J.; Hilgraf R.; Hörmann E.; McIntyre S.; Menges F.; Schönleber M.; Smidt S.; Wüstenberg B.; Zimmermann N. Iridium-Catalyzed Enantioselective Hydrogenation of Olefins. Adv. Synth. Catal. 2003, 345, 33–43. 10.1002/adsc.200390027.

[ref19] We believe that the presence of **2** in the reaction mixture results in an oxidative addition reaction on iridium. This should lead to a new metal complex that efficiently catalyzes the isomerization but not hydrogenation

[ref20] aKällström K.; Hedberg C.; Brandt P.; Bayer A.; Andersson P. G. Rationally Designed Ligands for Asymmetric Iridium-Catalyzed Hydrogenation of Olefins. J. Am. Chem. Soc. 2004, 126, 14308–14309. 10.1021/ja0464241. 15521722

[ref21] Rueping M.; Theissmann T.; Stoeckel M.; Antonchick A. P. Direct Enantioselective Access to 4-Substituted Tetrahydroquinolines by Catalytic Asymmetric Transfer Hydrogenation of Quinolines. Org. Biomol. Chem. 2011, 9, 6844–6850. 10.1039/c1ob05870c. 21837348

[ref22] aDe Castro K. A.; Ko J.; Park D.; Park S.; Rhee H. Reduction of Ethyl Benzoylacetate and Selective Protection of 2-(3-Hydroxy-1-Phenylpropyl)-4-Methylphenol: A New and Facile Synthesis of Tolterodine. Org. Process Res. Dev. 2007, 11, 918–921. 10.1021/op7001134.

[ref23] aAndersson P. G.; Schink H. E.; Österlund K. Asymmetric Total Synthesis of (+)-Tolterodine, a New Muscarinic Receptor Antagonist, via Copper-Assisted Asymmetric Conjugate Addition of Aryl Grignard Reagents to 3-Phenyl-Prop-2-Enoyl-Oxazolidinones. J. Org. Chem. 1998, 63, 8067–8070. 10.1021/jo981259r.

[ref24] aHedberg C.; Andersson P. Catalytic Asymmetric Total Synthesis of the Muscarinic Receptor Antagonist (*R*)-Tolterodine. Adv. Synth. Catal. 2005, 347, 662–666. 10.1002/adsc.200404234.

