# Supplementary material for: Highly Enantioselective Synthesis of 3,3-Diarylpropyl Amines and 4-Aryl Tetrahydroquinolines via Ir-Catalyzed Asymmetric Hydrogenation
Source: Org Lett. 2024 Dec 5;26(50):10903–9. doi: 10.1021/acs.orglett.4c04076 (PMC11668043; doi:10.1021/acs.orglett.4c04076)
Supplement: Supplementary file 1 — ol4c04076_si_001.pdf [file ol4c04076_si_001.pdf]

Highly Enantioselective Synthesis of 3,3-Diarylpropyl Amines and 4-Aryl Tetrahydroquinolines via Ir-Catalyzed Asymmetric Hydrogenation.

Martí Sidro[†,‡], Clara García-Mateos[†,‡], Pep Rojo[†,‡], Yisong Wen[†,‡] Antoni Riera[*,†,‡] and Xavier Verdaguer[*,†,‡]

[†]Institute for Research in Biomedicine (IRB Barcelona), Barcelona Institute of Science and Technology (BIST), Baldiri Reixach 10, 08028 Barcelona (Spain)

[‡]Departament de Química Inorgànica i Orgànica, Secció Química Orgànica, Universitat de Barcelona, Martí i Franquès 1, 08028 Barcelona (Spain)

Contact e-mail: xavier.verdaguer@irbbarcelona.org

Supporting Information

Table of contents

1. **General information S2**
2. **Full screening of catalysts for the AH S3**
3. **HPLC-MS chromatogram of 3b S4**
4. **Experimental procedures and characterization................................ S6**
5. **Stereochemical course of the AH S36**
6. **Failed asymmetric hydrogenations S37**
7. **References S38**
8. **NMR spectra of compounds S39**
9. **Chiral HPLC chromatograms S79**

1. General information

Unless otherwise indicated, materials were obtained from commercial suppliers and used without further purification. All reactions that required anhydrous conditions were performed in dried glassware under inert nitrogen atmosphere. Anhydrous THF and DCM were taken from a solvent purification system (SPS PS-MD-3). Other anhydrous solvents were purchased from Sigma Aldrich. Solvents were removed using a rotary evaporator. Silica gel chromatography was performed using an automated chromatography system (PuriFlash® 430, Interchim). Unless otherwise specified, starting materials were obtained from commercial sources. Hydrogenation reactions were carried out in a Büchi Glas Uster AG miniclave reactor or in a Berghof BR-100 reactor. Alternatively, reactions at low H_2 pressure were performed in Ace glass pressure tubes equipped with a gas inlet manifold and a manometer. For reactions that require heating an oil bath was used.

^{1}H and ^{13}C NMR spectra were recorded at 23 °C on the NMR spectrometers of the Centres Científics i Tecnològics de la Universitat de Barcelona. The employed spectrometers were Varian Mercury 400 MHz, Bruker 400 MHz and Bruker 500 MHz. ^{1}H NMR and ^{13}C NMR were referenced to internal solvent resonances or to TMS. Chemical shifts (δ) are expressed in ppm and the coupling constant (*J*) in Hertz (Hz). Multiplicities of the signals are reported as s (singlet), br (broad singlet), d (doublet), t (triplet), q (quartet), p (pentet), hept (heptet) and m (multiplet).

High resolution ESI-MS spectra were recorded in an LC/MSD-TOF G1969A (Agilent Technologies) of Centres Científics i Tecnològics de la Universitat de Barcelona or in a LTQ-FT Ultra (Thermo Scientific) of Institute for Research in Biomedicine (IRB Barcelona). **IR** spectra were recorded in a Thermo Nicolet 6700 FT-IR spectrometer using an ATR system of the Departament de Química Orgànica i Inorgànica of Universitat de Barcelona. Absorptions are given in wavenumbers (cm^{-1}). **Melting points** were determined using a Büchi M-540 apparatus.

HPLC analyses were performed on an Agilent Technologies Series 1100 chromatograph with UV detector by Enantia S.L. The conditions for each analysis are specified in every case. **Optical rotations** were measured at 28 °C using a Jasco P-2000 iRM-800 polarimeter. Concentration is expressed in g/100 mL. The cell sized 10 cm long and had 2 mL of capacity, measuring λ was 589 nm, which corresponds to a sodium lamp.

2. Full screening of catalysts for the AH

Unless otherwise specified full conversion was obtained. The best results are highlighted in bold.

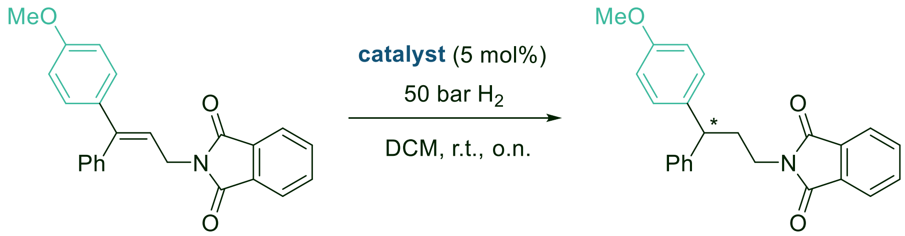

3a **5a**

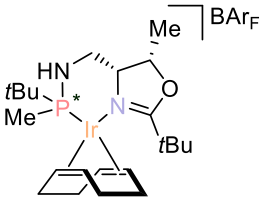

(R_P): 93% conv., 52% ee
(S_P): 89% conv., 51% ee

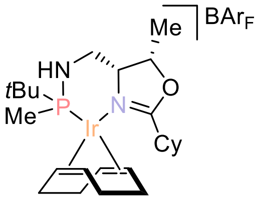

(R_P): 89% ee
(S_P): 94% ee

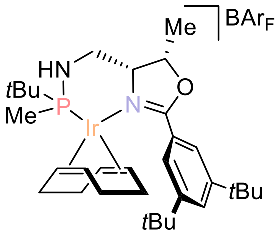

(R_P): 87% ee
PepPHOX
(S_P): **98% ee**

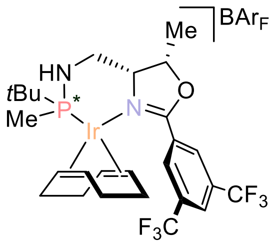

(R_P): 90% ee
(S_P): 94% ee

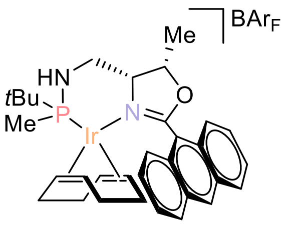

(R_P): 81% ee
(S_P): 89% ee

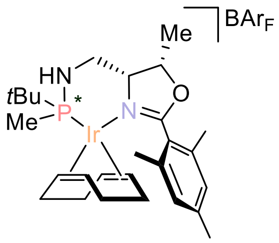

(R_P): 80% ee
(S_P): 95% ee

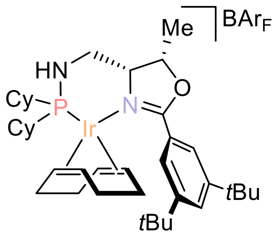

94% ee

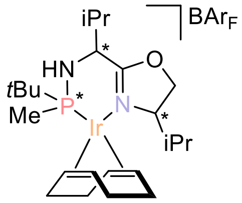

MaxPHOX-B
(S_P,R,S): **96% ee**
(S_P,S,S): 31% ee
(S_P,R,R): 77% ee
(S_P,S,R): 90% ee

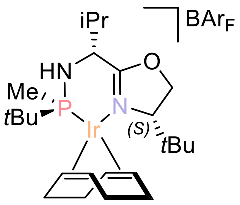

MaxPHOX-1C
(S_P,R,S): 91% ee

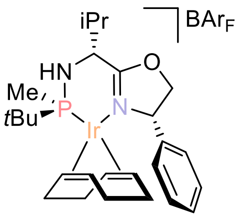

MaxPHOX-1A
(S_P,R,S): **97% ee**

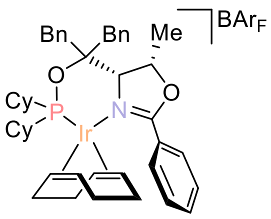

UbaPHOX
99% ee

3. HPLC-MS chromatogram of 3b

HPLC-MS: Kinetex EVO C18 50 x 4.6mm, 2.6um; Mobile phase: 10mM NH_4HCO_3 pH8 / ACN (95:5)---0.5 min---(95:5)---6.5 min----(0:100)---2 min---(0:100) post run 1.5min, λ = 210 nm, T = 40 °C. , positive ES-API.

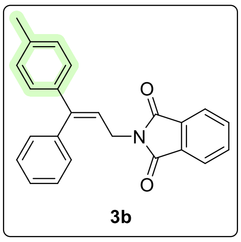

Molecular Weight: 353,42

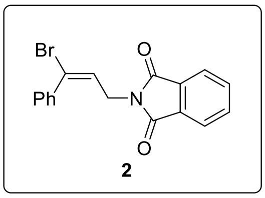

Molecular Weight: 342,19

Non-recrystallized batch – contaminated with 1.67% of 2

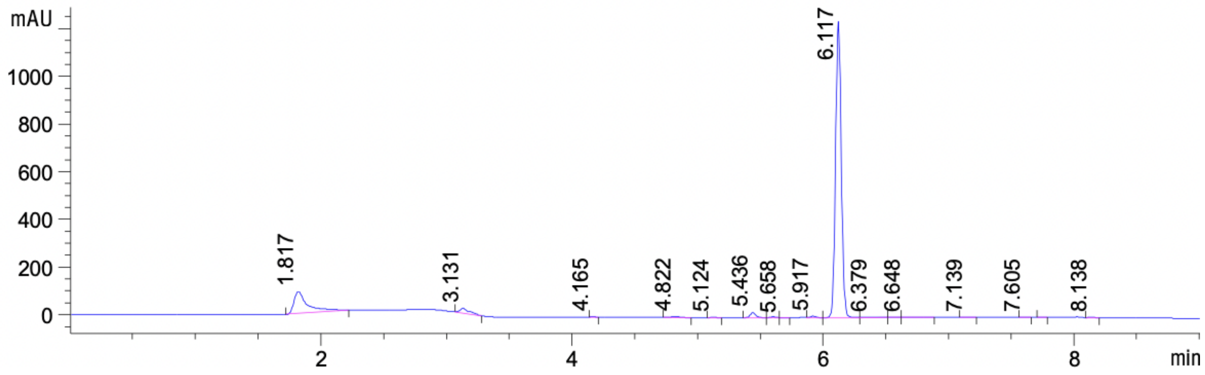

#	Meas. Ret. Time	Peak Type	Area	Area %
1	1.817	MM	696.07	14.26
2	3.131	MM	110.90	2.27
3	4.165	FM	5.65	0.12
4	4.822	VB	22.33	0.46
5	5.124	MM	2.26	0.05
6	5.436	BV	66.07	1.35
7	5.597	MF	11.47	0.23
8	5.658	FM	1.34	0.03
9	5.917	VB	19.88	0.41
10	6.117	BV	3901.98	79.94
11	6.379	VV	21.07	0.43
12	6.550	MF	6.62	0.14
13	6.648	FM	4.42	0.09
14	7.139	BB	3.45	0.07
15	7.605	MM	1.20	0.02
16	7.744	MM	1.44	0.03
17	8.138	BB	5.24	0.11

5.45min

MSD1 TIC, MS File

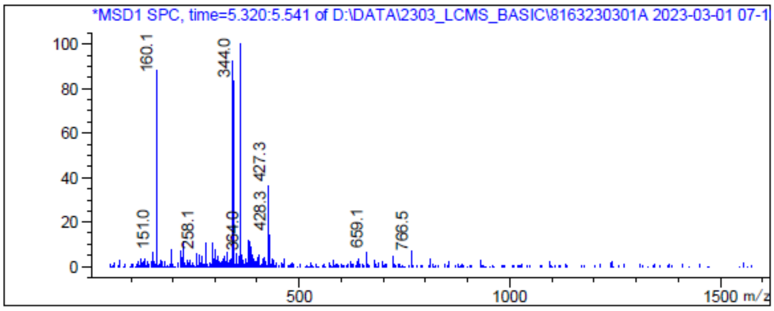

6.14min

MSD1 TIC, MS File

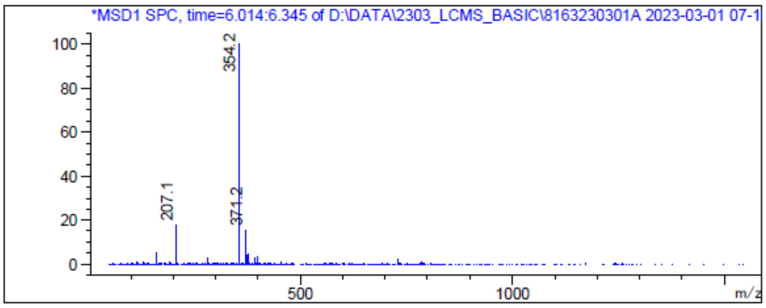

Recrystallized batch – contaminated with 0.18% of 2

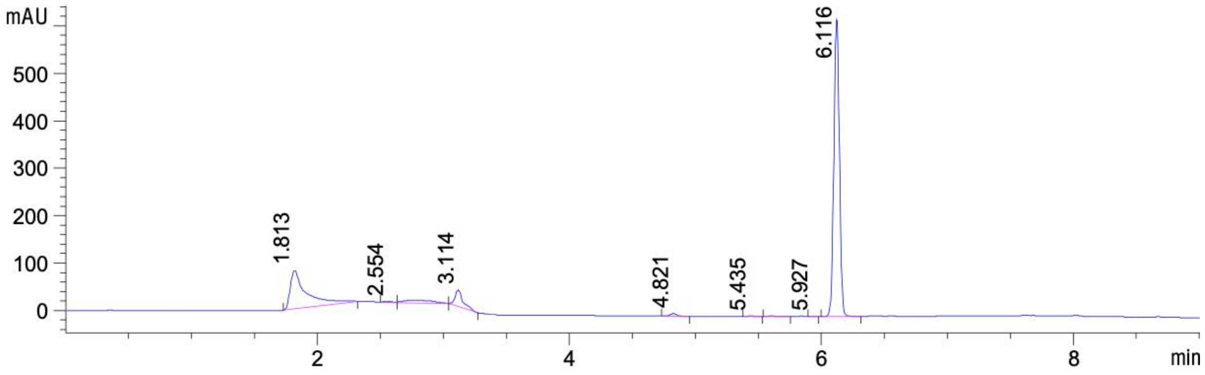

#	Meas. Ret. Time	Peak Type	Area	Area %
1	1.813	MM	741.76	24.97
2	2.554	MF	11.19	0.38
3	2.774	FM	97.82	3.29
4	3.114	MM	159.21	5.36
5	4.821	VB	21.94	0.74
6	5.435	VB	3.60	0.12
7	5.597	BB	6.04	0.20
8	5.927	MM	1.38	0.05
9	6.116	BV	1928.16	64.90

4. Experimental procedures and characterization

General synthetic scheme for the synthesis of substrate 1:

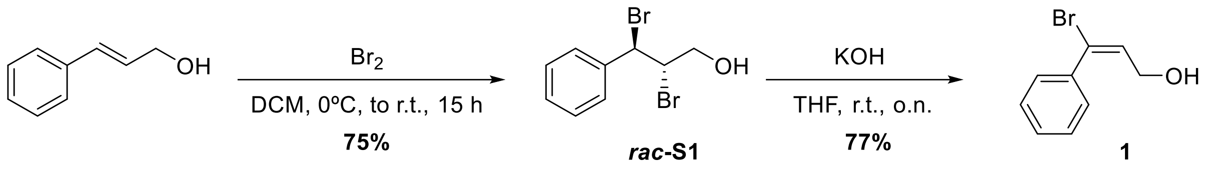

Preparation of rac-2,3-Dibromo-3-phenylpropan-1-ol (rac-S1)

Based on a literature procedure,[1] a 2L round-bottom flask was charged with (*E*)-cinnamyl alcohol (20.0 g, 0.15 mol, 1.0 equiv.) and dissolved in DCM (500 mL, 0.30 M). The mixture was cooled down to 0 °C and a solution of Br_2 (9.2 mL, 0.18 mol, 1.2 equiv.) in DCM (150 mL, 1.2 M) was added dropwise over 30 minutes. Then, the ice bath was removed, and the solution was left to stir at room temperature for 15 h. Upon completion, $NaHSO_3$ 10% was added, the layers were separated, and the organic phase was washed with brine. The organic phase was dried over anhydrous $MgSO_4$, filtered and concentrated under vacuum. The crude was recrystallized in hexanes/toluene (1:1) by heating at 70 °C and allowing the solution to reach room temperature. The crystals were filtered and washed twice with cold hexanes, affording pure ***rac*-S1** as a racemate (33.0 g, 75% yield, white solid).

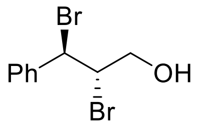

***rac*-S1**

^{1}H NMR (400 MHz, $CDCl_3$): δ 7.17 – 7.58 (m, 5H), 5.27 (d, *J* = 11.0 Hz, 1H), 4.71 (ddd, *J* = 11.0, 4.3, 2.8 Hz, 1H), 4.21 – 4.35 (m, 2H), 2.18 (br, 1H). Spectroscopic analysis matches with previously reported data.[1]

Preparation of (E)-3-Bromo-3-phenylprop-2-en-1-ol (1)

Based on a literature procedure,[1] a mixture of *rac*-**S1** (12.6 g, 42.3 mmol, 1.0 equiv.), KOH (5.6 g, 99.8 mmol, 2.0 equiv.) and THF (214 mL, 0.20 M) was stirred at room temperature overnight. The mixture was then filtered and concentrated under reduced pressure. Purification of the crude by flash chromatography (hexanes/EtOAc 8:2) afforded the desired product **1** in 98:2 *E*:*Z* ratio (7.07 g, 77% yield, pale yellow solid).

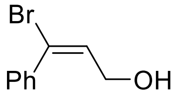

1

^{1}H NMR (400 MHz, $CDCl_3$): δ 7.28 – 7.46 (m, 5H), 6.43 (t, *J* = 7.2 Hz, 1H), 4.08 (d, *J* = 7.2 Hz, 2H), 1.43 (s, 1H). Spectroscopic analysis matches with previously reported data.[1]

Preparation of (E)-2-(3-Bromo-3-phenylallyl)isoindoline-1,3-dione (2)

Two different procedures were employed to synthesize ***2****:*

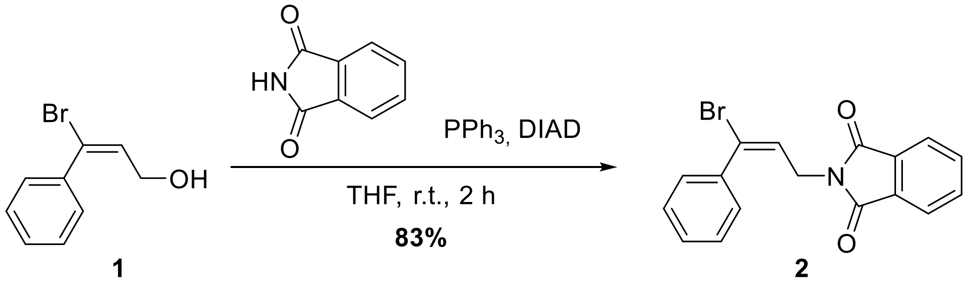

Procedure I: A round-bottom flask was charged with **1** (3.45 g, 16.2 mmol, 1.0 equiv.), PPh_3 (5.80 g, 21.0 mmol, 1.3 equiv.) and phthalimide (3.13 g, 21.0 mmol, 1.3 equiv.), purged with vacuum-nitrogen cycles and dissolved in THF (54 mL, 0.30 M). Then, the reaction was cooled down to 0 °C and DIAD (4.18 mL, 21.0 mmol, 1.3 equiv.) was added over 30 minutes with a syringe pump. After stirring the reaction for 2 h at room temperature, the solvent was evaporated and the crude was purified by flash chromatography (hexanes/EtOAc 9:1) affording **2** as a single diastereomer (4.61 g, 83% yield, white solid).

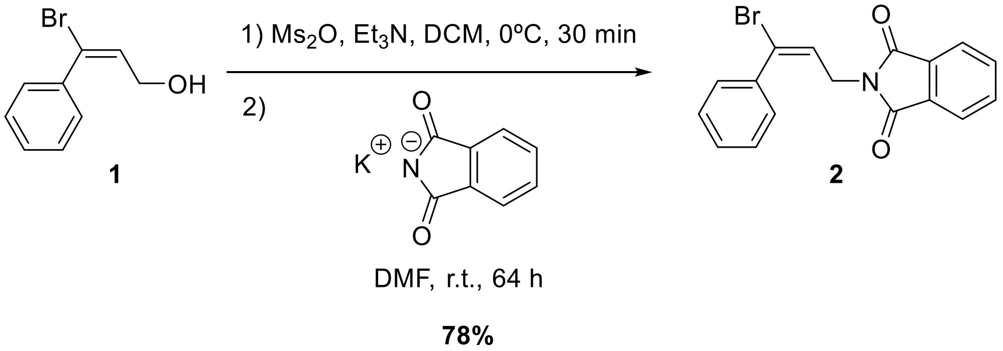

Procedure II: To a round-bottom flask charged with **1** (12.10 g, 41.3 mmol, 1 equiv.), anhydrous DCM (100 mL, 0.4 M) and Et_3N (9.5 mL, 68.2 mmol, 1.6 equiv.), a solution of Ms_2O (11.9 g, 68.3 mmol, 1.6 equiv.) in DCM (100 mL, 0.7 M) was added dropwise at 0 °C. The resulting mixture was stirred at 0 °C for 30 minutes. Upon completion, the solution was warmed up to room temperature, H_2O added, and phases separated. The aqueous phase was extracted with DCM 2 times, all organic layers collected, dried over anhydrous $MgSO_4$ and concentrated under reduced pressure. The obtained orange oil was used without further purification.

A round-bottom flask charged with the previous orange oil (16.92 g, 45.5 mmol, 1 equiv.), potassium phthalimide (12.90 g, 69.6 mmol, 1.5 equiv.) and DMF (300 mL, 0.19 M) was stirred at room temperature over-weekend. Upon completion, the solvent was removed under reduced pressure, EtOAc added, and the mixture extracted with H_2O. The organic layer was then washed 3 times with brine. To remove potassium phthalimide remaining traces the mixture was dissolved in DCM, NaOH 0.5 M added, stirred for 5 minutes and both phases separated. The obtained organic layer was washed again with NaOH 0.5 M, dried over anhydrous $MgSO_4$, filtered, and concentrated under reduced pressure. The crude was recrystallized in hexanes/toluene (3:1) by heating to reflux and allowing the solution to reach room temperature. The crystals were filtered and washed twice with cold hexanes, affording **2** as a single diastereomer (15.58 g, 78% yield, white solid).

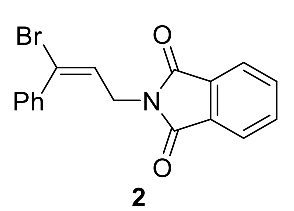

2

^{1}H NMR (400 MHz, CDCl$_3$): δ 7.77 – 7.89 (m, 2H), 7.65 – 7.75 (m, 2H), 7.48 (d, *J* = 7.5 Hz, 2H), 7.41 (t, *J* = 7.5 Hz, 2H), 7.29 – 7.37 (m, 1H), 6.25 (t, *J* = 6.8 Hz, 1H), 4.25 (d, *J* = 6.8 Hz, 2H). **^{13}C NMR (101 MHz, CDCl3):** δ 167.8, 137.8, 134.2, 132.1, 129.2, 129.0, 128.6, 127.4, 125.2, 123.5, 37.6. **HRMS (ESI) m/z:** [M+Na]$^+$ Calcd for $C_{17}H_{12}BrNO_2Na$ 363.9944; Found 363.9947. **IR υ_{max}:** 3048, 3023, 2925, 2021, 1769, 1698 cm^{-1}. **Melting point:** 103 – 104 °C.

4.1. General procedure 1: Synthesis of 3,3-diarylallyl alcohols 4 (Route B).

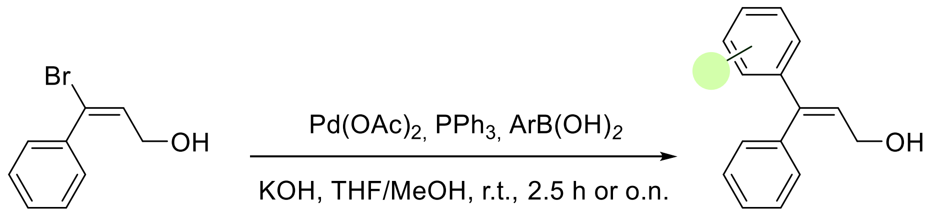

Procedure adapted from Monteiro and co-workers.[1]

To an oven dried and N_2 purged Schlenk flask charged with **1** (1 equiv.), $Pd(OAc)_2$ (0.5 mol%), PPh_3 (1 mol%), the corresponding *arylboronic acid* (1.1 equiv.) and KOH 85% (2 equiv.) were added. The flask was purged again with vacuum/N_2 cycles and a mixture of anhydrous-degassed MeOH/THF (1:1, 0.13 M) was added. The resulting mixture was stirred at room temperature for 2.5 h or overnight to reach full conversion (checked by TLC). Upon completion, the solution was diluted

with EtOAc, extracted with NaOH 1 M, washed with brine, dried over anhydrous $MgSO_4$, filtered, and concentrated under vacuum. Purification of the crude by flash chromatography (gradient from 0% to 10% of EtOAc in Cy) afforded the desired product in a 98:2 *E/Z* diastereomeric ratio.

Preparation of (E)-3-phenyl-3-(p-tolyl)prop-2-en-1-ol (4b)

Following **GP1**, **1** (426.2 mg, 2.0 mmol, 1 equiv.), *p*-tolylboronic acid (299.1 mg, 2.2 mmol, 1.1 equiv.), $Pd(OAc)_2$ (2.3 mg, 0.01 mmol, 0.5 mol%), PPh_3 (5.3 mg, 0.02 mmol, 1 mol%), and KOH (264.0 mg, 4.0 mmol, 2 equiv.) were used. The reaction was stirred for 2.5 h. After work-up and purification, **4b** was obtained (405.7 mg, 90%, colourless oil).

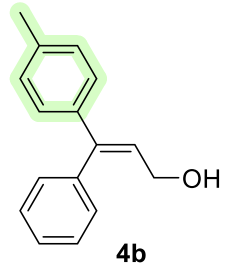

4b

^{1}H NMR (500 MHz, $CDCl_3$): δ 7.30 – 7.40 (m, 3H), 7.12 – 7.22 (m, 4H), 7.07 – 7.12 (m, 2H), 6.22 (t, *J* = 6.9 Hz, 1H), 4.21 (dd, *J* = 6.9, 5.5 Hz, 2H), 2.34 (s, 3H), 1.31 – 1.33 (t, *J* = 5.5 Hz, 1H). **^{13}C NMR (126 MHz, $CDCl_3$):** δ 144.4, 139.4, 139.1, 137.6, 129.9, 129.1, 128.3, 127.7, 127.7, 126.7, 60.9, 21.3. **IR υ_{max}:** 3298, 3079, 3053, 3021, 2920, 2853, 2110, 1903, 1793, 1653, 1627, 1599, 1575, 1508, 1492, 1440, 1407, 1368, 1310, 1261, 1271, 1187 cm^{-1}. Spectroscopic analysis matches with previously reported data.[2]

Preparation of (E)-3-(4-chlorophenyl)-3-phenylprop-2-en-1-ol (4c)

Following **GP1**, **1** (319.6 mg, 1.5 mmol, 1 equiv.), 4-chlorophenylboronic acid (258.0 mg, 1.7 mmol, 1.1 equiv.), $Pd(OAc)_2$ (1.7 mg, 0.008 mmol, 0.5 mol%), PPh_3 (3.9 mg, 0.015 mmol, 1 mol%), and KOH (168.3 mg, 3.0 mmol, 2 equiv.) were used. The reaction was stirred overnight. After work-up and purification, **4c** was obtained (318.9 mg, 96%, white solid).

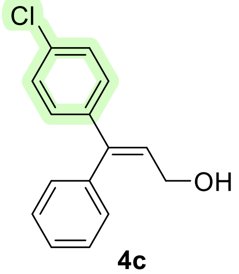

4c

^{1}H NMR (400 MHz, $CDCl_3$): δ 7.31 – 7.41 (m, 3H), 7.22 – 7.27 (m, 2H), 7.16 – 7.20 (m, 2H), 7.11 – 7.16 (m, 2H), 6.22 (t, J = 6.8 Hz, 1H), 4.21 (d, J = 6.8 Hz, 2H), 1.48 (br, 1H). **^{13}C NMR (101 MHz, $CDCl_3$):** δ 143.2, 140.4, 138.7, 133.6, 129.8, 129.0, 128.5, 128.5, 128.0, 127.9, 60.8. **HRMS (ESI) m/z:** $[M-OH]^+$ Calcd for $C_{15}H_{12}Cl$ 227.0622; Found 227.0629. **IR υ_{max}:** 3231, 3076, 2957, 2926, 2905, 2866, 2849, 1487, 1440, 1399, 1354, 1312, 1261, 1177, 1090 cm^{-1}. **Melting point:** 71 – 72 °C.

Preparation of (E)-3-(3-fluorophenyl)-3-phenylprop-2-en-1-ol (4e)

Following **GP1**, **1** (319.6 mg, 1.5 mmol, 1 equiv.), 3-fluorophenylboronic acid (230.9 mg, 1.7 mmol, 1.1 equiv.), $Pd(OAc)_2$ (1.7 mg, 0.008 mmol, 0.5 mol%), PPh_3 (3.9 mg, 0.015 mmol, 1 mol%), and KOH (168.3 mg, 3.0 mmol, 2 equiv.) were used. The reaction was stirred overnight. After work-up and purification, **4e** was obtained (330.8 mg, 90%, yellow oil).

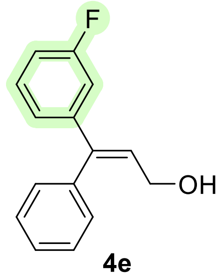

4e

^{1}H NMR (400 MHz, $CDCl_3$): δ 7.29 – 7.42 (m, 3H), 7.21 – 7.26 (m, 1H), 7.11 – 7.20 (m, 2H), 7.00 – 7.07 (m, 1H), 6.90 – 7.00 (m, 2H), 6.26 (t, J = 6.8 Hz, 1H), 4.21 (d, J = 6.8 Hz, 2H), 1.57 (br, 1H). **^{13}C NMR (101 MHz, $CDCl_3$):** δ 162.9 (d, *J* = 245.3 Hz), 144.2 (d, *J* = 7.3 Hz), 143.2 (d, *J* = 2.2 Hz), 138.6, 129.8 (d, *J* = 2.9 Hz), 129.7, 128.6, 128.5, 128.0, 123.3 (d, *J* = 2.9 Hz), 114.7 (d, *J* = 8.9 Hz), 114.5 (d, *J* = 8.0 Hz), 60.8. **HRMS (ESI) m/z:** $[M-OH]^+$ Calcd for $C_{15}H_{12}F$ 211.0918; Found 211.0922. **IR υ_{max}:** 3289, 3054, 302, 2924, 2864, 1653, 1608, 1578, 1481, 1441, 1364, 1340, 1265, 1220, 1190, 1131 cm^{-1}.

Preparation of (E)-3-(2-methoxyphenyl)-3-phenylprop-2-en-1-ol (4f)

Similarly to **GP1**, **1** (1.07 g, 5.0 mmol, 1 equiv.), 2-methoxyphenylboronic acid (835.8 mg, 5.5 mmol, 1.1 equiv.), $Pd(OAc)_2$ (5.6 mg, 0.03 mmol, 0.5 mol%), PPh_3 (13.1 mg, 0.05 mmol, 1 mol%), and KOH (660.1 mg, 10.0 mmol, 2 equiv.) were used. The reaction was stirred overnight. After work-up and purification, **4f** was obtained (1.18 g, 99%, colourless oil).

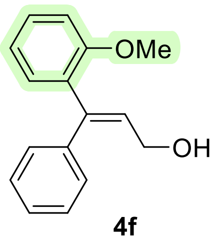

4f

^{1}H NMR (500 MHz, $CDCl_3$): δ 7.26 – 7.33 (m, 3H), 7.23 – 7.25 (m, 1H), 7.21 (dd, *J* = 7.5, 1.7 Hz, 1H), 7.13 – 7.18 (m, 2H), 6.94 (td, *J* = 7.5, 1.1 Hz, 1H), 6.85 (dd, *J* = 8.3, 1.1 Hz, 1H), 6.06 (t, *J* = 6.9 Hz, 1H), 4.31 – 4.33 (dd, *J* = 6.9, 3.2 Hz, 2H), 3.59 (s, 3H), 1.48 (br, 1H). **^{13}C NMR (126 MHz, $CDCl_3$):** δ 157.3, 142.1, 140.1, 132.5, 131.1, 130.2, 129.0, 129.0, 127.9, 127.2, 120.7, 111.7, 60.6, 55.7. **HRMS (ESI) m/z:** $[M-OH]^+$ Calcd for $C_{16}H_{15}O$ 223.1117; Found 223.1115. **IR υ_{max}:** 3311, 2054, 3021, 2931, 2873, 2832, 1811, 1735, 1718, 1701, 1684, 1615, 1595, 1576, 1487, 1459, 1433, 1293, 1239, 1179, 1161, 1112 cm^{-1}.

Preparation of (E)-3-(2-chlorophenyl)-3-phenylprop-2-en-1-ol (4g)

Similarly to **GP1**, **1** (213.0 mg, 1.0 mmol, 1 equiv.), 2-chlorophenylboronic acid (172.0 mg, 1.1 mmol, 1.1 equiv.), $Pd(OAc)_2$ (1.1 mg, 0.005 mmol, 0.5 mol%), PPh_3 (2.6 mg, 0.01 mmol, 1 mol%), and KOH (112.2 mg, 2.0 mmol, 2 equiv.) were used. The reaction was stirred overnight. After work-up and purification, **4g** was obtained (217.2 mg, 89%, colourless oil).

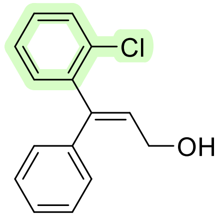

4g

^{1}H NMR (400 MHz, $CDCl_3$): δ 7.27 – 7.38 (m, 5H), 7.20 – 7.25 (m, 2H), 7.11 – 7.18 (m, 2H), 5.97 (t, *J* = 6.7 Hz, 1H), 4.41 (dd, *J* = 6.7, 5.6 Hz, 2H), 1.46 – 1.49 (t, *J* = 5.6 Hz). **^{13}C NMR (126 MHz, $CDCl_3$):** δ 142.1, 141.7, 138.5, 133.3, 131.5, 131.5, 129.9, 129.2, 128.7, 128.0, 127.6, 126.6, 60.3. **HRMS (ESI) m/z:** $[M-OH]^+$ Calcd for $C_{15}H_{12}Cl$ 227.062; Found 227.0624. **IR υ_{max}:** 3289, 3078, 3054, 3021, 2924, 2864, 1889, 1809, 1735, 1701, 1684, 1589, 1559, 1492, 1466, 1436, 1313, 1282, 1246, 1211, 1157, 1125 cm^{-1}.

Preparation of (E)-3-(2-chloro-4-methoxyphenyl)-3-phenylprop-2-en-1-ol (4h)

Following **GP1**, **1** (426.2 mg, 2.0 mmol, 1 equiv.), 2-chloro-4-methoxyphenylboronic acid (410.1 mg, 2.2 mmol, 1.1 equiv.), $Pd(OAc)_2$ (2.3 mg, 0.01 mmol, 0.5 mol%), PPh_3 (5.3 mg, 0.02 mmol, 1 mol%), and KOH (264.0 mg, 4.0 mmol, 2 equiv.) were used. The reaction was stirred for 2.5 h. After work-up and purification, **4h** was obtained (542.2 mg, 99%, yellowish oil).

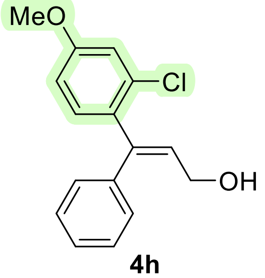

4h

^{1}H NMR (500 MHz, $CDCl_3$): δ 7.26 – 7.34 (m, 3H), 7.21 (d, *J* = 8.5 Hz, 1H), 7.10 – 7.16 (m, 2H), 6.90 (d, *J* = 2.6 Hz, 1H), 6.80 (dd, *J* = 8.5, 2.6 Hz, 1H), 5.95 (t, *J* = 6.7 Hz, 1H), 4.38 (dd, *J* = 6.7, 5.5 Hz, 2H), 3.80 (s, 3H), 1.44 – 1.46 (t, *J* = 5.5 Hz, 1H). **^{13}C NMR (126 MHz, $CDCl_3$):** δ 159.6, 141.9, 139.0, 134.3, 134.0, 132.3, 132.3, 131.3, 129.3, 128.1, 127.7, 115.2, 112.8, 60.5, 55.7. **HRMS (ESI) m/z:** $[M-OH]^+$ Calcd for $C_{16}H_{14}OCl$ 257.0733; Found 257.0728. **IR υ_{max}:** 3302, 3078, 3052, 3021, 2937, 2834, 1889, 1811, 1735, 1718, 1701, 1684, 1653, 1599, 1559, 1490, 1459, 1438, 1395, 1285, 1256, 1226, 1181, 1162 cm^{-1}.

Preparation of (E)-3-(2-methoxy-5-methylphenyl)-3-phenylprop-2-en-1-ol (4i)

Following **GP1**, **1** (1.01 g, 4.8 mmol, 1 equiv.), 2-methoxy-5-methylphenylboronic acid (870.0 mg, 5.2 mmol, 1.1 equiv.), $Pd(OAc)_2$ (5.3 mg, 0.02 mmol, 0.5 mol%), PPh_3 (12.5 mg, 0.05 mmol, 1 mol%), and KOH (628.4 mg, 9.5 mmol, 2 equiv.) were used. The reaction was stirred for 2.5 h. After work-up and purification, **4i** was obtained (1.17 g, 96%, yellowish oil).

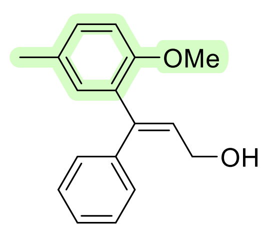

4i

^{1}H NMR (500 MHz, $CDCl_3$): δ 7.27 – 7.33 (m, 2H), 7.24 – 7.26 (m, 1H), 7.11 – 7.18 (m, 2H), 7.01 – 7.08 (m, 2H), 6.75 (d, *J* = 8.2 Hz, 1H), 6.03 (t, *J* = 6.8 Hz, 1H), 4.32 (d, *J* = 6.8 Hz, 2H), 3.54 (s, 3H), 2.25 – 2.32 (s, 3H), 1.54 (br, 1H). **^{13}C NMR (126 MHz, $CDCl_3$):** δ 155.2, 142.3, 140.1, 132.3, 131.7, 130.0, 129.9, 129.3, 129.0, 127.9, 127.1, 111.9, 60.6, 56.0, 20.6. **HRMS (ESI) m/z:** $[M\text{-}OH]^+$ Calcd for $C_{17}H_{17}O$ 237.1274; Found 237.1275. **IR υ_{max}:** 3319, 3052, 3019, 2920, 2855, 2832, 1889, 1824, 1668, 1602, 1492, 1459, 1440, 1405, 1287, 1259, 1237, 1179, 1153, 1127 cm^{-1}.

Preparation of (E)-3-(naphthalen-1-yl)-3-phenylprop-2-en-1-ol (4j)

Following **GP1, 1** (319.6 mg, 1.5 mmol, 1 equiv.), naphthalene-1-boronic acid (283.8 mg, 1.7 mmol, 1.1 equiv.), $Pd(OAc)_2$ (1.7 mg, 0.008 mmol, 0.5 mol%), PPh_3 (3.9 mg, 0.015 mmol, 1 mol%), and KOH (168.3 mg, 3.0 mmol, 2 equiv.) were used. The reaction was stirred overnight. After work-up and purification, **4j** was obtained (368.0 mg, 94%, yellow solid).

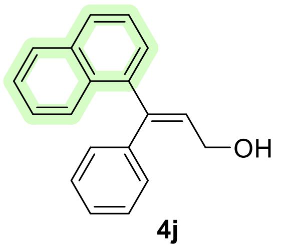

4j

^{1}H NMR (400 MHz, $CDCl_3$): δ 7.85 – 7.92 (m, 1H)**,** 7.81 (m, 2H), 7.45 (m, 1H), 7.38 – 7.43 (m, 2H), 7.28 – 7.37 (m, 2H), 7.18 – 7.28 (m, 4H), 6.07 (t, *J* = 6.8 Hz, 1H), 4.50 (d, *J* = 6.8 Hz, 2H), 1.62 (br, 1H). **^{13}C NMR (101 MHz, $CDCl_3$):** δ 143.3, 141.0, 139.9, 134.0, 131.8, 131.4, 129.1, 128.3, 128.1, 128.4, 127.7, 127.5, 126.2, 126.1, 125.7, 125.4, 60.6. **HRMS (ESI) m/z:** $[M\text{-}OH]^+$ Calcd for $C_{19}H_{15}$ 243.1168; Found 243.1176. **IR υ_{max}:** 3291, 3054, 3021, 2914, 2855, 1457, 1436, 1395, 1340, 1218, 1017 cm^{-1}. **Melting point:** 79 – 80 °C.

*Now the 2 different routes to synthesize 3 will be presented, they share the same characterization of products. Note that substrates **3a** and **3d** were only prepared via Route A.*

4.2. General procedure 2: Synthesis of 3,3-diarylallyl phthalimides (Route A).

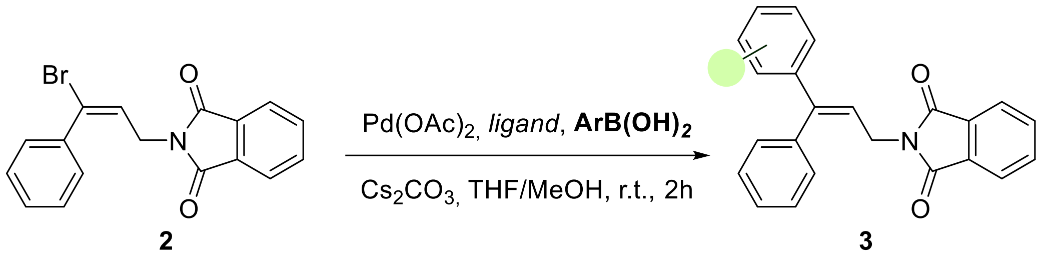

An anhydrous mixture of THF/MeOH (1:1) was deoxygenated for 30 min by bubbling N_2 into the solution. In parallel, an oven-dried vial was charged with $Pd(OAc)_2$ (2 mol%) and *ligand* (4 mol%), sealed and purged with vacuum-nitrogen cycles. Then, degassed THF/MeOH was added, and the resulting solution was heated in an oil bath at 90-100°C for 1-10 min (until colour change). This solution was cooled to room temperature and transferred via syringe to an oven-dried and N_2-purged flask containing **1** (1 equiv.), the corresponding aryl boronic acid (1.2 equiv.) and Cs_2CO_3 (2 equiv.). The reaction was stirred until completion (checked by ^{1}H NMR) at the set temperature. Upon completion, work-up was performed and purification of the crude by flash chromatography using Cy:EtOAc as eluent afforded the desired product **3**. In some examples recrystallization was needed after the purification to yield a successful hydrogenation.

4.3. General procedure 3: Synthesis of 3,3-diarylallyl phthalimides 3 (Route B).

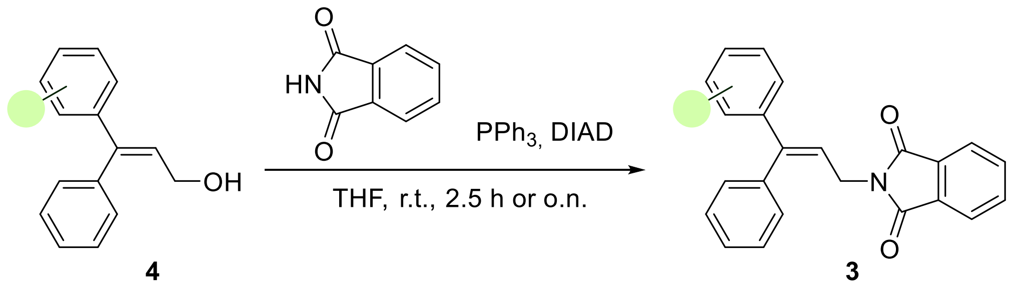

A round-bottom flask charged with **4** (1.0 equiv.), PPh_3 (1.3 equiv.) and phthalimide (1.3 equiv.), purged with vacuum-nitrogen cycles and dissolved in THF (0.3 M). Then, the reaction was cooled down to 0 °C and DIAD (1.3 equiv.) was added over 30 minutes with a syringe pump. After stirring the reaction for 2.5 h or overnight at room temperature, the solvent was evaporated, and the crude was purified by flash chromatography (Cy/EtOAc 9:1) to afford **3**. The non-desired *Z*-isomer traces were removed during the purification by flash chromatography or by a subsequent recrystallization.

Preparation of (E)-2-(3-(4-methoxyphenyl)-3-phenylallyl)isoindoline-1,3-dione (3a, Route A)

Similarly to **GP2**, $Pd(OAc)_2$ (24.0 mg, 0.11 mmol, 2 mol%) and PPh_3 (55.2 mg, 0.210 mmol, 4 mol%) were activated in degassed THF/MeOH (1:1, 15 mL), degassed H_2O (15 µL, 0.833 mmol, 16 mol%) was also added. The resulting solution was cooled to room temperature and transferred via syringe to an oven-dried and N_2-purged Schlenk flask containing **2** (1.81 g, 5.28 mmol, 1.0 equiv.) and 4-methoxyphenylboronic acid (961.2 mg, 6.33 mmol, 1.2 equiv.) solved in degassed THF/MeOH (1:1, 20 mL). Finally, KOH (85%, 604.4 mg, 10.50 mmol, 2.0 equiv.) instead of Cs_2CO_3 was added at 0 °C. After 2 h reaction time at room temperature the mixture was filtered through a pad of Celite, washed with DCM and concentrated under vacuum. Afterwards, EtOAc was added, and the mixture extracted with H_2O. The aqueous phase was then extracted 4 times with EtOAc, organic layers collected, dried over anhydrous $MgSO_4$, filtered, and concentrated under reduced pressure. Purification of the crude by flash chromatography (gradient from 0% to 30% of EtOAc in Cy) afforded pure **3a** (1.03 g, 67%, white solid). **3a** was recrystallized in hexanes/EtOAc prior to the hydrogenation.

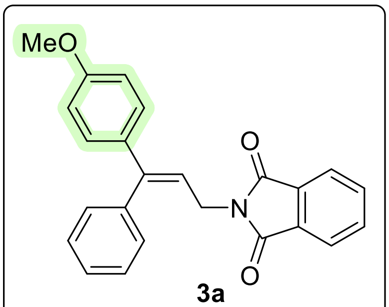

3a

^{1}H NMR (400 MHz, $CDCl_3$): δ 7.83 (m, 2H), 7.70 (m, 2H), 7.49 – 7.38 (m, 2H), 7.38 – 7.28 (m, 3H), 7.15 (d, J = 8.9 Hz, 2H), 6.78 (d, J = 8.9 Hz, 2H), 6.00 (t, J = 6.6 Hz, 1H), 4.37 (d, J = 6.6 Hz, 2H), 3.77 (s, 1H). **^{13}C NMR (101 MHz, $CDCl_3$):** δ 168.1, 159.4, 144.4, 139.1, 134.2, 134.0, 132.4, 129.9, 128.7, 128.5, 127.6, 123.3, 120.6, 113.6, 55.4, 37.8. **HRMS (ESI) m/z:** **[2M+Na]$^+$** Calcd for $C_{48}H_{38}N_2O_6Na$ 761.2622; Found 761.2626. **IR υ_{max}:** 3048, 3033, 2957, 2925, 2886, 1769, 1704, 1605, 1507 cm^{-1}. **Melting point:** 174 – 176 °C.

Preparation of (E)-2-(3-phenyl-3-(p-tolyl)allyl)isoindoline-1,3-dione (3b, Route A)

Following **GP2**, $Pd(OAc)_2$ (7.5 mg, 0.033 mmol, 2 mol%), *t*BuXPhos (52.5 mg, 0.12 mmol, 4 mol%), degassed THF/MeOH (1:1, 15 mL), **2** (598.8 mg, 1.75 mmol, 1.0 equiv.), *p*-tolylboronic acid (290.7 mg, 2.14 mmol, 1.2 equiv.), Cs_2CO_3 (1.14 g, 3.51 mmol, 2.0 equiv.) were used. After 2 h reaction time at room temperature the solvent was removed, EtOAc added, and the mixture extracted with H_2O. The aqueous phase was then extracted 4 times with EtOAc, organic layers collected, dried over anhydrous $MgSO_4$, filtered, and concentrated under reduced pressure. Purification of the crude

by flash chromatography (gradient from 0% to 10% of EtOAc in Cy) afforded pure **3b** (507.7 mg, 82%, white solid). **3b** was recrystallized in hexanes/EtOAc prior to the hydrogenation.

Preparation of (E)-2-(3-phenyl-3-(p-tolyl)allyl)isoindoline-1,3-dione (3b, Route B)

Following **GP3**, **4b** (270.0 mg, 1.2 mmol, 1.0 equiv.), PPh_3 (410.5 mg, 1.56 mmol, 1.3 equiv.), phthalimide (230.2 mg, 1.56 mmol, 1.3 equiv.), THF (4.0 mL) and DIAD (0.31 mL, 1.56 mmol, 1.3 equiv.) were used. The reaction was stirred for 2.5 h and after purification **3b** was obtained (270.3 mg, 64%, white solid). A recrystallization in hexanes/EtOAc was necessary to obtain **3b** as a single diastereomer.

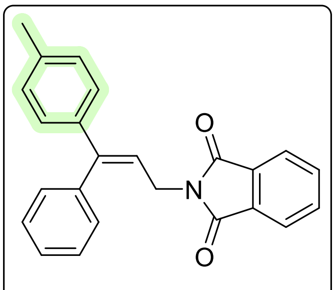

3b

^{1}H NMR (400 MHz, $CDCl_3$): δ 7.79 – 7.88 (m, 2H), 7.66 – 7.75 (m, 2H), 7.43 (t, *J* = 7.3 Hz, 2H), 7.28 – 7.38 (m, 3H), 7.11 (d, *J* = 8.0 Hz, 2H), 7.05 (d, *J* = 8.0 Hz, 2H), 6.05 (t, *J* = 6.6 Hz, 1H), 4.38 (d, *J* = 6.6 Hz, 2H), 2.31 (s, 3H). **^{13}C NMR (101 MHz, $CDCl_3$):** δ 168.1, 144.8, 139.1, 138.8, 137.6, 134.0, 132.4, 129.9, 129.0, 128.5, 127.6, 127.4, 123.3, 121.5, 37.8, 21.2. **HRMS (ESI) m/z:** $[2M+Na]^+$ Calcd for $C_{48}H_{38}N_2O_4Na$ 729.2724; Found 729.2724. **IR υ_{max}:** 3046, 2915, 2215, 1967, 1767, 1698, 1394 cm^{-1}. **Melting point:** 164 – 165 °C.

Preparation of (E)-2-(3-(4-chlorophenyl)-3-phenylallyl)isoindoline-1,3-dione (3c, Route A)

Similarly to **GP2**, $Pd(OAc)_2$ (2.6 mg, 0.012 mmol, 2 mol%), *t*BuXPhos (10.2 mg, 0.024 mmol, 4 mol%), degassed THF/MeOH (1:1, 5 mL), **2** (199.7 mg, 0.58 mmol, 1.0 equiv.), (4-chlorophenyl)boronic acid (109.4 mg, 0.70 mmol, 1.2 equiv.), Cs_2CO_3 (382.9 mg, 1.18 mmol, 2.0 equiv.) were used. After 2 h reaction time at 50 °C instead of room temperature, the mixture was filtered through a pad of Celite, washed with DCM and concentrated under vacuum. Afterwards, DCM was added, and the mixture extracted with H_2O. The aqueous phase was then extracted 4 times with DCM, organic layers collected, dried over anhydrous $MgSO_4$, filtered, and concentrated under reduced pressure. Purification of the crude by flash chromatography (gradient from 0% to 10% of EtOAc in Cy) afforded pure **3c** (157.7 mg, 72%, white solid).

Preparation of (E)-2-(3-(4-chlorophenyl)-3-phenylallyl)isoindoline-1,3-dione (3c, Route B)

Following **GP3**, **4c** (228.0 mg, 0.93 mmol, 1.0 equiv.), PPh_3 (318.5 mg, 1.21 mmol, 1.3 equiv.), phthalimide (178.7 mg, 1.21 mmol, 1.3 equiv.), THF (3.1 mL) and DIAD (0.24 mL, 1.21 mmol, 1.3 equiv.) were used. The reaction was stirred for 2.5 h and after purification **3c** was obtained (151.0 mg, 75%, white solid). A recrystallization in hexanes/EtOAc was necessary to obtain **3c** as a single diastereomer.

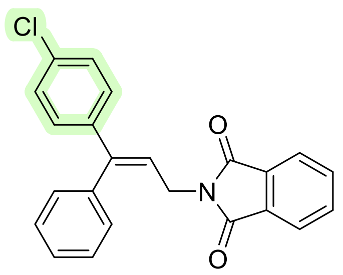

3c

^{1}H NMR (400 MHz, $CDCl_3$): δ 7.84 (m, 2H), 7.71 (m, 2H), 7.44 (t, *J* = 7.7 Hz, 2H), 7.34 – 7.39 (m, 1H), 7.29 – 7.34 (m, 2H), 7.21 (d, *J* = 8.7 Hz, 2H), 7.14 (d, *J* = 8.7 Hz, 2H), 6.06 (t, *J* = 6.6 Hz, 1H), 4.39 (d, *J* = 6.6 Hz, 2H). **^{13}C NMR (101 MHz, $CDCl_3$):** δ 168.1, 143.9, 140.1, 138.4, 134.1, 133.6, 132.3, 129.8, 128.8, 128.7, 128.4, 127.9, 123.4, 122.9, 37.7. **HRMS (ESI) m/z:** $[M+Na]^+$ Calcd for $C_{23}H_{16}ClNO_2Na$ 396.0762; Found 396.0758. **IR υ_{max}:** 3044, 2956, 2916, 2849, 2354, 1976, 1768, 1702, 1394 cm^{-1}. **Melting point:** 131 – 134 °C.

Preparation of (E)-2-(3-(4-fluorophenyl)-3-phenylallyl)isoindoline-1,3-dione (3d, Route A)

Following **GP2**, $Pd(OAc)_2$ (3.0 mg, 0.013 mmol, 2 mol%), *t*BuXPhos (10.2 mg, 0.024 mmol, 4 mol%), degassed THF/MeOH (1:1, 5 mL), **2** (201.2 mg, 0.59 mmol, 1.0 equiv.), 4-fluorophenylboronic acid (98.6 mg, 0.70 mmol, 1.2 equiv.), Cs_2CO_3 (391.9 mg, 1.20 mmol, 2.0 equiv.) were used. After 2 h reaction time at room temperature the solvent was removed, DCM added, and the mixture extracted with H_2O. The aqueous phase was then extracted 3 times with DCM, organic layers collected, dried over anhydrous $MgSO_4$, filtered, and concentrated under reduced pressure. Purification of the crude by flash chromatography (gradient from 0% to 10% of EtOAc in Cy) afforded pure **3d** (193.3 mg, 82%, white solid).

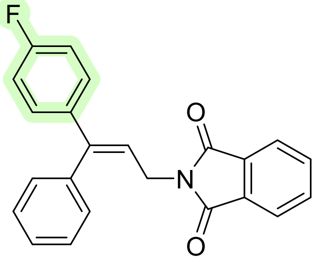

3d

^{1}H NMR (400 MHz, $CDCl_3$): δ 7.83 (m, 2H), 7.71 (m, 2H), 7.41 – 7.46 (m, 2H), 7.33 – 7.39 (m, 3H), 7.29 – 7.33 (m, 2H), 7.15 – 7.22 (m, 2H), 6.89 – 6.96 (m, 2H), 6.01 (t, *J* = 6.6 Hz, 1H), 4.38 (d, *J* = 6.6 Hz, 2H). **^{13}C NMR (101 MHz, $CDCl_3$):** δ 168.1, 162.5 (d, *J* = 247.2 Hz), 143.9, 138.7, 137.8 (d, *J* = 3.3 Hz), 134.1, 132.3, 129.8, 129.1 (d, *J* = 8.1 Hz), 128.6, 127.9, 123.4, 122.3, 115.1 (d, *J* = 21.3

Hz), 37.7. **HRMS (ESI) m/z:** [M+Na]$^+$ Calcd for $C_{23}H_{16}FNO_2Na$ 380.1057; Found 380.1061. **IR υ_{max}:** 3059, 3029, 2925, 2256, 1977, 1770, 1704, 1393 cm^{-1}. **Melting point:** 117 – 118 °C.

Preparation of (E)-2-(3-(3-fluorophenyl)-3-phenylallyl)isoindoline-1,3-dione (3e, Route A)

Following **GP2**, $Pd(OAc)_2$ (3.0 mg, 0.013 mmol, 2 mol%), *t*BuXPhos (10.2 mg, 0.024 mmol, 4 mol%), degassed THF/MeOH (1:1, 5 mL), **2** (202.2 mg, 0.59 mmol, 1.0 equiv.), 3-fluorophenylboronic acid (101.1 mg, 0.72 mmol, 1.2 equiv.), Cs_2CO_3 (390.9 mg, 1.20 mmol, 2.0 equiv.) were used. The reaction mixture was set at 50 °C and after 2 h, more 3-fluorophenylboronic acid (25.0 mg, 0.18 mmol, 0.3 equiv.) was added. After 2 h more the solvent was removed, DCM added, and the mixture extracted with H_2O. The aqueous phase was then extracted 4 times with DCM, organic layers collected, dried over anhydrous $MgSO_4$, filtered, and concentrated under reduced pressure. Purification of the crude by flash chromatography (gradient from 0% to 10% of EtOAc in Cy) afforded pure **3e** (201.6 mg, 95%, white solid). **3e** was recrystallized in hexanes/EtOAc prior to the hydrogenation.

Preparation of (E)-2-(3-(3-fluorophenyl)-3-phenylallyl)isoindoline-1,3-dione (3e, Route B)

Following **GP3**, **4e** (121.5 mg, 0.53 mmol, 1.0 equiv.), PPh_3 (181.5 mg, 0.69 mmol, 1.3 equiv.), phthalimide (101.8 mg, 0.69 mmol, 1.3 equiv.), THF (1.8 mL) and DIAD (0.14 mL, 0.69 mmol, 1.3 equiv.) were used. The reaction was stirred for 2.5 h and after purification **3e** was obtained (115.4 mg, 65%, white solid). A recrystallization in hexanes/EtOAc was necessary to obtain **3e** as a single diastereomer.

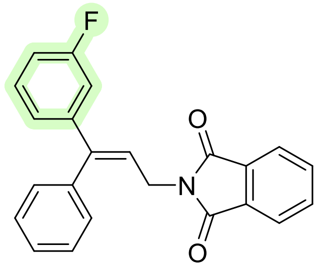

3e

^{1}H NMR (400 MHz, $CDCl_3$): δ 7.84 (m, 2H), 7.71 (m, 2H), 7.42 – 7.47 (m, 2H), 7.32 – 7.41 (m, 1H), 7.30 – 7.34 (m, 2H), 7.15 – 7.25 (m, 1H), 6.98 – 7.03 (m, 1H), 6.87 – 6.97 (m, 2H), 6.09 (t, *J* = 6.6 Hz, 1H), 4.38 (d, *J* = 6.6 Hz, 2H). **^{13}C NMR (101 MHz, $CDCl_3$):** δ 168.1, 162.9 (d, *J* = 245.2 Hz), 143.9 (d, *J* = 2.2 Hz), 143.9 (d, *J* = 2.8 Hz), 138.3, 134.1, 132.3, 129.8, 129.7 (d, *J* = 8.4 Hz), 128.7, 128.0, 123.5, 123.4, 123.1 (d, *J* = 2.9 Hz), 114.6 (d, *J* = 14.7 Hz), 114.4 (d, *J* = 15.6 Hz), 37.7. **HRMS (ESI) m/z:** [M+Na]$^+$ Calcd for $C_{23}H_{16}FNO_2Na$ 380.1057; Found 380.1067 **IR υ_{max}:** 3027, 2921, 2206, 1946, 1769, 1699, 1392 cm^{-1}. **Melting point:** 148 – 149 °C.

Preparation of (E)-2-(3-(2-methoxyphenyl)-3-phenylallyl)isoindoline-1,3-dione (3f, Route A)

Following **GP2**, $Pd(OAc)_2$ (3.3 mg, 0.015 mmol, 2 mol%), PPh_3 (7.2 mg, 0.027 mmol, 4 mol%), degassed THF/MeOH (1:1, 5 mL), **2** (201.0 mg, 0.89 mmol, 1.0 equiv.), 2-methoxyphenylboronic acid (107.0 mg, 0.70 mmol, 1.2 equiv.), Cs_2CO_3 (386.0 mg, 1.18 mmol, 2.0 equiv.) were used. After 2 h reaction time at room temperature solvent was removed, DCM added, and the mixture extracted with brine. The aqueous phase was then extracted 3 times with DCM, organic layers collected, dried over anhydrous $MgSO_4$, filtered, and concentrated under reduced pressure. Purification of the crude by flash chromatography (gradient from 0% to 10% of EtOAc in Cy) afforded pure **3f** (178.5 mg, 82%, white solid).

Preparation of (E)-2-(3-(2-methoxyphenyl)-3-phenylallyl)isoindoline-1,3-dione (3f, Route B)

Following **GP3**, **4f** (1.12 g, 4.66 mmol, 1.0 equiv.), PPh_3 (1.59 g, 6.06 mmol, 1.3 equiv.), phthalimide (891.5 mg, 6.06 mmol, 1.3 equiv.), THF (15.0 mL) and DIAD (1.19 mL, 6.06 mmol, 1.3 equiv.) were used. The reaction was stirred for 2.5 h and after purification **3f** was obtained (1.30 g, 76%, white solid). A recrystallization in hexanes/EtOAc was necessary to obtain **3f** as a single diastereomer.

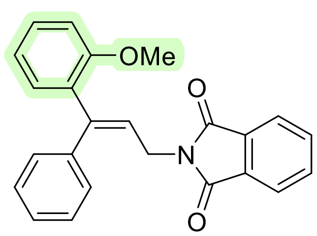

3f

^{1}H NMR (500 MHz, CDCl$_3$): δ 7.75 (m, 2H), 7.61 (m, 2H), 7.27 (d, *J* = 2.6 Hz, 3H), 7.09 – 7.23 (m, 4H), 6.78 – 6.86 (t, *J* = 7.5 Hz, 1H), 6.72 (d, *J* = 8.3 Hz, 1H), 5.80 (t, *J* = 6.6 Hz, 1H), 4.41 (d, *J* = 6.6 Hz, 2H), 3.48 (s, 3H). **^{13}C NMR (126 MHz, CDCl$_3$):** δ 168.1, 157.2, 142.9, 139.9, 134.0, 132.4, 131.0, 129.1, 129.0, 128.0, 127.1, 124.9, 123.3, 120.6, 111.7, 55.7, 37.5. **HRMS (ESI) m/z:** $[2M+Na]^+$ Calcd for $C_{48}H_{38}N_2O_6Na$ 761.2622; Found 761.2615. **IR υ_{max}:** 2957, 2918, 2833, 2212, 1991, 1763, 1709, 1388 cm^{-1}. **Melting point:** 138 – 139 °C.

Preparation of (E)-2-(3-(2-chlorophenyl)-3-phenylallyl)isoindoline-1,3-dione (3g, Route A)

Following **GP2**, $Pd(OAc)_2$ (7.9 mg, 0.035 mmol, 2 mol%), PPh_3 (19.2 mg, 0.045 mmol, 4 mol%), degassed THF/MeOH (1:1, 15 mL), **2** (600.6 mg, 1.76 mmol, 1.0 equiv.), 2-chlorophenylboronic acid (328.4 mg, 2.10 mmol, 1.2 equiv.), Cs_2CO_3 (1.14 g, 3.48 mmol, 2.0 equiv.) were used. After 2 h reaction time at room temperature the solvent was removed, EtOAc added, and the mixture extracted with H_2O. The aqueous phase was then extracted 3 times with EtOAc, organic

layers collected, dried over anhydrous $MgSO_4$, filtered, and concentrated under reduced pressure. Purification of the crude by flash chromatography (gradient from 0% to 10% of EtOAc in Cy) afforded pure **3g** (386.4 mg, 59%, white solid). **3g** was recrystallized in heptane/TBME prior to the hydrogenation.

Preparation of (E)-2-(3-(2-chlorophenyl)-3-phenylallyl)isoindoline-1,3-dione (3g, Route B)

Following **GP3**, **4g** (150.0 mg, 0.61 mmol, 1.0 equiv.), PPh_3 (209.0 mg, 0.80 mmol, 1.3 equiv.), phthalimide (117.2 mg, 0.80 mmol, 1.3 equiv.), THF (2.0 mL) and DIAD (0.16 mL, 0.80 mmol, 1.3 equiv.) were used. The reaction was stirred overnight and after purification **3g** was obtained as a single diastereomer (121.3 mg, 53%, white solid).

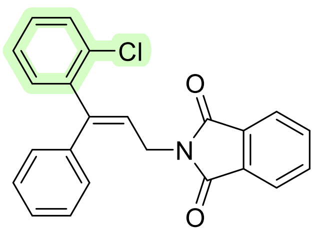

3g

^{1}H NMR (400 MHz, $CDCl_3$): δ 7.84 (m, 2H), 7.71 (m, 2H), 7.30 – 7.41 (m, 4H), 7.25 – 7.34 (m, 3H), 7.14 – 7.25 (m, 2H), 5.77 (t, *J* = 6.6 Hz, 1H), 4.57 (d, *J* = 6.6 Hz, 2H). **^{13}C NMR (101 MHz, $CDCl_3$):** δ 168.1, 143.1, 141.7, 138.4, 134.1, 133.3, 132.3, 131.6, 130.0, 129.5, 128.9, 128.3, 127.7, 126.6, 126.5, 123.4, 37.3. **HRMS (ESI) m/z:** $[M+Na]^+$ Calcd for $C_{23}H_{16}ClNO_2Na$ 396.0762 / found 396.0760. **IR υ_{max}:** 3054, 3025, 2930, 2394, 2297, 1961, 1774, 1710, 1426, 1394 cm^{-1}. **Melting point:** 117 – 118 °C.

Preparation of (E)-2-(3-(2-chloro-4-methoxyphenyl)-3-phenylallyl)isoindoline-1,3-dione (3h, Route A)

Similarly to **GP2**, $Pd(OAc)_2$ (3.3 mg, 0.015 mmol, 2 mol%) and PPh_3 (8.3 mg, 0.020 mmol, 4 mol%) were activated in degassed THF/MeOH (1:1, 5 mL), degassed H_2O (2 µL, 0.11 mmol, 16 mol%) was also added. The resulting solution was cooled to room temperature and transferred via syringe to an oven-dried and N_2-purged Schlenk flask containing **2** (250.9 mg, 0.73 mmol, 1.0 equiv.) and 2-chloro-4-methoxyphenylboronic acid (163.4 mg, 0.88 mmol, 1.2 equiv.). Finally, KOH (85%, 98.2 mg, 1.50 mmol, 2.0 equiv.) instead of Cs_2CO_3 was added at 0 °C. After 2 h reaction time at room temperature the mixture was filtered through a pad of Celite, washed with DCM and concentrated under vacuum. Afterwards, DCM was added, and the resulting mixture extracted with NaOH (1 M). The aqueous phase was then extracted 3 times with DCM, organic layers collected, dried over anhydrous $MgSO_4$, filtered, and concentrated under reduced pressure. Purification of the crude by

flash chromatography (gradient from 0% to 10% of EtOAc in Cy) afforded pure **3h** (224.1 mg, 76%, white oil).

Preparation of (E)-2-(3-(2-chloro-4-methoxyphenyl)-3-phenylallyl)isoindoline-1,3-dione (3h, Route B)

Following **GP3**, **4h** (516.0 mg, 1.88 mmol, 1.0 equiv.), PPh_3 (640.4 mg, 2.44 mmol, 1.3 equiv.), phthalimide (359.2 mg, 2.44 mmol, 1.3 equiv.), THF (6.3 mL) and DIAD (0.48 mL, 2.44 mmol, 1.3 equiv.) were used. The reaction was stirred for 2.5 h and after purification **3h** was obtained as a single diastereomer (633.9 mg, 84%, white solid).

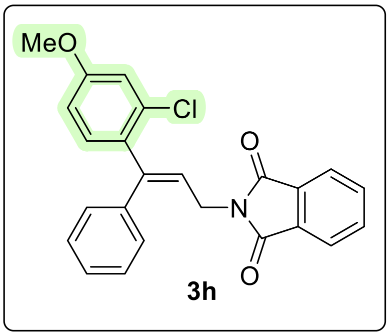

^{1}H NMR (500 MHz, $CDCl_3$): δ 7.83 (m, 2H), 7.70 (m, 2H), 7.31 – 7.41 (m, 3H), 7.22 – 7.35 (m, 2H), 7.19 (d, *J* = 8.5 Hz, 1H), 6.86 (d, *J* = 2.6 Hz, 1H), 6.75 (dd, *J* = 8.5, 2.6 Hz, 1H), 5.74 (t, *J* = 6.6 Hz, 1H), 4.55 (d, *J* = 6.6 Hz, 2H), 3.77 (s, 3H). **^{13}C NMR (126 MHz, $CDCl_3$):** δ 168.1, 160.0, 142.7, 138.8, 134.2, 134.1, 133.9, 132.3, 132.2, 129.4, 128.3, 127.6, 126.2, 123.4, 115.2, 112.2, 55.6, 37.4. **HRMS (ESI) m/z:** $[2M+Na]^+$ Calcd for $C_{48}H_{36}Cl_2N_2O_6Na$ 829.1843; Found 829.1836. **IR υ_{max}:** 3467, 3079, 3055, 3025, 2925, 2837, 2354, 1941, 1770, 1707, 1492, 1387 cm^{-1}. **Melting point:** 56 – 64 °C.

Preparation of (E)-2-(3-(2-methoxy-5-methylphenyl)-3-phenylallyl)isoindoline-1,3-dione (3i, Route A)

Similarly to **GP2**, $Pd(OAc)_2$ (2.6 mg, 0.012 mmol, 2 mol%) and PPh_3 (6.5 mg, 0.023 mmol, 4 mol%) were activated in degassed THF/MeOH (1:1, 5 mL), degassed H_2O (2 µL, 0.09 mmol, 16 mol%) was also added. The resulting solution was cooled to room temperature and transferred via syringe to an oven-dried and N_2-purged Schlenk flask containing **2** (1806.5 mg, 5.28 mmol, 1.0 equiv.) and 2-methoxy-5-methylphenylboronic acid (120.0 mg, 0.70 mmol, 1.2 equiv.) solved in degassed THF/MeOH (1:1, 20 mL). Finally, Cs_2CO_3 (400.9 mg, 1.17 mmol, 2.0 equiv.) was added. After 2 h reaction time at room temperature the mixture was filtered through a pad of Celite, washed with DCM and concentrated under vacuum. Afterwards, DCM was added, and the resulting mixture extracted with NaOH (1 M). The aqueous phase was then extracted 3 times with DCM, organic layers collected, dried over anhydrous $MgSO_4$, filtered, and concentrated under reduced pressure. Purification of the

crude by flash chromatography (gradient from 0% to 5% of EtOAc in hexanes) afforded pure **3i** (167.6 mg, 75%, white solid).

Preparation of (E)-2-(3-(2-methoxy-5-methylphenyl)-3-phenylallyl)isoindoline-1,3-dione (3i, Route B)

Following **GP3**, **4i** (1.13 g, 4.45 mmol, 1.0 equiv.), PPh_3 (1.59 g, 5.79 mmol, 1.3 equiv.), phthalimide (851.8 mg, 5.79 mmol, 1.3 equiv.), THF (15.0 mL) and DIAD (1.14 mL, 5.79 mmol, 1.3 equiv.) were used. The reaction was stirred for 2.5 h and after purification **3i** was obtained (1.07 g, 63%, white solid). A recrystallization in hexanes/EtOAc was necessary to obtain **3i** as a single diastereomer.

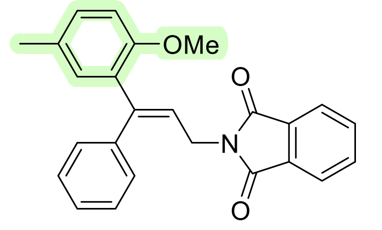

3i

^{1}H NMR (500 MHz, $CDCl_3$): δ 7.80 – 7.87 (m, 2H), 7.65 – 7.76 (m, 2H), 7.35 (d, *J* = 4.1 Hz, 4H), 7.22 – 7.31 (m, 1H), 7.00 – 7.06 (m, 2H), 6.71 (d, *J* = 8.0 Hz, 1H), 5.85 (t, *J* = 6.6 Hz, 1H), 4.49 (d, *J* = 6.6 Hz, 2H), 3.52 (s, 3H), 2.26 (s, 3H). **^{13}C NMR (126 MHz, $CDCl_3$):** δ 168.1, 155.2, 143.1, 139.9, 134.0, 132.4, 132.2, 131.5, 129.8, 129.3, 129.1, 128.0, 127.0, 124.7, 123.3, 112.0, 56.0, 37.5, 20.5. **HRMS (ESI) m/z:** $[2M+Na]^+$ Calcd for $C_{50}H_{42}N_2O_6Na$ 789.2935; Found 789.2934. **IR υ_{max}:** 3018, 2996, 2932, 2629, 2198, 1917, 1789, 1705, 1392 cm^{-1} **Melting point:** 123-124 °C.

Preparation of (E)-2-(3-(naphthalen-1-yl)-3-phenylallyl)isoindoline-1,3-dione (3j, Route A)

Similarly to **GP2**, $Pd(OAc)_2$ (2.6 mg, 0.012 mmol, 2 mol%) and PPh_3 (6.5 mg, 0.023 mmol, 4 mol%) were activated in degassed THF/MeOH (1:1, 5 mL), degassed H_2O (2 µL, 0.09 mmol, 16 mol%) was also added. The resulting solution was cooled to room temperature and transferred via syringe to an oven-dried and N_2-purged Schlenk flask containing **2** (1.81 g, 5.28 mmol, 1.0 equiv.) and naphthalene-1-boronic acid (123.1 mg, 0.70 mmol, 1.2 equiv.) solved in degassed THF/MeOH (1:1, 20 mL). Finally, Cs_2CO_3 (400.9 mg, 1.17 mmol, 2.0 equiv.) was added. After 2 h reaction time at room temperature the mixture was filtered through a pad of Celite, washed with DCM and concentrated under vacuum. Afterwards, DCM was added, and the resulting mixture extracted with NaOH (1 M). The aqueous phase was then extracted 3 times with DCM, organic layers collected, dried over anhydrous $MgSO_4$, filtered, and concentrated under reduced pressure. Purification of the crude by

flash chromatography (gradient from 0% to 5% of EtOAc in hexanes) afforded pure **3j** (210.8 mg, 93%, white solid).

Preparation of (E)-2-(3-(naphthalen-1-yl)-3-phenylallyl)isoindoline-1,3-dione (3j, Route B)

Following **GP3, 4j** (336.0 mg, 1.29 mmol, 1.0 equiv.), PPh_3 (440.1 g, 1.68 mmol, 1.3 equiv.), phthalimide (247.0 mg, 1.68 mmol, 1.3 equiv.), THF (4.3 mL) and DIAD (0.33 mL, 1.68 mmol, 1.3 equiv.) were used. The reaction was stirred for 2.5 h and after purification **3j** was obtained (470.0 mg, 94%, white solid). A recrystallization in hexanes/EtOAc was necessary to obtain **3j** as a single diastereomer.

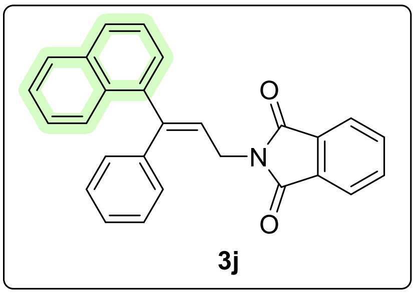

^{1}H NMR (500 MHz, $CDCl_3$): δ 7.98 – 8.00 (m, 1H), 7.82 – 7.86 (m, 2H), 7.76 – 7.80 (m, 2H), 7.67 – 7.72 (m, 2H), 7.32 – 7.49 (m, 8H), 7.27 (m, 1H), 5.85 (t, *J* = 6.4 Hz, 1H), 4.71 (d, *J* = 6.4 Hz, 2H). **^{13}C NMR (126 MHz, $CDCl_3$):** δ 168.2, 143.8, 140.9, 139.7, 134.1, 133.9, 132.3, 131.8, 129.2, 128.5, 128.3, 128.0, 127.7, 127.3, 126.4, 126.2, 126.19, 125.7, 125.3, 123.4, 37.6. **HRMS (ESI) m/z:** $[2M+Na]^+$ Calcd for $C_{54}H_{38}N_2O_4Na$ 801.2724; Found 801.2715. **IR υ_{max}:** 3052, 3030, 2923, 2211, 1914, 1769, 1704, 1392 cm^{-1}. **Melting point:** 118-120 °C. Spectroscopic analysis matches with previously reported data.[3]

4.4. General procedure 4: Asymmetric hydrogenation of 3,3-diarylallyl phthalimides 3.

Here is described the general procedure for the asymmetric hydrogenation. This procedure is used in both, Route A and Route B. However, the one described, and the results/chromatograms reported correspond to Route B which offers better selectivity. The selectivities obtained via Route A can be found on the main article. Substrates ***5a*** *and* ***5d*** *were only synthesized via Route A.*

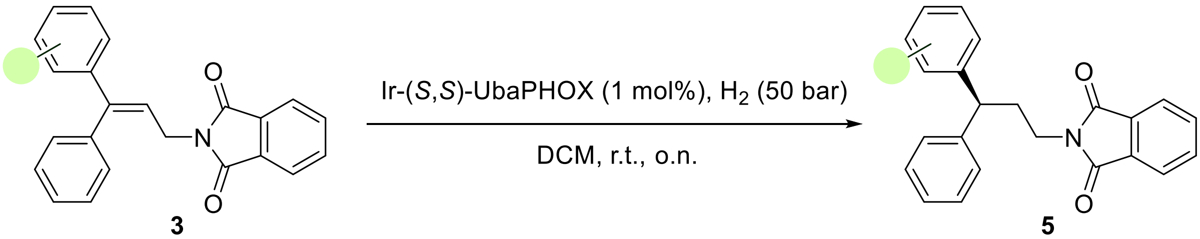

The corresponding substrate **3** (1.0 equiv.), Ir-(*S*,*S*)-UbaPHOX (1 mol%) and a PTFE-coated stir-bar were placed in a glass tube. Anhydrous DCM was added, and the glass tube was placed inside a stainless-steel high-pressure reactor. The reactor was closed and connected to a hydrogen manifold. The connection was purged with vacuum-nitrogen cycles, the valve of the reactor opened, evacuated, and finally charged at 50 bar of hydrogen pressure. The valve was closed, the hydrogen manifold unplugged, and the mixture was left to stir at room temperature overnight. The reactor was then depressurized, the solvent evaporated, and the conversion of the reaction was determined by ^{1}H-NMR. Purification of the crude by flash chromatography (gradient from 0% to 10% - 20% of EtOAc in Cy or hexanes) afforded the desired product **5**. The enantiomeric excess was determined by chiral HPLC chromatography.

Racemates: *To find suitable chiral HPLC separation conditions, racemates of compounds **3a** and **3c** were prepared by treating the corresponding allyl amine **2** with Pd/C moistened with water (10 %, 15 mol%) in EtOH at balloon pressure of H_2 for 24 h (48 h for **3c**). Afterwards, reaction crudes were filtered through a small plug of celite to afford analytically pure compounds. Racemates **3f** and **3i** were obtained setting the pressure at 3 bar of H_2 instead of a balloon. Racemate **3d** was obtained using 15 bar of H_2. To obtain racemates **3j** and **3e** the pressure was raised to 50 bar of H_2. Racemate **3b** was also set at 50 bar but DCM was used as solvent instead of EtOH. To obtain racemates **3h** and **3g**, the allyl amine **2** was treated in DCM with less Pd/C moistened with water (10%, 10 mol%) and with 0.1 equivalents of methanesulfonic acid to avoid dechlorination. Racemate **3g** required 4 bar of H_2 pressure and 48 h of reaction time. **3h** required instead 3 bar and 24 h. Analytical data matched with the chiral substrates.*

Preparation of (S)-2-(3-(4-methoxyphenyl)-3-phenylpropyl)isoindoline-1,3-dione (5a)

Following **GP4**, Ir-(*S*,*S*)-UbaPHOX (26.1 mg, 0.015 mmol, 1 mol%), **3a** (553.6 mg, 1.50 mmol) and DCM (5.0 mL, 0.30 M) were used. Purification of the crude by flash chromatography (gradient from 0% to 20% of EtOAc in Cy) afforded pure **5a** (549.2 mg, 99%, 99% ee, white solid).

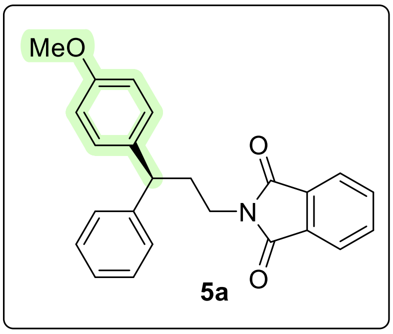

^{1}H NMR (400 MHz, $CDCl_3$): δ 7.77 (m, 2H), 7.67 (m, 2H), 7.21 – 7.28 (m, 4H), 7.18 (d, *J* = 8.6 Hz, 2H), 7.06 – 7.15 (m, 1H), 6.78 (d, *J* = 8.4 Hz, 2H), 3.97 (t, *J* = 7.8 Hz, 1H), 3.66 – 3.71 (m, 2H), 3.72 (s, 3H), 2.44 (q, *J* = 8.4, 7.8 Hz, 2H). **^{13}C NMR (101 MHz, $CDCl_3$):** δ 168.4, 158.1, 144.6, 136.2, 133.9, 132.2, 128.7, 128.7, 127.7, 126.3, 123.2, 114.0, 55.3, 48.6, 37.3, 34.1. **HRMS (ESI) m/z:** $[M+Na]^+$ Calcd for $C_{24}H_{21}NO_3Na$ 394.1419; Found 394.1414. **IR υ_{max}:** 3057, 3026, 2958, 2940, 1769, 1703, 1610, 1509 cm^{-1}. **Melting point:** 108 – 109 °C. **HPLC:** Chiralpak IC, *n*-heptane:IPA (95:5), 1 mL/min, λ = 220 nm, t_{R1} = 16.9 min, t_{R2} = 19.1 min. **[α]$_D$:** + 4.4 (c 1.11, $CHCl_3$).

Preparation of (S)-2-(3-phenyl-3-(p-tolyl)propyl)isoindoline-1,3-dione (5b)

Following **GP4**, Ir-(*S,S*)-UbaPHOX (17.1 mg, 0.0099 mmol, 1 mol%), **3b** (354.2 mg, 1.002 mmol) and DCM (4.20 mL, 0.24 M) were used. Purification of the crude by flash chromatography (gradient from 0% to 10% of EtOAc in Cy) afforded pure **5b** (354.4 mg, 99%, 98% ee, white solid).

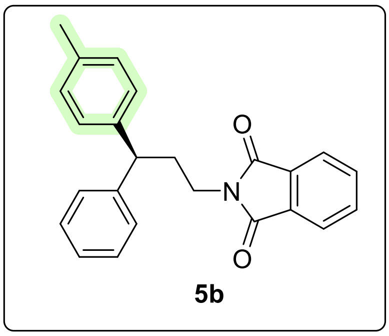

^{1}H NMR (500 MHz, $CDCl_3$): δ 7.69 (m, 2H), 7.59 (m, 2H), 7.12 – 7.20 (m, 4H), 7.08 (d, *J* = 8.0 Hz, 2H), 7.00 – 7.05 (m, 1H), 6.96 (d, *J* = 8.0 Hz, 2H), 3.90 (t, *J* = 7.7 Hz, 1H), 3.60 – 3.68 (m, 2H), 2.31 – 2.45 (m, 2H), 2.15 (s, 3H). **^{13}C NMR (126 MHz, $CDCl_3$):** δ 168.4, 144.4, 141.1, 136.0, 133.9, 132.3, 129.4, 128.7, 127.7, 127.7, 126.3, 123.2, 49.1, 37.3, 34.0, 21.1. **HRMS (ESI) m/z:** $[M+Na]^+$ Calcd for $C_{24}H_{21}NO_2Na$ 378.1469; Found 378.1469. **IR υ_{max}:** 3012, 2919, 2859, 2175-1973, 1768, 1703, 1396 cm^{-1}. **Melting point:** 89 – 91 °C. **HPLC:** Chiralpak IA, *n*-heptane:IPA (97:3), 0.5 mL/min, λ = 210 nm, t_{R1} = 24.9 min, t_{R2} = 26.4 min. **[α]$_D$:** + 5.3 (c 1.03, $CHCl_3$).

Preparation of (S)-2-(3-(4-chlorophenyl)-3-phenylpropyl)isoindoline-1,3-dione (5c)

Following **GP4**, Ir-(*S,S*)-UbaPHOX (2.7 mg, 0.0016 mmol, 1 mol%), **3c** (55.6 mg, 0.15 mmol) and DCM (0.74 mL, 0.20 M) were used. Purification of the crude by flash chromatography (gradient from 0% to 10% of EtOAc in Cy) afforded pure **5c** (55.5 mg, 99%, 99% ee, white oil).

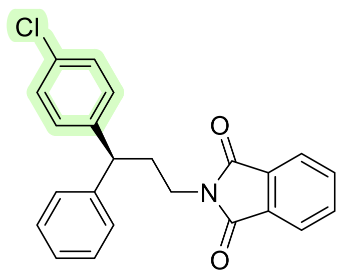

5c

^{1}H NMR (400 MHz, $CDCl_3$): δ 7.70 (m, 2H), 7.60 (m, 2H), 7.12 – 7.22 (m, 4H), 7.12 (m, 4H), 7.02 – 7.09 (m, 1H), 3.91 (t, *J* = 7.7 Hz, 1H), 3.57 – 3.67 (m, 2H), 2.30 – 2.42 (m, 2H). **^{13}C NMR (101 MHz, $CDCl_3$):** δ 168.3, 143.5, 142.5, 133.9, 132.1, 132.0, 129.0, 128.7, 127.5, 126.6, 123.1, 48.7, 37.0, 33.7. **HRMS (ESI) m/z:** $[M+H]^+$ Calcd for $C_{23}H_{19}ClNO_2$: 376.1099; Found 376.1100. **IR υ_{max}:** 3462, 3025, 2935, 2235-1959, 1770, 1703, 1614, 1489, 1466, 1451, 1436, 1393, 1366, 1243, 1187 cm^{-1}. **HPLC:** Chiralpak IA, *n*-heptane:IPA (92:8), 1 mL/min, λ = 210 nm, t_{R1} = 21.9 min, t_{R2} = 23.0 min. **$[\alpha]_D$:** + 1.1 (c 1.03, $CHCl_3$).

Preparation of (S)-2-(3-(4-fluorophenyl)-3-phenylpropyl)isoindoline-1,3-dione (5d)

Following **GP4**, Ir-(*S,S*)-UbaPHOX (3.0 mg, 0.0017 mmol, 1 mol%), **3d** (60.0 mg, 0.168 mmol) and DCM (0.84 mL, 0.2 M) were used. Purification of the crude by flash chromatography (gradient from 0% to 20% of EtOAc in hexanes) afforded pure **5d** (48.8 mg, 81%, 99% ee, yellow solid).

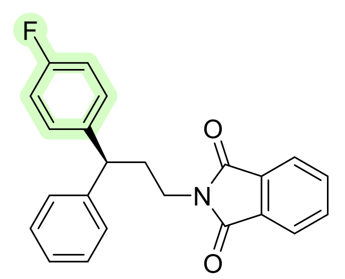

5d

^{1}H NMR (400 MHz, $CDCl_3$): δ 7.65 – 7.75 (m, 2H), 7.55 – 7.64 (m, 2H), 7.15 – 7.20 (m, 2H), 7.11 – 7.17 (m, 1H), 7.02 – 7.07 (m, 1H), 6.79 – 6.90 (m, 2H), 3.93 (t, J = 7.7 Hz, 1H), 3.58 – 3.67 (m, 2H), 2.28 – 2.43 (m, 2H). **^{13}C NMR (101 MHz, $CDCl_3$):** δ 168.4, 161.5 (d, *J* = 244.6 Hz), 143.9, 139.8 (d, *J* = 3.2 Hz), 134.0, 132.2, 129.22 (d, *J* = 7.9 Hz), 128.8, 127.7, 126.6, 123.2, 115.45 (d, *J* = 21.2 Hz), 48.7, 37.1, 34.1. **HRMS (ESI) m/z:** $[M+Na]^+$ Calcd for $C_{23}H_{18}FNO_2Na$ 382.1214; Found 382.1218. **IR υ_{max}:** 3028, 2945, 2253-1916, 1769, 1698, 1397 cm^{-1}. **Melting point:** 77-78 °C **HPLC:** Chiralpak IA, *n*-heptane:EtOH (9:1), 0.5 mL/min, λ = 220 nm, t_{R1} = 24.4 min, t_{R2} = 29.6 min. **$[\alpha]_D$**: -1.88 (c 1.07, $CHCl_3$).

Preparation of (S)-2-(3-(3-fluorophenyl)-3-phenylpropyl)isoindoline-1,3-dione (5e)

Following **GP4**, Ir-(*S,S*)-UbaPHOX (3.1 mg, 0.0018 mmol, 1 mol%), **3e** (62.2 mg, 0.17 mmol) and DCM (0.84 mL, 0.21 M) were used. Purification of the crude by flash chromatography (gradient from 0% to 10% of EtOAc in Cy) afforded pure **5e** (62.1 mg, 99%, 98% ee, white solid).

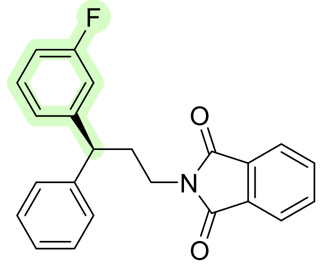

5e

^{1}H NMR (500 MHz, $CDCl_3$): δ 7.70 (m, 2H), 7.60 (m, 2H), 7.18 (m, 4H), 7.08 – 7.14 (m, 1H), 7.02 – 7.08 (m, 1H), 6.98 (d, *J* = 7.7 Hz, 1H), 6.88 (dt, *J* = 10.2, 2.1 Hz, 1H), 6.72 (tdd, *J* = 8.4, 2.6, 1.0 Hz, 1H), 3.93 (t, *J* = 7.7 Hz, 1H), 3.63 (d, *J* = 7.3 Hz, 2H), 2.31 – 2.45 (m, 2H). **^{13}C NMR (126 MHz, $CDCl_3$):** δ 168.4, 163.0 (d, *J* = 245.9 Hz), 146.8 (d, *J* = 6.8 Hz), 143.3, 134.0, 132.2, 130.1 (d, *J* = 8.3 Hz), 128.8, 127.7, 126.7, 123.4 (d, *J* = 2.8 Hz), 123.2, 114.7 (d, *J* = 21.5 Hz), 113.3 (d, *J* = 21.1 Hz), 49.2 (d, *J* = 1.7 Hz), 37.1, 33.8. **HRMS (ESI) m/z:** $[M+Na]^+$ Calcd for $C_{23}H_{18}FNO_2Na$ 382.1214; Found 382.1215. **IR υ_{max}:** 3015, 2917, 2891, 2832, 2385, 2245- 1970, 1695, 1578, 1404 cm^{-1}. **Melting point:** 89 – 90 °C. **HPLC:** Chiralpak IA, *n*-heptane:EtOH (70:30), 0.5 mL/min, λ = 210 nm, t_{R1} = 14.3 min, t_{R2} = 15.6 min. $[\alpha]_D$: - 1.9 (c 1.03, $CHCl_3$).

Preparation of (S)-2-(3-(2-methoxyphenyl)-3-phenylpropyl)isoindoline-1,3-dione (5f)

Following **GP4**, Ir-(*S*,*S*)-UbaPHOX (3.0 mg, 0.0017 mmol, 1 mol%), **3f** (62.1 mg, 0.168 mmol) and DCM (0.84 mL, 0.20 M) were used. Purification of the crude by flash chromatography (gradient from 0% to 15% of EtOAc in hexanes) afforded pure **5f** (58.4 mg, 99%, 99% ee, white solid).

AH hydrogenation of 5f at 0.5 mol% of catalyst loading

Following **GP4**, Ir-(*S*,*S*)-UbaPHOX (2.0 mg, 0.0011 mmol, 0.5 mol%), **3f** (85.4 mg, 0.22 mmol) and DCM (1.1 mL, 0.20 M) were used. Full conversion was observed by NMR and the product was not isolated (Full conversion, 98% ee).

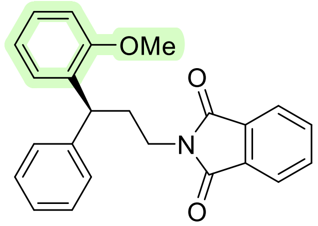

5f

^{1}H NMR (400 MHz, $CDCl_3$): δ 7.78 (m, 2H), 7.67 (m, 2H), 7.27 – 7.34 (m, 2H), 7.19 – 7.25 (m, 3H), 7.06 – 7.14 (m, 2H), 6.88 (td, *J* = 7.5, 1.2 Hz, 1H), 6.79 (dd, *J* = 8.2, 1.2 Hz, 1H), 4.50 (t, *J* = 7.8 Hz, 1H), 3.76 (s, 3H), 3.66 – 3.75 (m, 2H), 2.43 (m, 2H). **^{13}C NMR (101 MHz, $CDCl_3$):** δ 168.4, 156.9, 143.8, 133.8, 132.5, 132.3, 128.4, 128.2, 127.7, 127.4, 126.1, 123.1, 120.7, 110.8, 55.5, 41.5, 37.4, 33.1. **HRMS (ESI) m/z:** $[2M+Na]^+$ Calcd for $C_{48}H_{42}N_2O_6Na$ 765.2935; Found 765.2925. **IR υ_{max}:** 3010, 2929, 2242-1962, 1763, 1704, 1396 cm^{-1} **Melting point:** 149-151 °C. **HPLC:** Chiralcel ODH, *n*-heptane:IPA (9:1), 0.5 mL/min, λ = 220 nm, t_{R1} = 15.7 min, t_{R2} = 17.0 min. $[\alpha]_D$: - 12.0 (c 1.02, $CHCl_3$).

Preparation of (S)-2-(3-(2-chlorophenyl)-3-phenylpropyl)isoindoline-1,3-dione (5g)

Similarly to **GP4**, Ir-(*S*,*S*)-UbaPHOX (16.3 mg, 0.0094 mmol, 2 mol%), **3g** (175.7 mg, 0.47 mmol) and DCM (1.60 mL, 0.29 M) were used. The reaction was left under H_2 pressure for 64 h instead of overnight. Purification of the crude by flash chromatography (gradient from 0% to 10% of EtOAc in Cy) afforded pure **5g** (175.9 mg, 99%, 99% ee, white wax).

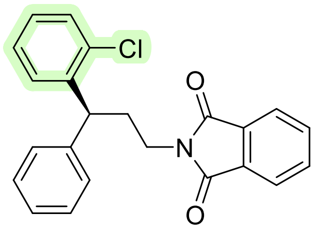

5g

^{1}H NMR (400 MHz, $CDCl_3$): δ 7.72 (m, 2H), 7.60 (m, 2H), 7.22 – 7.30 (m, 3H), 7.10 – 7.20 (m, 4H), 6.97 – 7.10 (m, 2H), 4.52 (t, *J* = 7.7 Hz, 1H), 3.56 – 3.74 (m, 2H), 2.26 – 2.47 (m, 2H). **^{13}C NMR (101 MHz, $CDCl_3$):** δ 168.4, 142.4, 141.3, 134.2, 134.0, 132.2, 130.0, 128.6, 128.4, 128.2, 127.7, 127.2, 126.6, 123.2, 44.9, 37.0, 33.7. **HRMS (ESI) m/z:** $[M+H]^+$ Calcd for $C_{23}H_{18}ClNO_2$ 376.1099; Found 376.1105. **IR υ_{max}:** 3463, 3059, 3026, 2939, 2245-1950, 1771, 1704, 1614, 1494, 1467, 1436, 1394, 1367, 1242 cm^{-1}. **HPLC:** Chiralpak IC, *n*-heptane:IPA (99:1), 0.5 mL/min, λ = 210 nm, t_{R1} = 31.0, min, t_{R2} = 36.5 min. **$[\alpha]_D$:** - 3.4 (c 1.00, $CHCl_3$).

Preparation of (S)-2-(3-(2-chloro-4-methoxyphenyl)-3-phenylpropyl)isoindoline-1,3-dione (5h)

Following **GP4**, Ir-(*S*,*S*)-UbaPHOX (3.0 mg, 0.0017 mmol, 1 mol%), **3h** (67.8 mg, 0.168 mmol) and DCM (0.84 mL, 0.2 M) were used. Purification of the crude by flash chromatography (gradient from 0% to 15% of EtOAc in hexanes) afforded **5h** (64.4 mg, 93%, 99% ee, colourless oil).

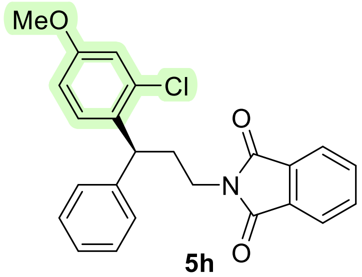

5h

^{1}H NMR (500 MHz, $CDCl_3$): δ 7.71 (dd, *J* = 5.4, 3.0 Hz, 2H), 7.60 (dd, *J* = 5.4, 3.0 Hz, 2H), 7.19 – 7.22 (m, 3H), 7.15 – 7.18 (m, 2H), 7.03 – 7.07 (m, 1H), 6.80 (d, *J* = 2.7 Hz, 1H), 6.70 (dd, *J* = 8.7, 2.7 Hz, 1H), 4.43 (t, *J* = 7.7 Hz, 1H), 3.66 (s, 3H), 3.55 – 3.66 (m, 2H), 2.25 – 2.40 (m, 2H). **^{13}C NMR (126 MHz, $CDCl_3$):** δ 168.3, 158.5, 143.0, 134.6, 133.9, 133.3, 132.3, 128.9, 128.6, 128.0, 126.5, 123.2, 115.1, 113.4, 55.6, 44.2, 37.0, 33.8. **HRMS (ESI) m/z:** $[2M+Na]^+$ Calcd for $C_{48}H_{40}Cl_2N_2O_6Na$ 833.218; Found 833.2153. **IR υ_{max}:** 3023, 2923, 2217-1961, 1771, 1699, 1394 cm^{-1}. **Melting point:** 54-55 °C. **HPLC**: Chiralpak IC, *n*-heptane:IPA (9:1), 0.5 mL/min, λ = 210 nm, t_{R1} = 14.7 min, t_{R2} = 18.2 min. $[\alpha]_D$: + 0.29 (c 0.92, $CHCl_3$), - 0.21 (c1.09, MeOH).

Preparation of (S)-2-(3-(2-methoxy-5-methylphenyl)-3-phenylpropyl)isoindoline-1,3-dione (5i)

Following **GP4**, Ir-(*S,S*)-UbaPHOX (2.5 mg, 0.0014 mmol, 1 mol%), **3i** (53.6 mg, 0.140 mmol) and DCM (0.7 mL, 0.2 M) were used. Purification of the crude by flash chromatography (gradient from 0% to 15% of EtOAc in hexanes) afforded pure **5i** (70.0 mg, 99%, 99% ee, colourless oil).

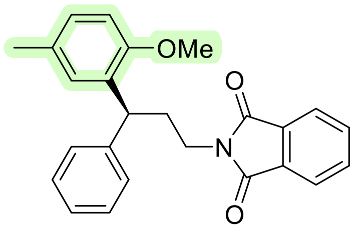

5i

^{1}H NMR (400 MHz, CDCl$_3$): δ 7.69 (dd, *J* = 5.4, 3.0 Hz, 2H), 7.58 (dd, *J* = 5.5, 3.0 Hz, 2H), 7.23 (d, *J* = 6.7 Hz, 2H), 7.15 (dd, *J* = 15.2, 7.6 Hz, 3H), 6.99 – 7.05 (m, 1H), 6.94 (d, *J* = 2.2 Hz, 1H), 6.78 (dd, *J* = 8.3, 2.2 Hz, 1H), 6.59 (d, *J* = 8.2 Hz, 1H), 4.39 (t, *J* = 7.7 Hz, 1H), 3.65 (s, 3H), 2.13 (s, 3H), 3.59 – 3.63 (m, 2H), 2.35 (q, *J* = 7.5 Hz, 2H). **^{13}C NMR (101 MHz, CDCl$_3$):** δ 168.4, 154.8, 144.0, 133.8, 132.3, 132.2, 129.8, 128.4, 128.4, 128.2, 127.6, 126.1, 123.1, 110.9, 55.7, 41.6, 37.4, 33.0, 20.8. **HRMS (ESI) m/z:** [2M+Na]$^+$ Calcd for $C_{50}H_{46}N_2O_6Na$ 793.3248; Found 793.3248. **IR υ_{max}:** 3020, 2927, 2200-1973, 1769, 1704, 1393 cm^{-1}. **HPLC**: Chiralpak IC, *n*-heptane:IPA (98:2), 0.5 mL/min, λ = 220 nm, t_{R1} = 23.9 min, t_{R2} = 25.7 min. [**α**]$_D$: + 5.46 (c 0.75, CHCl$_3$).

Preparation of (R)-2-(3-(2-methoxy-5-methylphenyl)-3-phenylpropyl)isoindoline-1,3-dione (R-5i)

Similarly to **GP4**, Ir-(*R,R*)-UbaPHOX instead of the (*S,S*) enantiomer (29.4 mg, 0.017 mmol, 1 mol%), **3i** (650.0 mg, 1.7 mmol) and DCM (7.5 mL, 0.23 M) were used. Purification of the crude by flash chromatography (gradient from 0% to 15% of EtOAc in Cy) afforded pure (***R*)-5i** (653.0 mg, 99%, 99% ee, colourless oil).

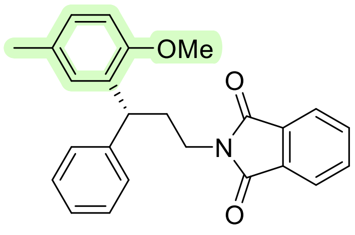

***R*-5i**

Spectroscopic analysis matches with **5i**. **HPLC**: Chiralpak IC, *n*-heptane:IPA (98:2), 0.5 mL/min, λ = 220 nm, t_{R1} = 35.8 min, t_{R2} = 39.5 min. [**α**]$_D$: -1.92 (c 0.70, CHCl$_3$).

Preparation of (S)-2-(3-(naphthalen-1-yl)-3-phenylpropyl)isoindoline-1,3-dione (5j)

Following **GP4**, Ir-(*S*,*S*)-UbaPHOX (2.7 mg, 0.0015 mmol, 1 mol%), **3j** (58.9 mg, 0.152 mmol) and DCM (0.76 mL, 0.2 M) were used. Purification of the crude by flash chromatography (gradient from 0% to 15% of EtOAc in hexanes) afforded pure **5j** (53.1 mg, 90%, 98% ee, white solid).

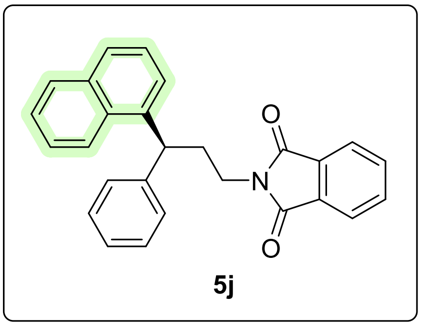

5j

^{1}H NMR (400 MHz, $CDCl_3$): δ 8.05 (d, *J* = 8.2 Hz, 1H), 7.63 – 7.75 (m, 3H), 7.53 – 7.65 (m, 3H), 7.48 (d, *J* = 7.2 Hz, 1H), 7.27 (d, *J* = 7.6 Hz, 2H), 7.36 (m, 3H), 7.11 – 7.19 (m, 2H), 7.02 (t, *J* = 7.3 Hz, 1H), 4.78 (t, *J* = 7.5 Hz, 1H), 3.72 (m, 2H), 2.41 – 2.59 (m, 2H). **^{13}C NMR (101 MHz, $CDCl_3$):** δ 168.4, 143.9, 139.3, 134.2, 133.9, 132.2, 131.8, 129.0, 128.7, 127.3, 128.1, 126.5, 126.2, 125.6, 125.5, 124.4, 123.7, 123.2, 44.4, 37.3, 34.8. **HRMS (ESI) m/z:** $[M+Na]^+$ Calcd for $C_{27}H_{21}NO_2Na$ 414.1465; Found 414.1472. **IR υ_{max}:** 3021, 2928, 2253-1976, 1762, 1703, 1396 cm^{-1}. **Melting point:** 128-129 °C. **HPLC**: Chiralpak IA, *n*-heptane:IPA (98:2), 0.5 mL/min, λ = 210 nm, t_{R1} = 17.7 min, t_{R2} = 22.0 min.$[\alpha]_D$: + 19.1 (c 1.08, $CHCl_3$).

4.5. General procedure 5: Deprotection of 3,3-diarylpropyl phthalimides 5.

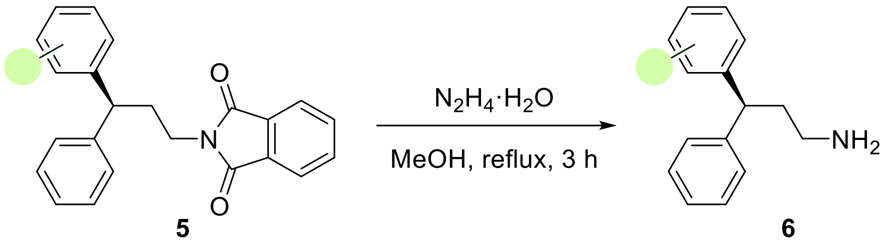

$N_2H_4 \cdot H_2O$ (64%, 2.0 – 4.0 equiv.) was added to a solution of the corresponding chiral 3,3-diarylpropyl phthalimide **5** (1.0 equiv.) in MeOH (0.16 M) and the resulting mixture was stirred at reflux for 3 h. Upon completion, the solvent was removed, H_2O added, and the pH adjusted to 12 with NaOH (1 M). Next, DCM was added, the mixture stirred for 20 min, and extracted. Finally, the aqueous phase was extracted with DCM 4 times, the organic layers collected, dried over anhydrous $MgSO_4$, filtered, and concentrated under reduced pressure to afford the desired product.

Preparation of (S)-3-phenyl-3-(p-tolyl)propan-1-amine (6b)

Following **GP5, 5b** (354.4 mg, 1.00 mmol, 1.0 equiv.), $N_2H_4 \cdot H_2O$ (64 %, 0.15 mL, 1.99 mmol, 2.0 equiv.) and MeOH (6.20 mL, 0.16 M) were used. After work-up, **6b** was obtained (224.7 mg, 99%, yellowish oil).

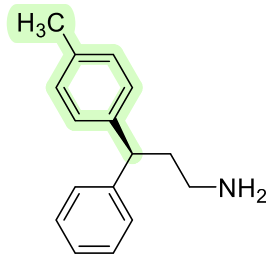

6b

^{1}H NMR (400 MHz, $CDCl_3$): δ 7.13 – 7.23 (m, 4H), 7.04 – 7.12 (m, 3H), 7.01 (d, *J* = 8.0 Hz, 2H), 3.91 (t, *J* = 7.7 Hz, 1H), 2.59 (s, 2H), 2.22 (br, 2H), 2.11 (q, *J* = 7.7 Hz, 2H), 1.33 (br, 2H). **^{13}C NMR (101 MHz, $CDCl_3$):** δ 145.1, 141.9, 135.8, 129.3, 128.6, 127.9, 127.8, 126.2, 48.5, 40.7, 39.6, 21.1. **HRMS (ESI) m/z:** $[M+H]^+$ Calcd for $C_{16}H_{20}N$ 226.1590; Found 226.1584. **IR ν_{max}:** 3364, 3271, 3082, 3053, 3022, 2922, 2858, 2395-1901, 1599, 1582, 1511, 1493, 1450, 1385, 1315 cm^{-1}. **$[\alpha]_D$:** + 4.4 (c 1.03, $CHCl_3$).

Preparation of (S)-3-(2-chlorophenyl)-3-phenylpropan-1-amine (6g)

Following **GP5, 5g** (189.1 mg, 0.50 mmol, 1.0 equiv.), $N_2H_4 \cdot H_2O$ (64 %, 0.15 mL, 2.00 mmol, 4.0 equiv.) and MeOH (3.10 mL, 0.16 M) were used. After work-up, **6g** was obtained (106.0 mg, 99%, yellowish oil).

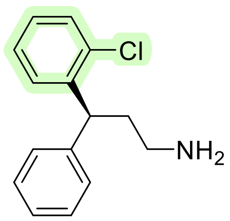

6g

^{1}H NMR (400 MHz, $CDCl_3$) δ 7.19 – 7.32 (m, 6H), 7.10 – 7.17 (m, 2H), 7.04 (ddd, *J* = 8.0, 7.3, 1.7 Hz, 1H), 4.52 (t, *J* = 7.8 Hz, 1H), 2.61 (t, *J* = 7.1 Hz, 2H), 2.02 – 2.21 (m, 2H). **^{13}C NMR (101 MHz, $CDCl_3$):** 143.3, 142.2, 134.2, 129.8, 128.7, 128.6, 128.2, 127.5, 127.1, 126.5, 44.2, 40.5, 39.4. **HRMS (ESI) m/z:** $[M+H]^+$ Calcd for $C_{15}H_{17}NCl$ 246.1044; Found 246.1049. **IR ν_{max}:** 3367, 3059, 3024, 2927, 2856, 2192-1924, 1802, 1664, 1590, 1493, 1469, 1440, 1389, 1258 cm^{-1}. **$[\alpha]_D$:** - 43,5 (c 1.04, $CHCl_3$).

Preparation of (S)-3-(2-chloro-4-methoxyphenyl)-3-phenylpropan-1-amine (6h)

Following **GP5, 5h** (442.0 mg, 1.09 mmol, 1.0 equiv.), $N_2H_4 \cdot H_2O$ (64 %, 0.17 mL, 3.5 mmol, 3.0 equiv.) and MeOH (7.0 mL, 0.16 M) were used. After work-up, **6h** was obtained (300.6 mg, 99%, yellowish oil).

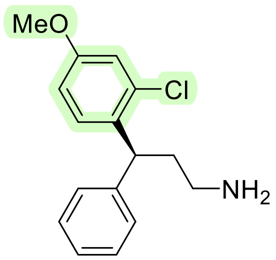

6h

^{1}H NMR (500 MHz, $CDCl_3$): δ 7.27 – 7.29 (m, 1H), 7.16 – 7.25 (m, 3H), 6.89 (d, *J* = 2.7 Hz, 1H), 6.78 (dd, *J* = 8.7, 2.7 Hz, 1H), 4.51 (t, *J* = 7.8 Hz, 1H), 3.76 (s, 3H), 2.70 (br, 2H), 2.18 (m, 2H), 1.85 (br, 2H). **^{13}C NMR (126 MHz, $CDCl_3$):** δ 158.4, 143.8, 134.6, 134.1, 129.1, 128.6, 128.1, 126.4, 114.9, 113.5, 55.6, 43.5, 40.4, 39.2. **HRMS (ESI) m/z:** $[M+H]^+$ Calcd for $C_{16}H_{19}ONCl$ 276.1150; Found 276.1154. **IR υ_{max}:** 3024, 2931, 2857, 2834, 2115, 2063, 1996, 1718, 1653, 1602, 1571, 1490, 1451, 1436, 1395, 1284, 1258, 1231, 1181, 1073, 1034 cm^{-1}. $[\alpha]_D$: - 36.2 (c 1.00, $CHCl_3$).

Preparation of (R)-3-(2-methoxy-5-methylphenyl)-3-phenylpropan-1-amine ((R)-6i)

Following **GP5**, (***R***)**-5i** (617.4 mg, 1.6 mmol, 1.0 equiv.), $N_2H_4 \cdot H_2O$ (64 %, 0.25 mL, 3.2 mmol, 2.0 equiv.) and MeOH (10.0 mL, 0.16 M) were used. After work-up, (***R***)**-6i** was obtained (421.4 mg, 99%, yellowish oil).

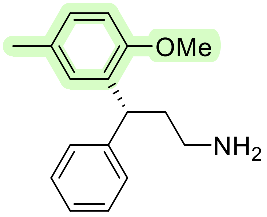

***R*-6i**

^{1}H NMR (500 MHz, $CDCl_3$): δ 7.26 – 7.31 (m, 3H), 7.23 – 7.26 (m, 1H), 7.15 (m, 1H), 6.99 (d, *J* = 2.2 Hz, 1H), 6.95 (ddd, *J* = 8.3, 2.3, 0.8 Hz, 1H), 6.73 (d, *J* = 8.2 Hz, 1H), 4.49 (t, *J* = 7.9 Hz, 1H), 3.76 (s, 3H), 2.66 (m, 2H), 2.25 (s, 3H), 2.11 – 2.18 (m, 2H), 1.46 (br, 2H). **^{13}C NMR (126 MHz, $CDCl_3$):** δ 155.0, 145.0, 133.0, 129.9, 128.6, 128.3, 128.2, 127.5, 126.0, 110.9, 55.8, 40.8, 40.4, 39.1, 20.9. **HRMS (ESI) m/z:** $[M+H]^+$ Calcd for $C_{17}H_{22}ON$ 256.1696; Found 256.1695. **IR υ_{max}:** 3024, 2927, 2860, 2834, 1718, 1599, 1496, 1451, 1287, 1239, 1179, 1155, 1110 cm^{-1}. $[\alpha]_D$: + 21.3 (c 0.92, $CHCl_3$).

Preparation of (R)-N,N-diisopropyl-3-(2-methoxy-5-methylphenyl)-3-phenylpropan-1-amine (7)

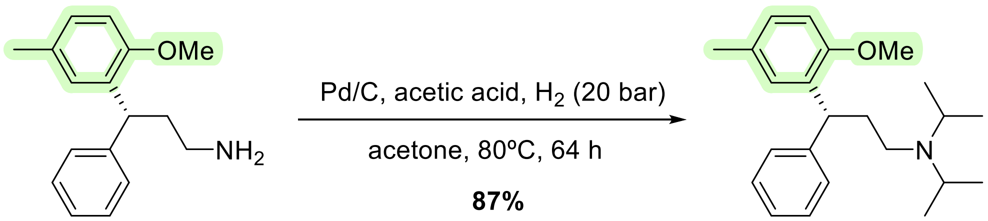

***R*-6i** **7**

(***R***)**-6i** (314.5 mg, 1.23 mmol, 1.0 equiv.), Pd/C moistened with water (10%, 62.9 mg, 10 mol%), and a PTFE-coated stir-bar were placed in a glass tube. Acetone (15.0 mL, 0.082 M) and glacial acetic acid (70 μL, 1.23 mmol, 1.0 equiv.) were added, and the glass tube was placed inside a

stainless-steel high-pressure reactor. The reactor was closed and connected to a hydrogen manifold. The valve of the reactor was opened and purged with vacuum-nitrogen cycles, vacuum-hydrogen cycles, and finally charged at 20 bar of hydrogen pressure. The valve was closed, the hydrogen manifold unplugged, and the mixture left to stir inside an oil bath at 80 °C for 64 h. The reactor was then depressurized, and the solution concentrated under reduced pressure. The resulting oil was redissolved in DCM, filtered through a pad of Celite and the solvent removed again under reduced pressure. Purification of the crude by flash chromatography (gradient from 0% to 10% of EtOAc in Cy containing 2% TEA) afforded pure **7** (364.4 mg, 87%, colourless oil).

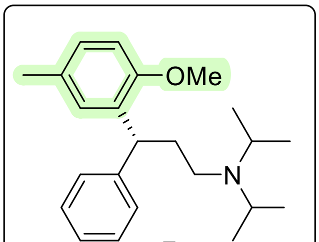

7

^{1}H NMR (500 MHz, $CDCl_3$): δ 7.27 – 7.30 (m, 2H), 7.23 – 7.26 (m, 2H), 7.06 (d, *J* = 2.4 Hz, 1H), 7.10 – 7.17 (m, 1H), 6.94 (ddd, *J* = 8.3, 2.4, 0.9 Hz, 1H), 6.71 (d, *J* = 8.3 Hz, 1H), 4.35 (t, *J* = 7.7 Hz, 1H), 3.74 (s, 3H), 2.97 (m, 2H), 2.30 – 2.37 (m, 2H), 2.26 (s, 3H), 2.08 – 2.16 (m, 2H), 0.93 (dd, *J* = 6.5, 2.2 Hz, 12H). **^{13}C NMR (126 MHz, $CDCl_3$):** δ 155.1, 145.3, 133.7, 129.7, 128.5, 128.4, 128.2, 127.3, 125.8, 110.8, 55.7, 48.9, 44.3, 41.5, 37.2, 20.9, 20.7. **IR ν_{max}:** 2961, 2929, 2868, 2832, 1718, 1600, 1496, 1451, 1384, 1287, 1239, 1202, 1161, 1112, 1034 cm^{-1}. $[\alpha]_D$: - 7.9 (c 1.00, $CHCl_3$). Spectroscopic analysis matches with previously reported data.[5]

Preparation of (R)-2-(3-(diisopropylamino)-1-phenylpropyl)-4-methylphenol ((R)-Tolterodine, 8)

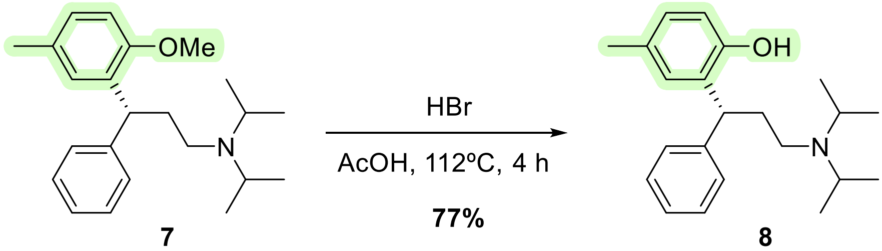

A N_2-purged solution of **8** (179.0 mg, 0.53 mmol, 1.0 equiv.) in AcOH (1.3 mL, 0.45 M) was treated with a solution of HBr 33% in AcOH (1.3 mL, 7.79 mmol, 13.2 equiv.). The reaction mixture was stirred at 112 °C for 4 h. Upon completion, the mixture was cooled to room temperature, diluted with water and extracted with EtOAC two times. All organic fractions were collected, washed with NaOH 1 M three times (until pH >10) and with brine two times. The organic layer was dried over anhydrous $MgSO_4$, filtered, and concentrated under reduced pressure. Purification of the crude by

flash chromatography (gradient from 0% to 10% of EtOAc in Cy containing 2% TEA) afforded pure **8** (131.7 mg, 77%, yellowish oil).

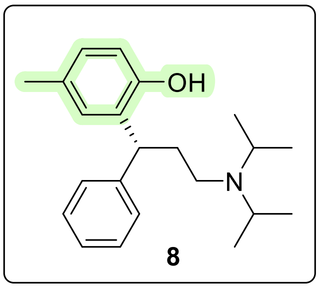

8

^{1}H NMR (500 MHz, $CDCl_3$): δ 7.32 (d, *J* = 4.3 Hz, 4H), 7.19 – 7.25 (m, 1H), 6.85 (ddd, *J* = 8.2, 2.2, 0.7 Hz, 1H), 6.80 (d, *J* = 8.1 Hz, 1H), 6.53 (d, *J* = 2.1 Hz, 1H), 4.48 (dd, *J* = 11.3, 4.1 Hz, 1H), 3.23 (hept, *J* = 6.7 Hz, 2H), 2.69 – 2.76 (m, 1H), 2.30 – 2.42 (m, 2H), 2.11 (s, 3H), 2.01 – 2.10 (m, 1H), 1.10 (dd, *J* = 27.5, 6.7 Hz, 12H). **^{13}C NMR (126 MHz, $CDCl_3$):** δ 153.3, 144.9, 132.5, 129.5, 128.8, 128.6, 128.4, 127.9, 126.3, 118.3, 48.2, 42.3, 39.5, 33.4, 19.7, 20.9, 20.1. **IR υ_{max}:** 3185, 3024, 2963, 2926, 2866, 1802, 1772, 1750, 1718, 1699, 1684, 1669, 1600, 1492, 1449, 1388, 1360, 1326, 1250, 1203, 1161, 1133, 1105, 1030 cm^{-1}. **$[\alpha]_D$:** + 21.6 (c 0.95, MeOH). Spectroscopic analysis matches with previously reported data.[5]

Preparation of (S)-N,N-dimethyl-3-phenyl-3-(p-tolyl)propan-1-amine (S-Tolpropamine, 9)

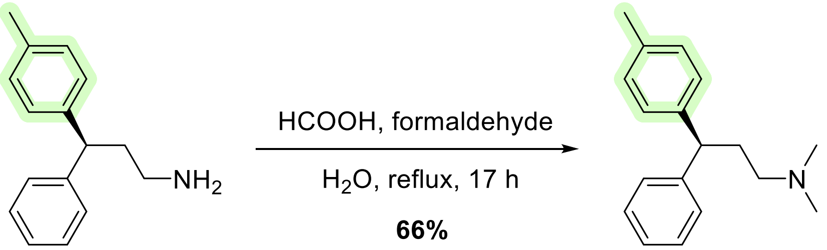

6b **9**

A pressure tube containing **6b** (77.1 mg, 0.34 mmol, 1.0 equiv.), formic acid (74 μL, 1.71 mmol, 5.0 equiv.) and formaldehyde (36% in H_2O, 3.0 mL) was heated at 100 °C for 17 h. Upon reaction completion H_2O was added and the pH adjusted to 12 with NaOH (40%). Then the solution was extracted with DCM 4 times, the organic layers collected, dried over anhydrous $MgSO_4$, filtered, and concentrated under reduced pressure. Purification of the crude by flash chromatography (gradient from 0% to 10% of EtOAc in Cy containing 2% TEA) afforded pure **9** (57.5 mg, 66%, colourless oil).

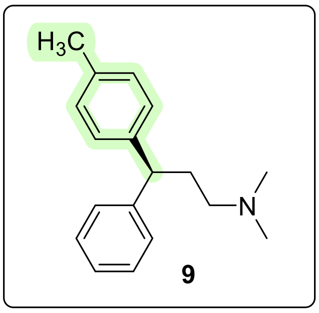

^{1}H NMR (400 MHz, $CDCl_3$): δ 7.13 – 7.23 (m, 4H), 7.04 – 7.12 (m, 3H), 7.01 (d, *J* = 8.2 Hz, 2H), 3.82 – 3.92 (br, 1H), 2.22 (s, 3H), 2.10 – 2.15 (m, 10H). **^{13}C NMR (101 MHz, $CDCl_3$):** δ 145.3, 142.0, 135.8, 129.3, 128.6, 127.9, 127.8, 126.2, 58.3, 48.8, 45.7, 33.8, 21.1. **HRMS (ESI) m/z:** $[M+H]^+$ Calcd for $C_{18}H_{24}N$ 254.1903; Found 254.1897. **IR υ_{max}:** 3083, 3058, 3023, 2968, 2940, 2855, 2613, 2762, 2360, 2342, 2176-1959, 1599, 1511, 1492, 1451, 1377, 1263, 1223 cm^{-1}. **$[\alpha]_D$:** + 3.0 (c 0.97, $CHCl_3$). Spectroscopic analysis matches with previously reported data.[4]

4.6. General procedure 6: Cyclization of 3,3-diarylpropyl amines 6.

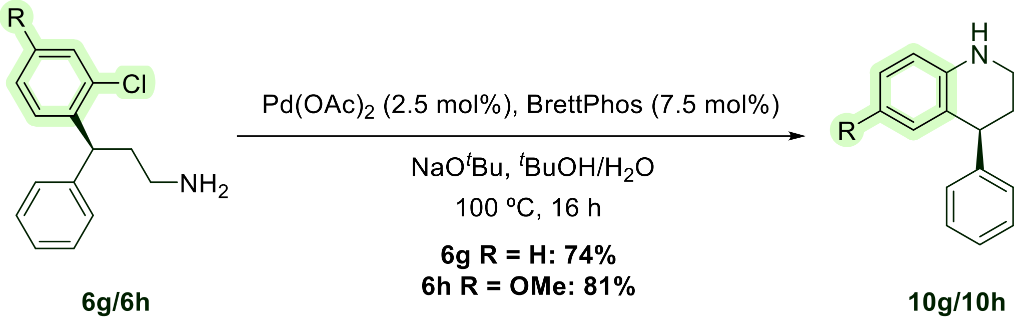

An oven-dried vial was charged with $Pd(OAc)_2$ (2.5 mol%) and BrettPhos (7.5 mol%). The vial was sealed and purged with vacuum-nitrogen cycles. Then, *t*BuOH and degassed H_2O (18 mol%) were sequentially added and the solution was heated at 110 °C for 1 min or less (until a colour change is observed). The resulting mixture was transferred via cannula to an oven-dried and N_2 purged Schlenk tube containing the corresponding 3,3-diarylpropyl amine (1 equiv.) and NaO*t*Bu (1.2 equiv.) solved in anhydrous *t*BuOH. The reaction was heated at 110 °C for 16 h. Upon completion, the reaction was cooled down to room temperature, diluted with EtOAc, H_2O added, stirred for 10 minutes and phases separated. The aqueous phase was extracted with EtOAc 3 times, organic phases collected were collected, washed with brine, dried over anhydrous $MgSO_4$, filtered, and concentrated under reduced pressure. Purification of the crude by flash chromatography (gradient from 0% to 10% of EtOAc in Cy) afforded the desired product.

Preparation of (S)-4-phenyl-1,2,3,4-tetrahydroquinoline (10g)

Following **GP6**, **6g** (166.2 mg, 0.68 mmol, 1 equiv.), $Pd(OAc)_2$ (6.19 mg, 0.017 mmol, 2.5 mol%), BrettPhos (27.23 mg, 0.051 mmol, 7.5 mol%), NaO*t*Bu (77.99 mg, 0.81 mmol, 1.2 equiv.), H_2O (2.2 μL, 0.12 mmol, 18 mol%) and *t*BuOH (4.5 mL, 0.15 M) were used. After work-up and purification, **10g** was obtained (101.0 mg, 74%, white solid).

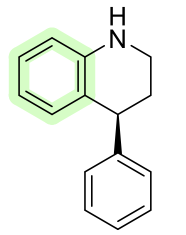

10g

^{1}H NMR (400 MHz, $CDCl_3$): δ 7.24 – 7.34 (m, 2H), 7.18 – 7.24 (m, 1H), 7.12 – 7.17 (m, 2H), 7.01 (m, 1H), 6.75 (d, *J* = 6.7 Hz, 1H), 6.53 – 6.61 (m, 2H), 4.15 (t, *J* = 6.1 Hz, 1H), 3.95 (br, 1H), 3.19 – 3.36 (m, 2H), 2.16 – 2.28 (m, 1H), 2.00 – 2.12 (m, 1H). **^{13}C NMR (101 MHz, $CDCl_3$):** δ 146.8, 145.1, 130.6, 128.8, 128.4, 127.4, 126.2, 123.5, 117.2, 114.3, 43.0, 39.3, 31.2. **HRMS (ESI) m/z:** $[M+H]^+$ Calcd for $C_{15}H_{16}N$ 210.1277; Found 210.1272. **IR υ_{max}:** 3405 3009, 2949, 2918, 2851, 2397, 2236-1957, 1727, 1605, 1582, 1502, 1491, 1448, 1354, 1314, 1271, 1248 cm^{-1}. **Melting point:** 73 – 76 °C. **$[\alpha]_D$:** + 70.3 (c 0.97, $CHCl_3$). Spectroscopic data matches with previously reported.[6]

Preparation of (S)-6-methoxy-4-phenyl-1,2,3,4-tetrahydroquinoline (10h)

Following **GP6**, **6h** (200.0 mg, 0.73 mmol, 1 equiv.), $Pd(OAc)_2$ (4.1 mg, 0.018 mmol, 2.5 mol%), BrettPhos (29.2 mg, 0.054, 7.5 mol%), NaO*t*Bu (73.8 mg, 0.87 mmol, 1.2 equiv.), H_2O (2.4 μL, 0.13 mmol, 18 mol%) and *t*BuOH (4.8 mL, 0.15 M) were used. After work-up and purification, **10h** was obtained (140.3 mg, 81%, yellow solid).

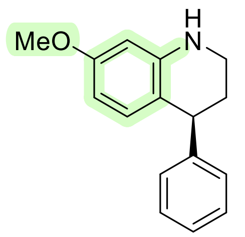

10h

^{1}H NMR (500 MHz, $CDCl_3$): δ 7.27 – 7.32 (m, 2H), 7.18 – 7.22 (m, 1H), 7.11 – 7.17 (m, 2H), 6.65 (dd, *J* = 8.4, 0.9 Hz, 1H), 6.17 (dd, *J* = 8.4, 2.5 Hz, 1H), 6.11 (d, *J* = 2.5 Hz, 1H), 4.09 (t, *J* = 6.1 Hz, 1H), 3.95 (br, 1H), 3.75 (s, 3H), 3.18 – 3.32 (m, 2H), 2.19 (m, 1H), 2.03 (m, 1H). **^{13}C NMR (126 MHz, $CDCl_3$):** δ 159.3, 147.0, 146.0, 131.4, 128.8, 128.4, 126.2, 116.4, 103.3, 99.2, 55.2, 42.4, 39.3, 31.5. **HRMS (ESI) m/z:** $[M+H]^+$ Calcd for $C_{16}H_{18}ON$ 240.1383; Found 240.1381. **IR υ_{max}:** 3397, 2989, 2955, 2929, 2853, 2832, 1615, 1576, 1500, 1459, 1353, 1317, 1287, 1250, 1202, 1164, 1131, 1034 cm^{-1}. **Melting point:** 72 – 73 °C. **$[\alpha]_D$:** + 56.7 (c 1.00, $CHCl_3$)

5. Stereochemical course of the asymmetric hydrogenation

The stereochemical course of the asymmetric hydrogenation using Ir-(*S*,*S*)-UbaPHOX was predicted with Andersson's quadrant model.[7,8] Since there is no X-ray structure of Ir-(S,S)-UbaPHOX available in the literature, a similar crystal structure was analyzed (CCDC-167319).[9]

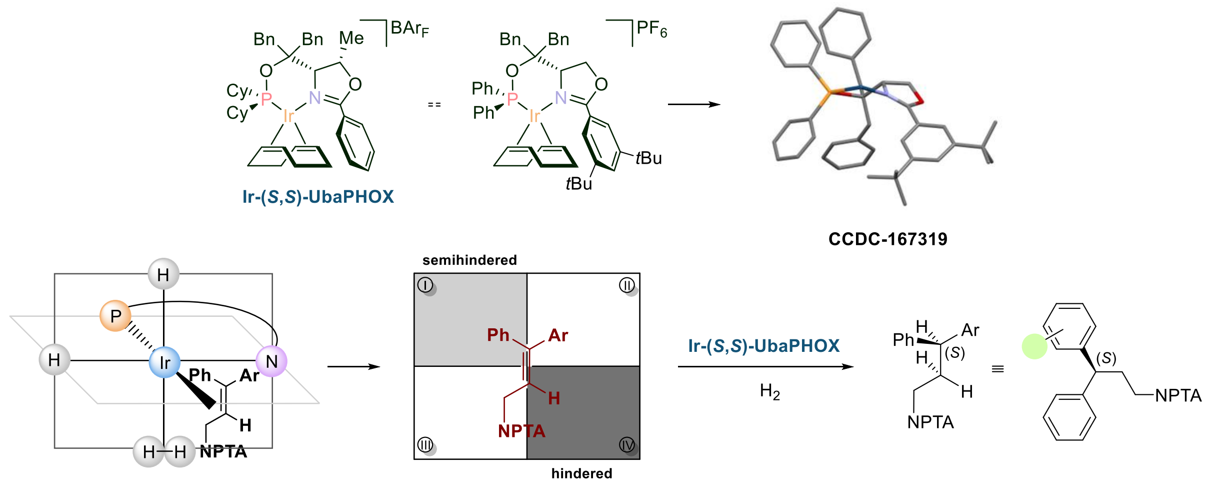

The model begins by examining the chiral three-dimensional structure of the catalyst through X-ray analysis (hydrogens, COD and counterion are omitted for clarity). The catalytic system is then divided into four quadrants around the metal center, and steric hindrance is analyzed within each quadrant. It is observed that the fourth quadrant experiences the highest hindrance due to the presence of the disubstituted-phenyl group in the oxazoline moiety. Additionally, the first quadrant, influenced by one of the phosphorus substituents, exhibits the second highest hindrance. The remaining two quadrants are considered open/unhindered.

The, the olefin coordinates perpendicularly to the plane defined by the chiral P,N-ligand, *cis* to the nitrogen and *trans* to the phosphorus. To minimize steric interactions, the alkene preferentially coordinates with the smallest substituent (hydrogen) close to the most hindered site. At last, hydrogen insertion occurs from behind the substrate, leading to the formation of one enantiomer. Using this model, it was concluded that the hydrogenated products in the current work correspond to the *S*-enantiomer. By comparing the optical rotation of **10g** with the literature,[6] the prediction obtained with the model was confirmed.

6. Failed asymmetric hydrogenations

The asymmetric hydrogenation of substrates containing acetyl, furan and tiophene moieties was attempted without success. Here in the results, it can be observed that they provided low conversions and low selectivity. We hypothesize that this is most likely due to the coordination of these moieties to the iridium center resulting in catalyst deactivation.

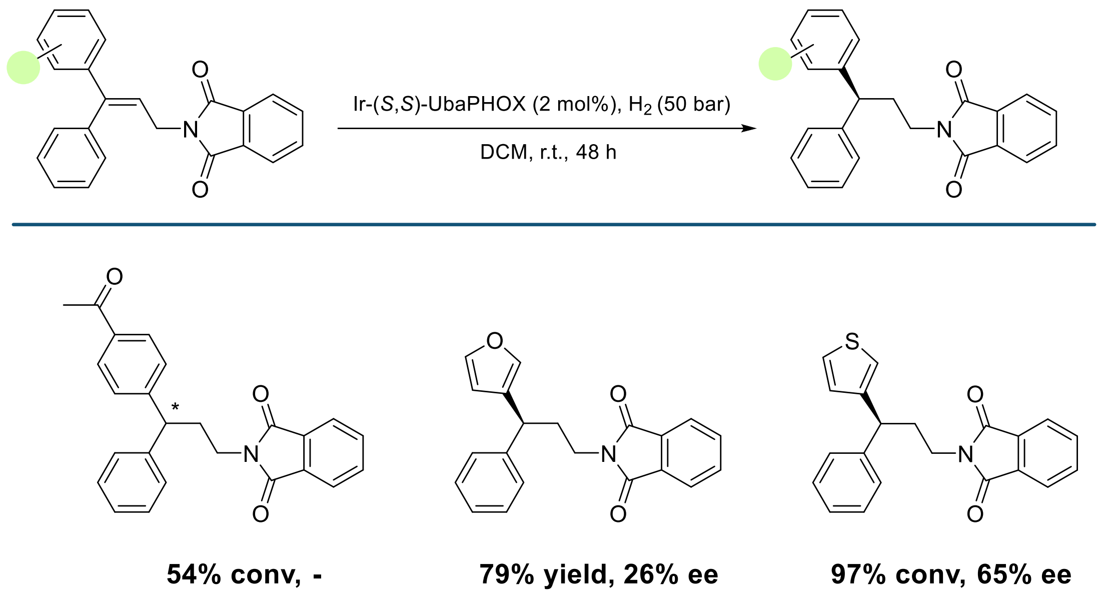

7. References

1. Limberger, J.; Claudino, T. S.; Monteiro, A. L. Stereoselective Synthesis of (*E*)-3,3-Diaryl and (*E*)-3-Aryl-3-Aryloxy Allylamines and Allylalcohols from Trans-Cinnamyl Chloride and Alcohol. *RSC Adv.* **2014**, *4*, 45558–45565.
2. He, Z.; Kirchberg, S.; Fröhlich, R.; Studer, A. Oxidative Heck Arylation for the Stereoselective Synthesis of Tetrasubstituted Olefins Using Nitroxides as Oxidants. *Angew. Chem. Int. Ed.* **2012**, *51*, 3699–3702.
3. Zhang, J.; Chen, T.; Wang, Y.; Zhou, F.; Zhang, Z.; Gridnev, I. D.; Zhang, W. Asymmetric Hydrogenation of γ -branched Allylamines for the Efficient Synthesis of γ -chirogenic Amines. *Nat. Sci.* **2021**, *1*, e10021.
4. Gu, F.; Huang, W.; Liu, X.; Chen, W.; Cheng, X. Substituted Hantzsch Esters as Versatile Radical Reservoirs in Photoredox Reactions. *Adv. Synth. Catal.* **2018**, *360*, 925–931.
5. Roesner, S.; Aggarwal, V. K. Enantioselective Synthesis of (*R*)-Tolterodine Using Lithiation/Borylation–Protodeboronation Methodology. *Can. J. Chem.* **2012**, *90*, 965–974.
6. Rueping, M.; Theissmann, T.; Stoeckel, M.; Antonchick, A. P. Direct Enantioselective Access to 4-Substituted Tetrahydroquinolines by Catalytic Asymmetric Transfer Hydrogenation of Quinolines. *Org. Biomol. Chem.* **2011**, *9*, 6844–6850.
7. Church, T. L.; Rasmussen, T.; Andersson, P. G. Enantioselectivity in the Iridium-Catalyzed Hydrogenation of Unfunctionalized Olefins. *Organometallics* **2010**, *29*, 6769–6781.
8. Källström, K.; Hedberg, C.; Brandt, P.; Bayer, A.; Andersson, P. G. Rationally Designed Ligands for Asymmetric Iridium-Catalyzed Hydrogenation of Olefins. *J. Am. Chem. Soc.* **2004**, *126*, 14308–14309.
9. Blankenstein, J.; Pfaltz, A. A New Class of Modular Phosphinite–Oxazoline Ligands: Ir-Catalyzed Enantioselective Hydrogenation of Alkenes. *Angew. Chem. Int. Ed.* **2001**, *40*, 4445–4447.

8. NMR spectra of compounds

Spectra of S1:

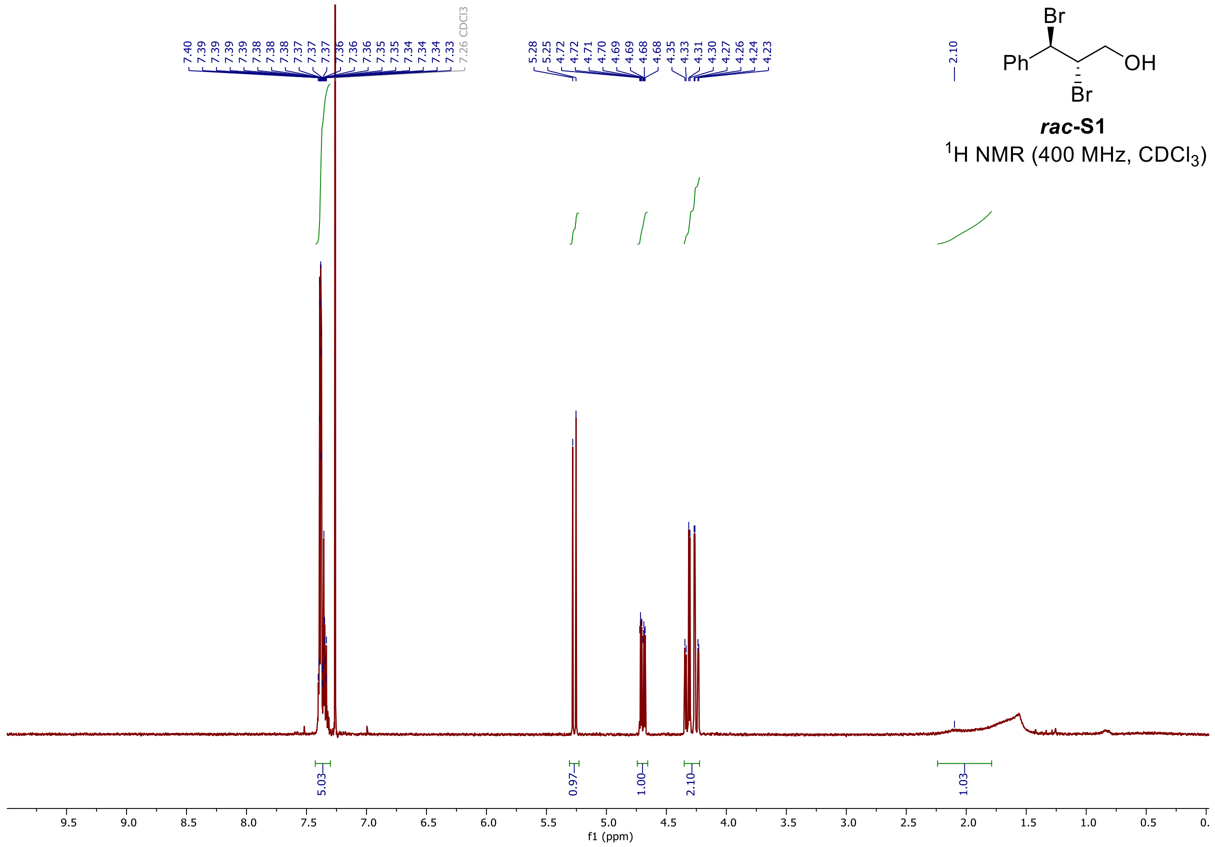

Spectra of 1:

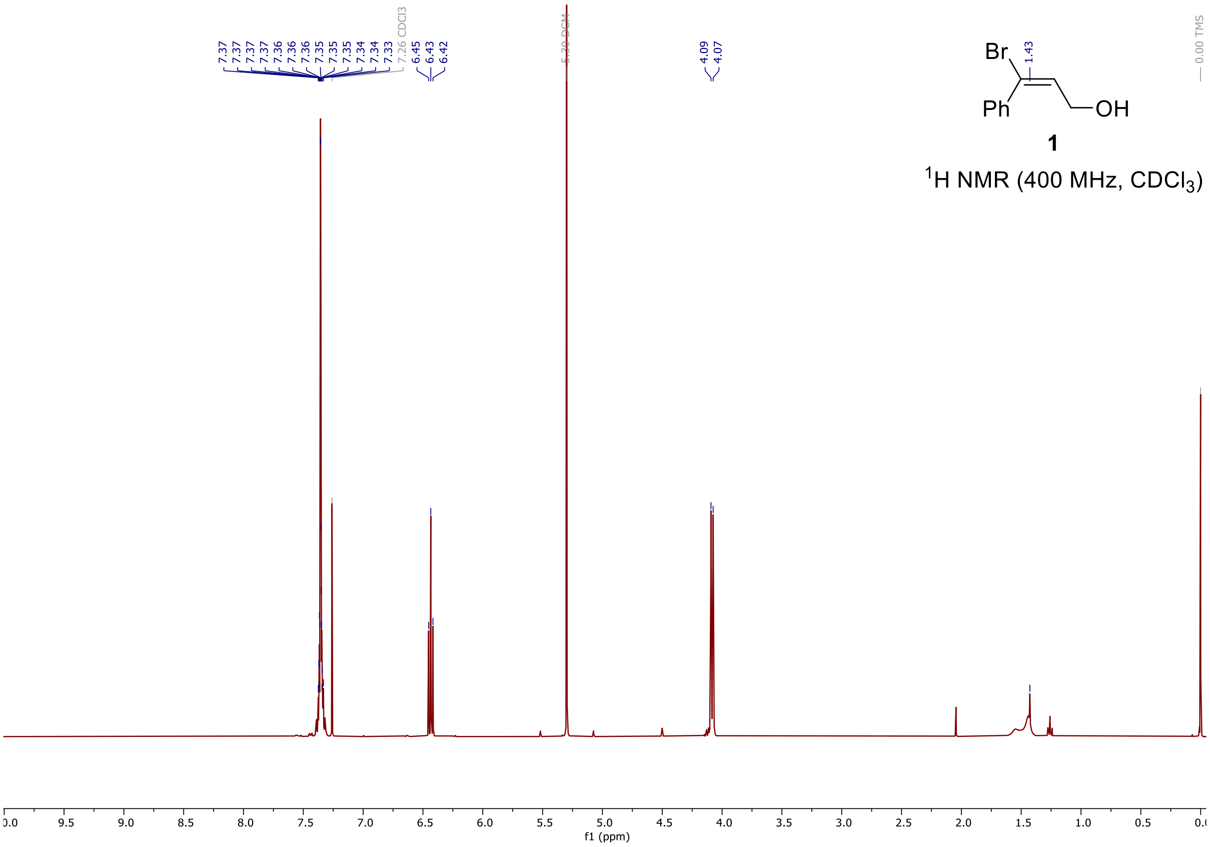
7.37
7.37
7.37
7.37
7.36
7.36
7.36
7.35
7.35
7.35
7.35
7.34
7.34
7.33
7.26 CDCl3
6.45
6.43
6.42
5.30 DCM
4.09
4.07
1.43
0.00 TMS
Br
Ph
OH
1
^{1}H NMR (400 MHz, $CDCl_3$)
f1 (ppm)

Spectra of 2:

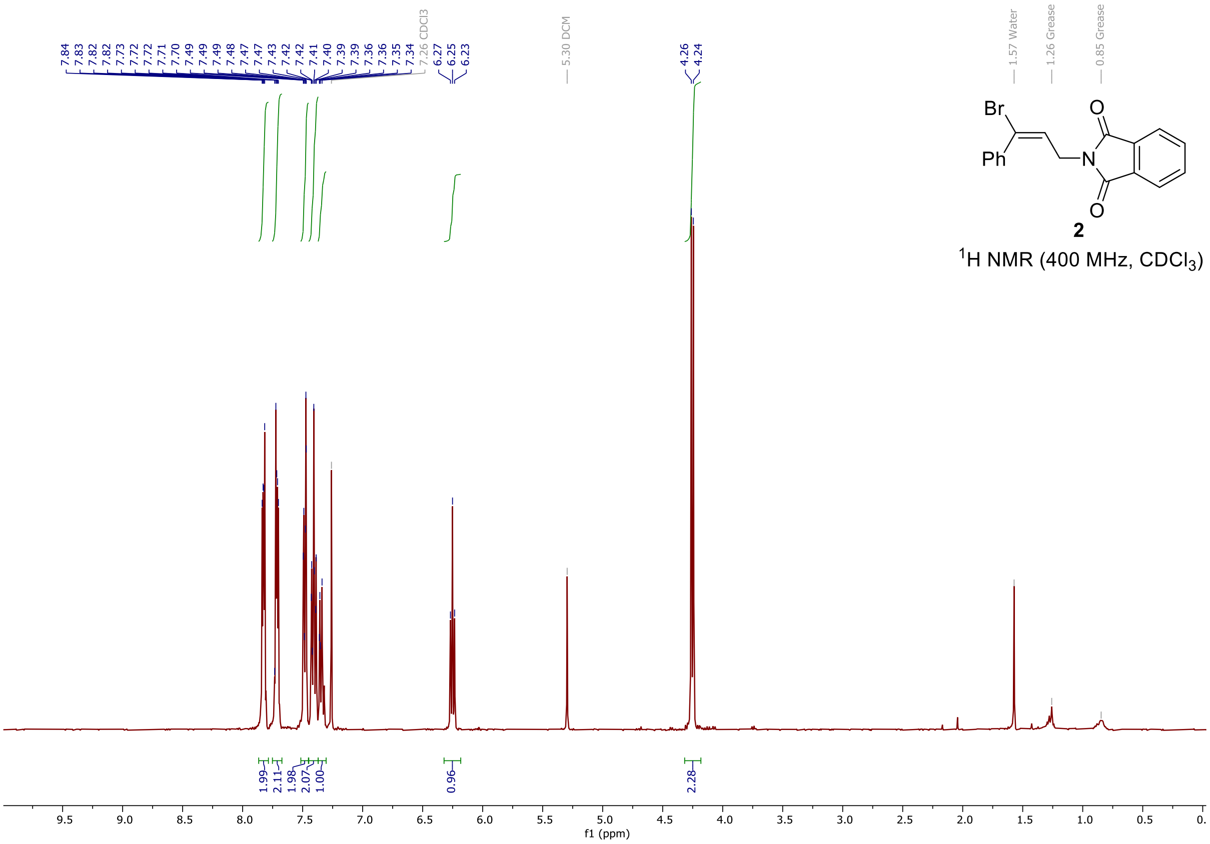

^{1}H NMR (400 MHz, $CDCl_3$)

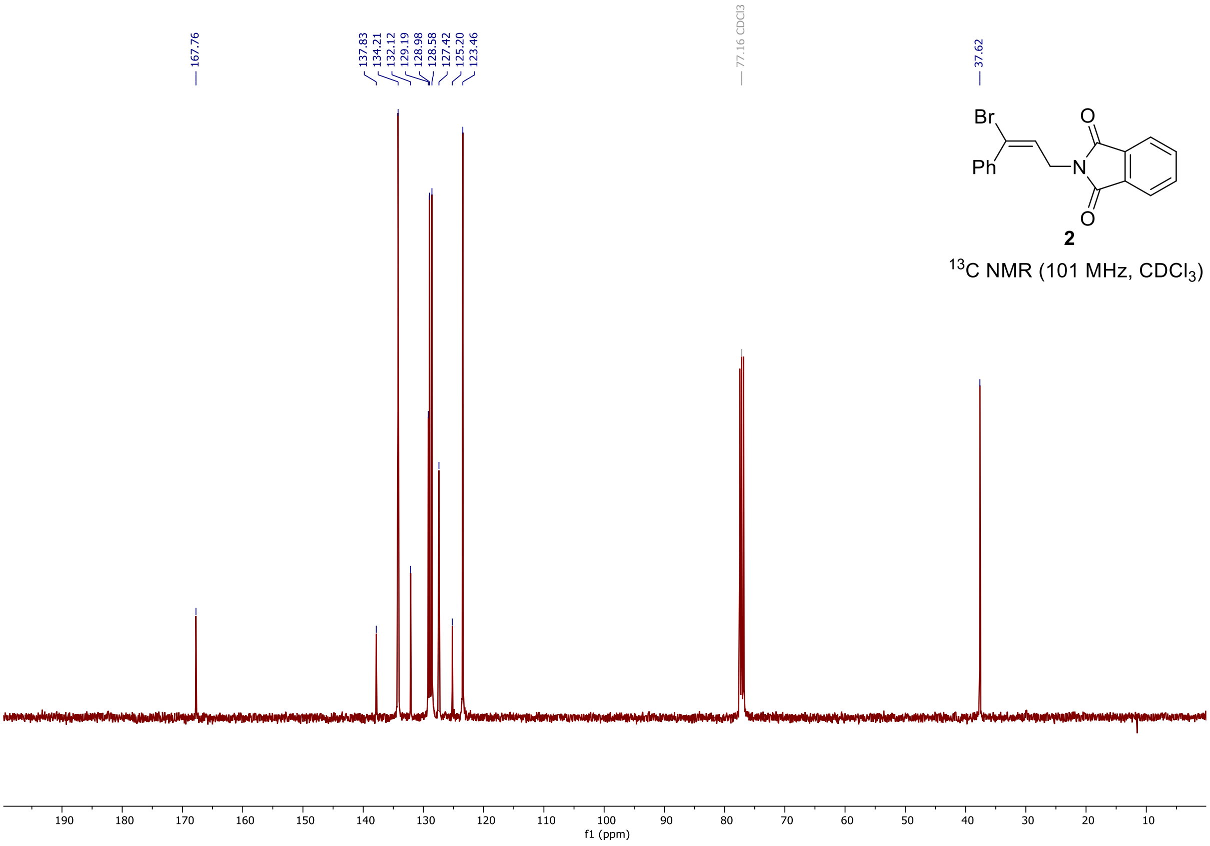

^{13}C NMR (101 MHz, $CDCl_3$)

Spectra of 3a:

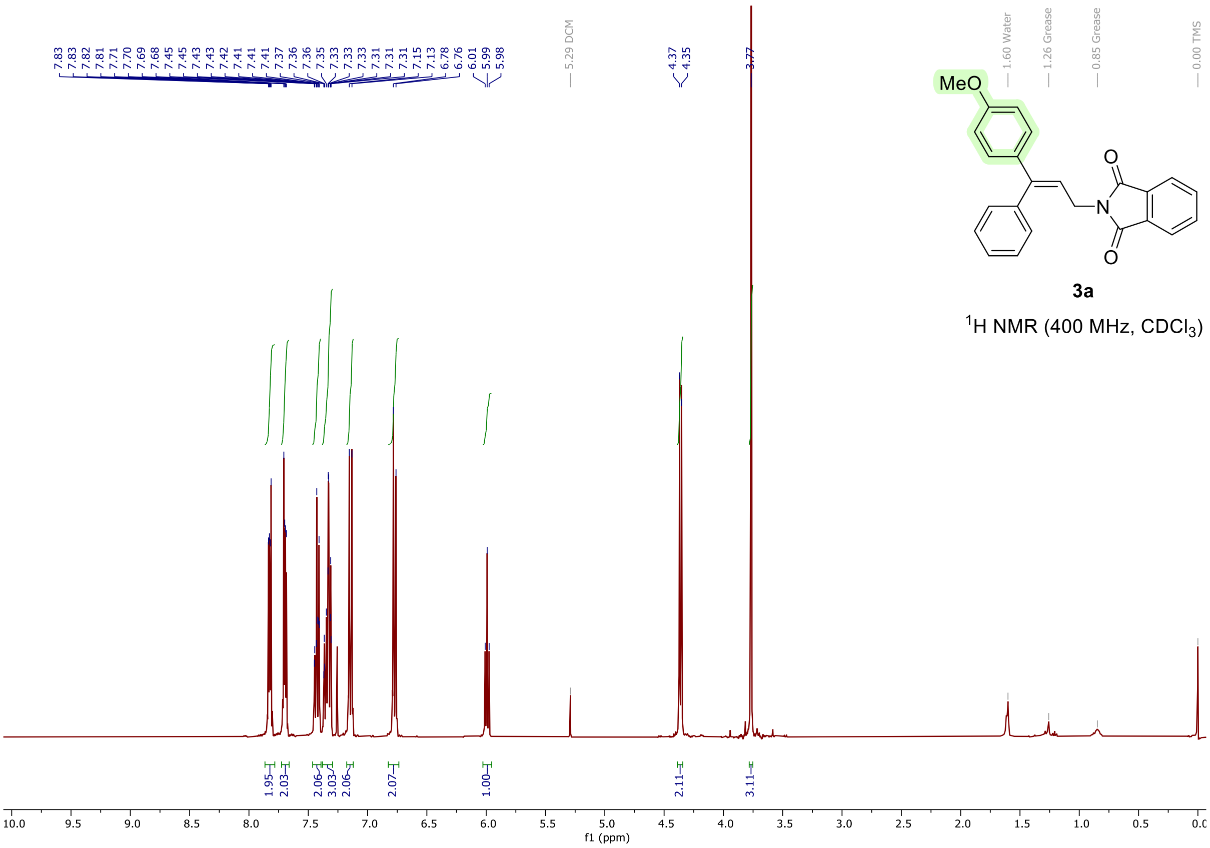

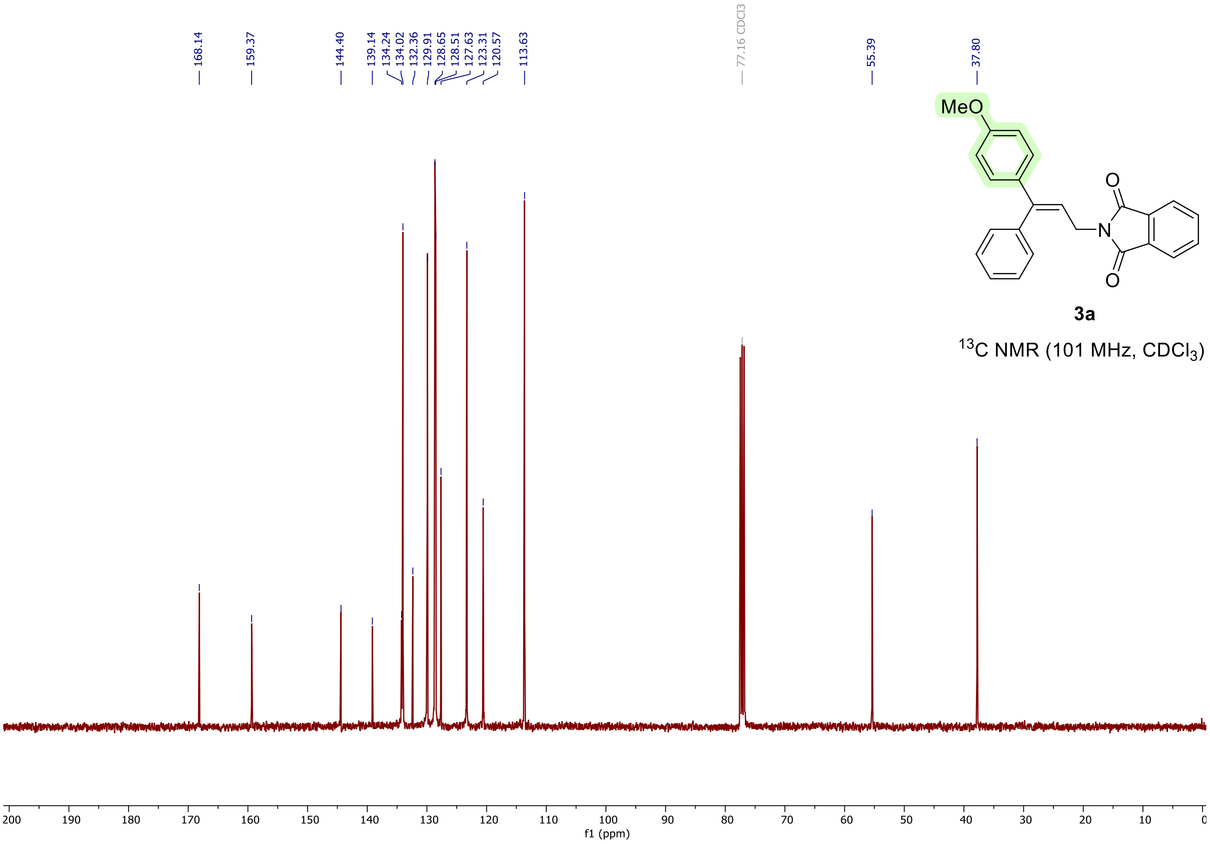

Spectra of 3b:

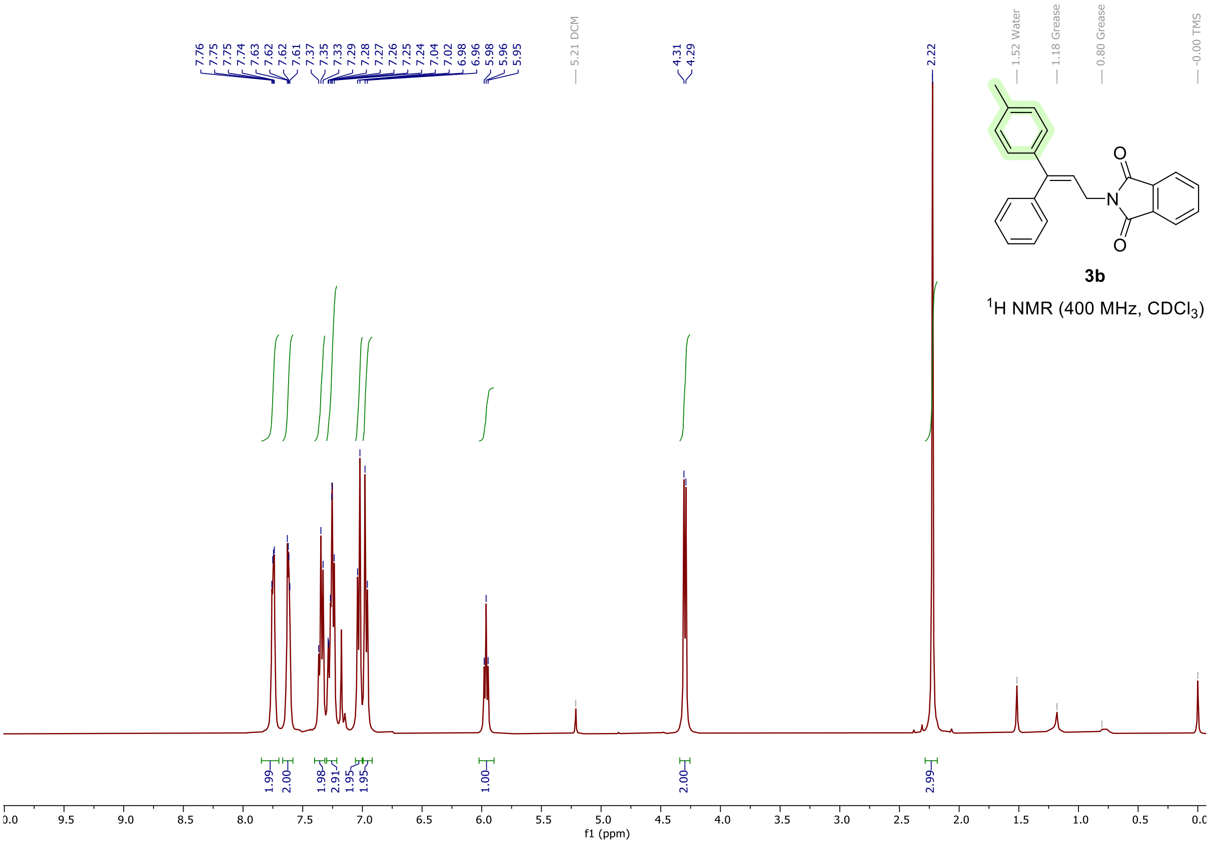

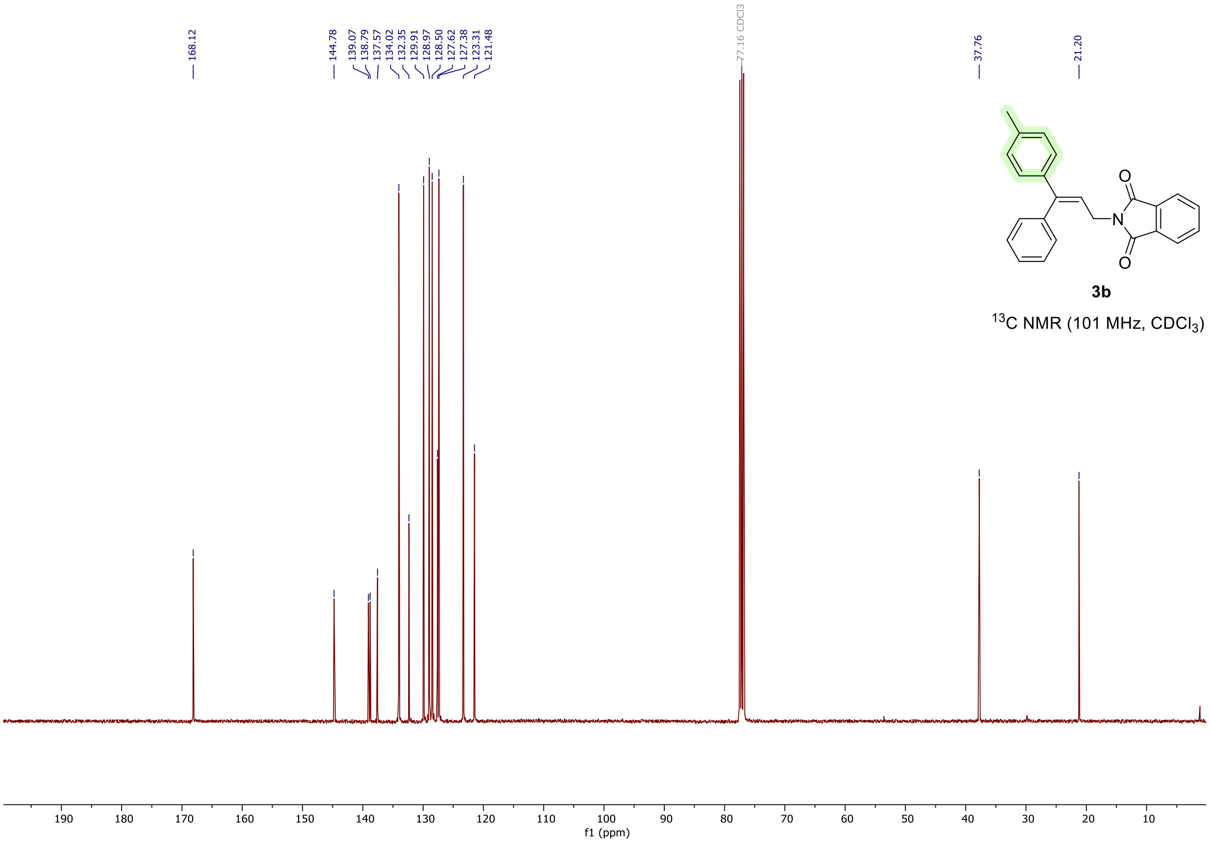

Spectra of 3c:

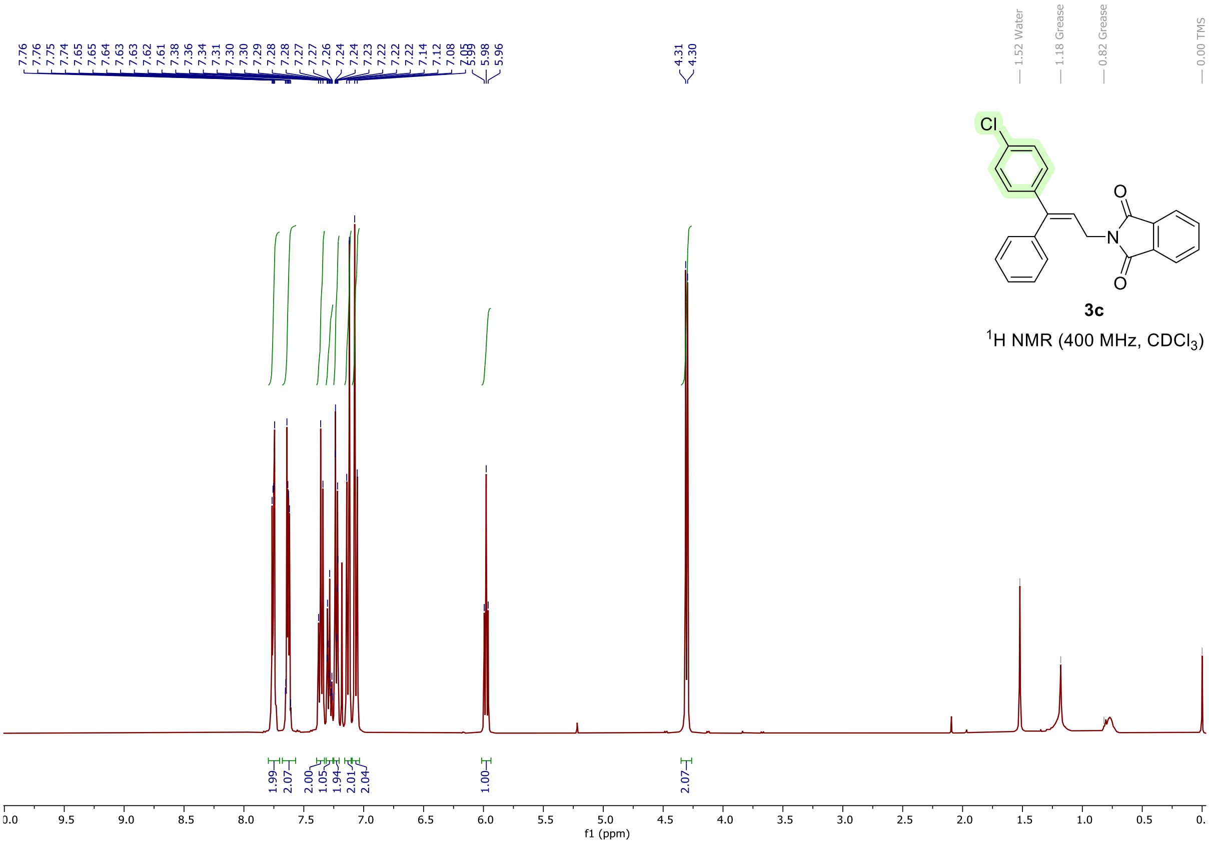

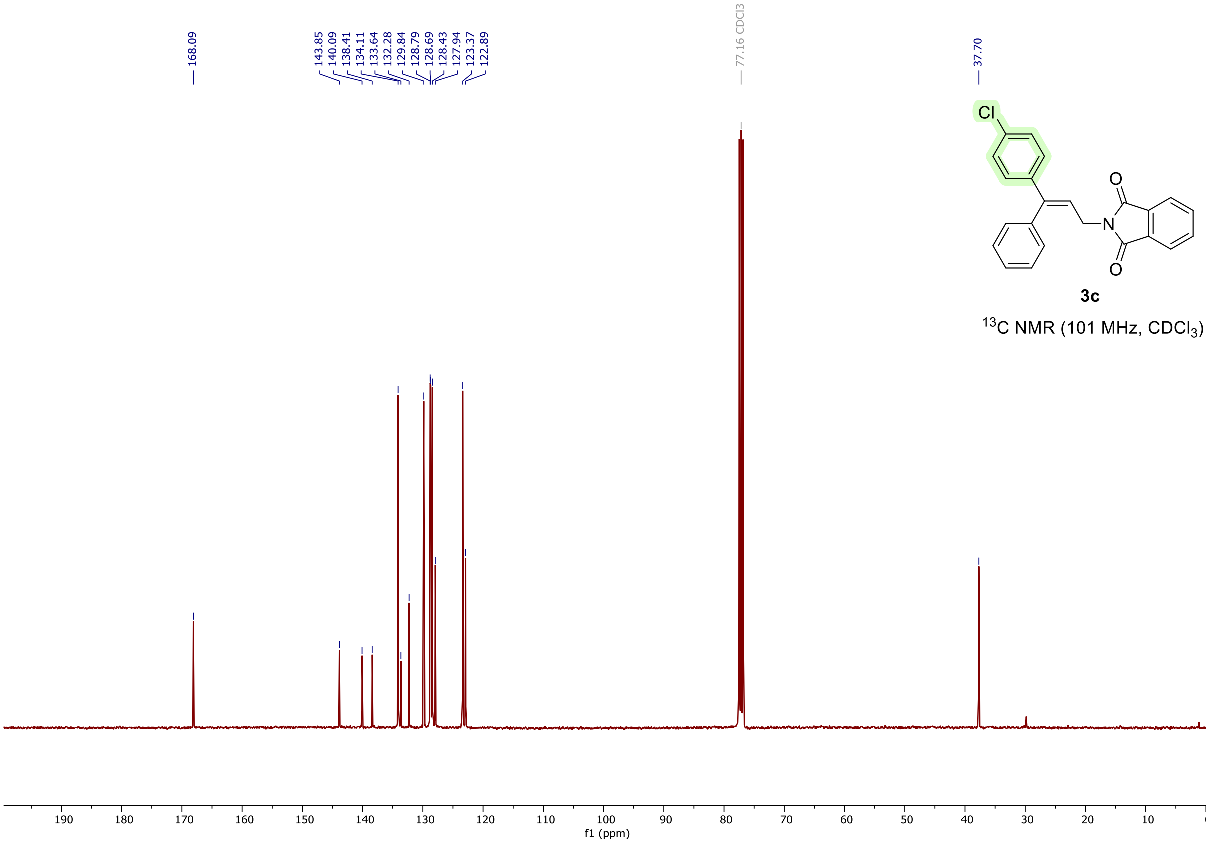

Spectra of 3d:

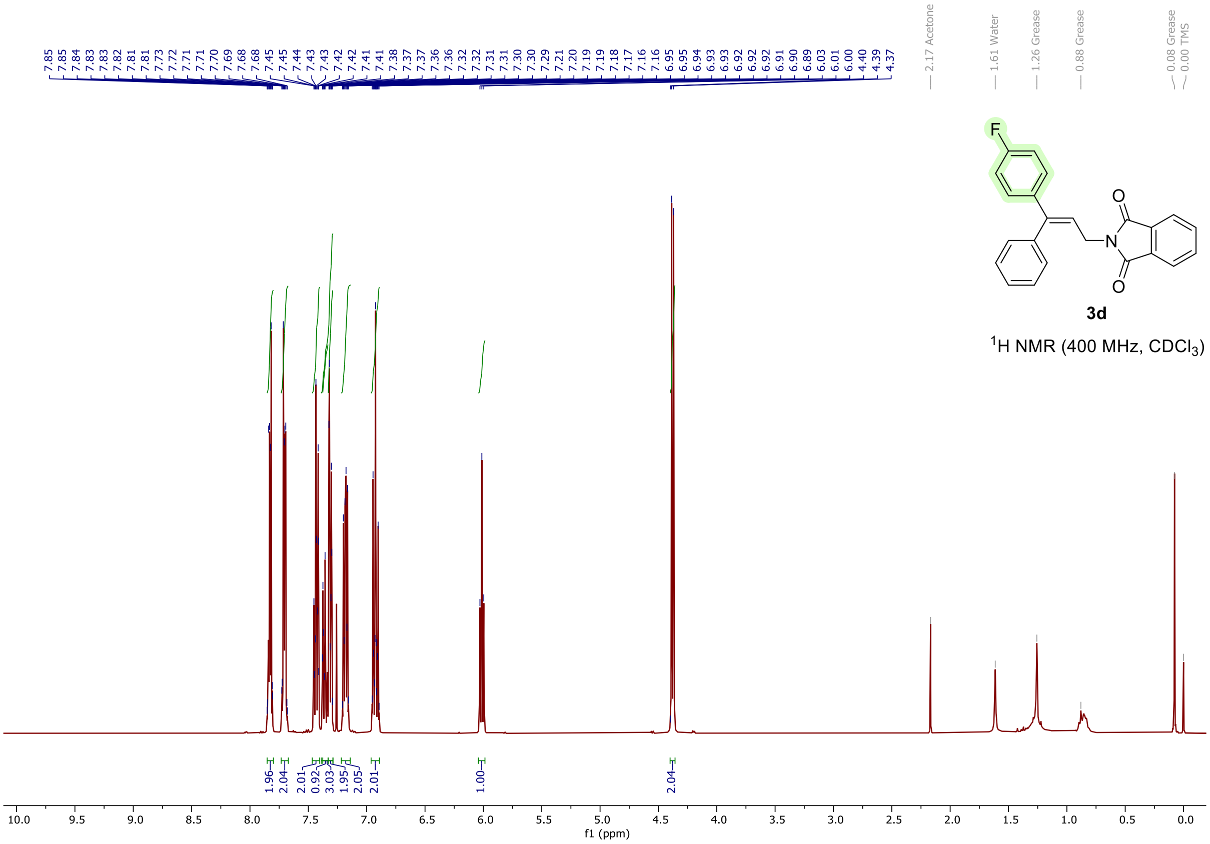

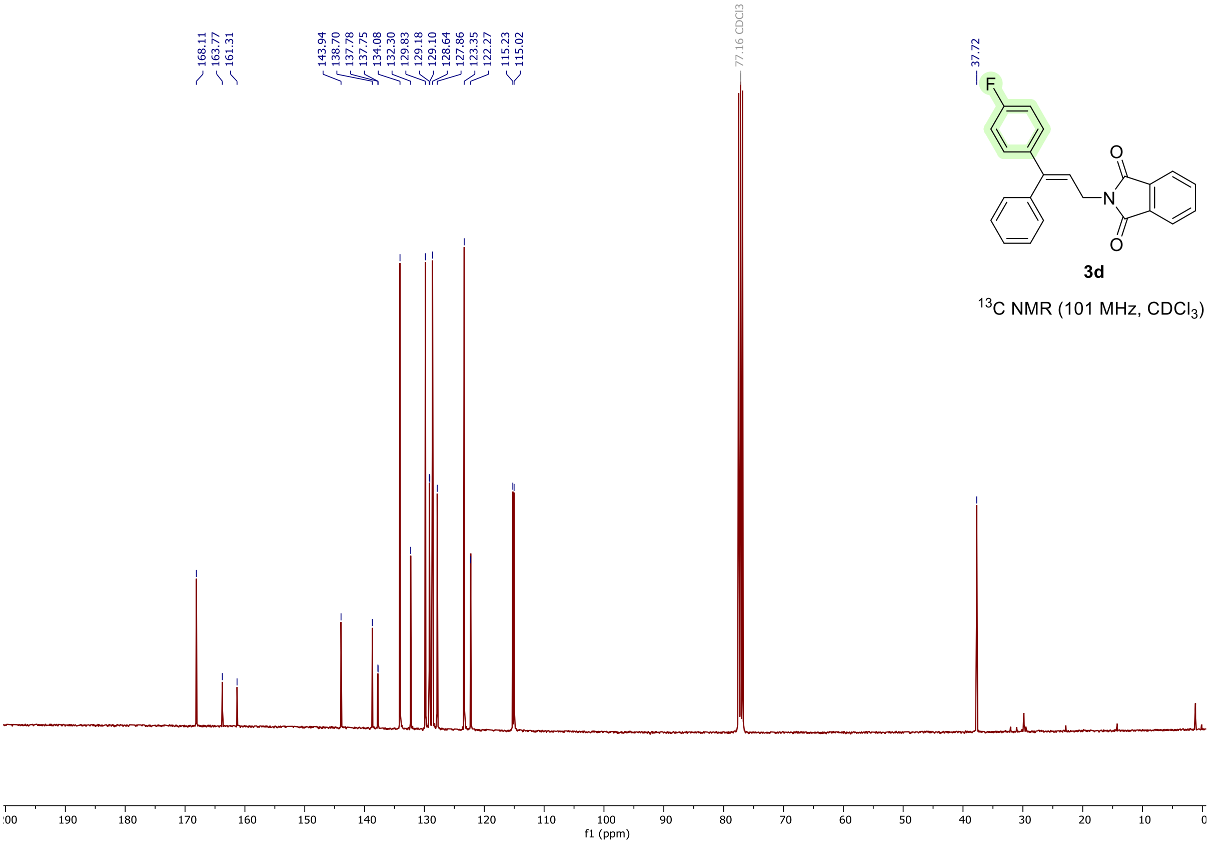

Spectra of 3e:

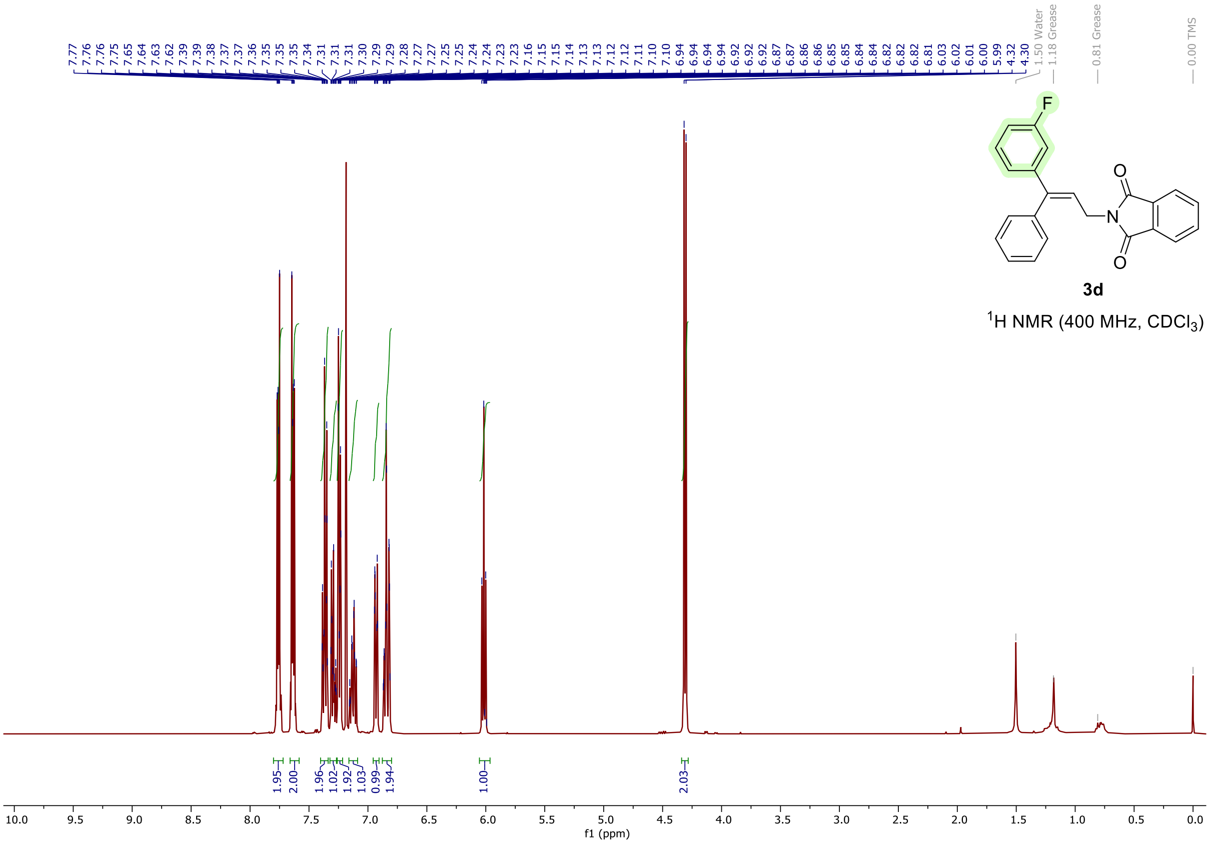

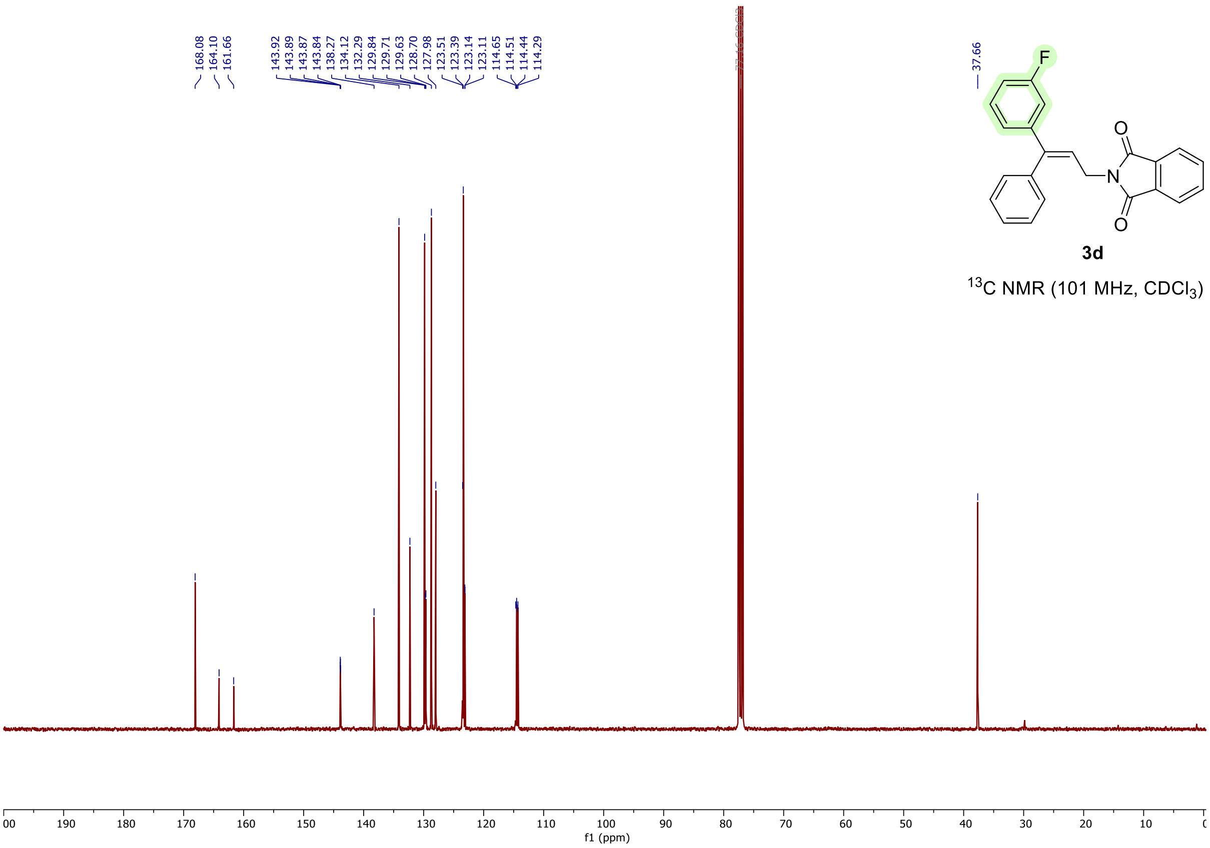

Spectra of 3f:

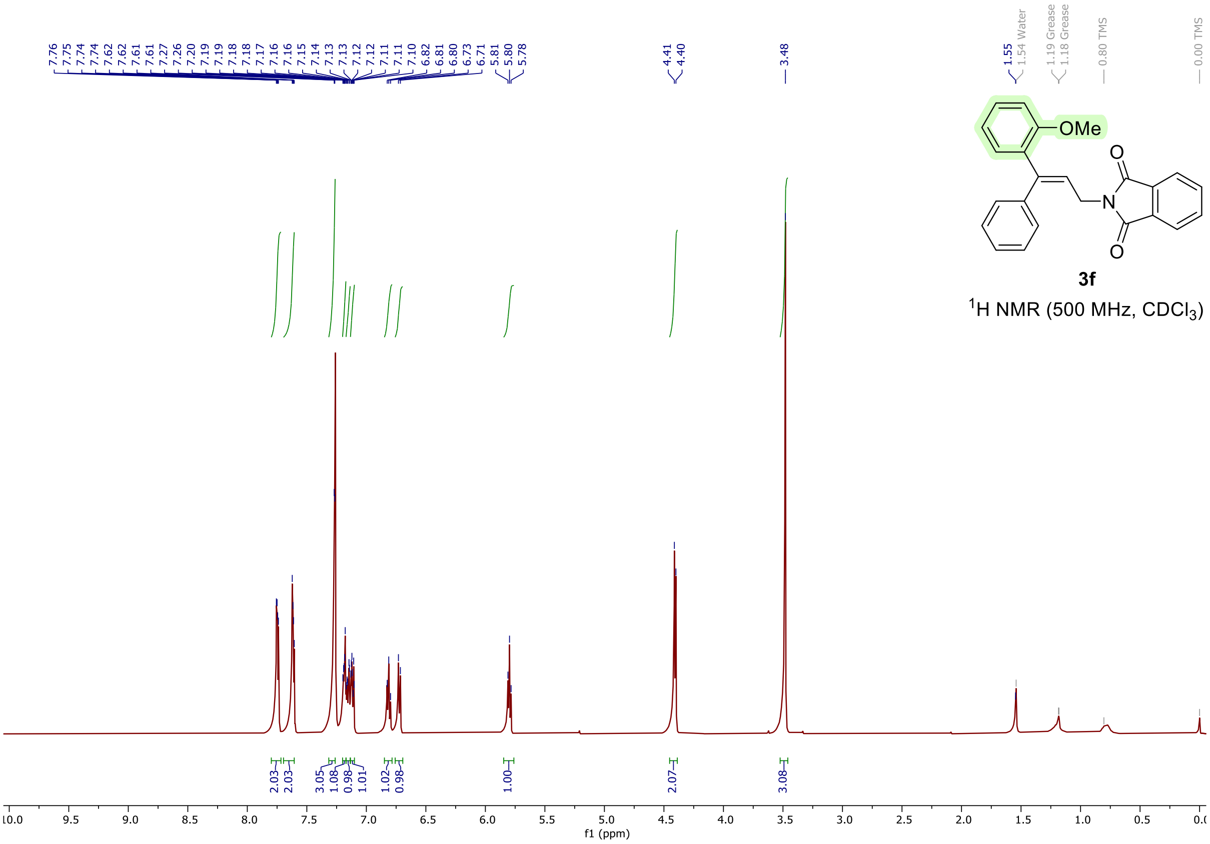

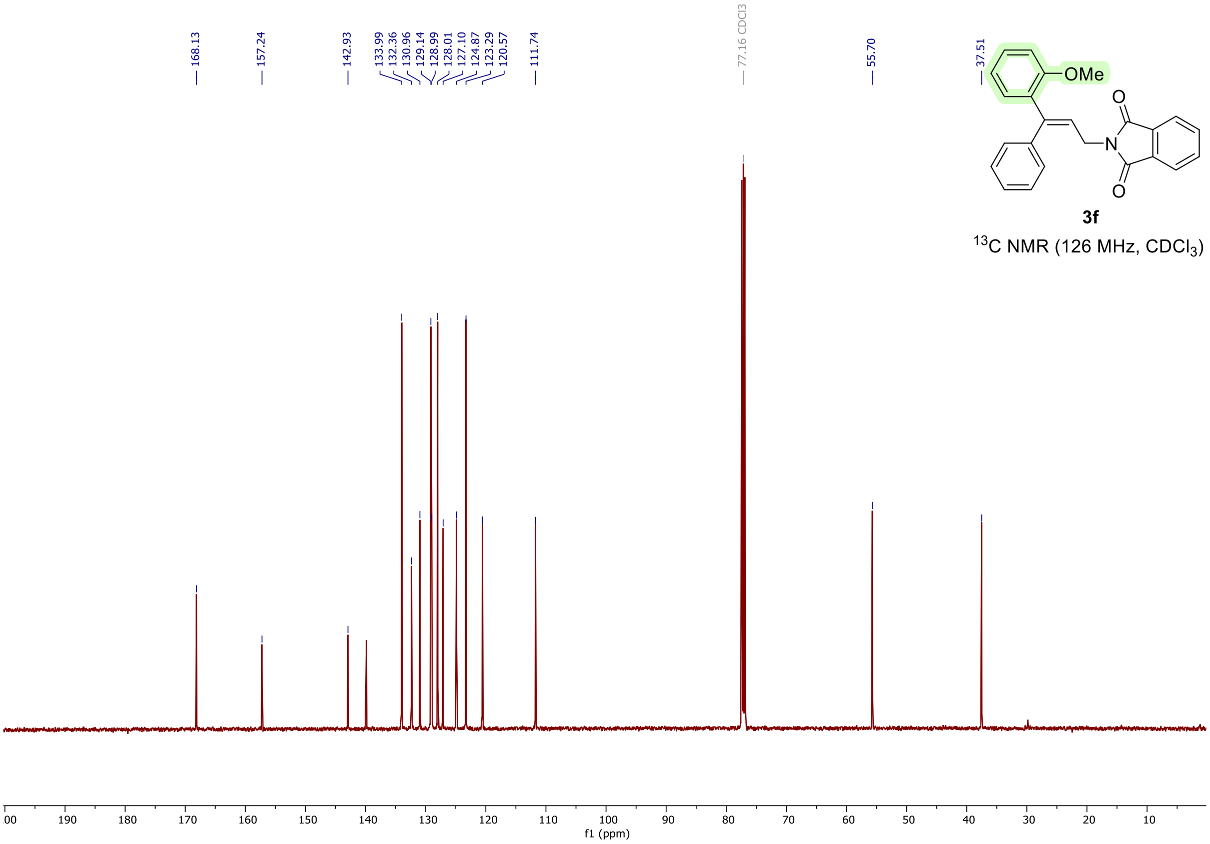

Spectra of 3g:

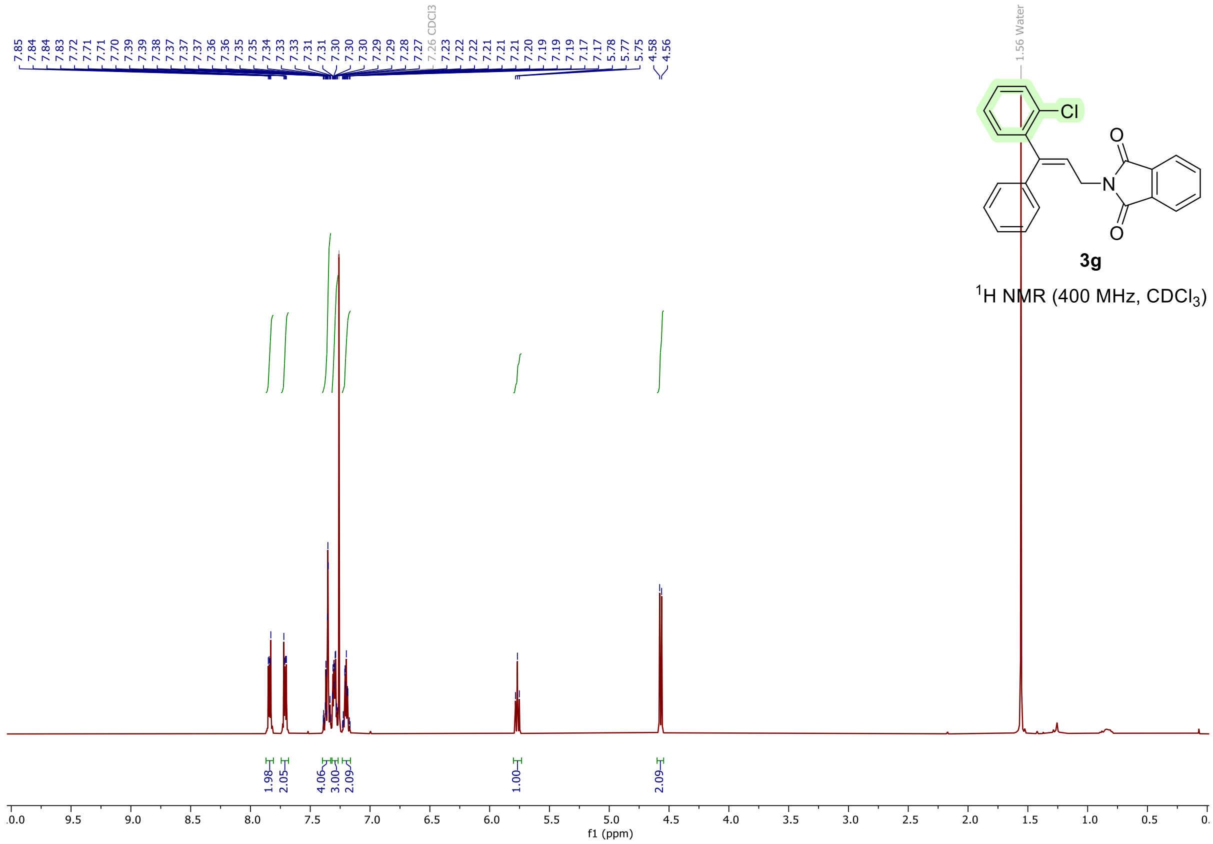

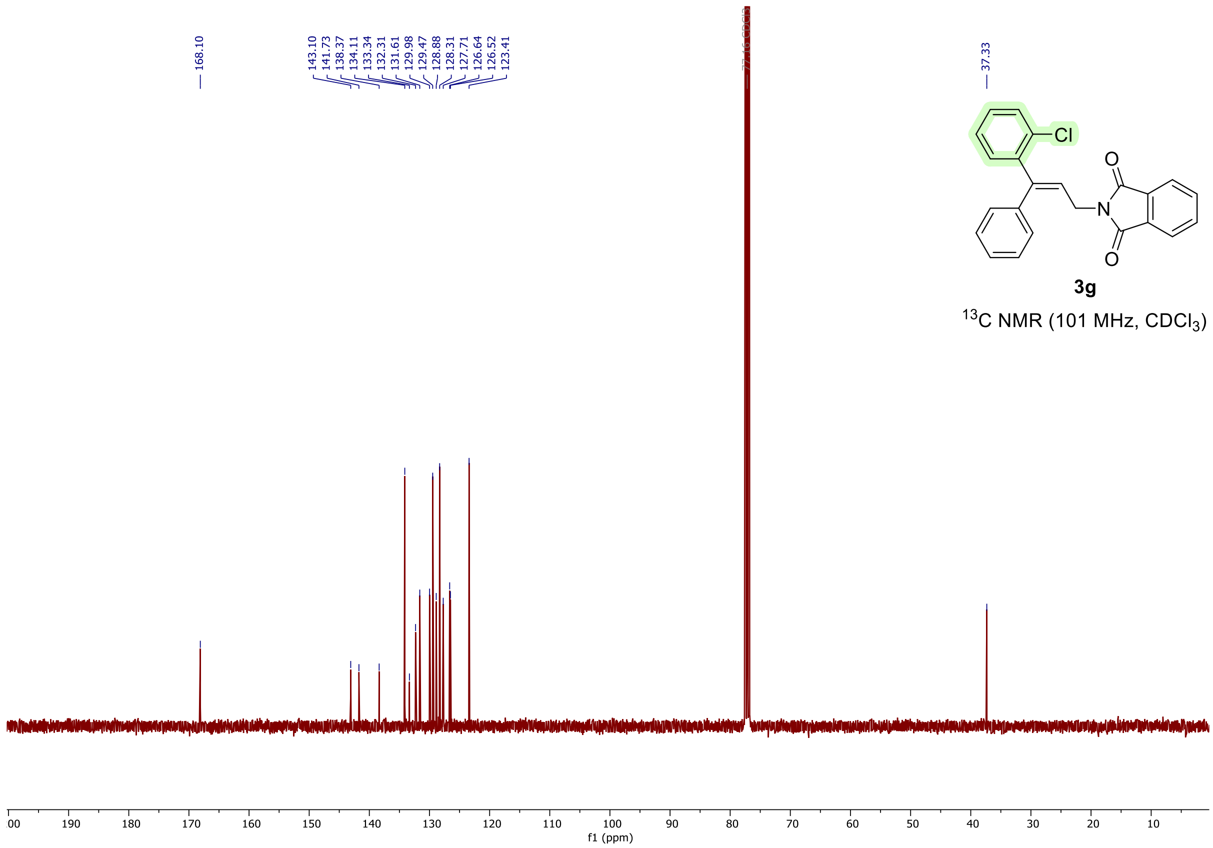

Spectra of 3h:

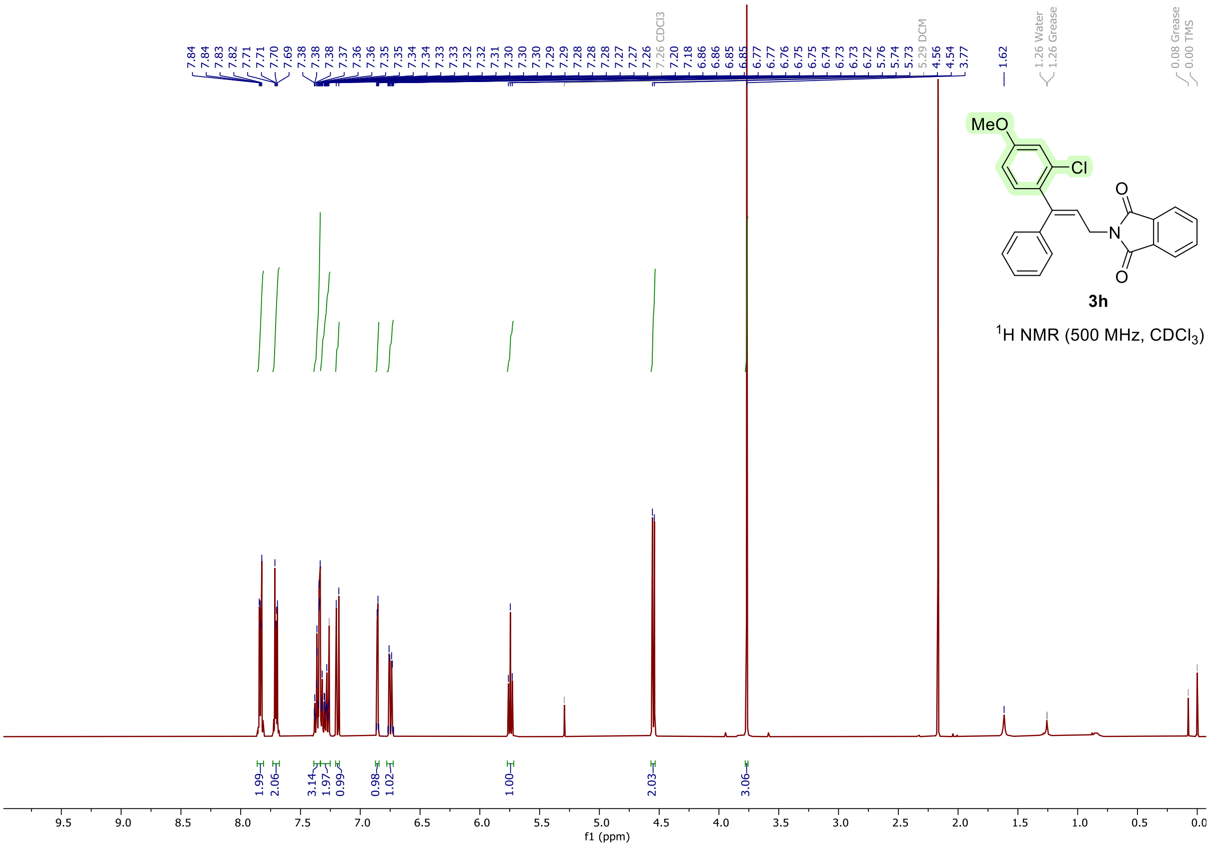

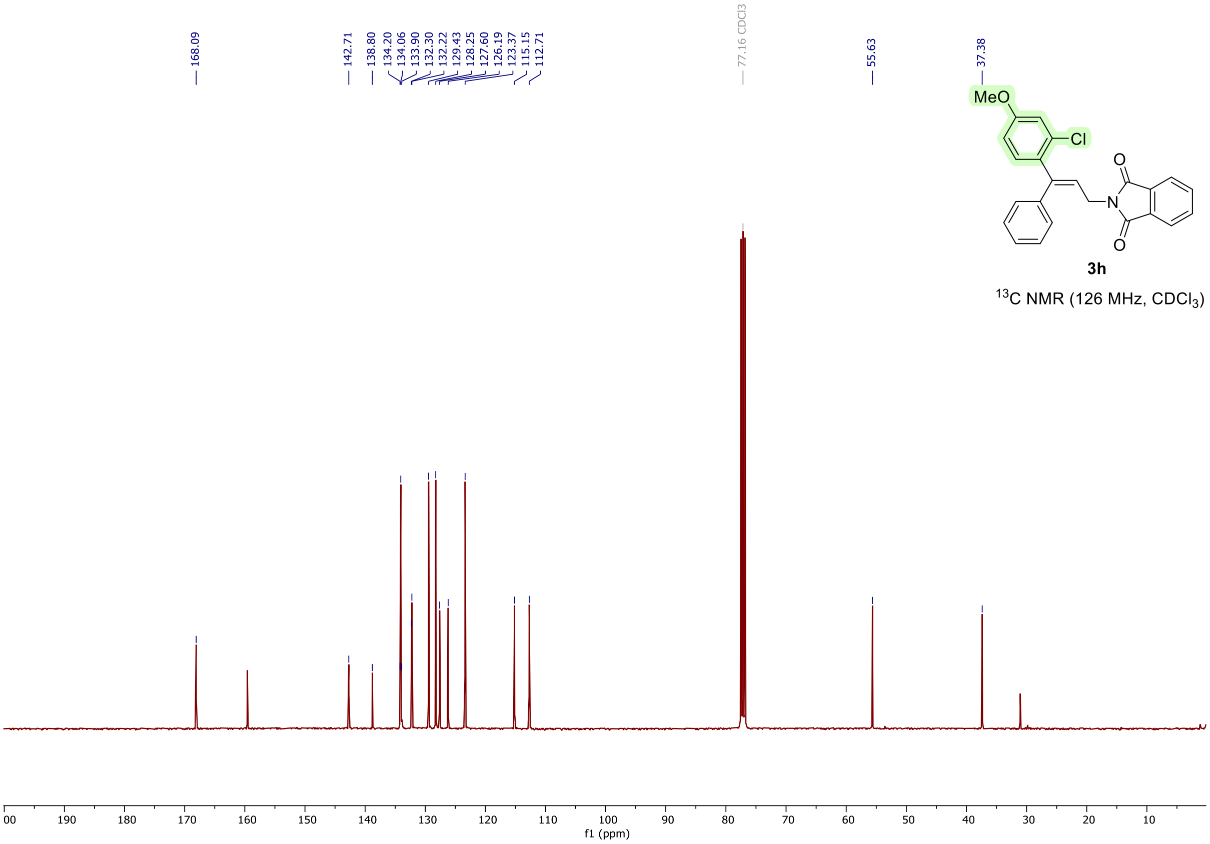

Spectra of 3i:

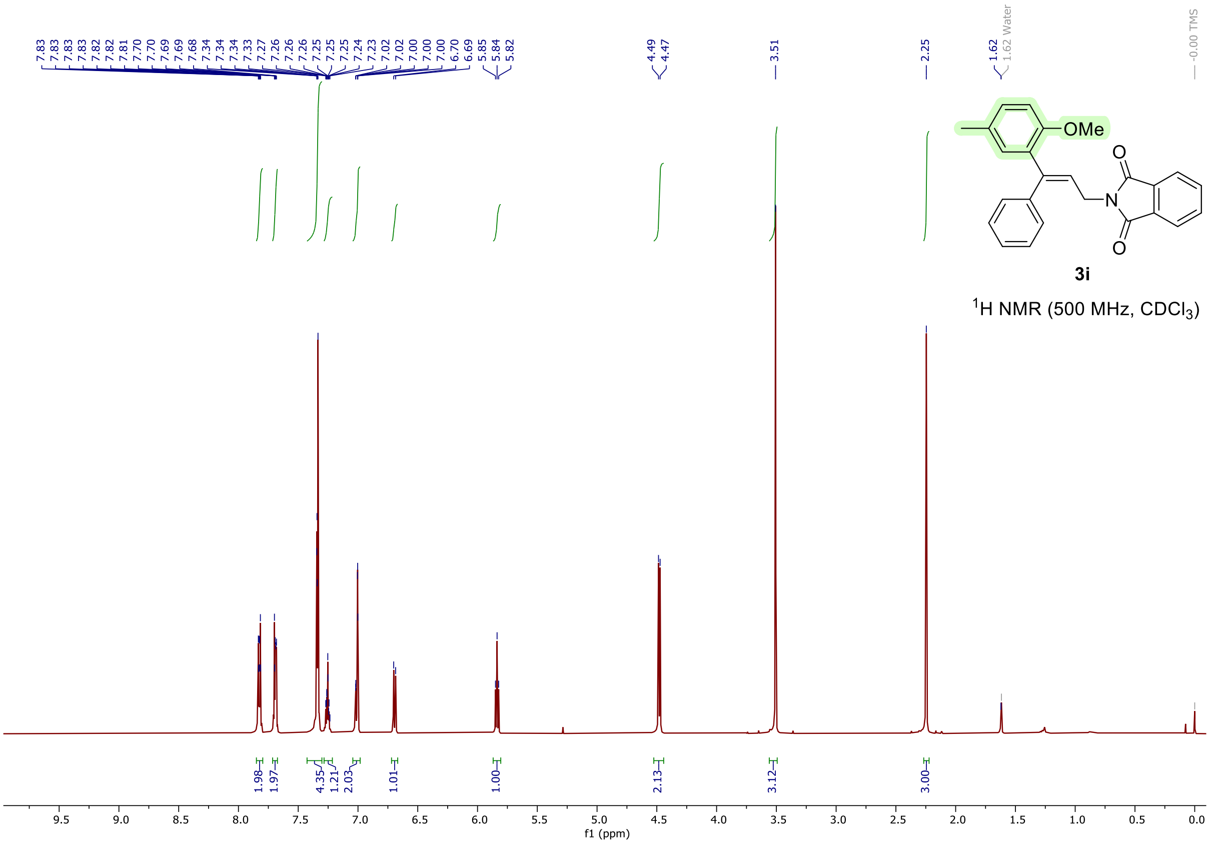

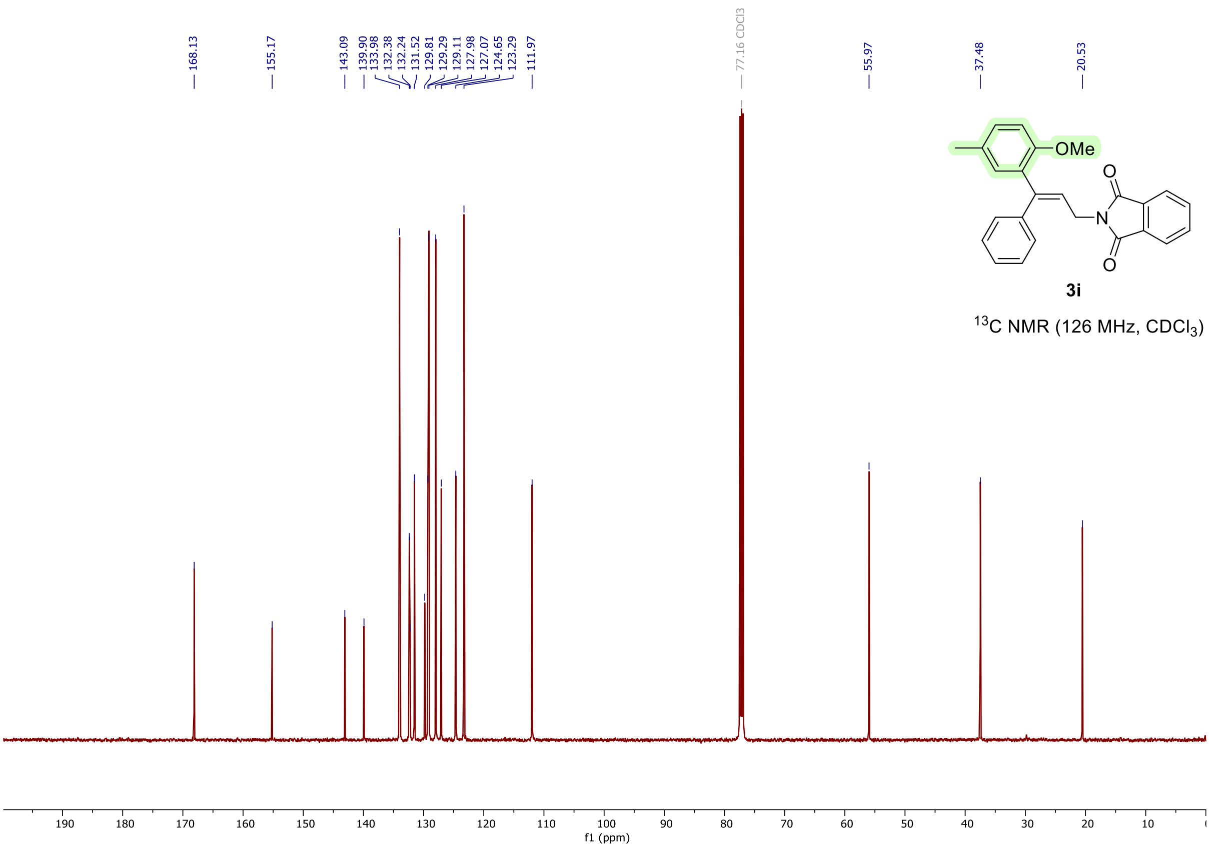

Spectra of 3j:

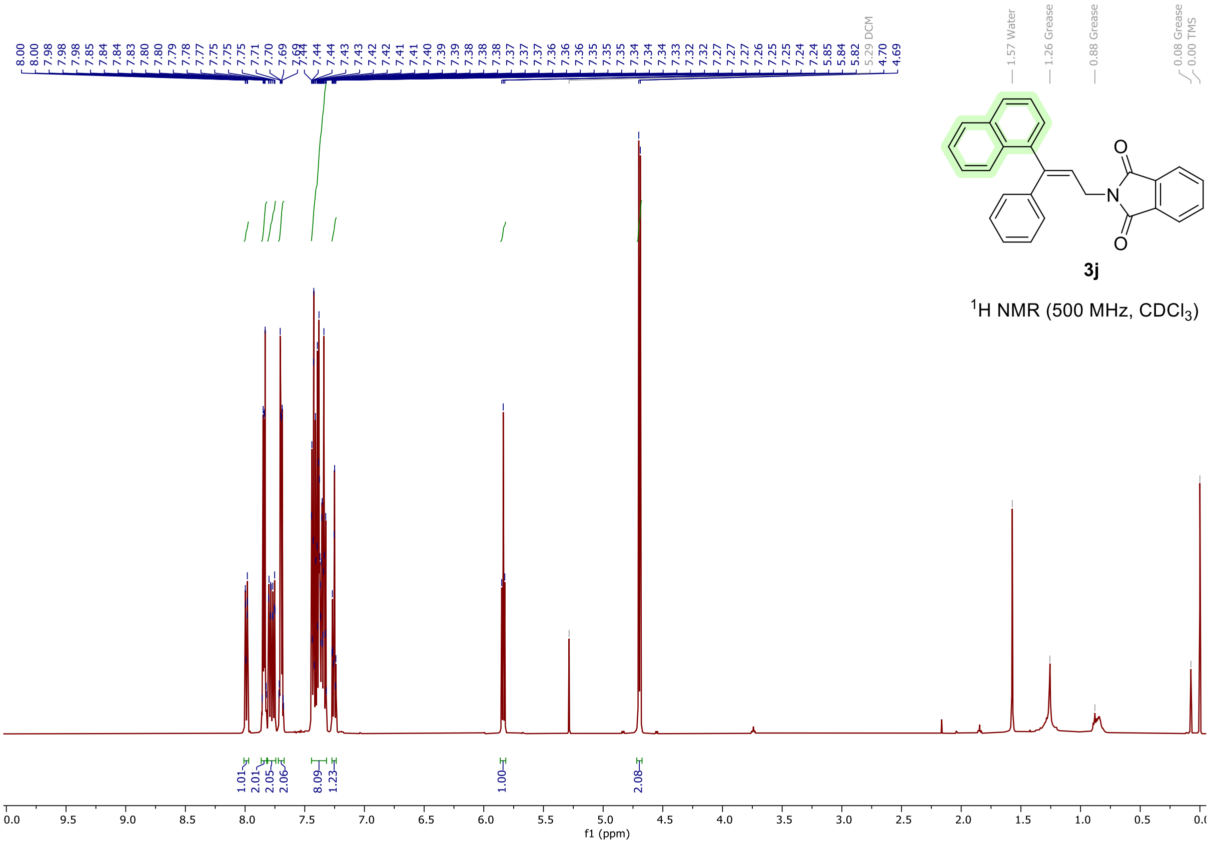

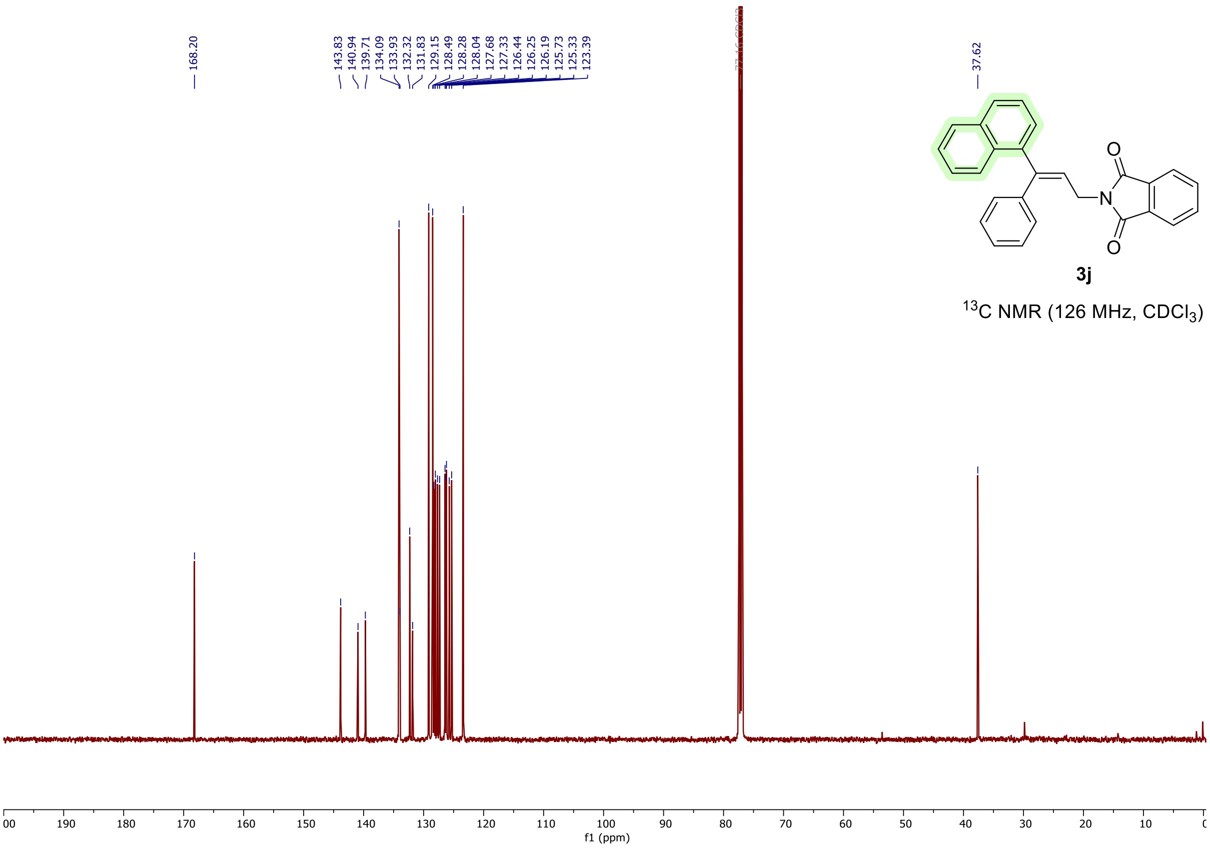

Spectra of 4b:

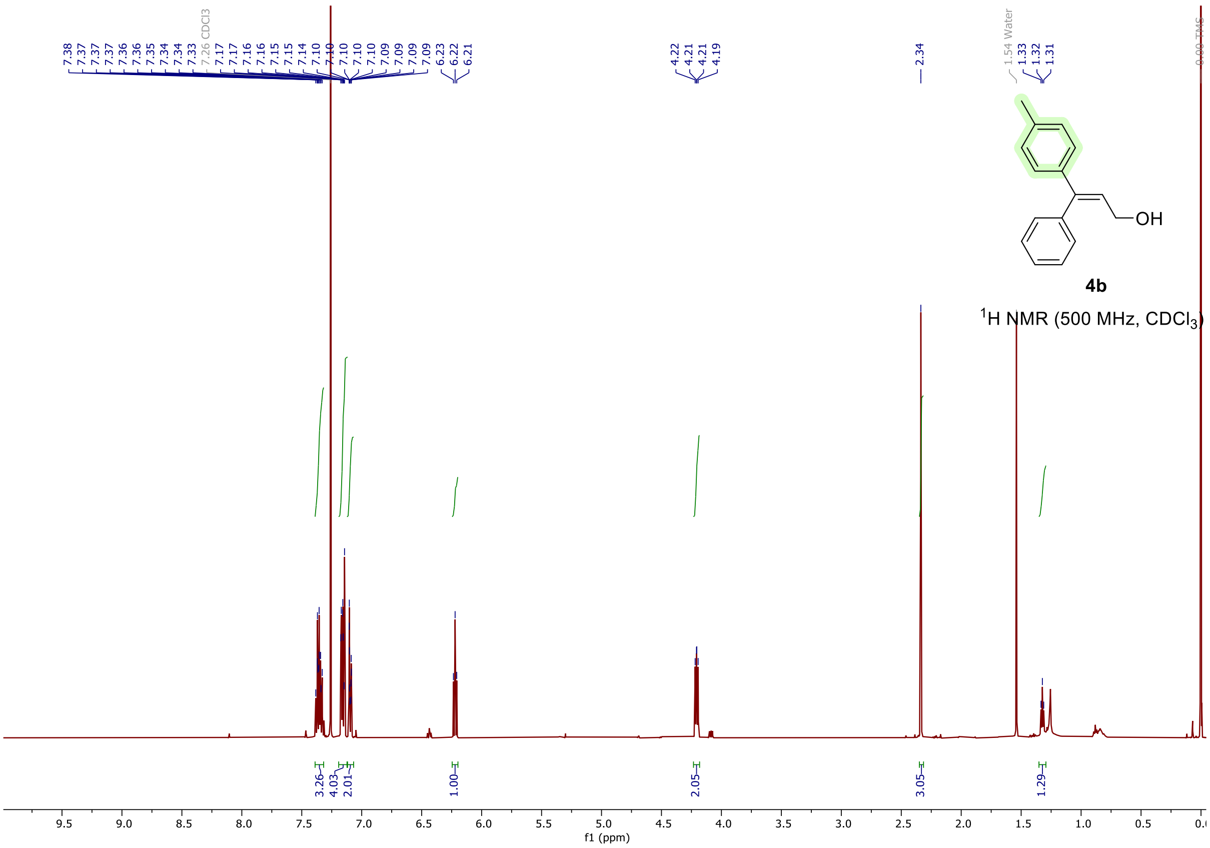

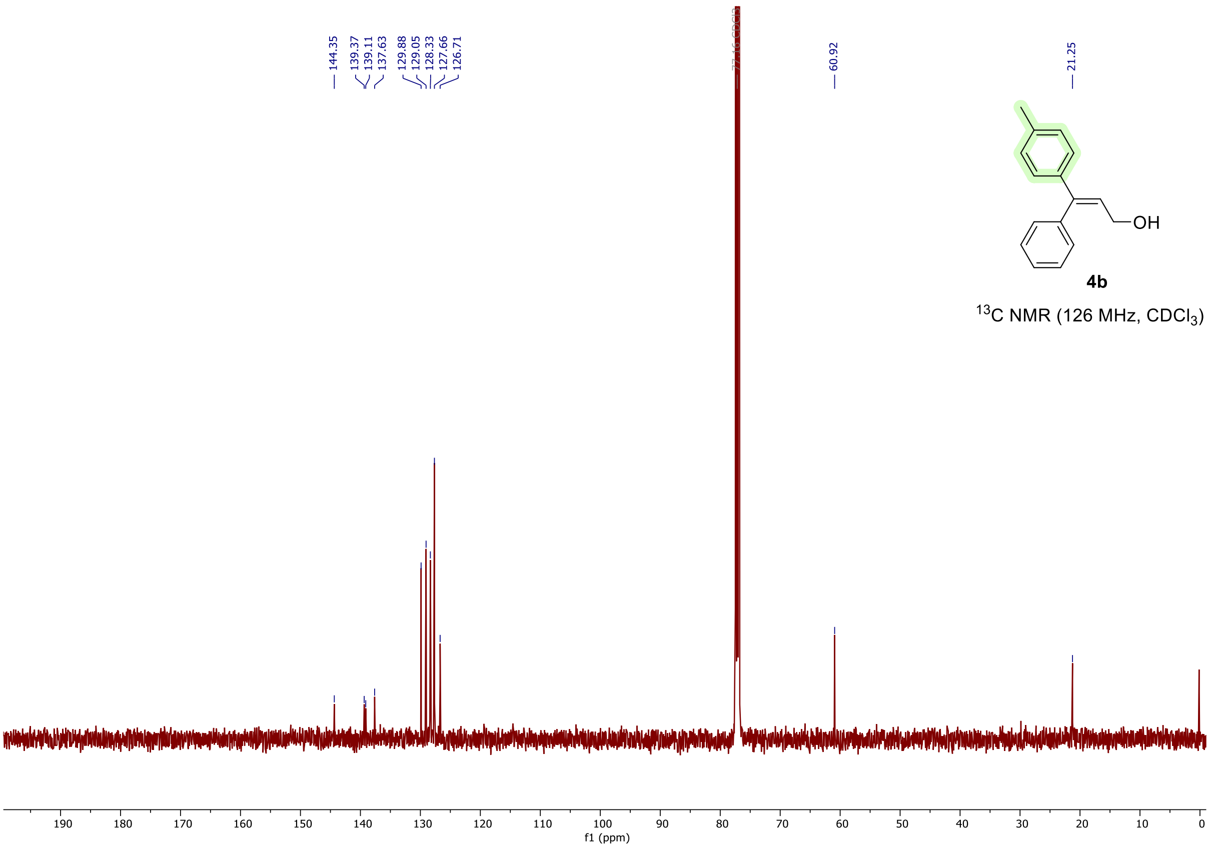

Spectra of 4c:

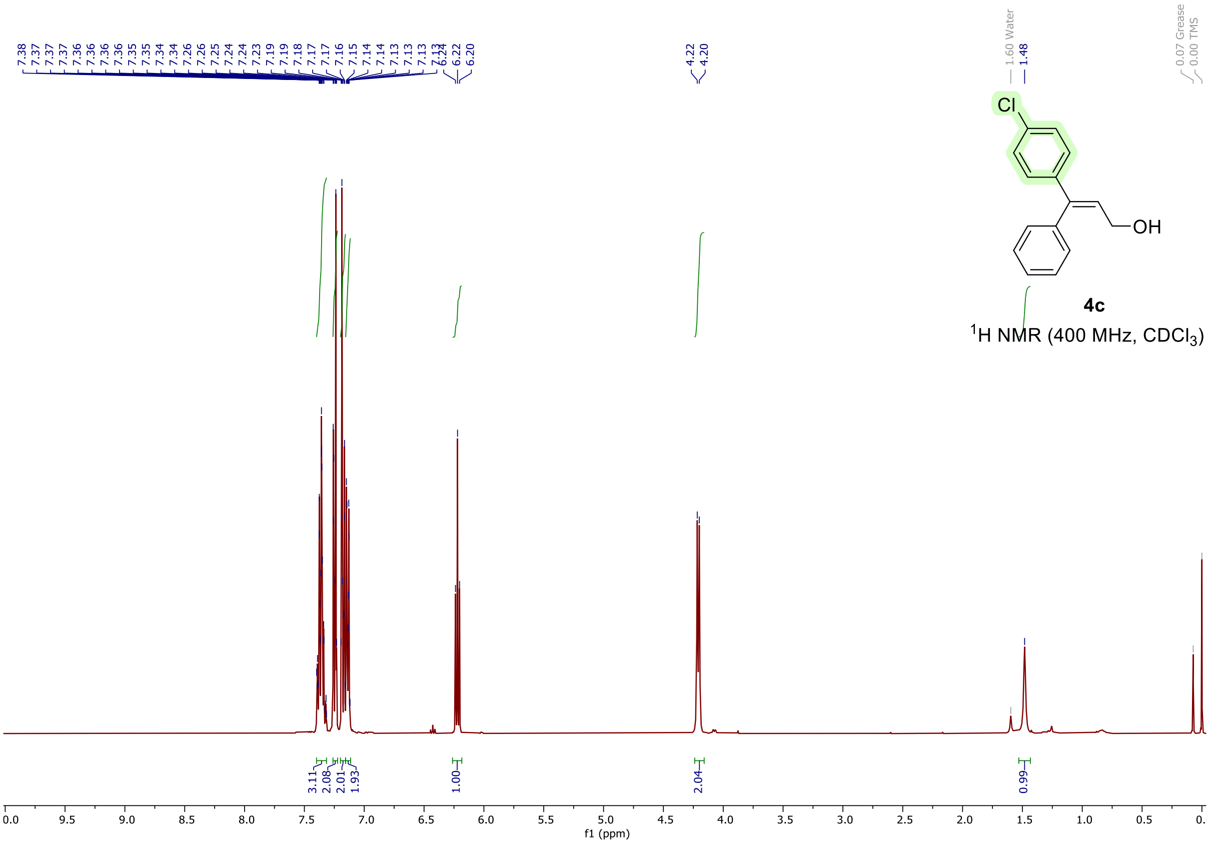

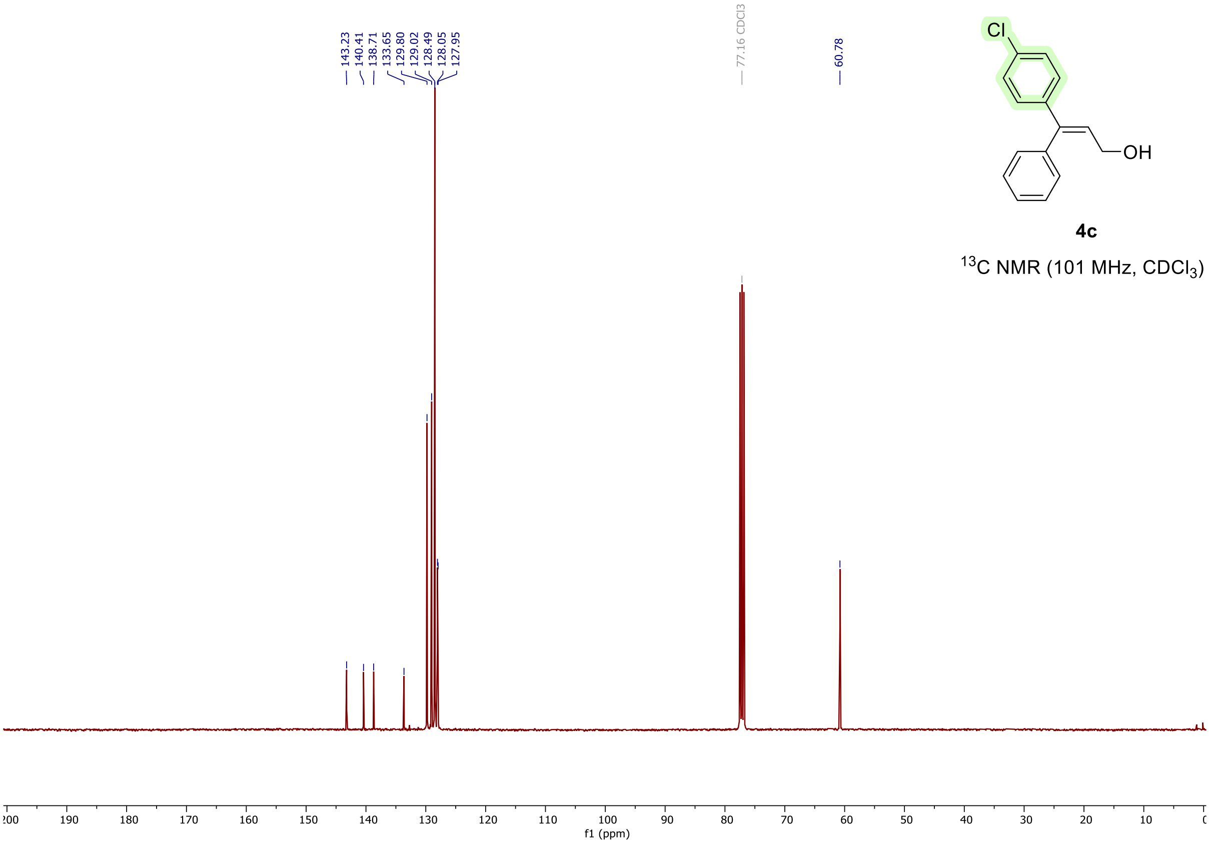

Spectra of 4e:

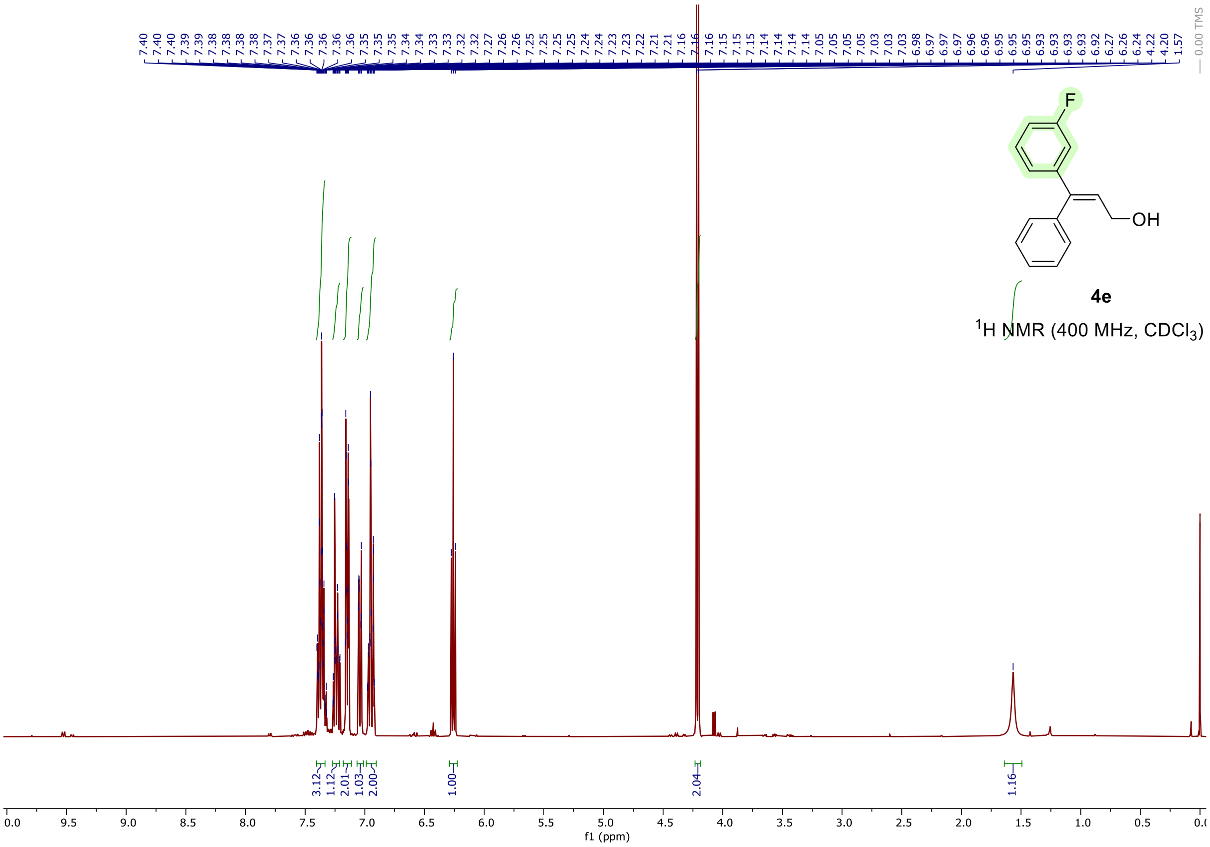

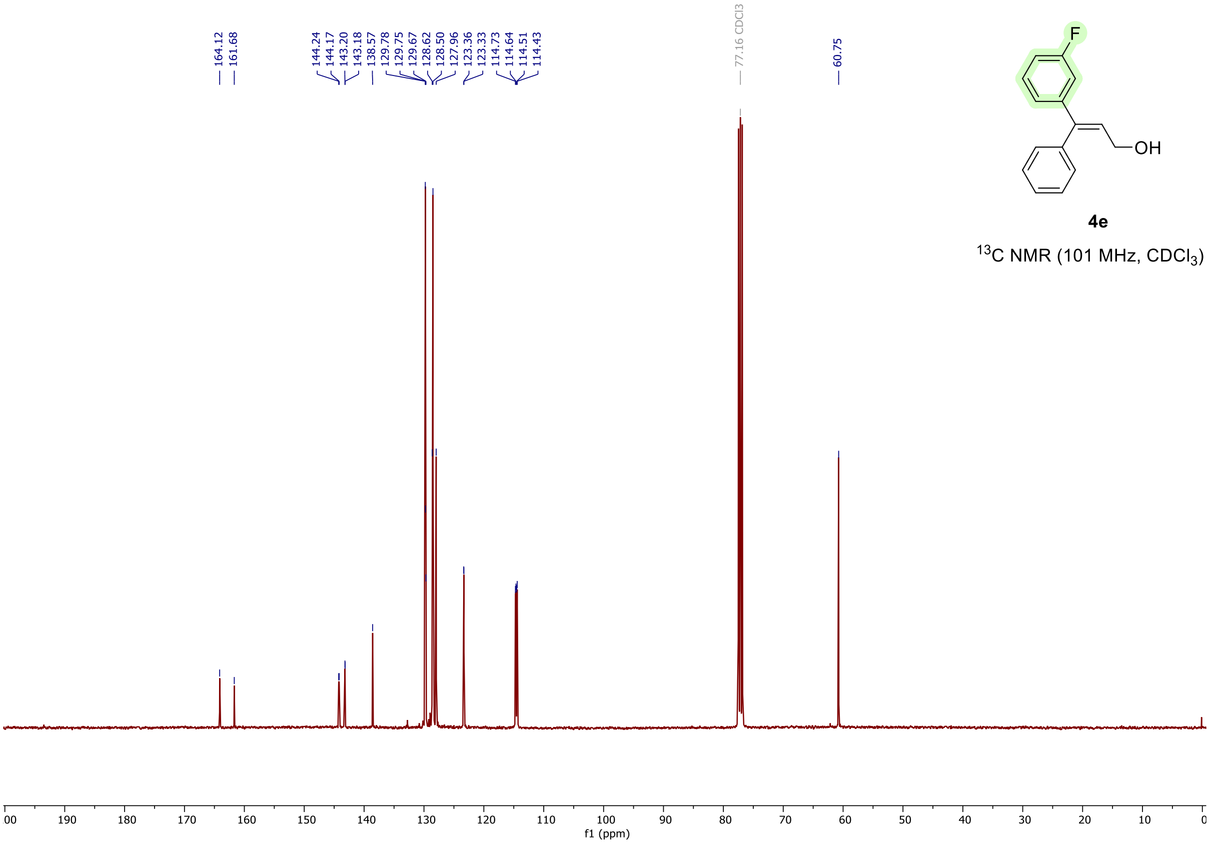

Spectra of 4f:

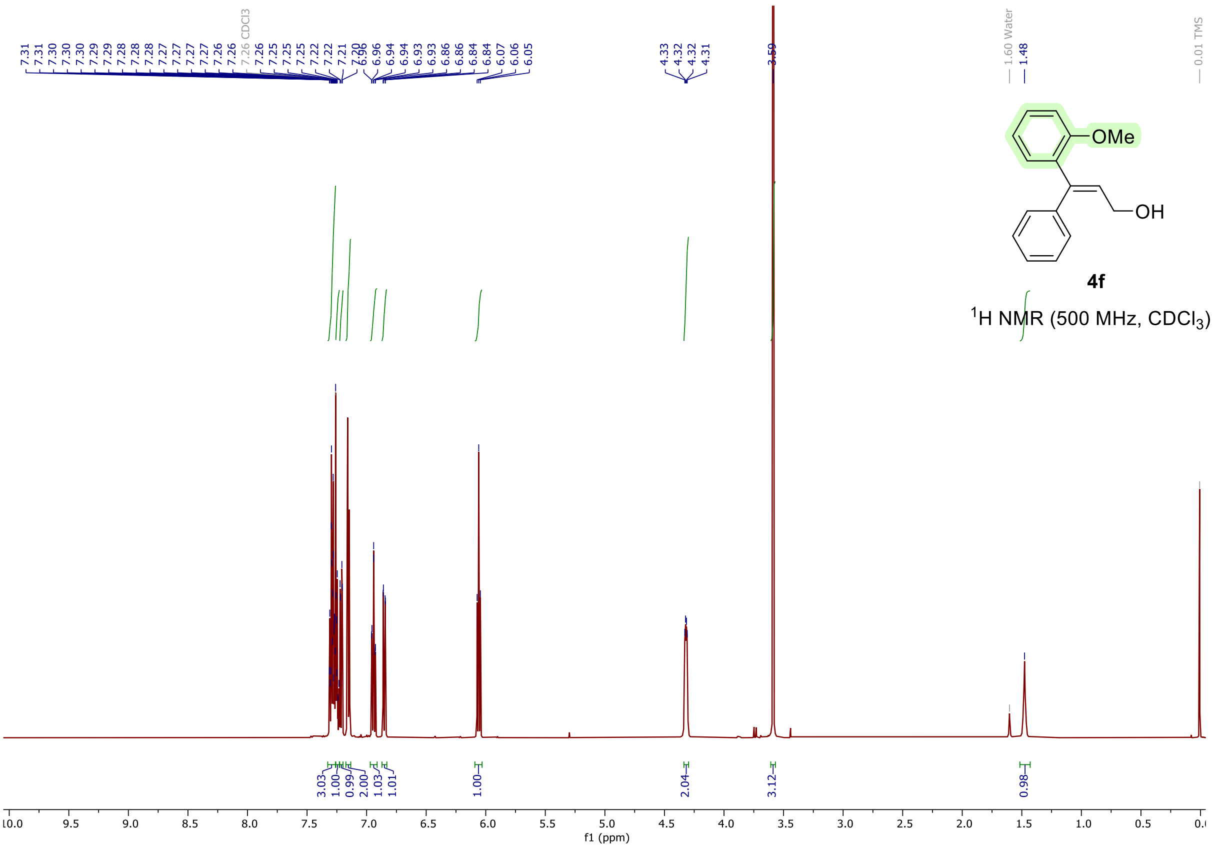

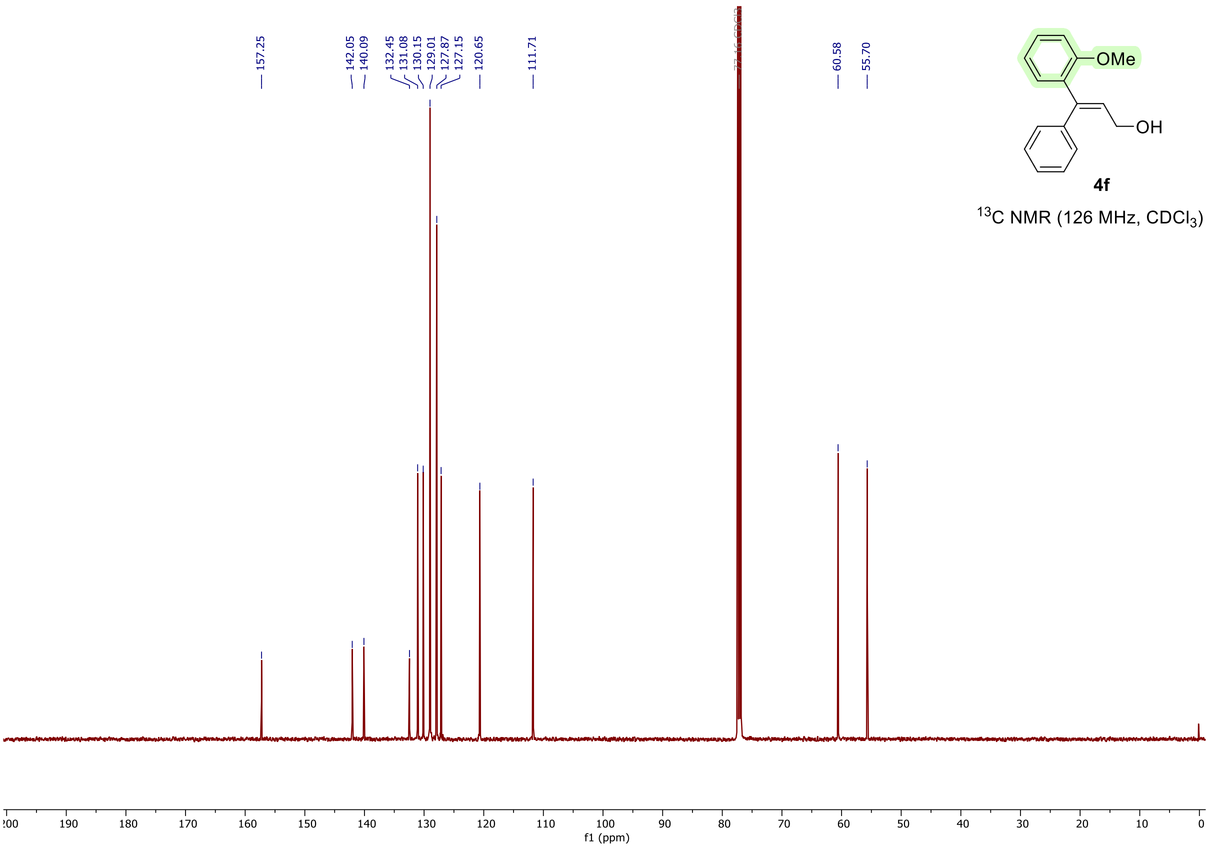

Spectra of 4g:

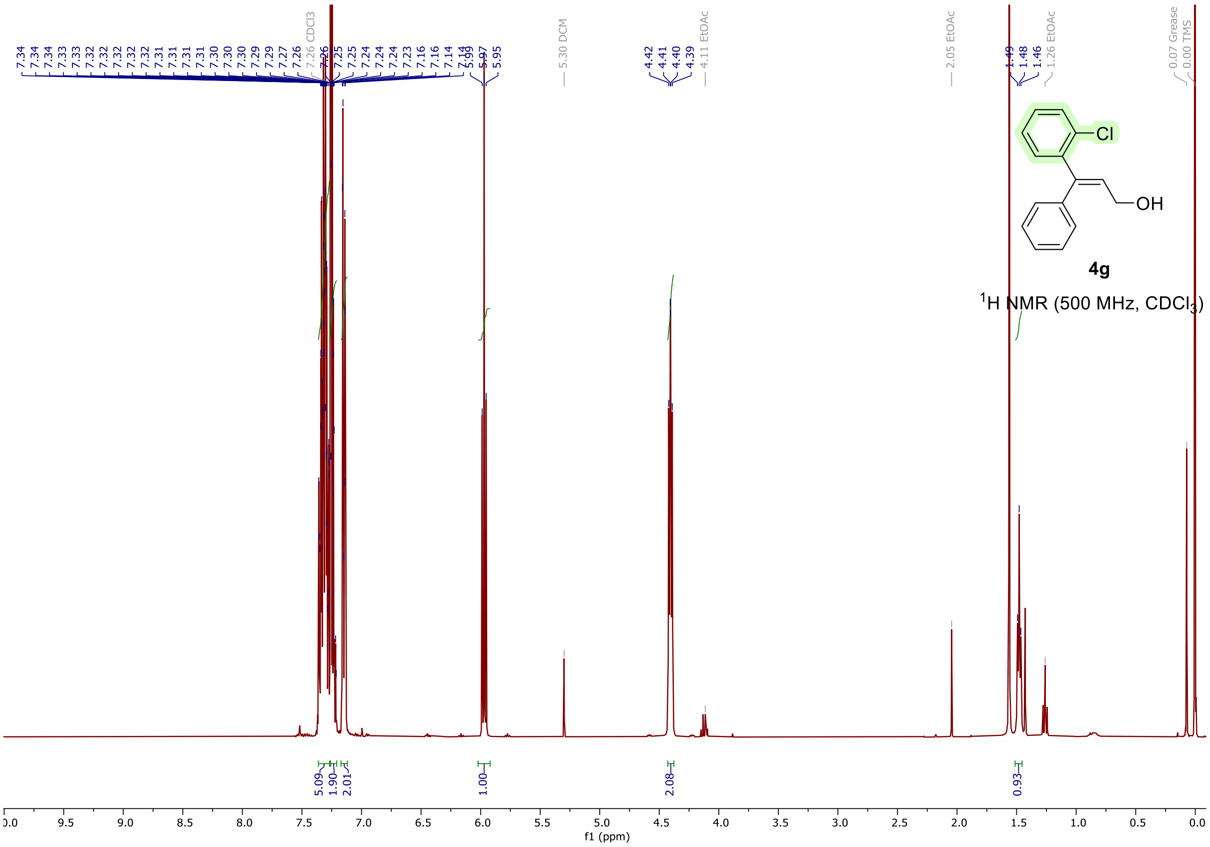

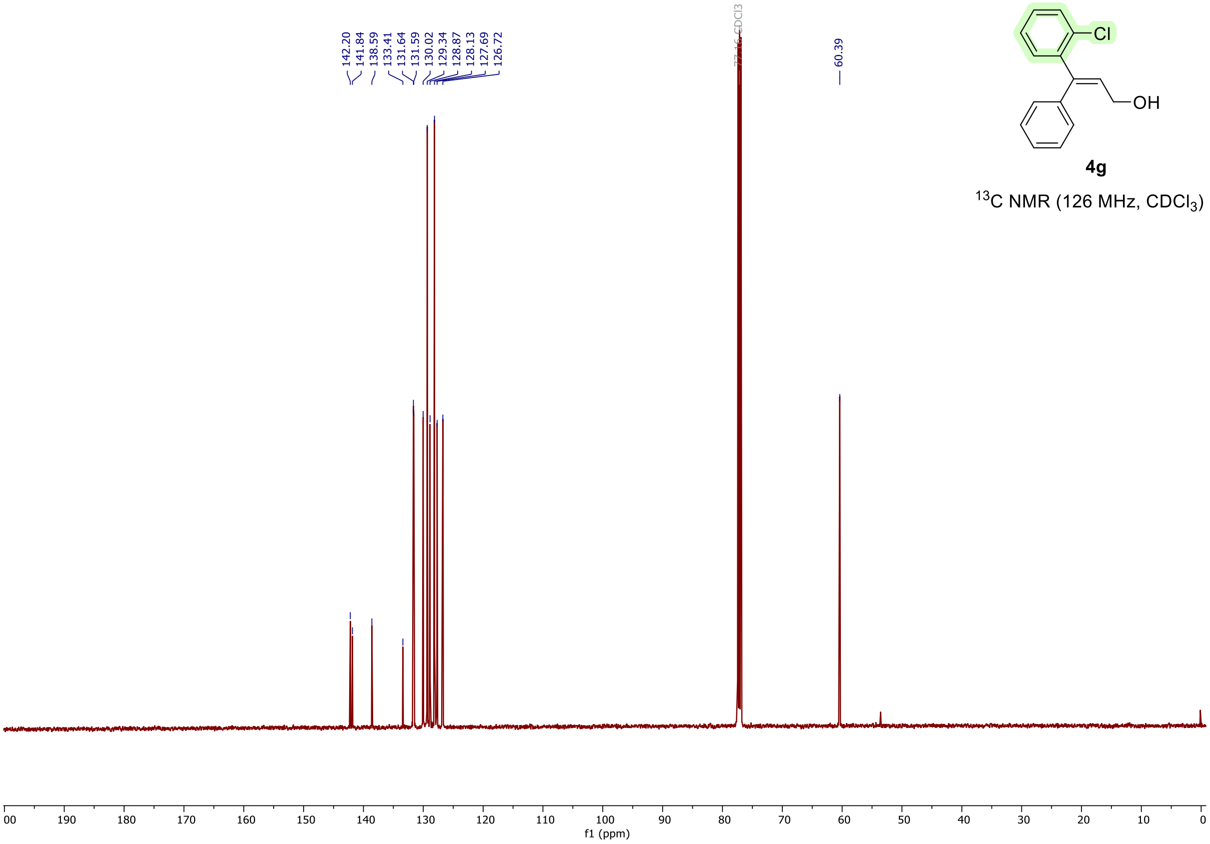

Spectra of 4h:

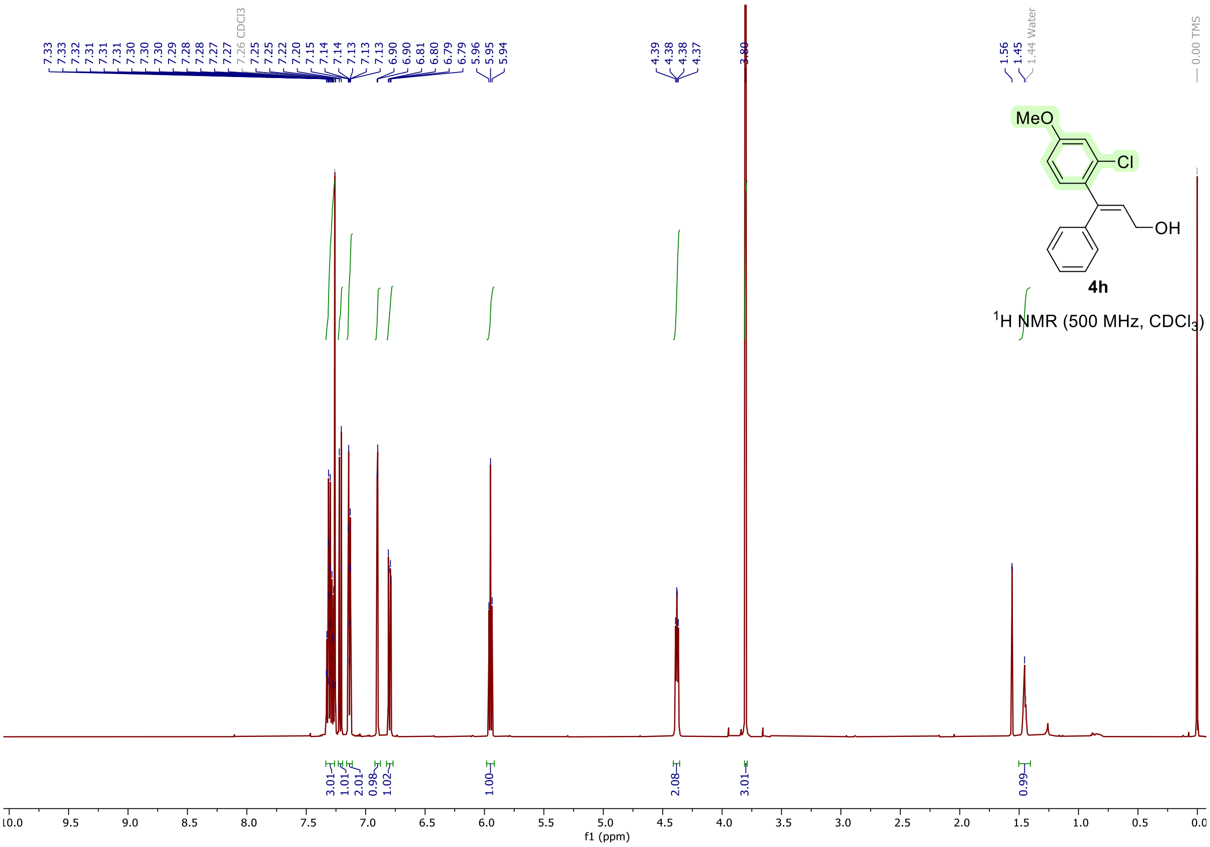

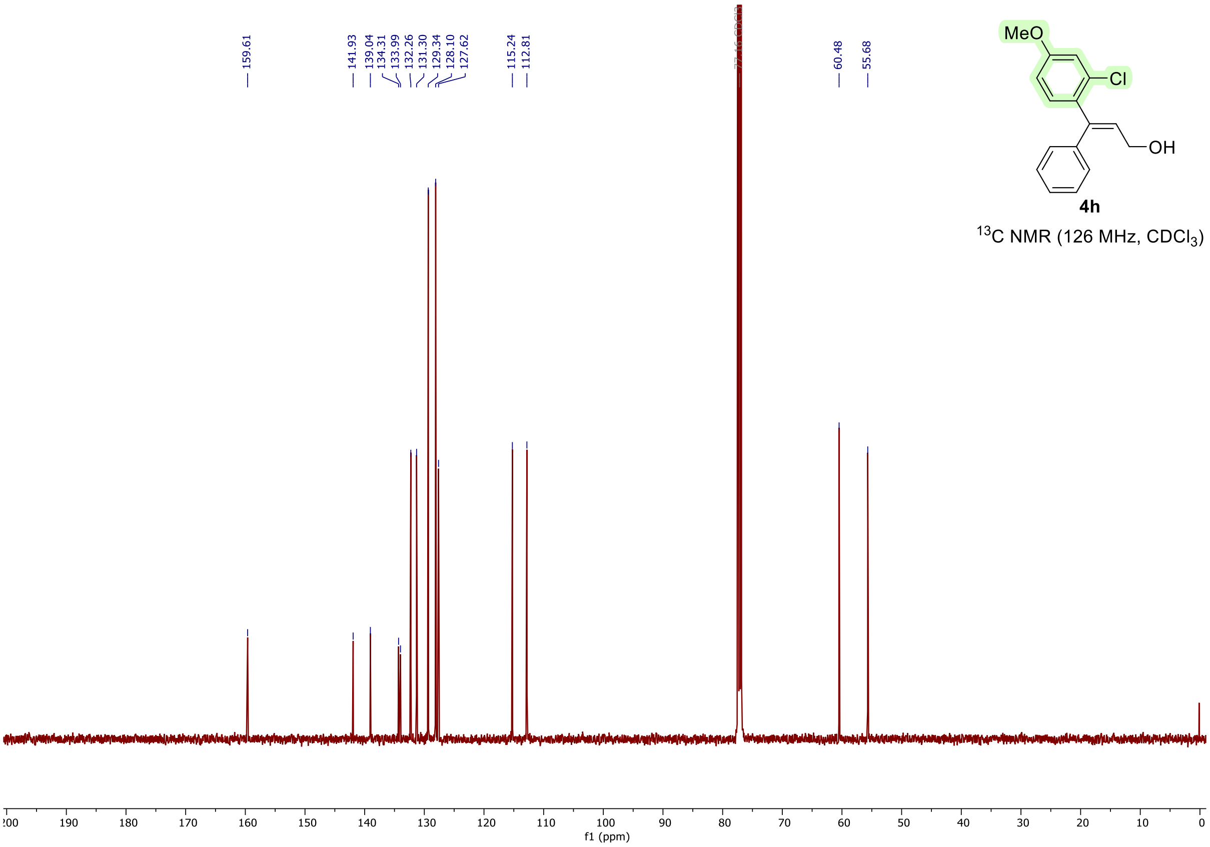

Spectra of 4i:

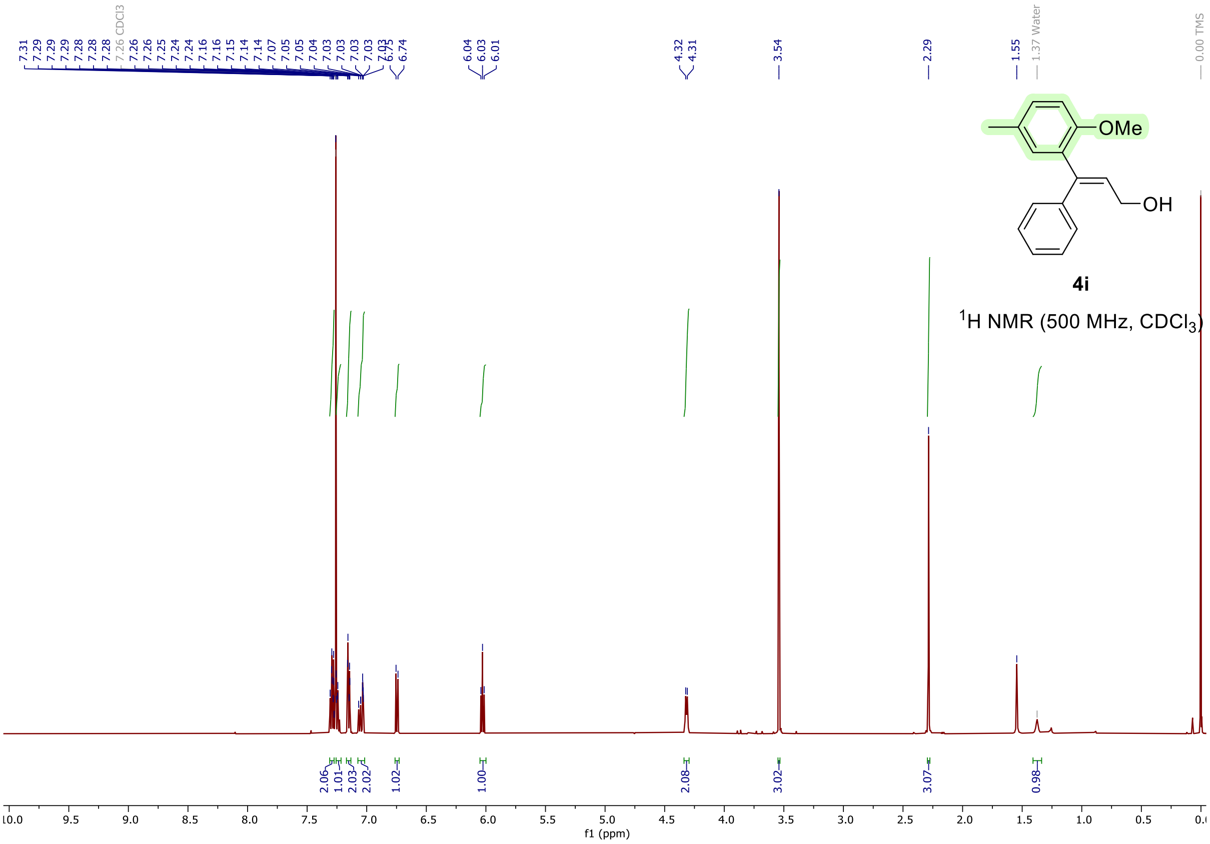

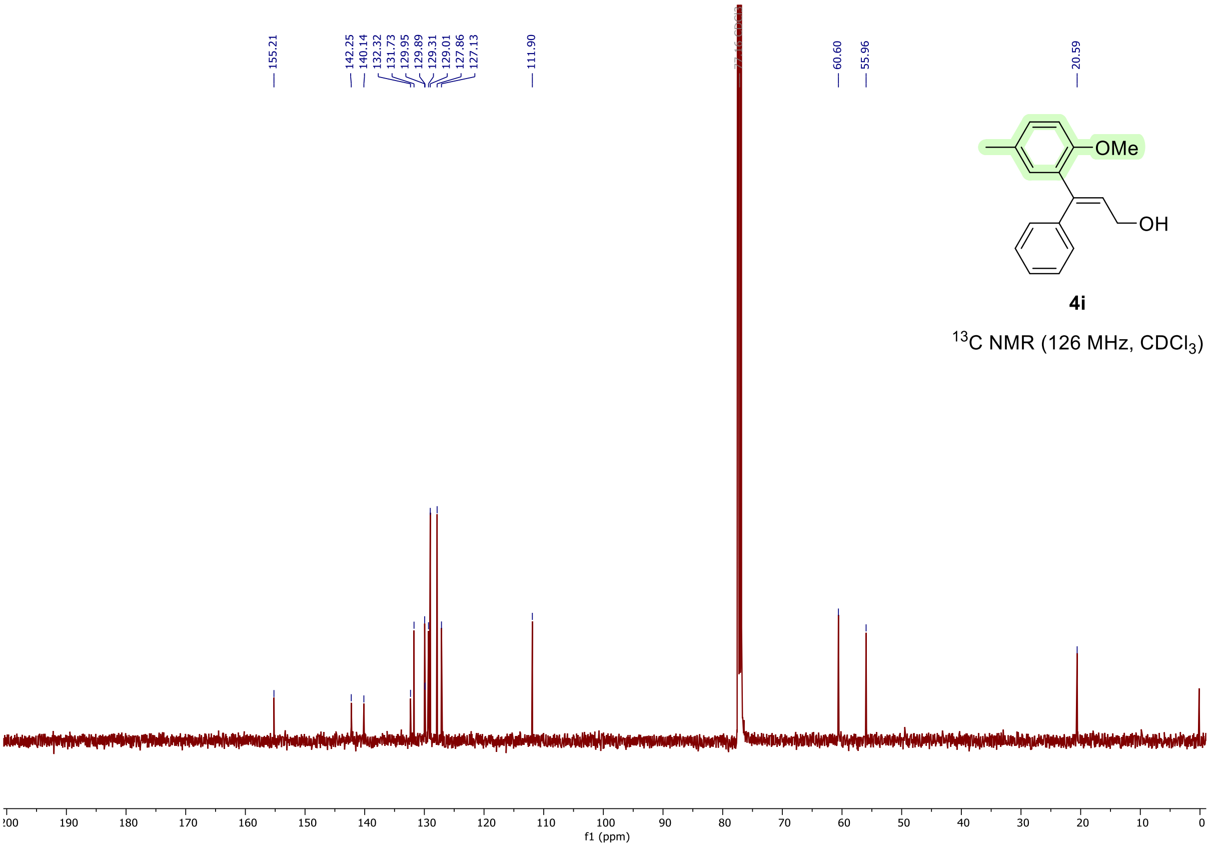

Spectra of 4j:

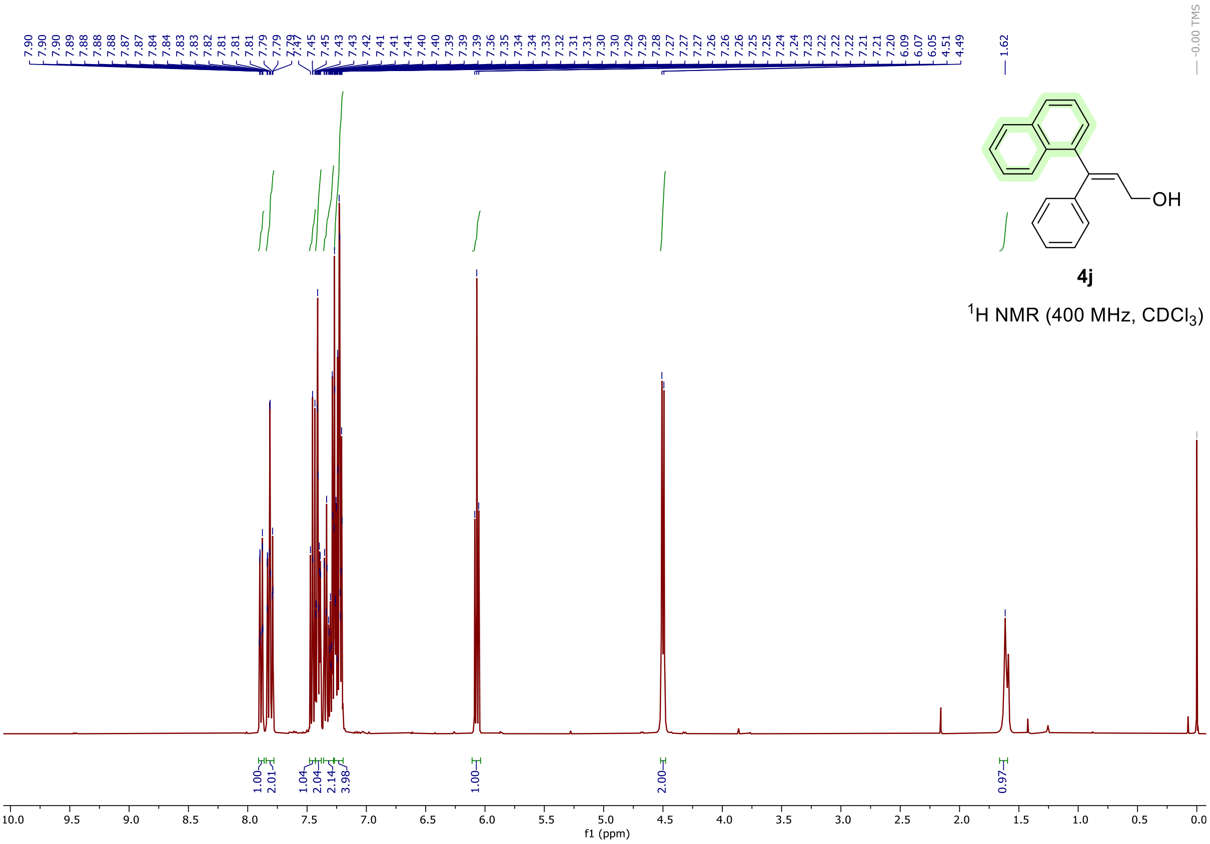

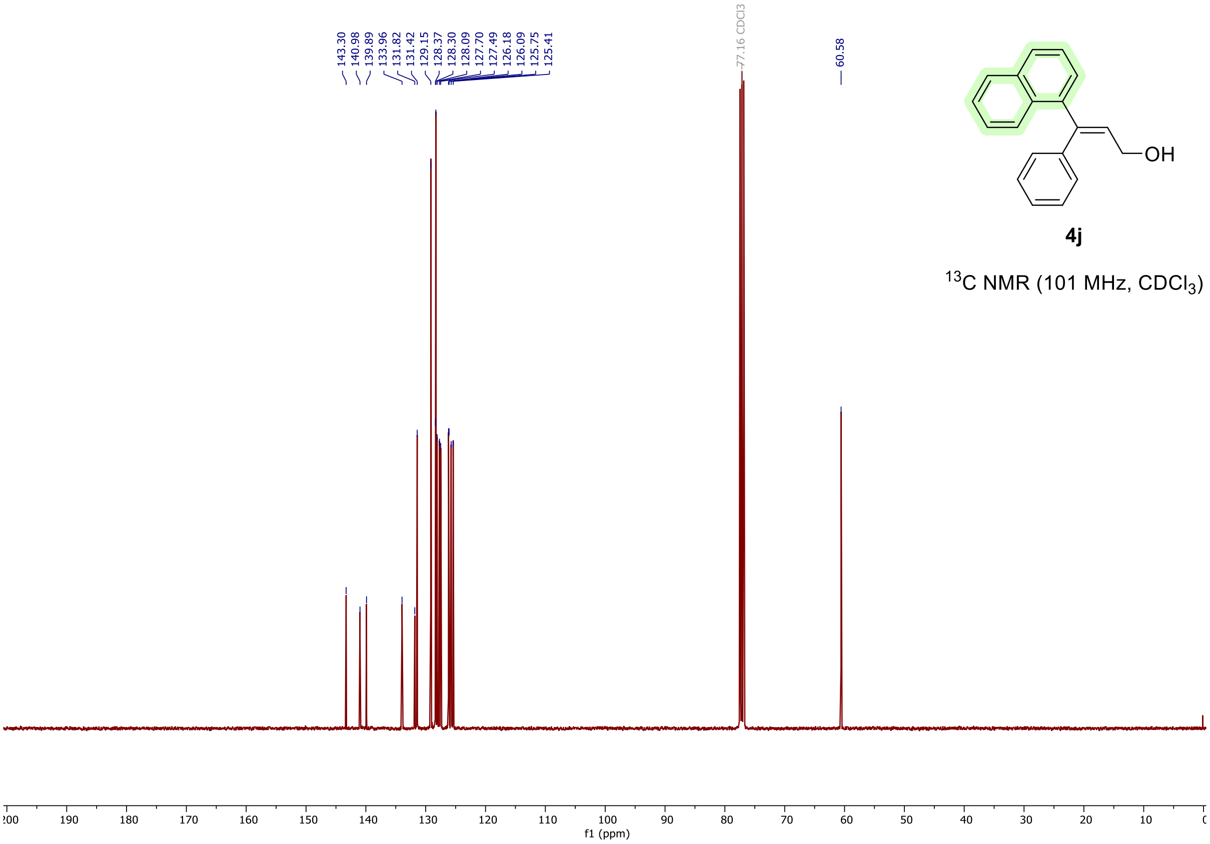

Spectra of 5a:

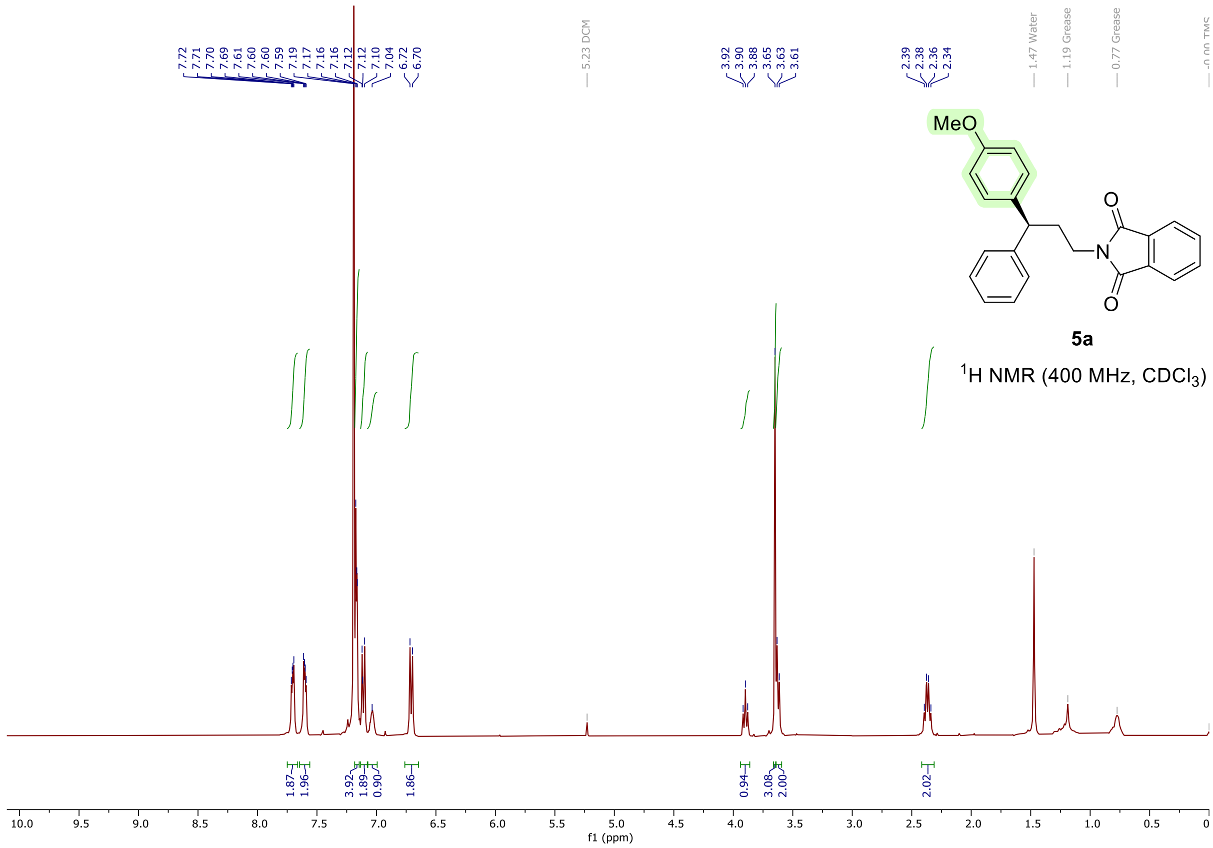

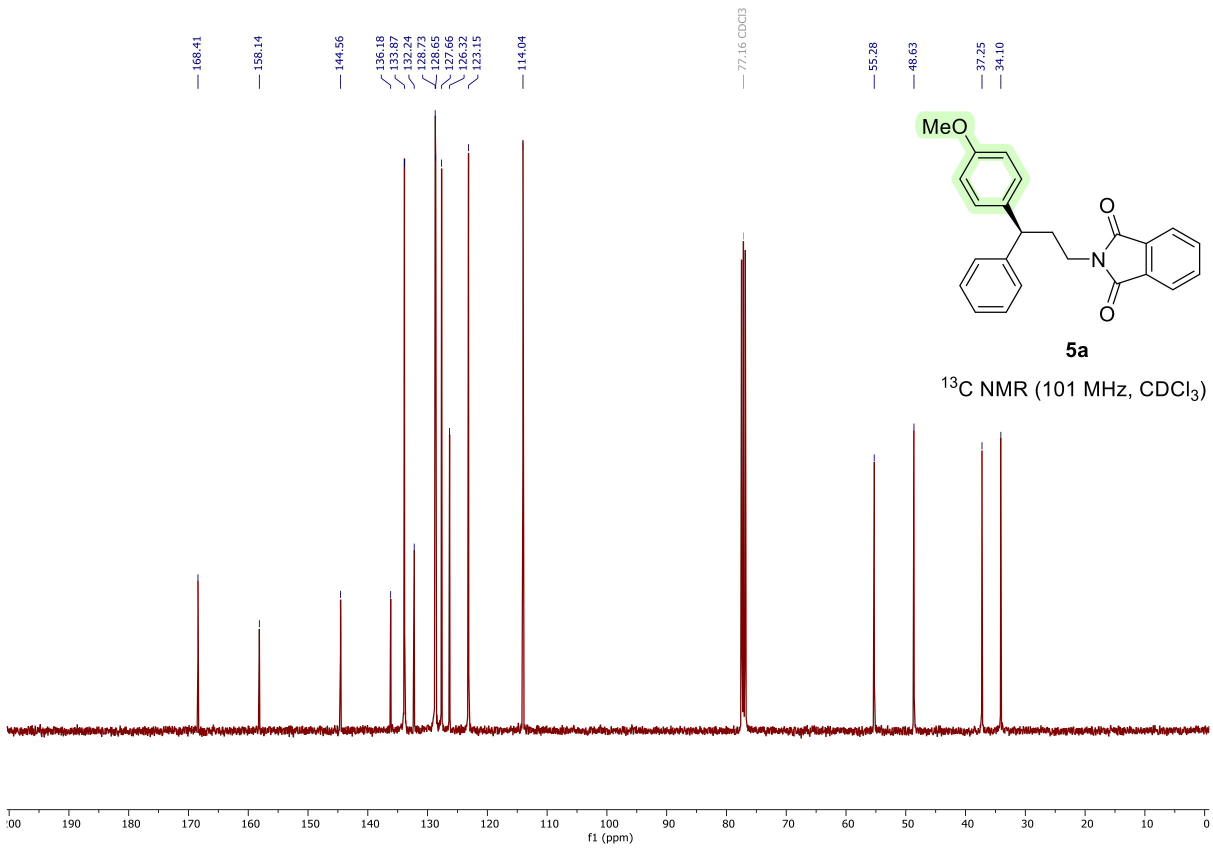

Spectra of 5b:

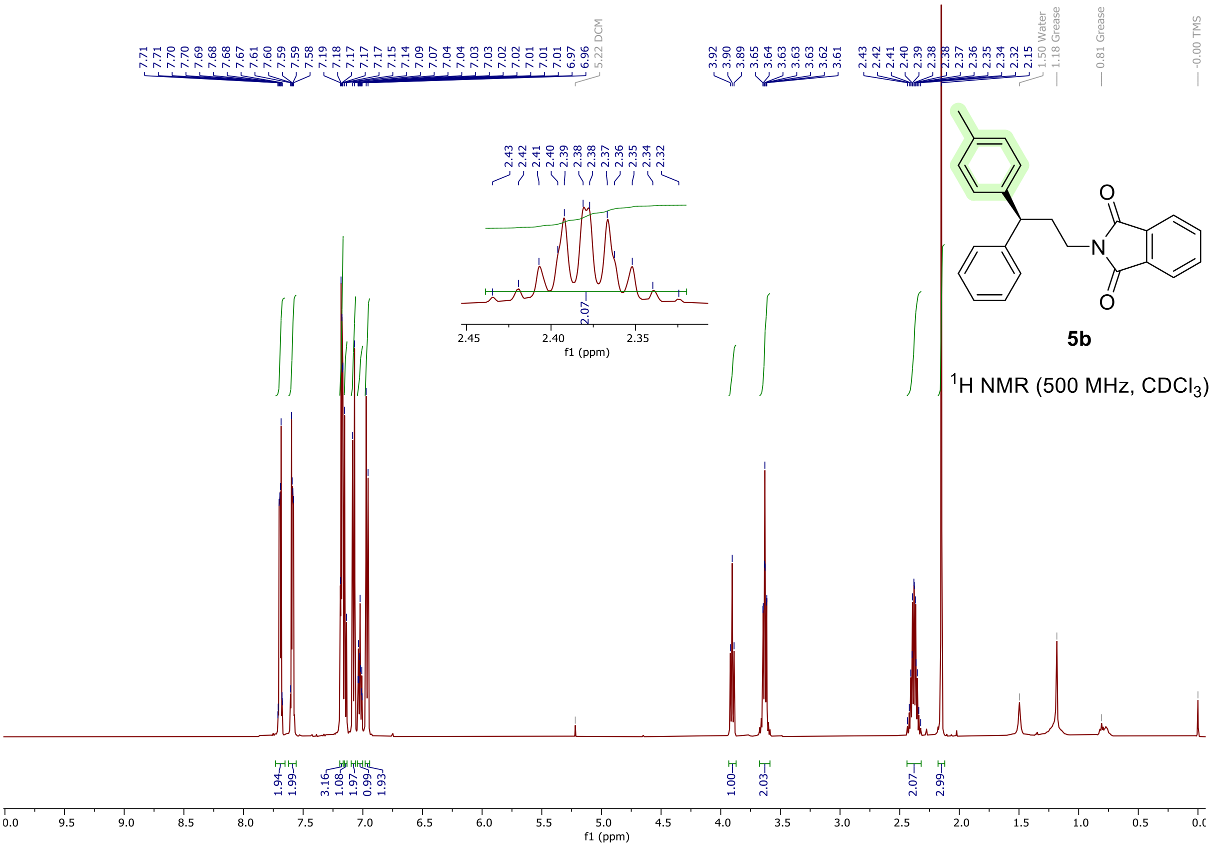

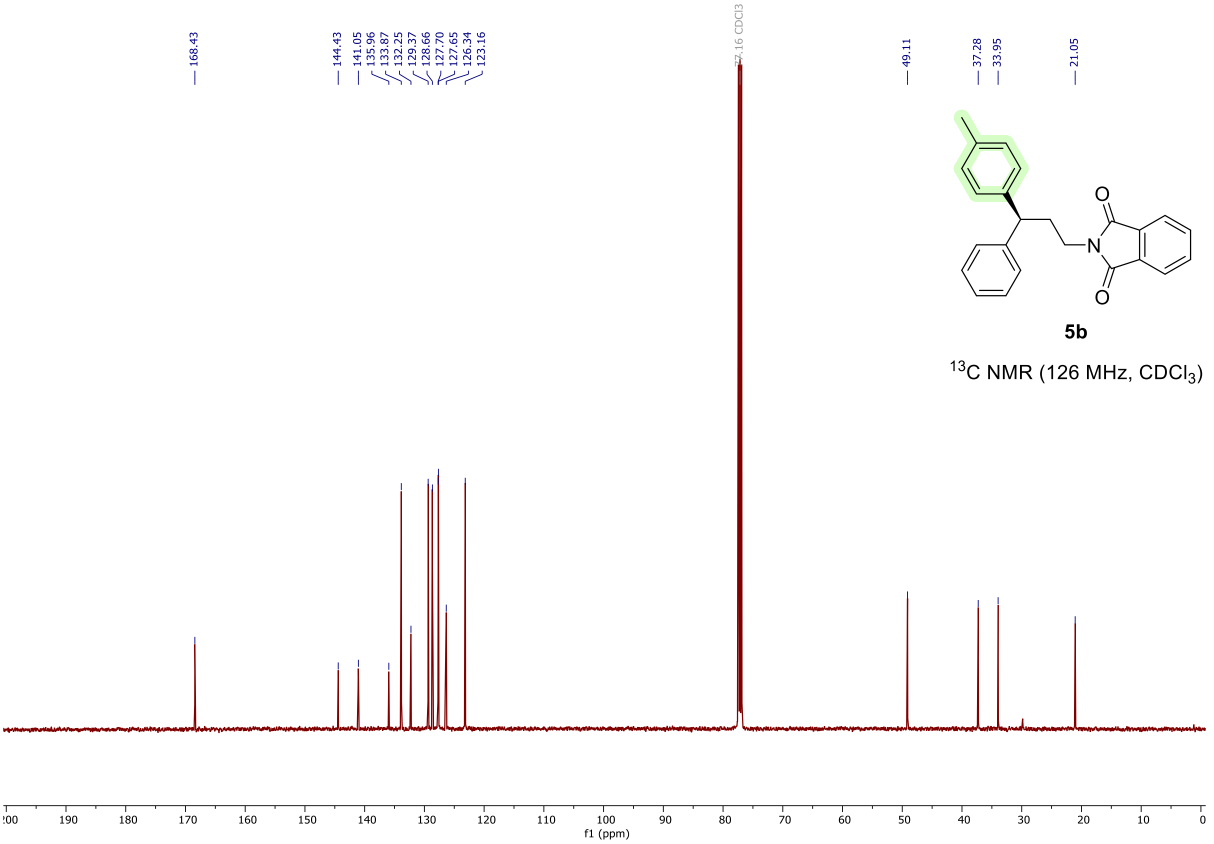

Spectra of 5c:

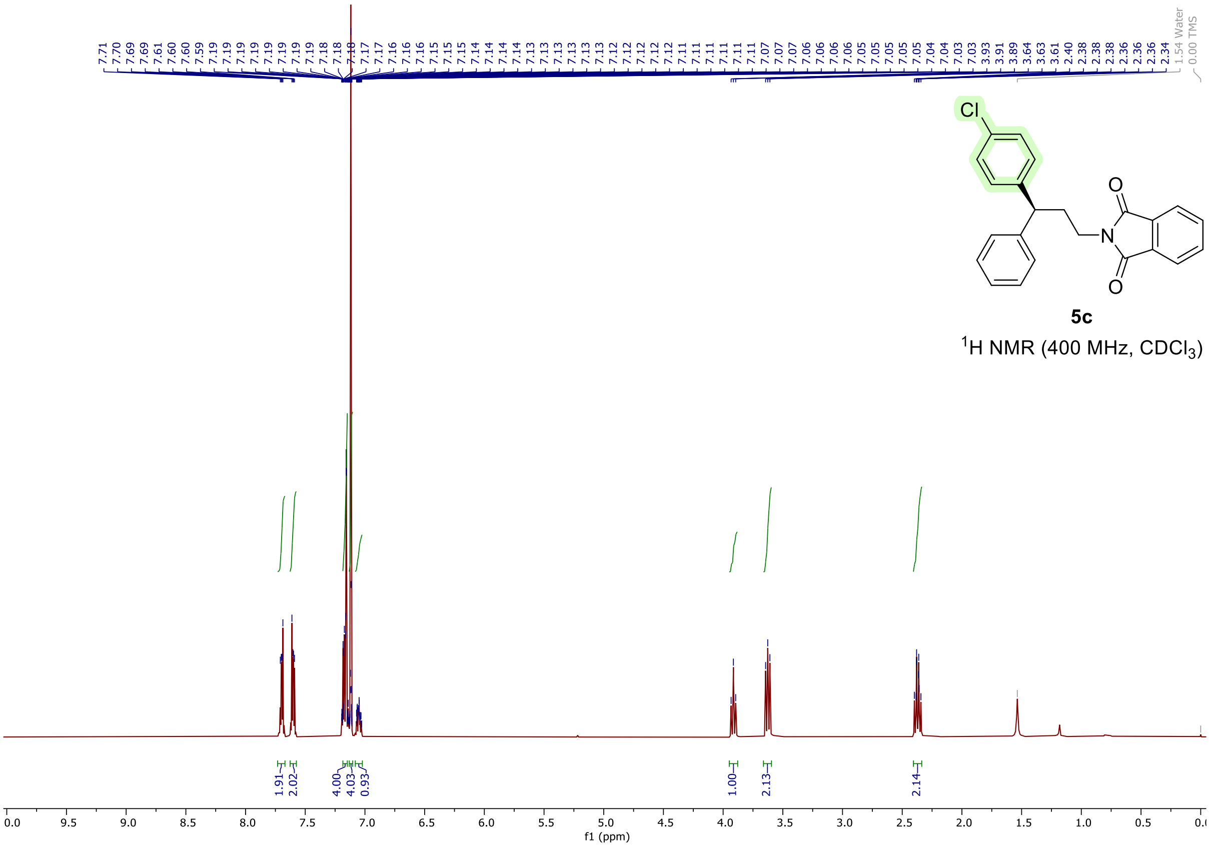

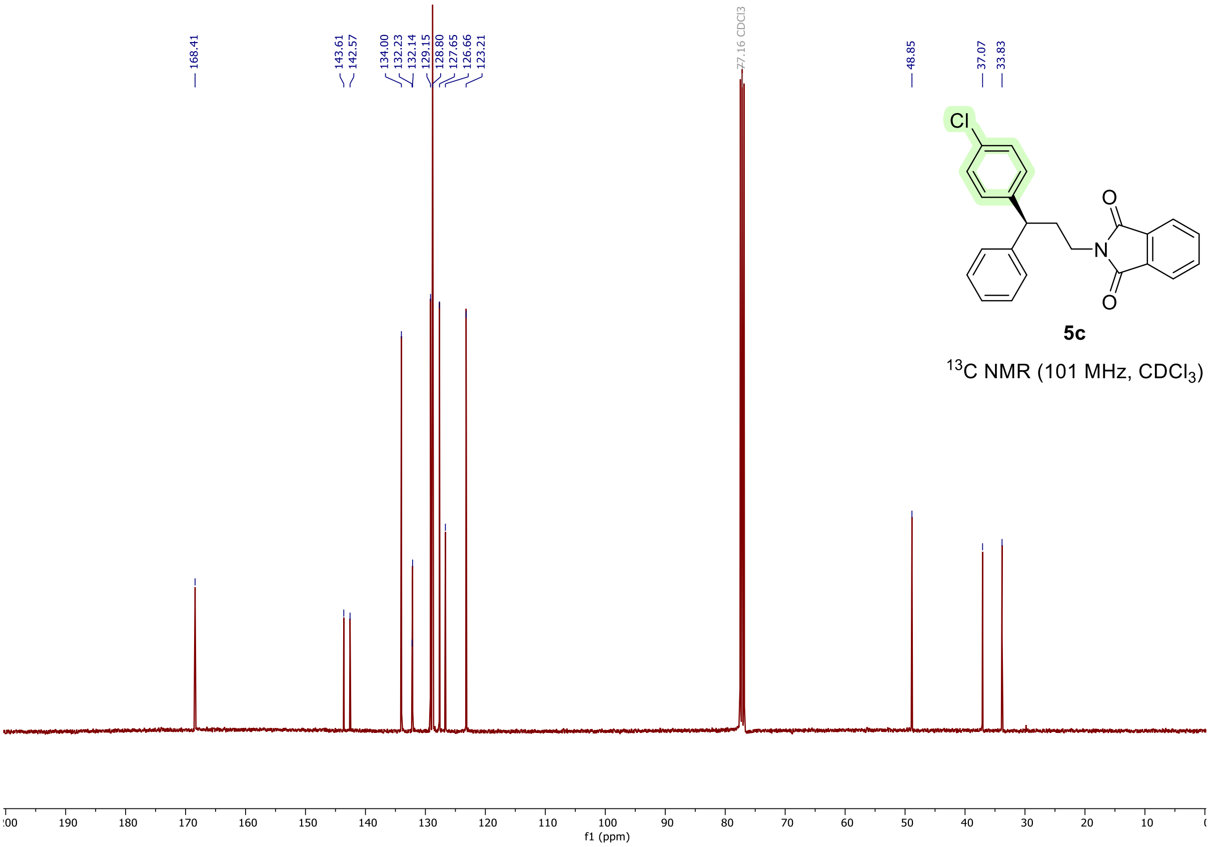

Spectra of 5d:

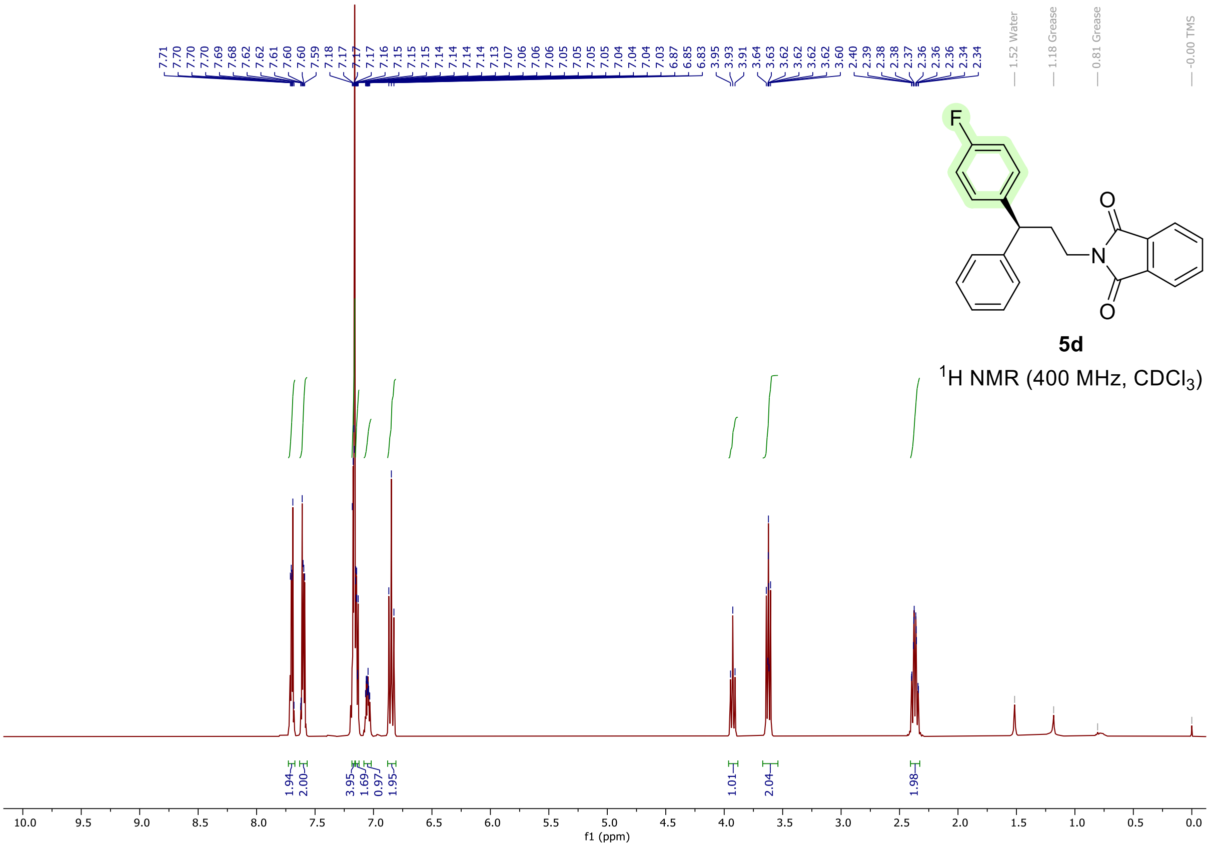

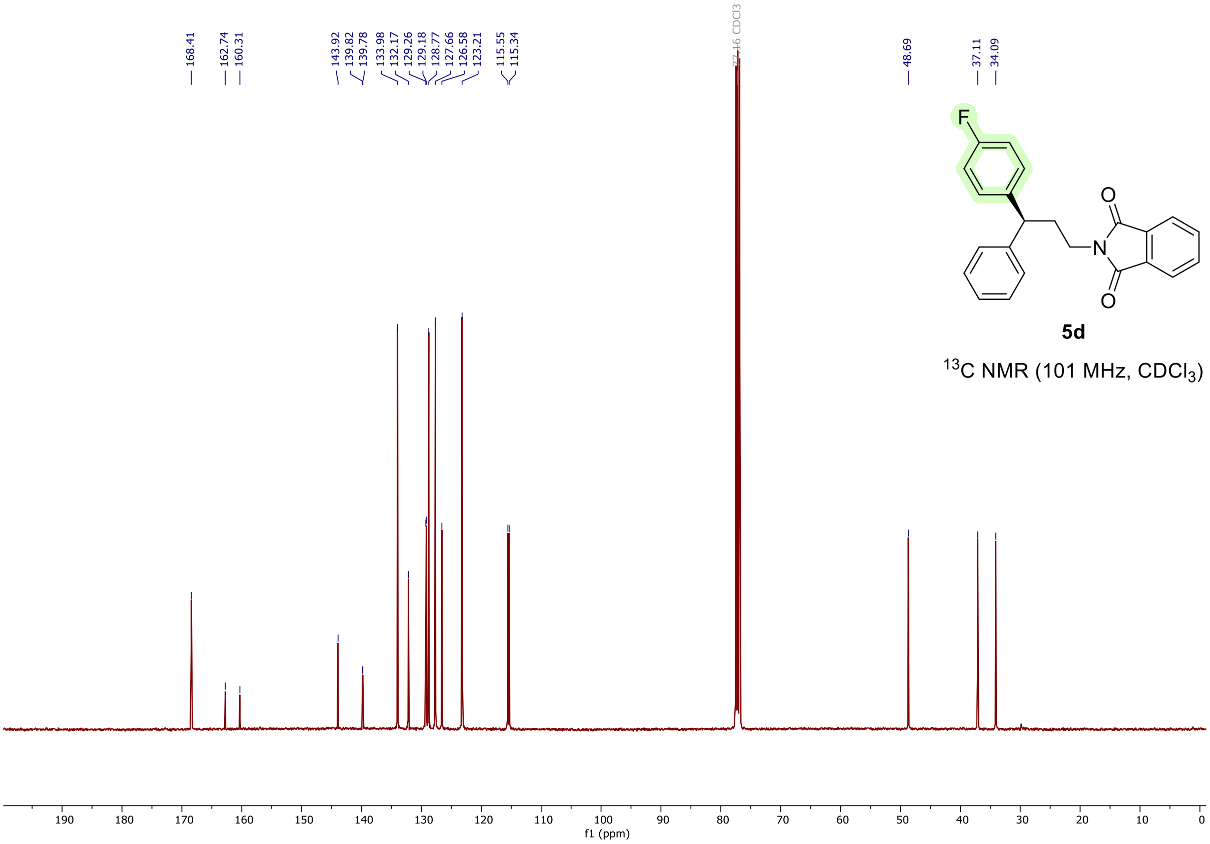

Spectra of 5e:

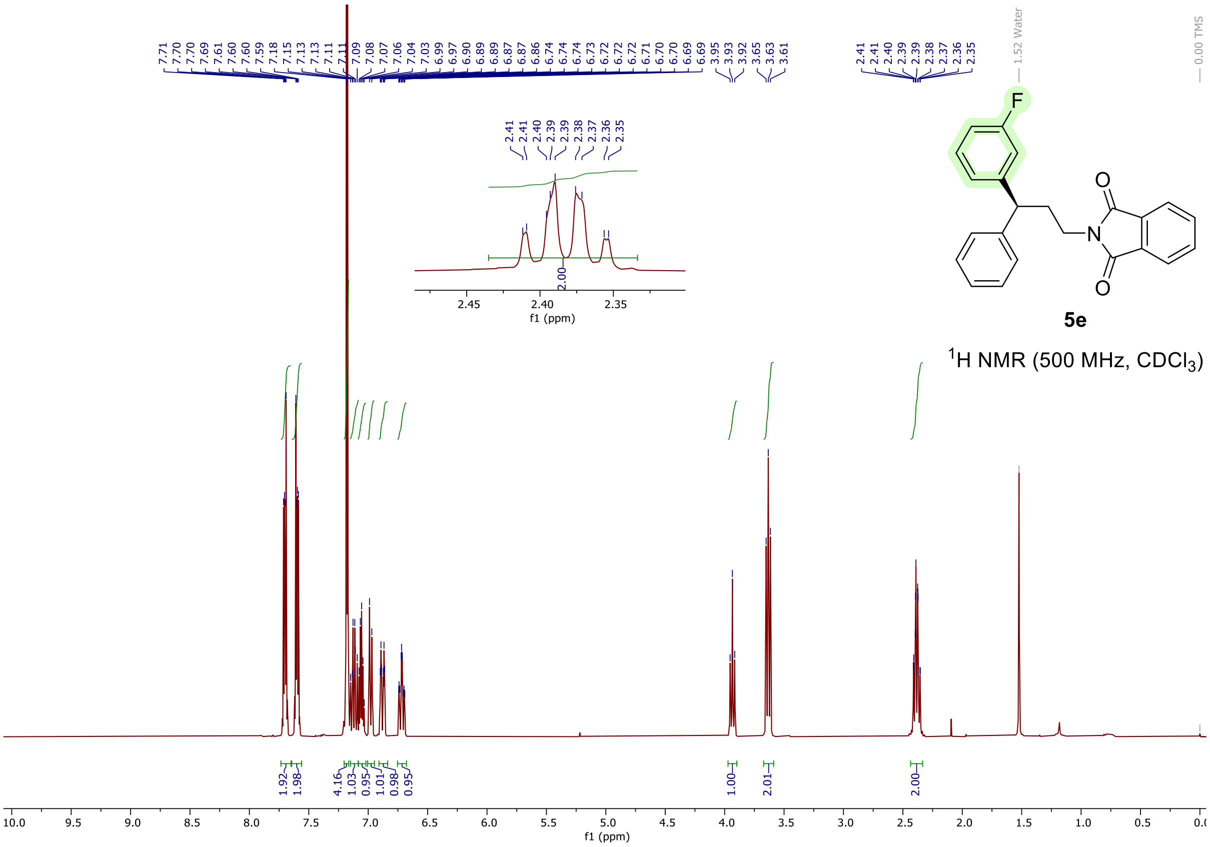

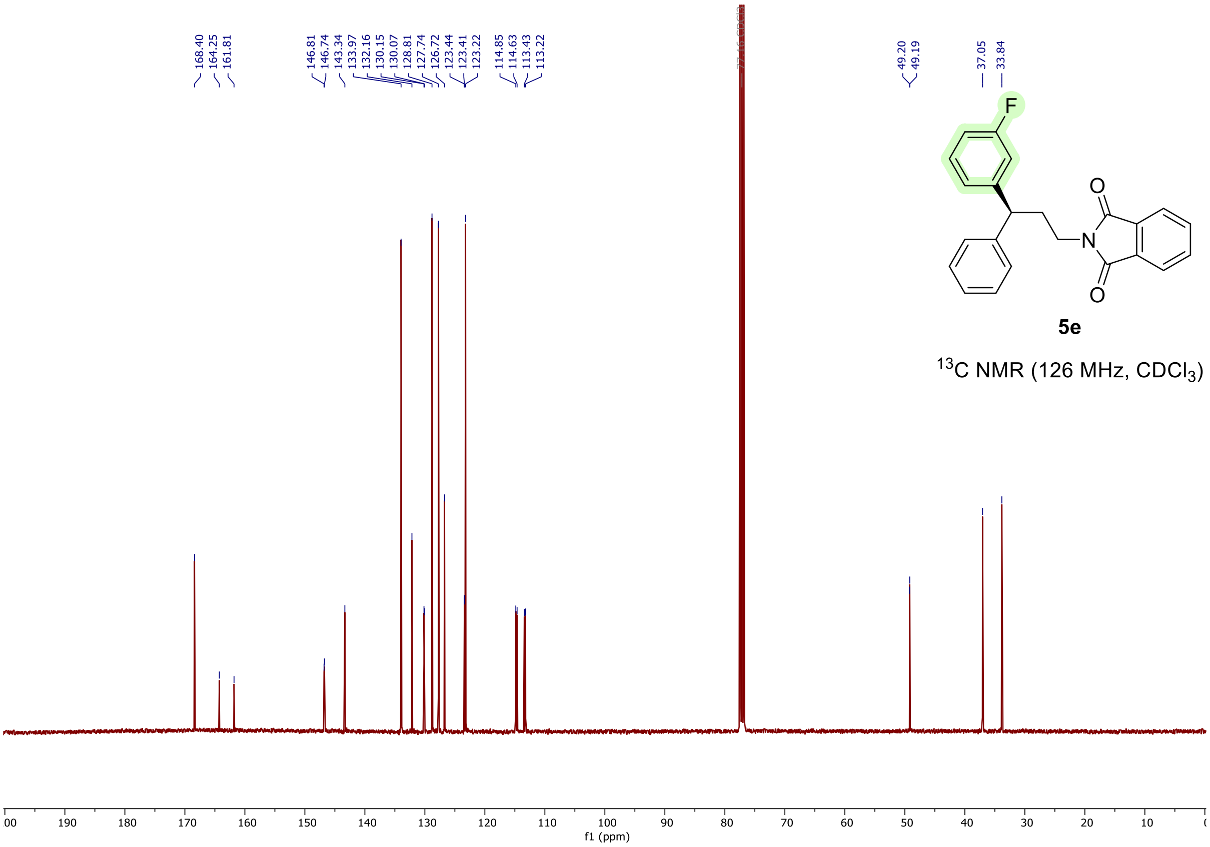

Spectra of 5f:

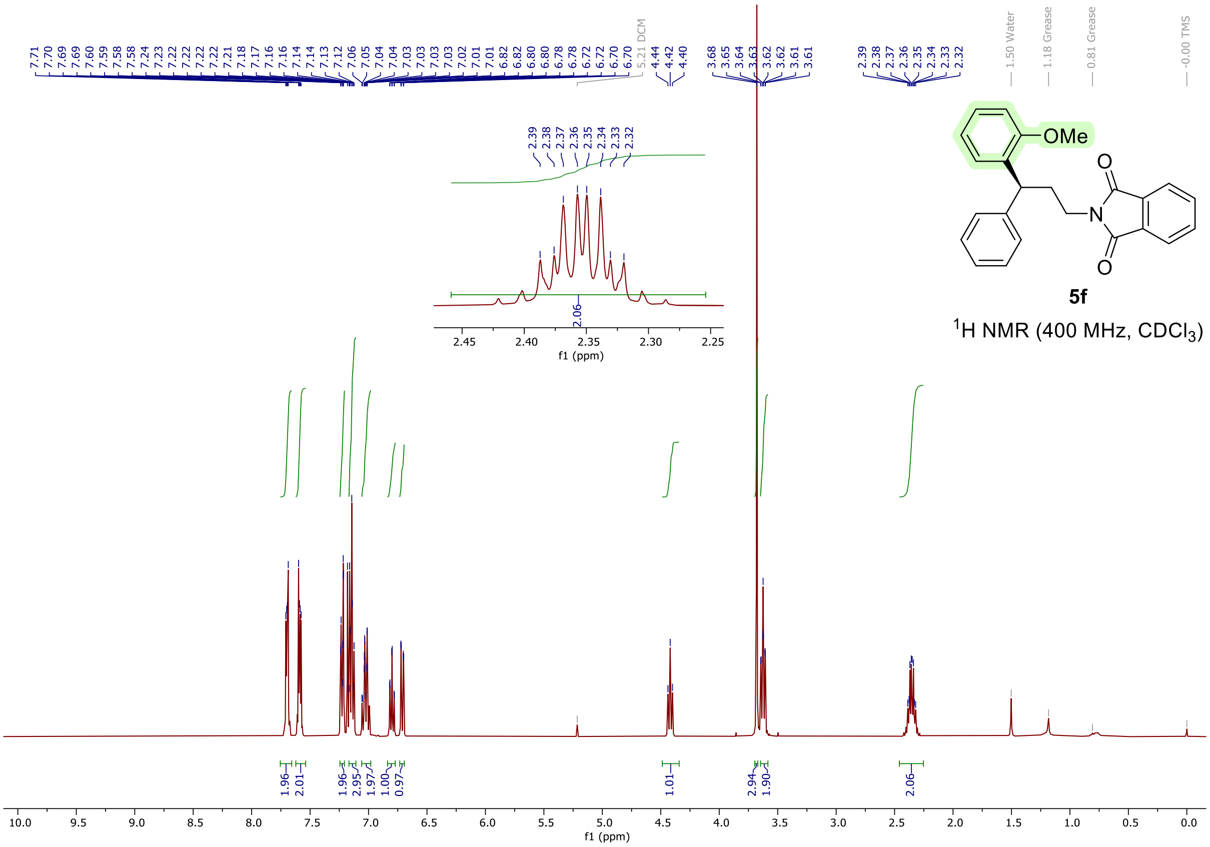

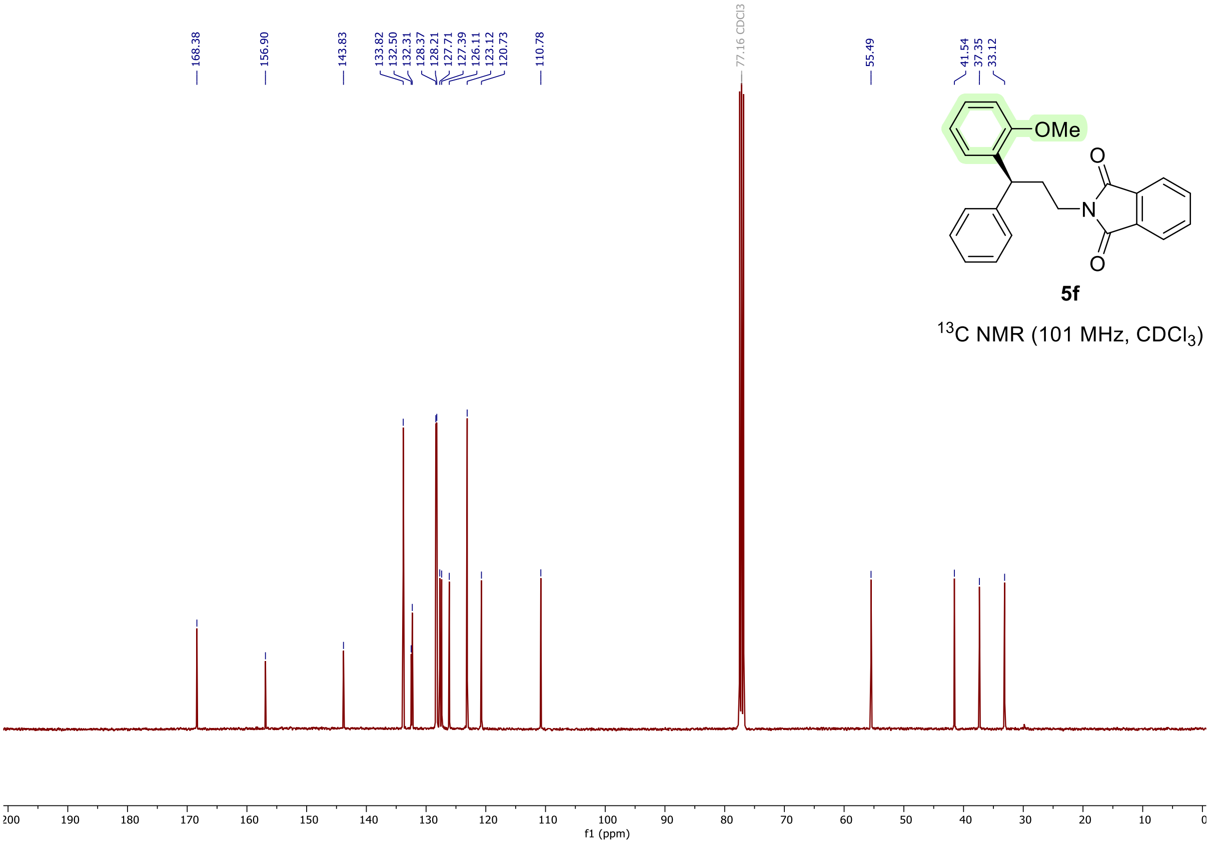

Spectra of 5g:

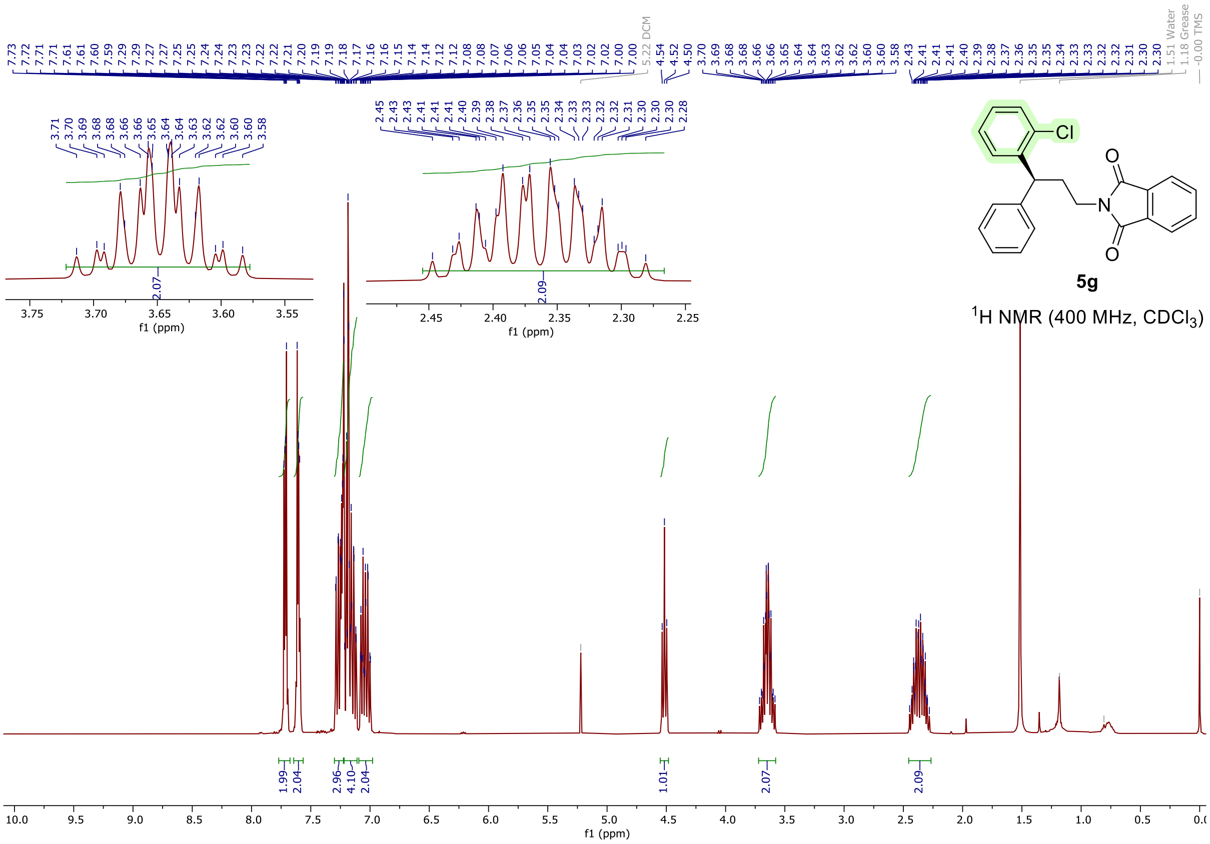

5g

^{1}H NMR (400 MHz, $CDCl_3$)

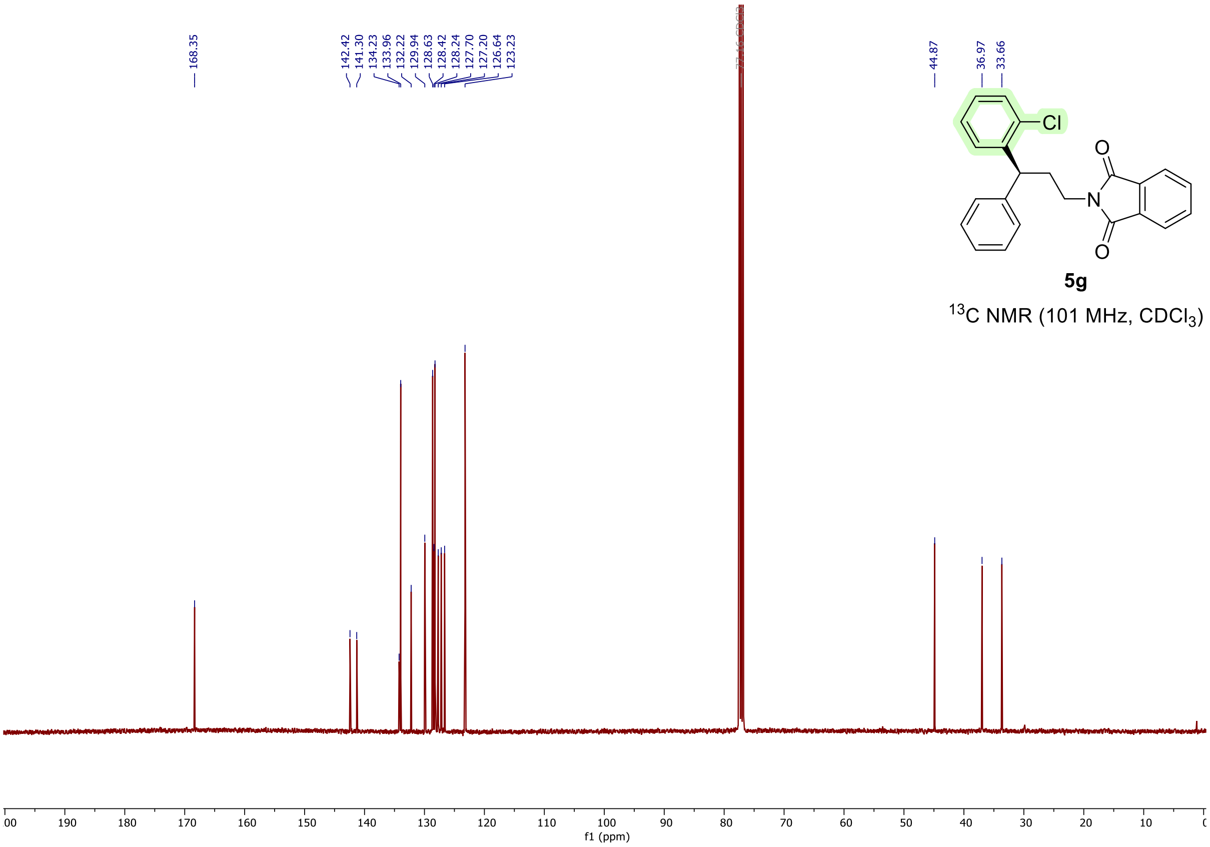

5g

^{13}C NMR (101 MHz, $CDCl_3$)

Spectra of 5h:

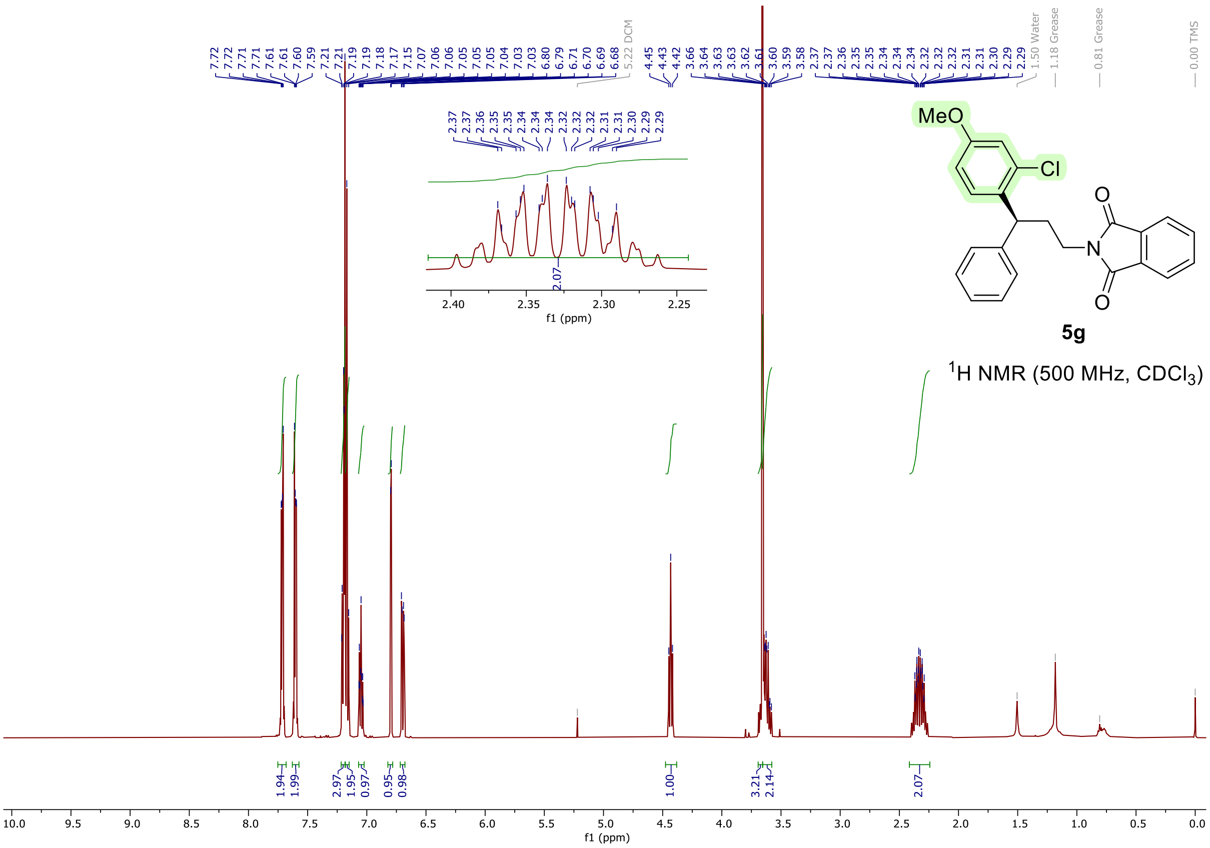

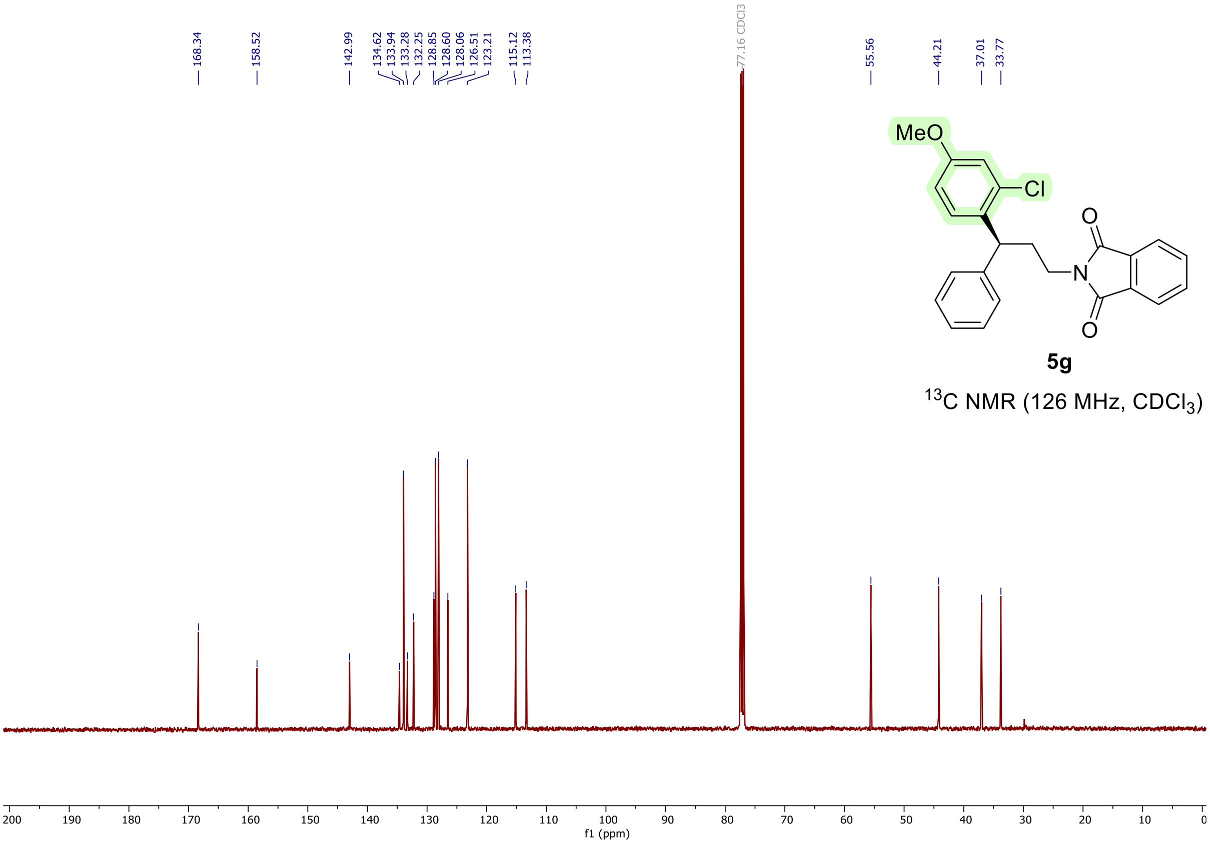

Spectra of 5i:

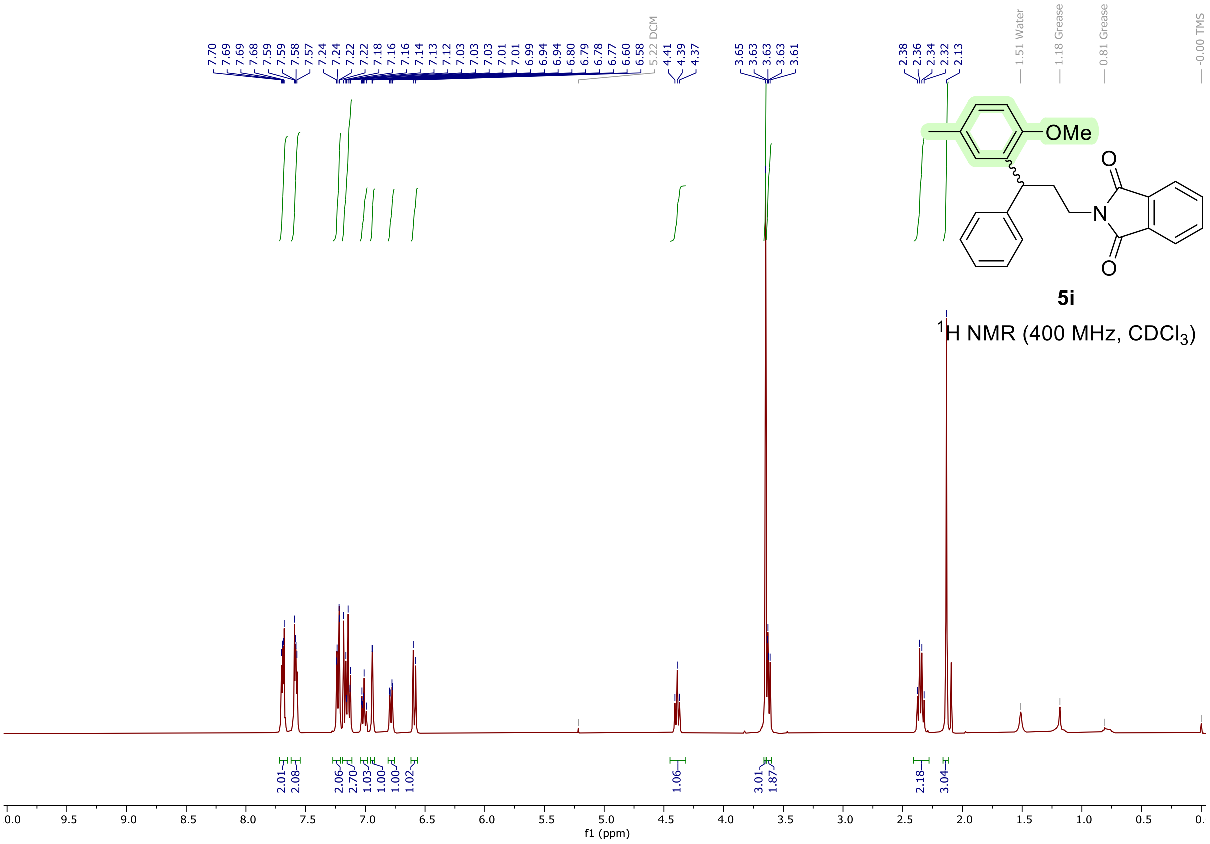

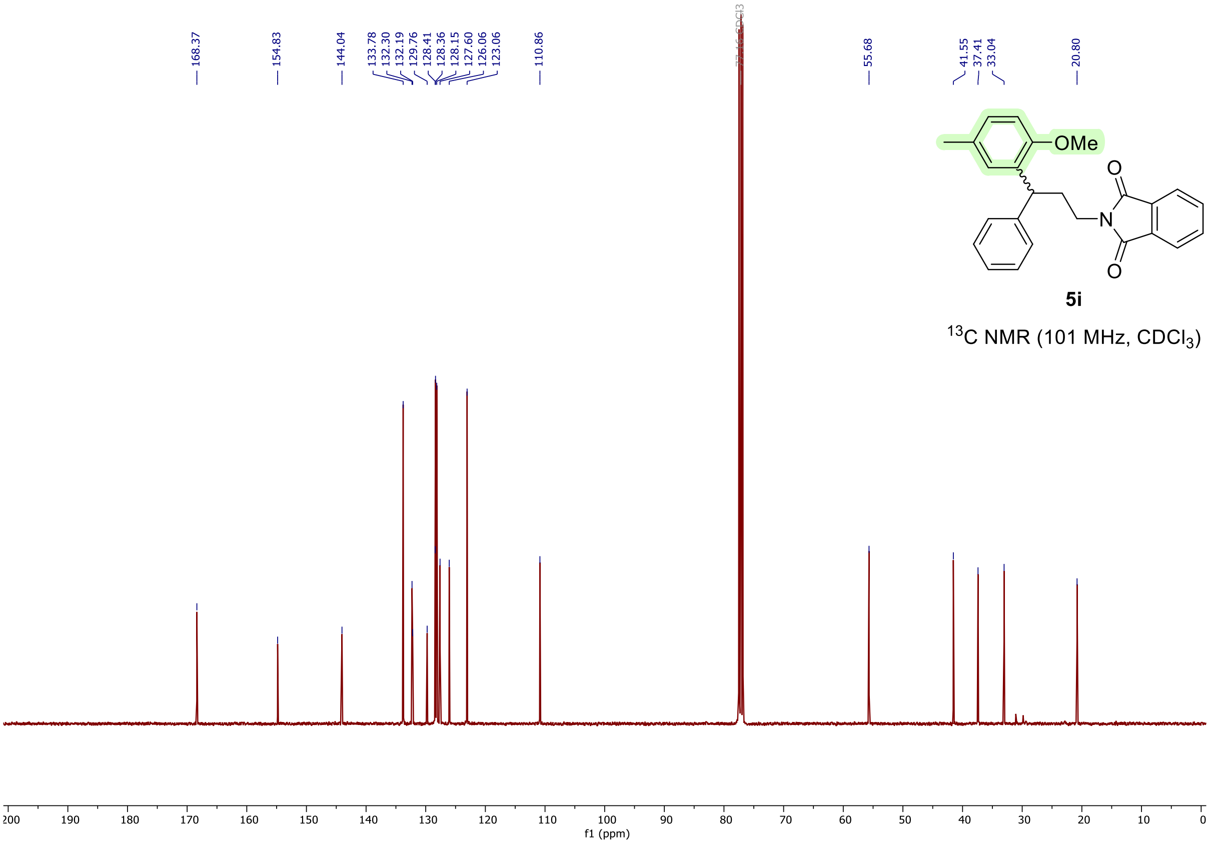

Spectra of 5j:

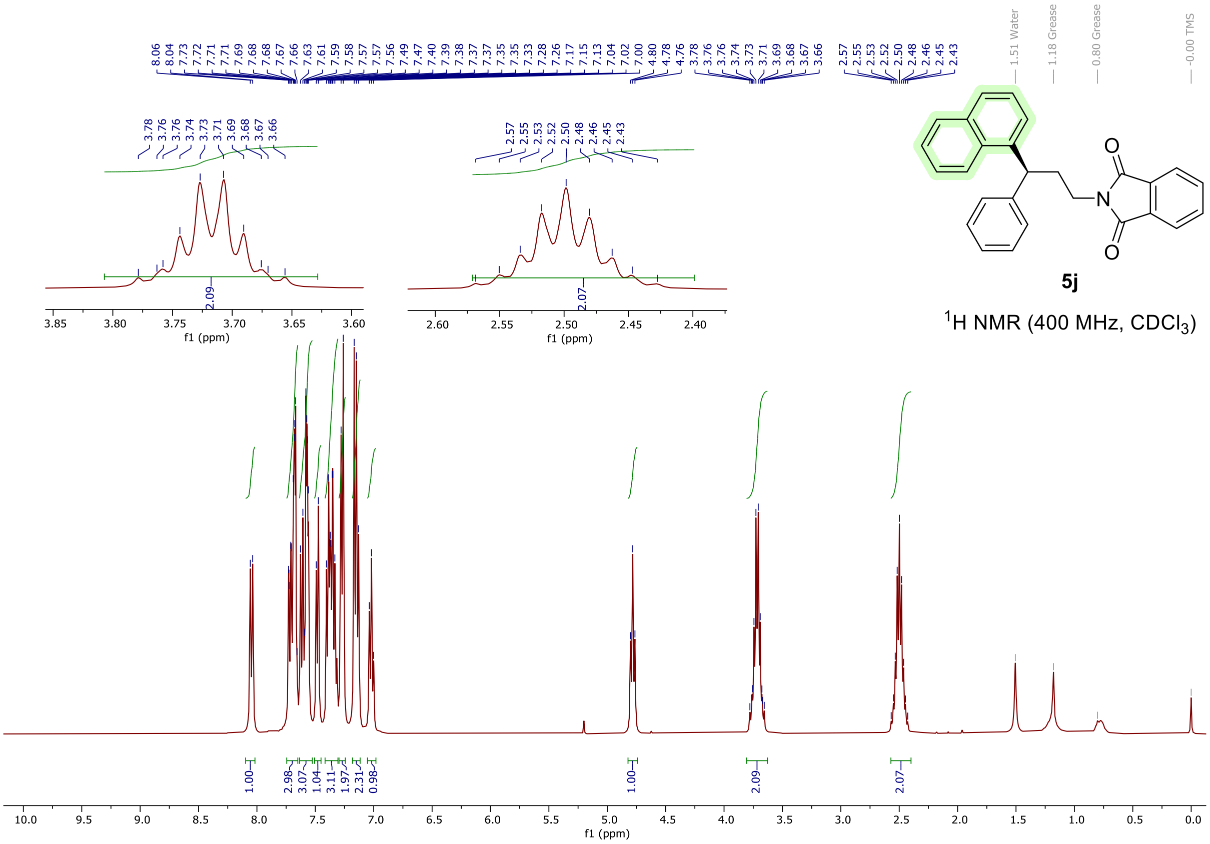

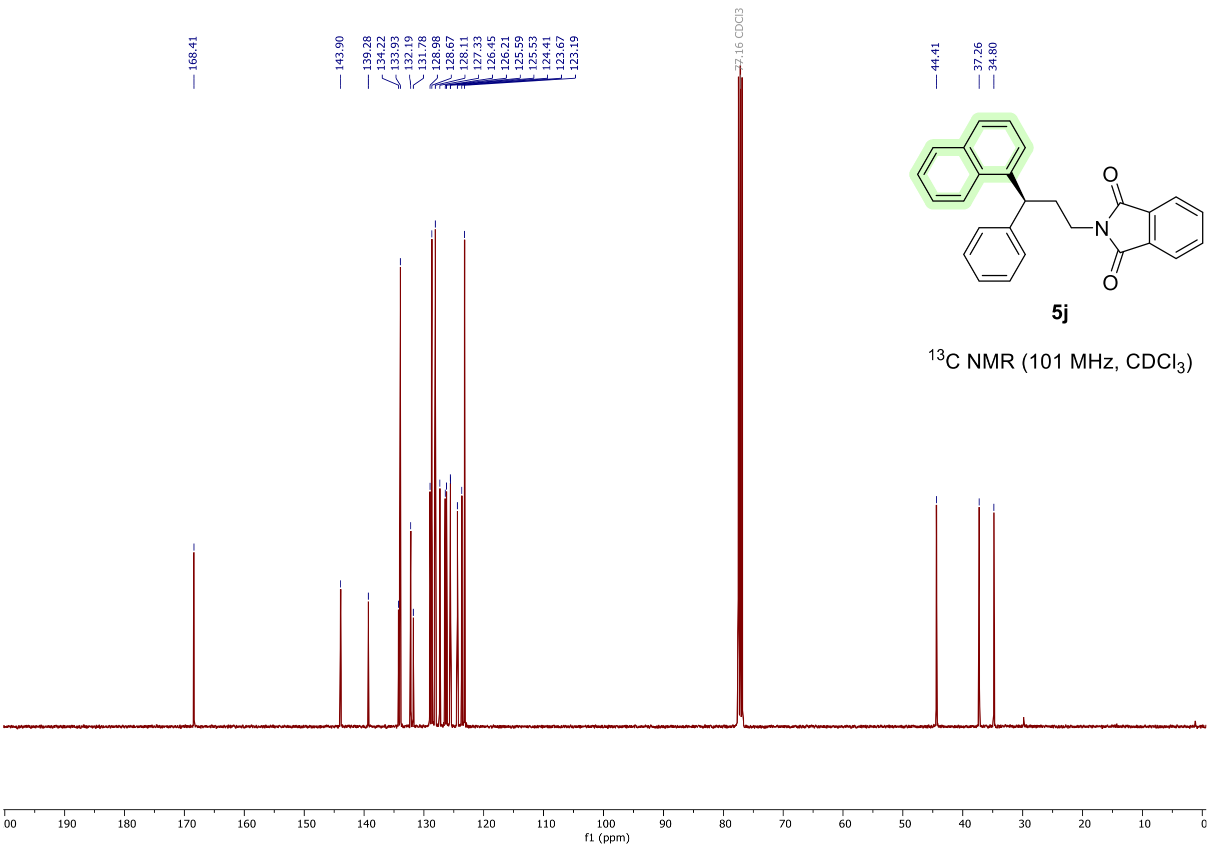

Spectra of 6b:

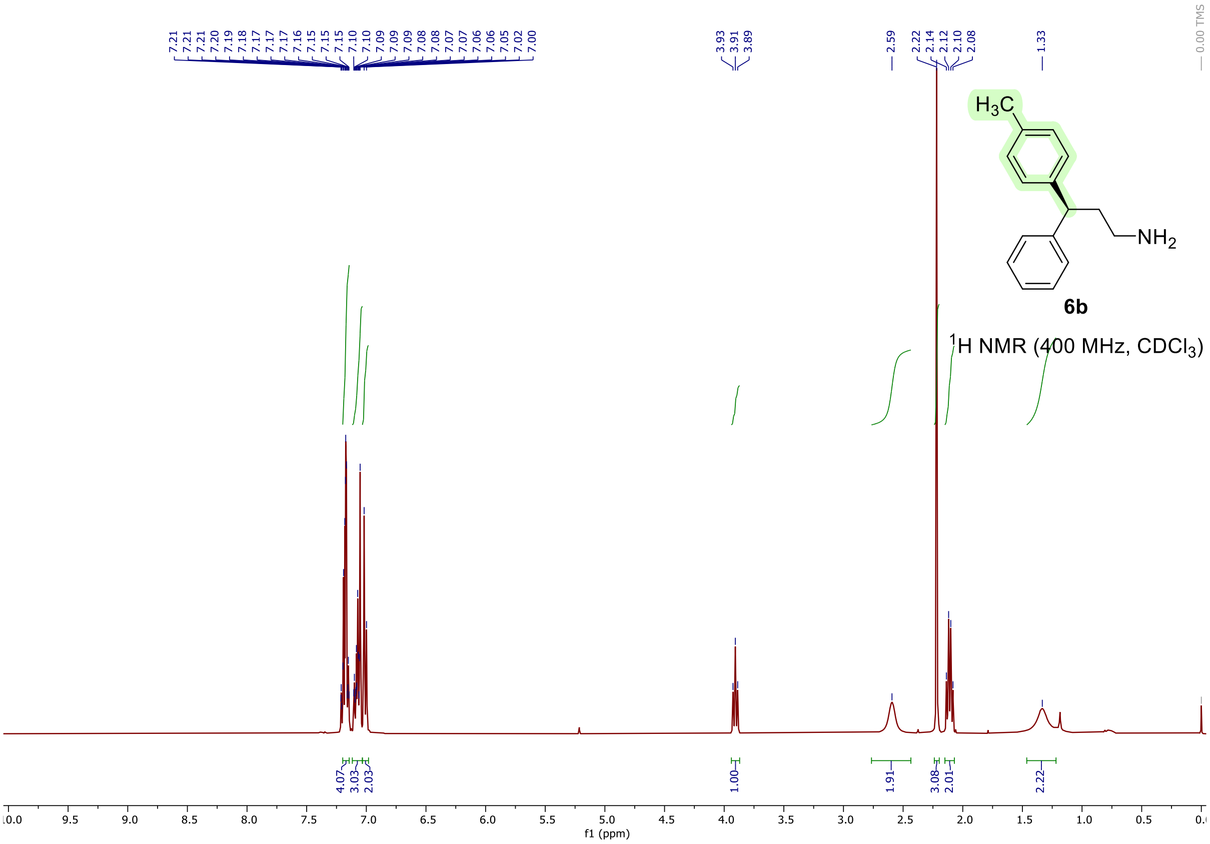

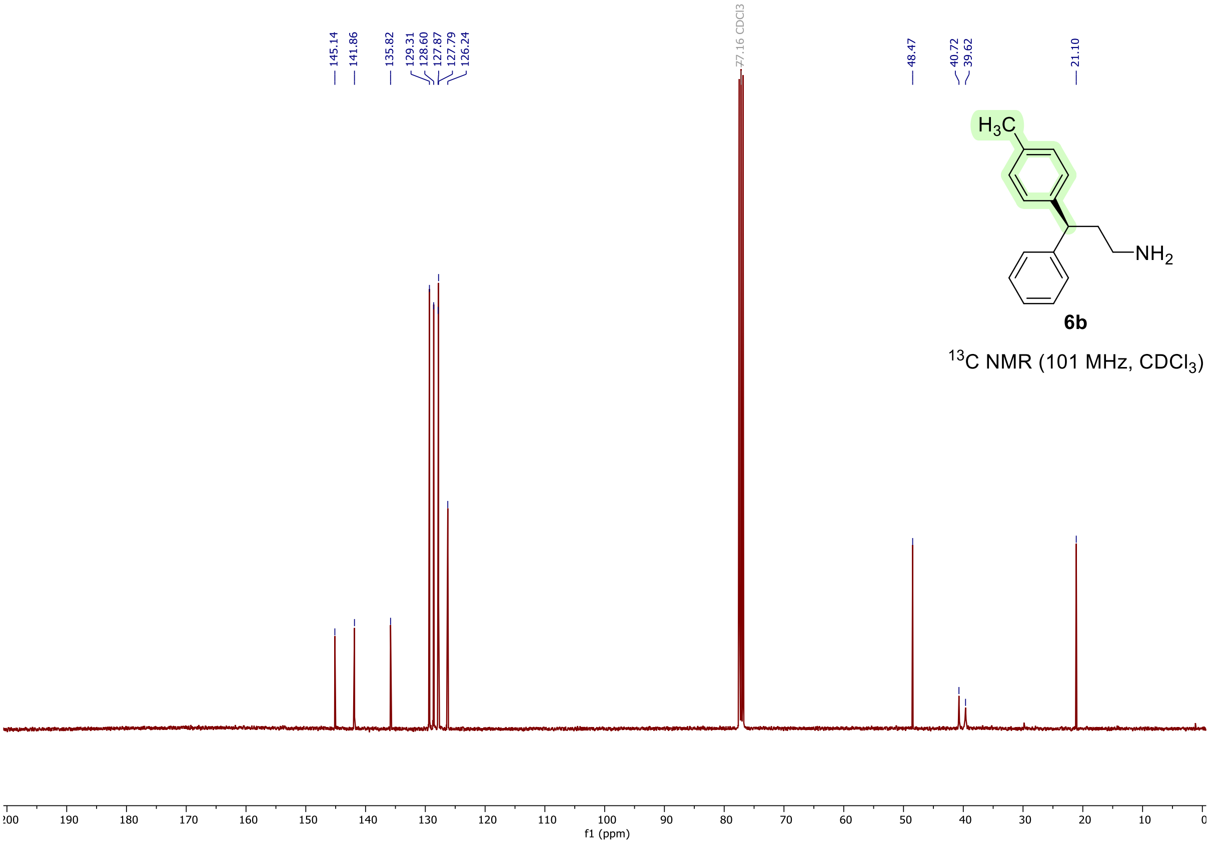

Spectra of 6g:

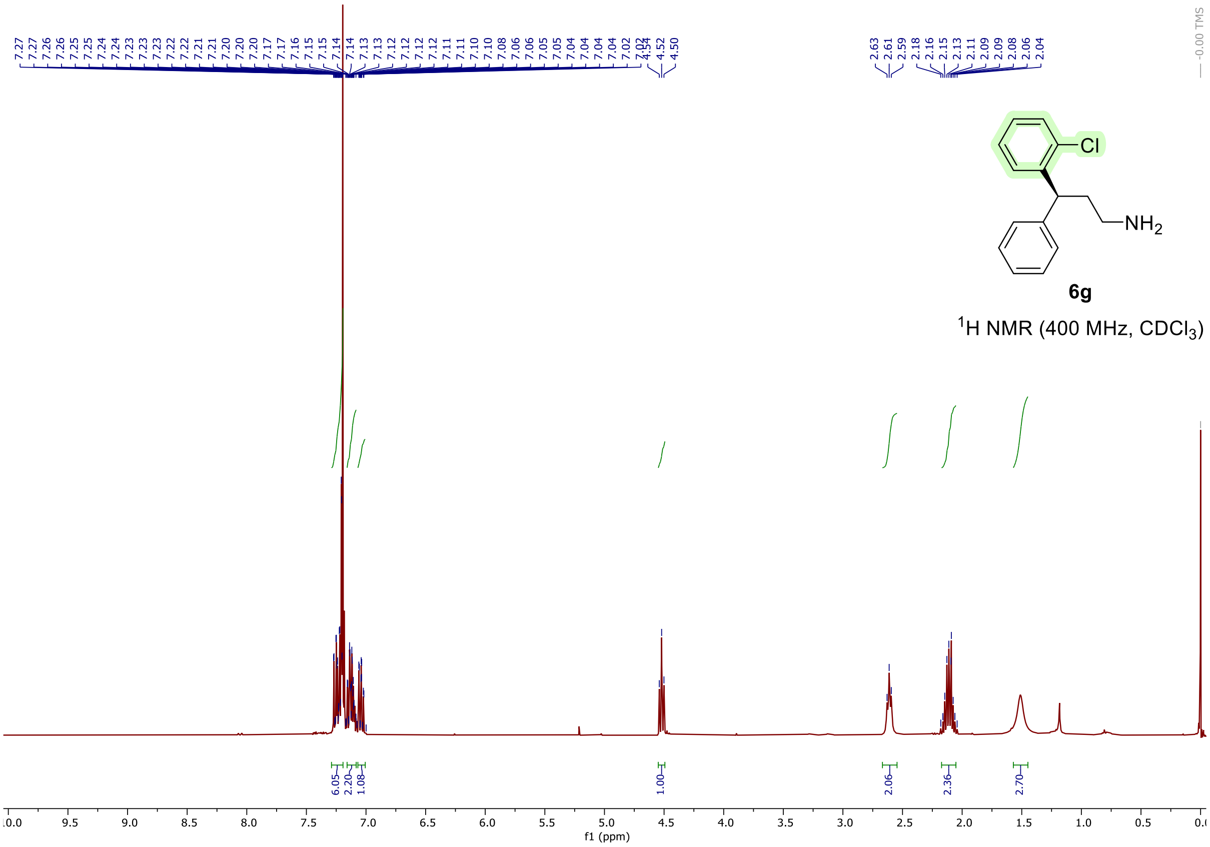

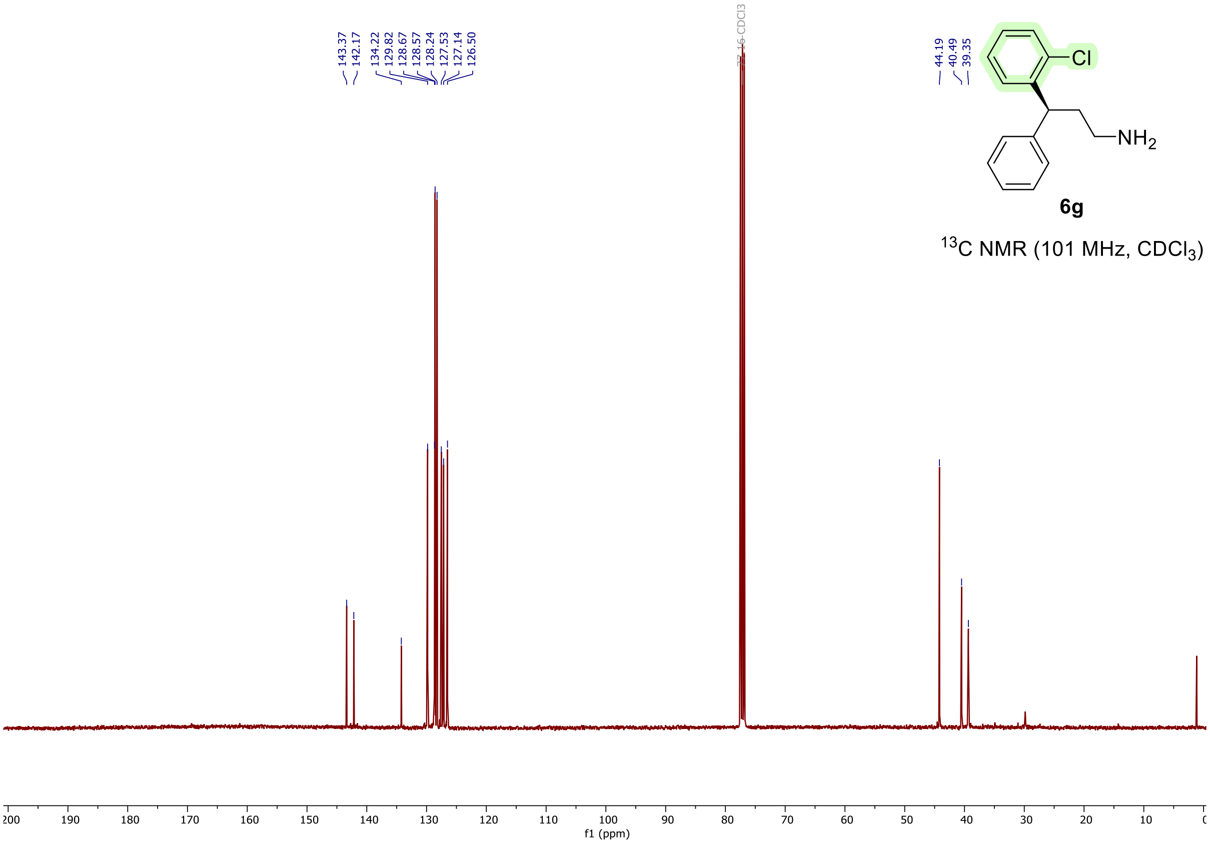

Spectra of 6h:

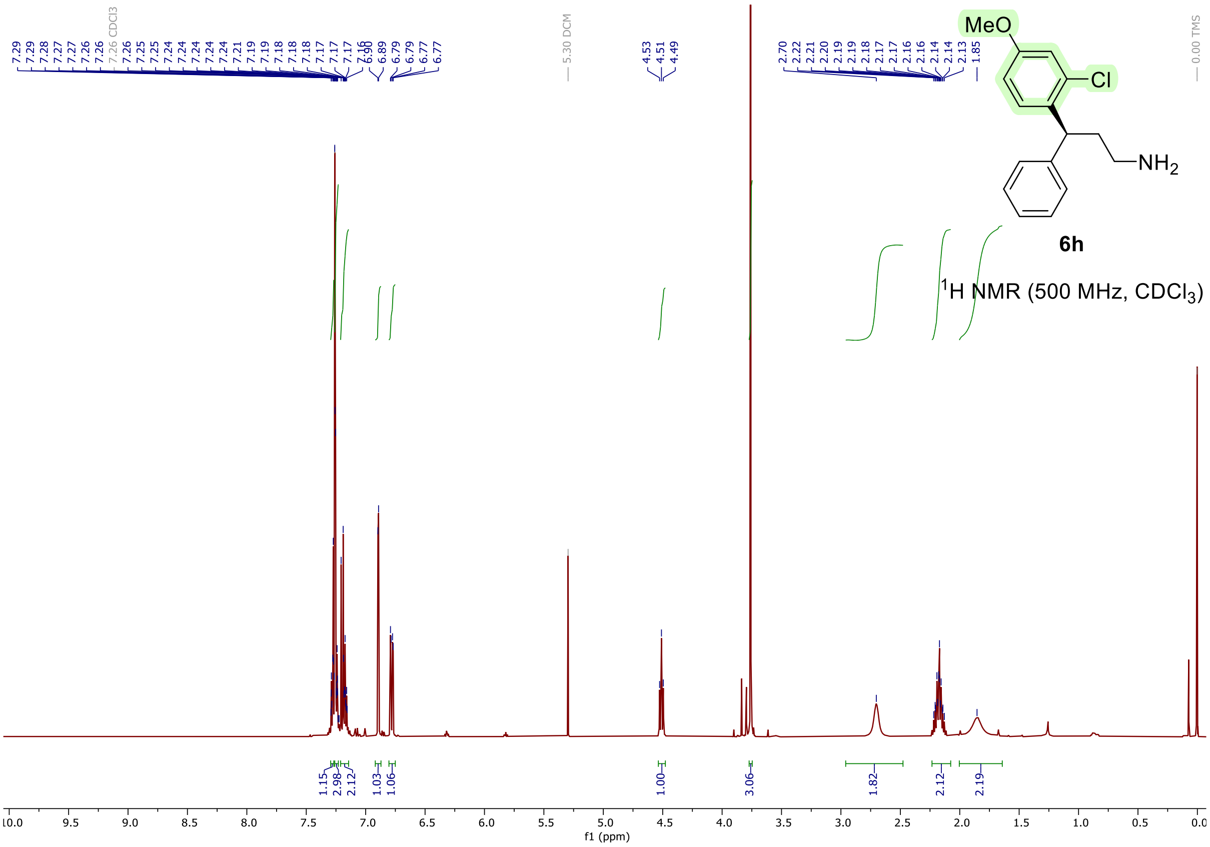

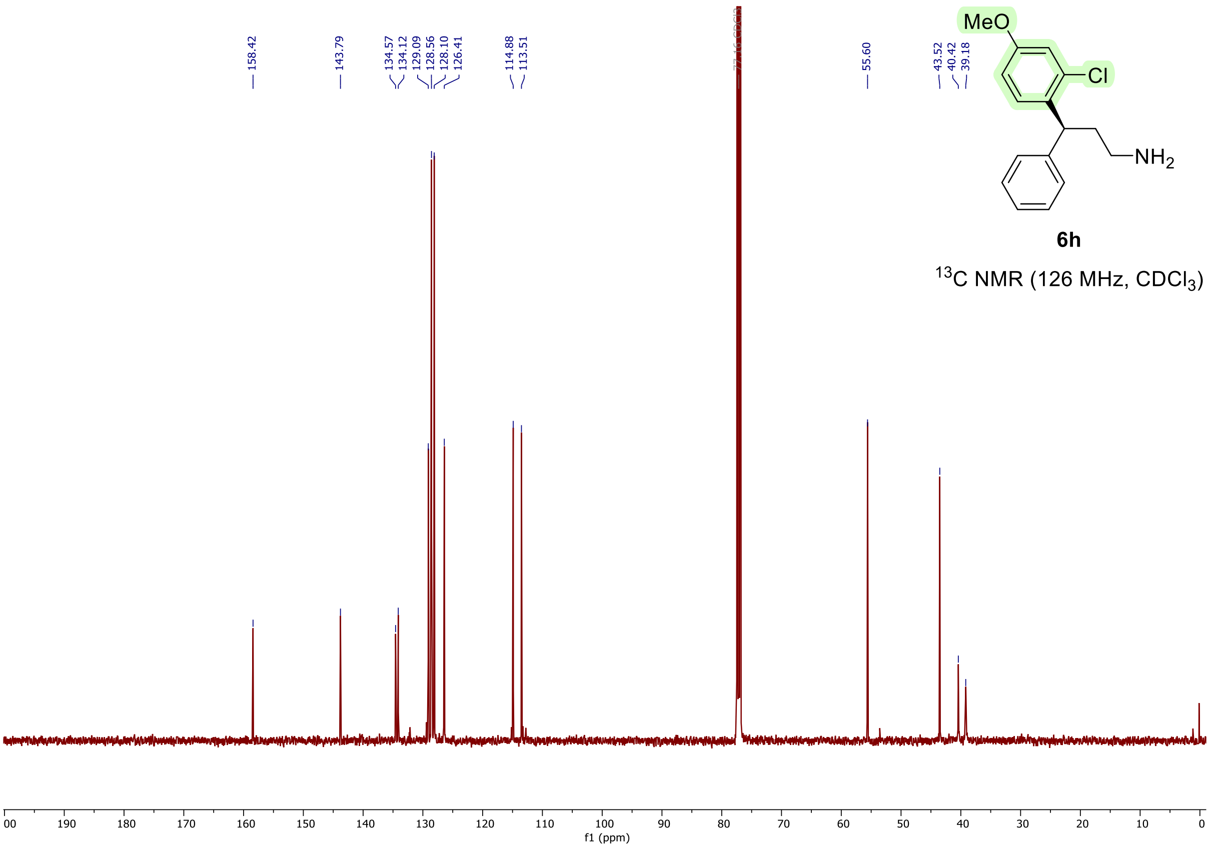

Spectra of (R)-6i:

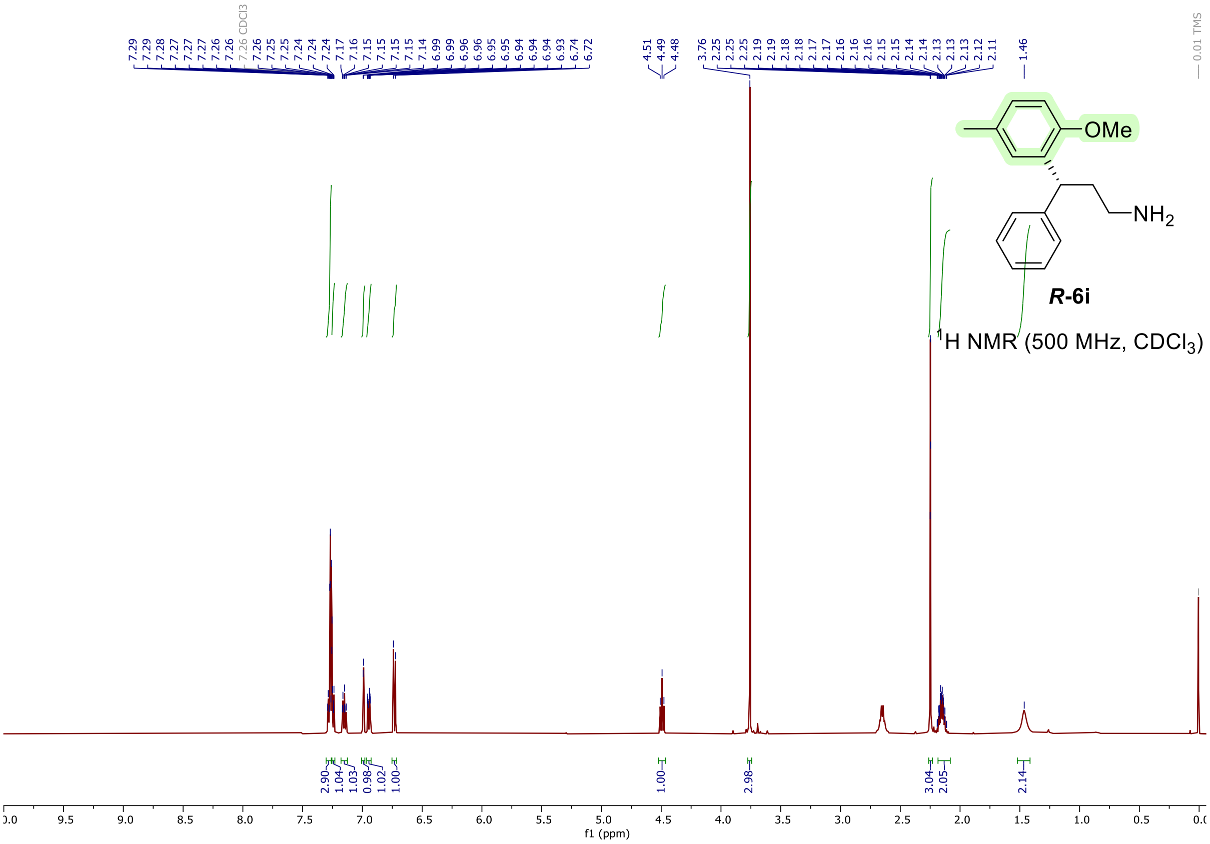

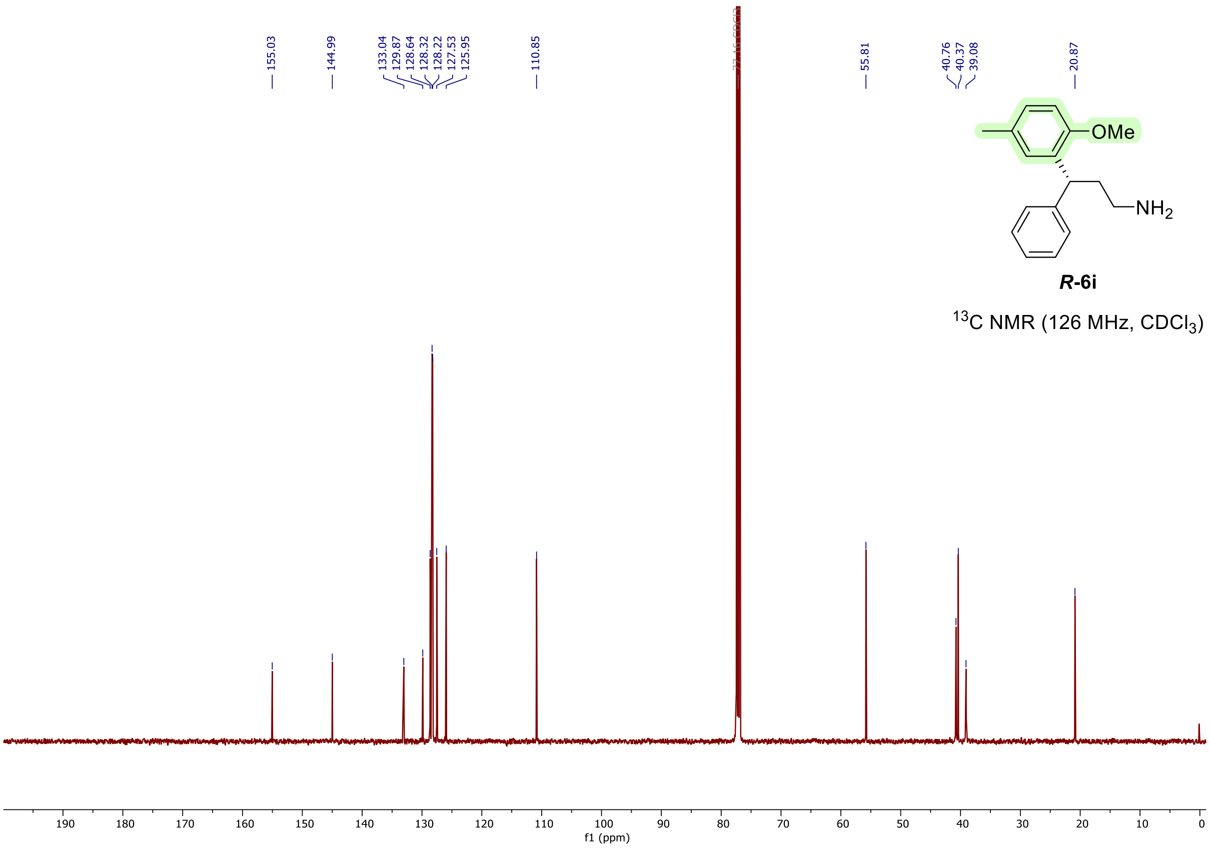

Spectra of 7:

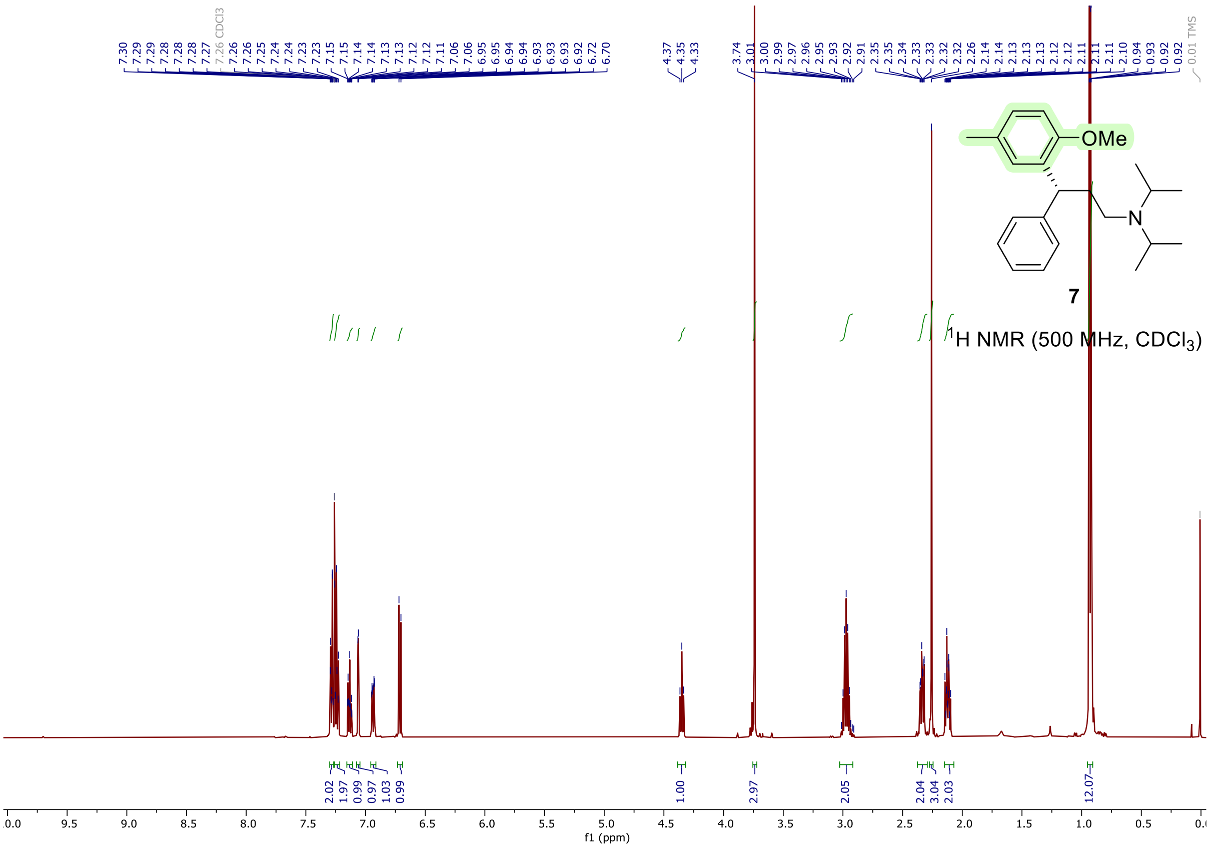

OMe
N
7
^{1}H NMR (500 MHz, CDCl$_3$)
f1 (ppm)

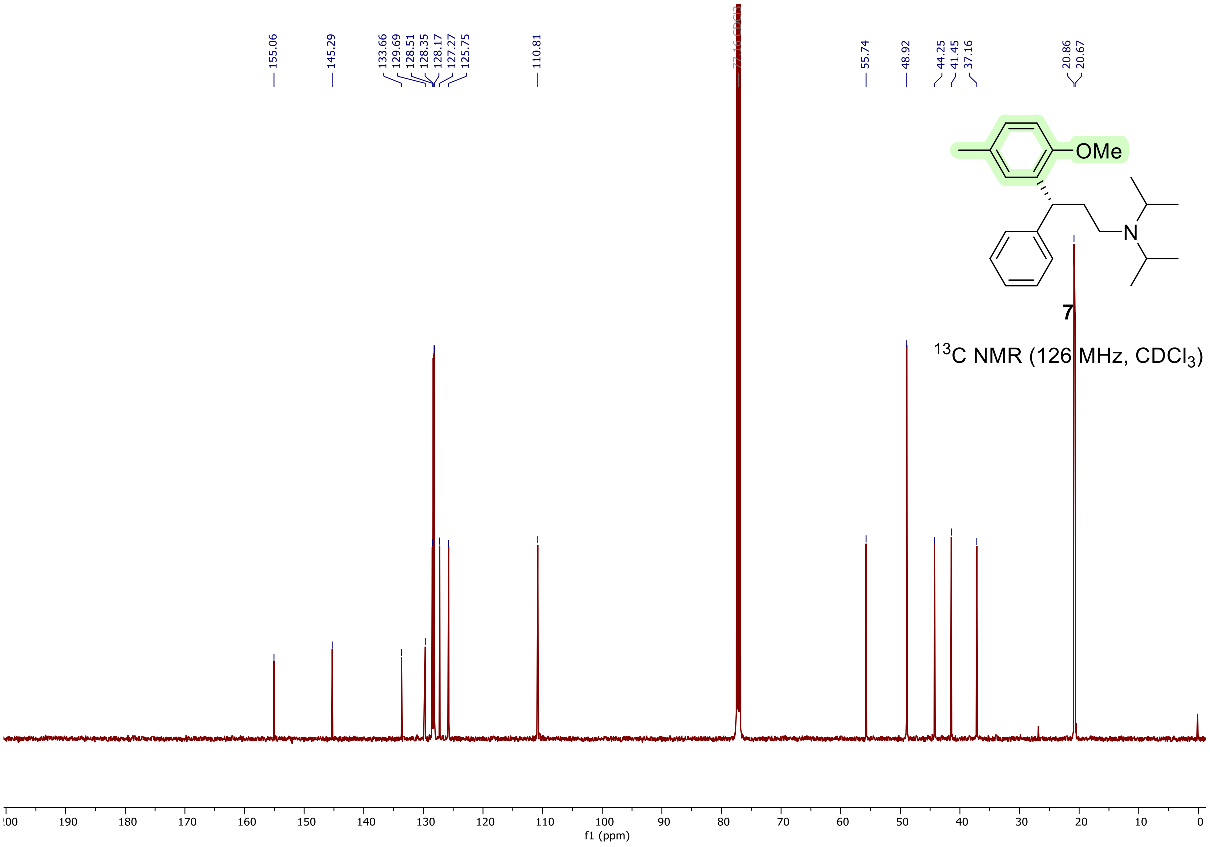

155.06
145.29
133.66
129.69
128.51
128.35
128.17
127.27
125.75
110.81
55.74
48.92
44.25
41.45
37.16
20.86
20.67
OMe
N
7
^{13}C NMR (126 MHz, CDCl$_3$)
f1 (ppm)

Spectra of 8 ((R)-Tolterodine):

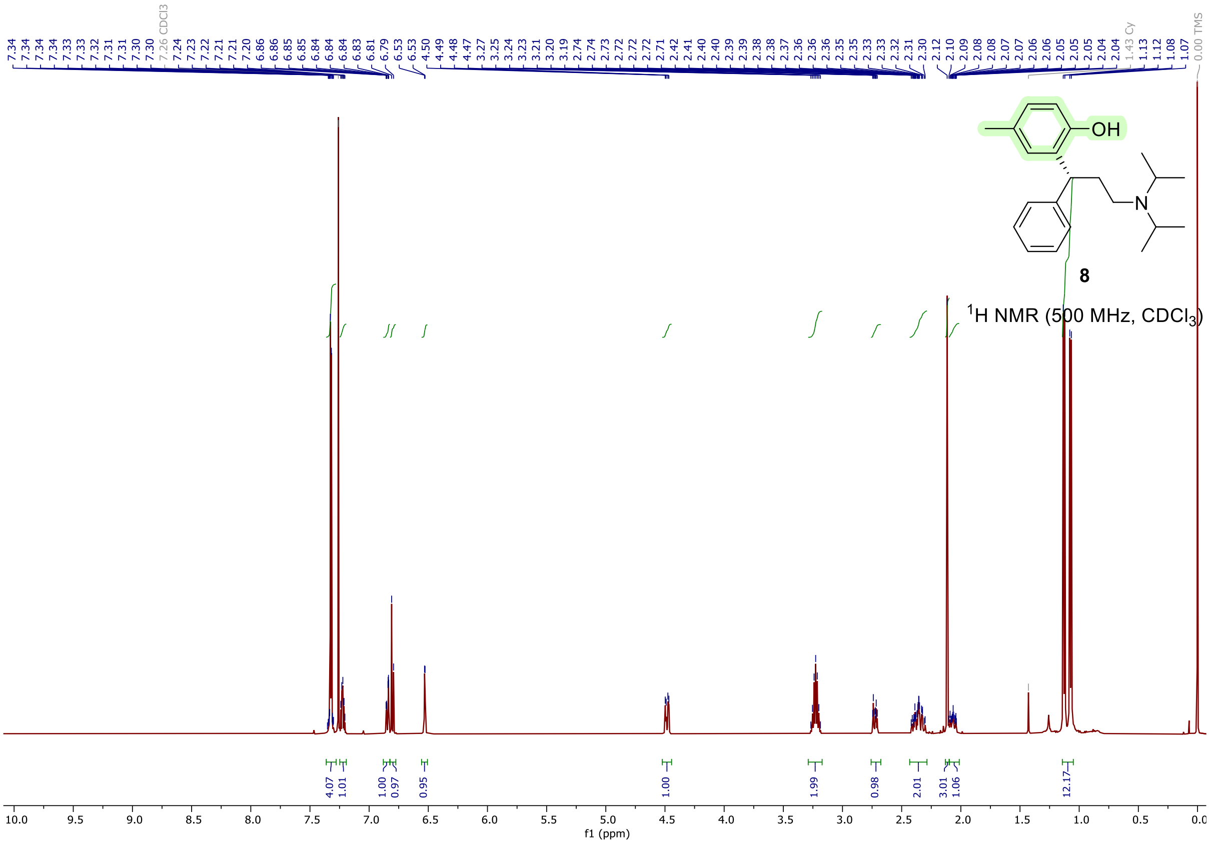

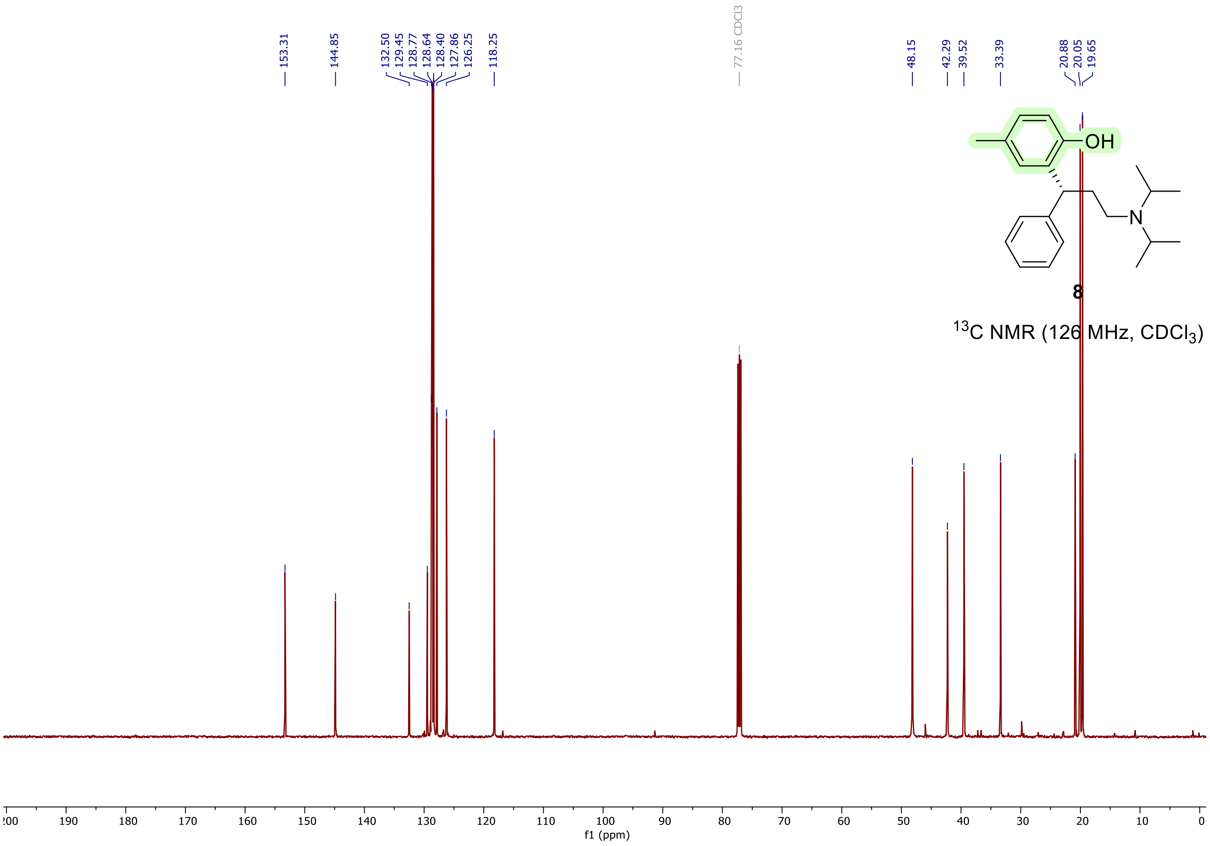

Spectra of 9 (S-Tolpropamine):

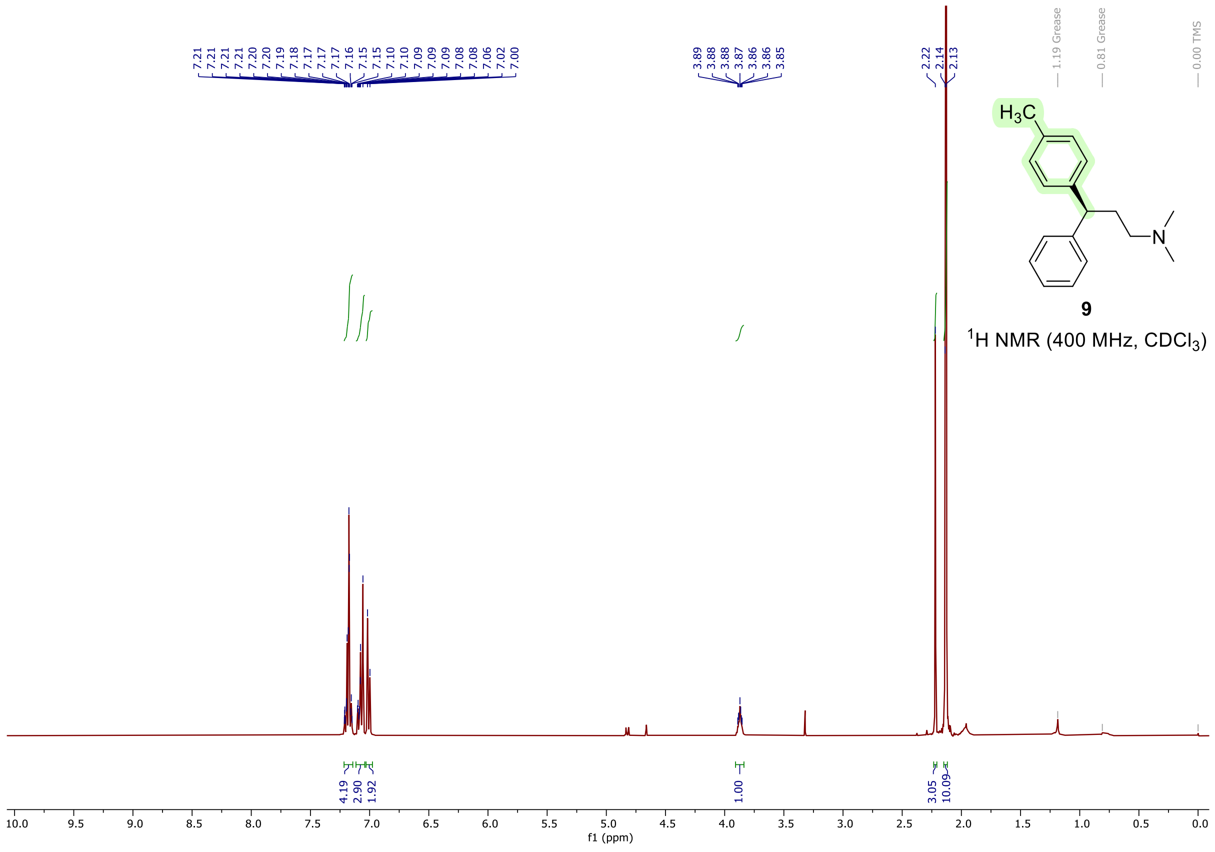

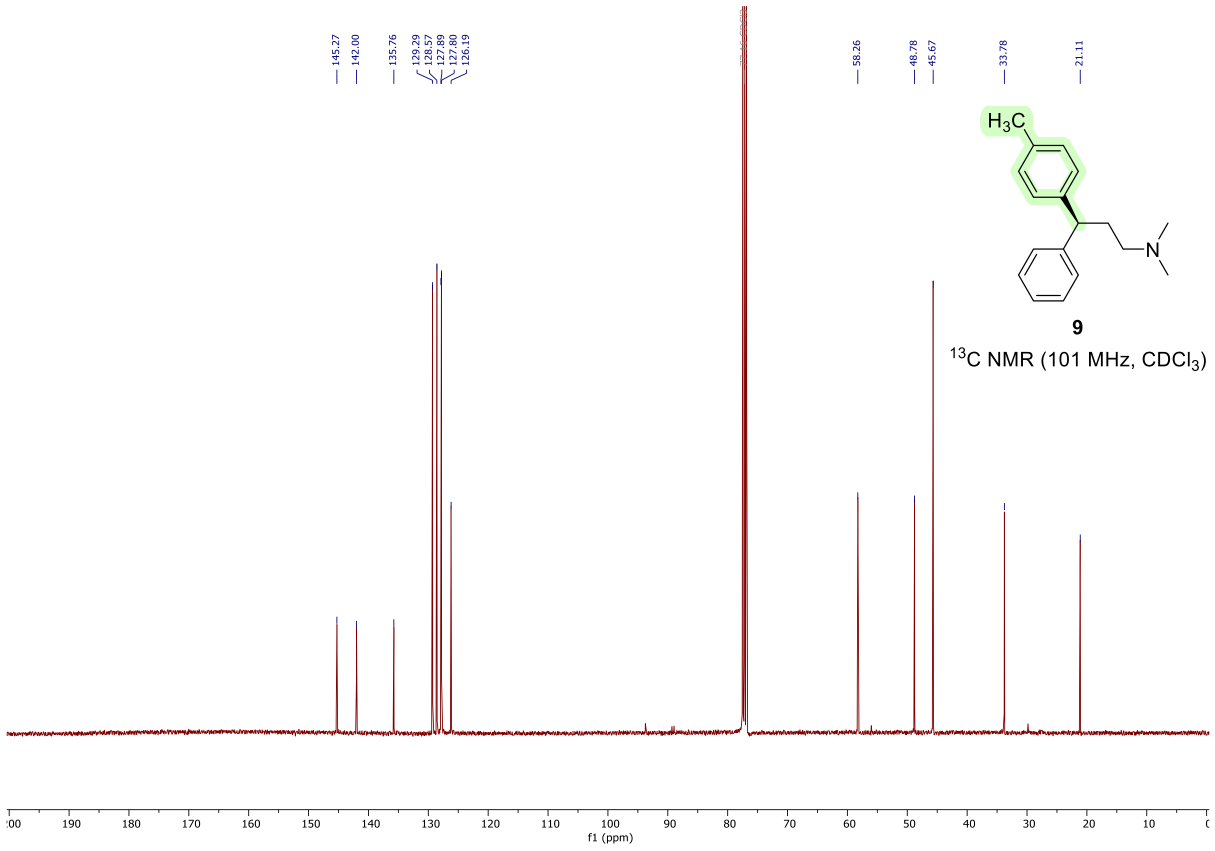

Spectra of 10g:

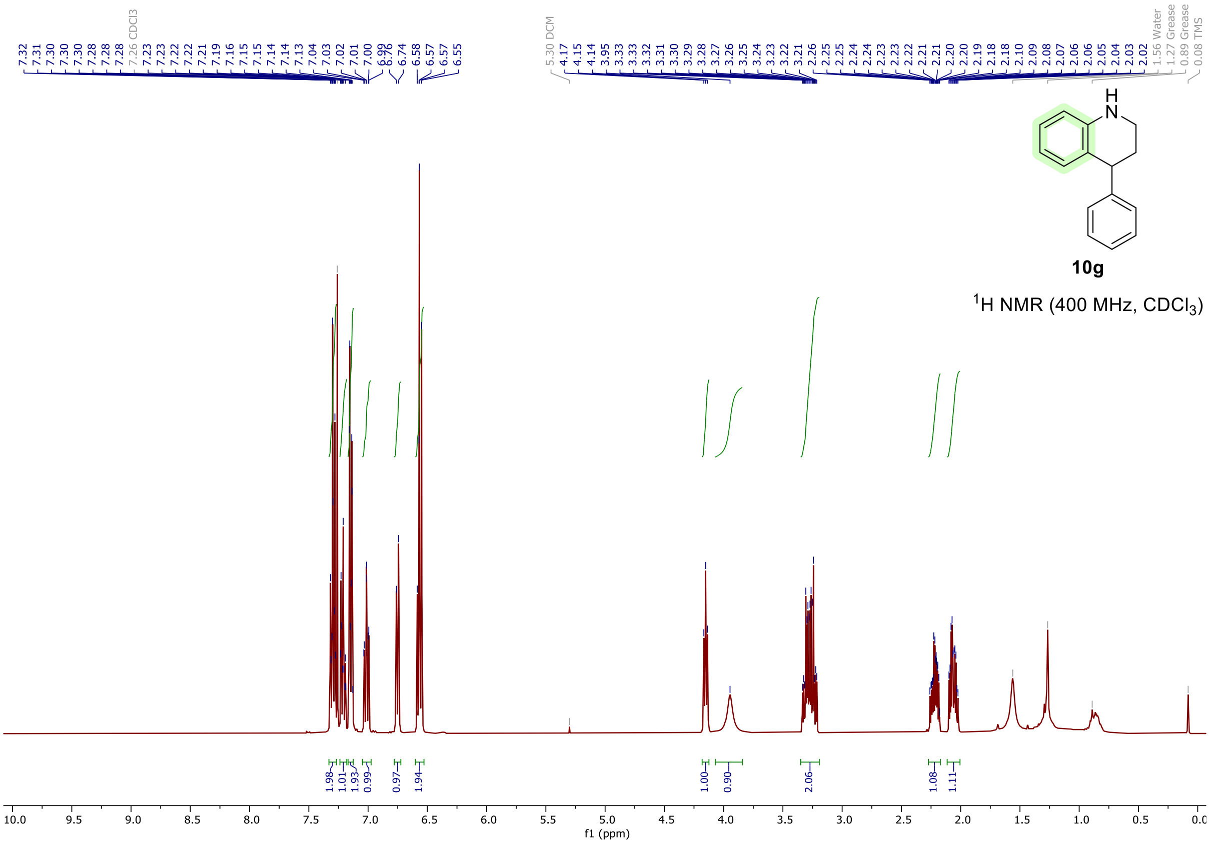

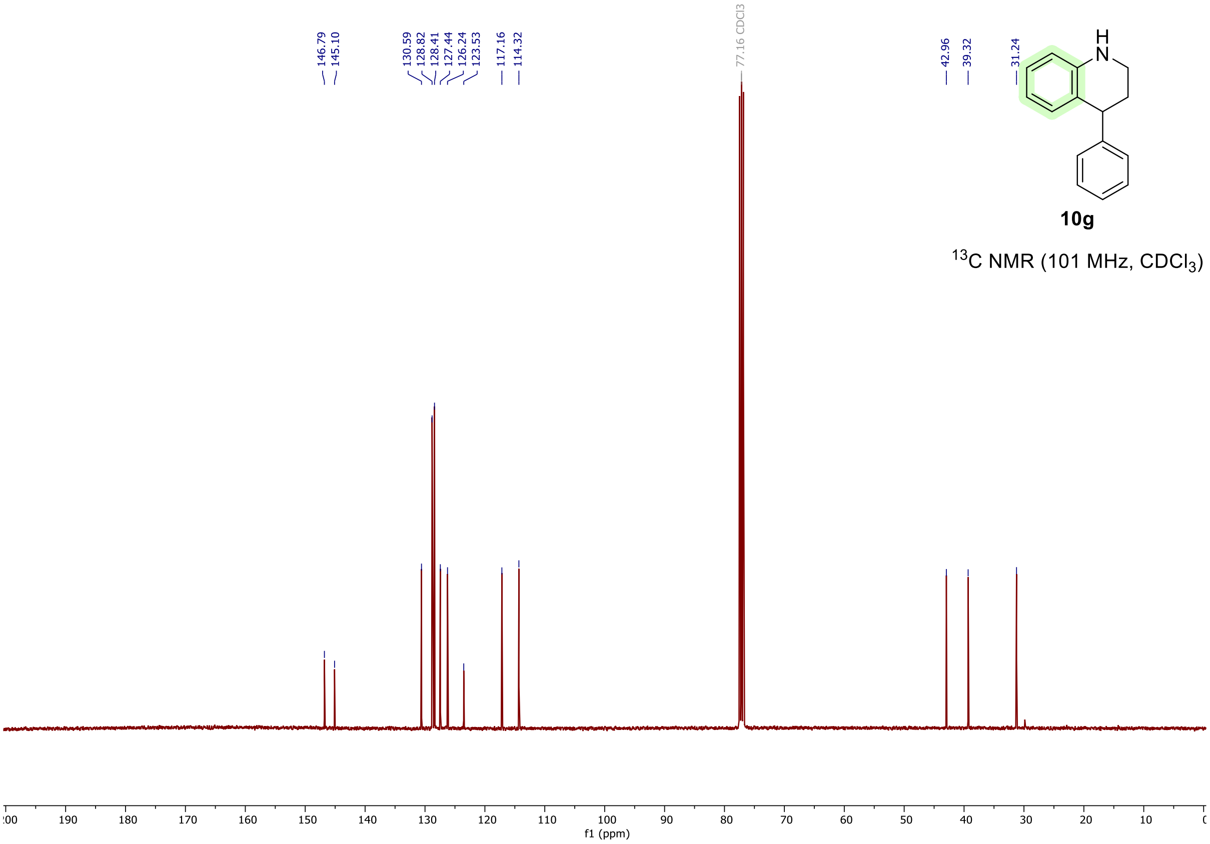

Spectra of 10h:

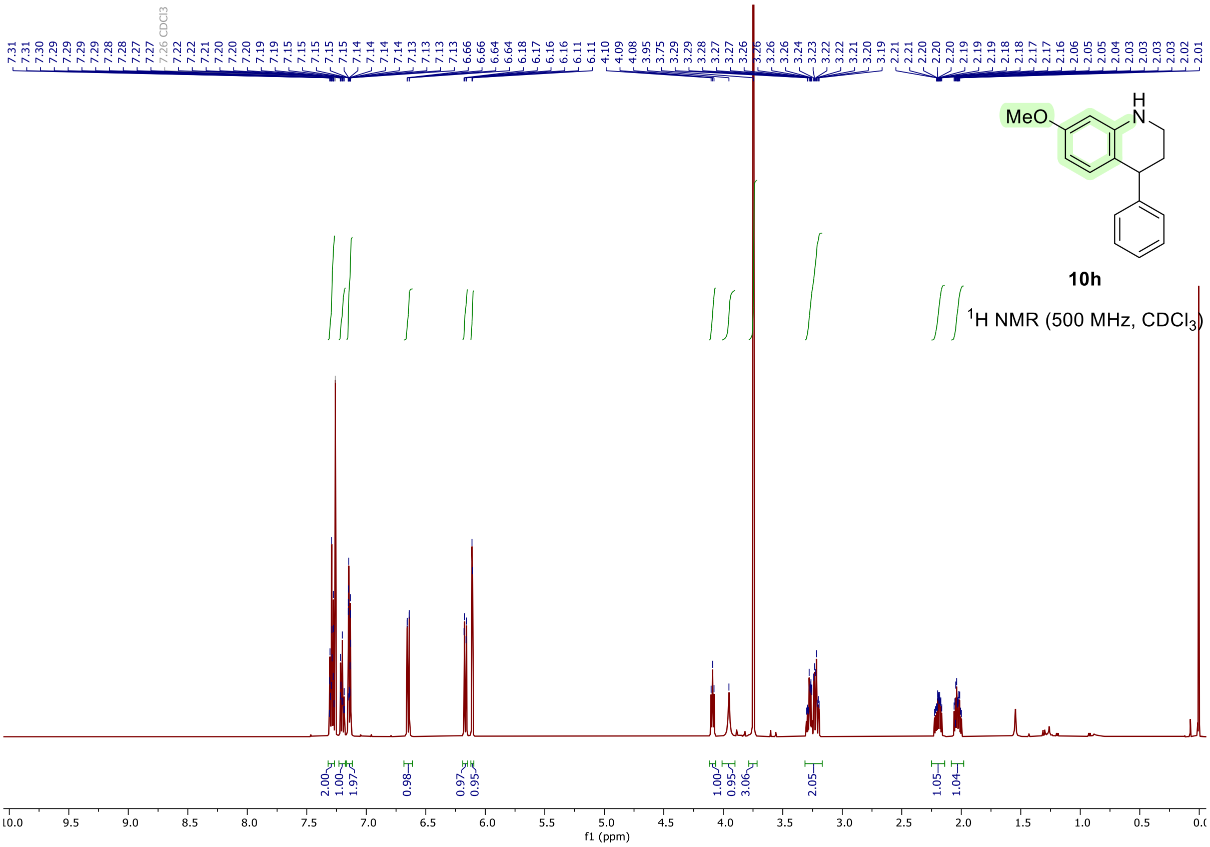

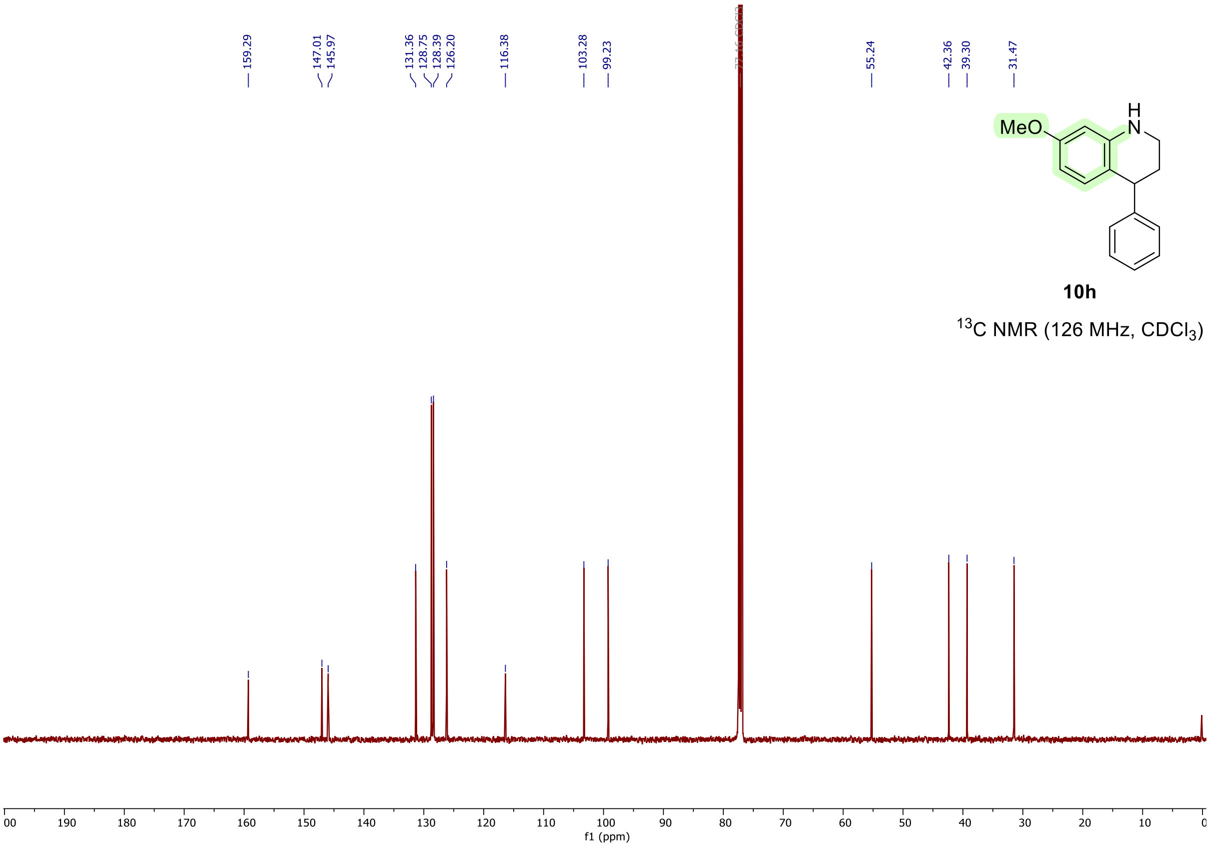

9. Chiral HPLC chromatograms

(S)-2-(3-(4-methoxyphenyl)-3-phenylpropyl)isoindoline-1,3-dione (5a) – 99% ee

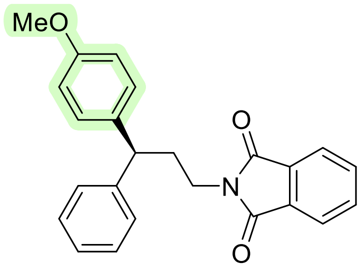

5a

HPLC: Chiralpak IC, *n*-heptane:IPA (95:5), 1 mL/min, λ = 220 nm, t_{R1} = 16.9 min, t_{R2} = 19.1 min.

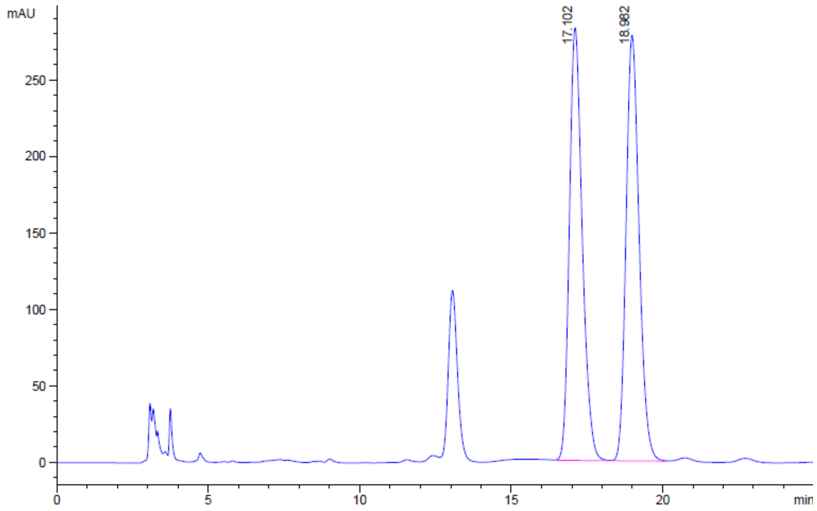

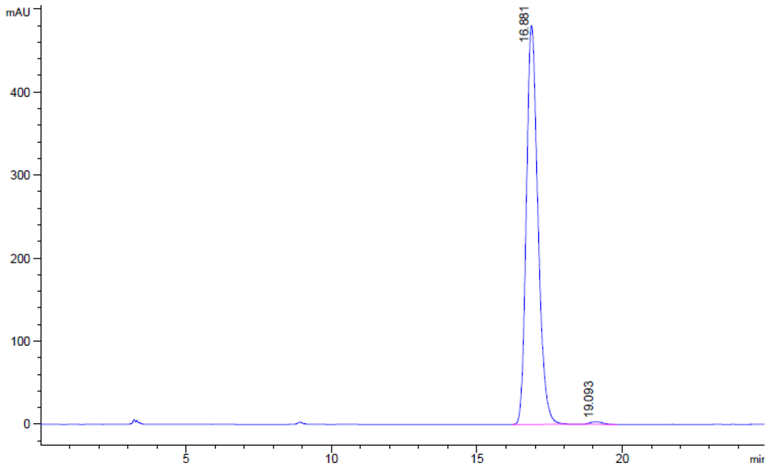

#	Meas. R	Pea	Width	Area	Height	Area %
1	16.881	BB	0.425	13301.513	480.557	99.291
2	19.093	BB	0.409	94.951	3.195	0.709

(S)-2-(3-phenyl-3-(p-tolyl)propyl)isoindoline-1,3-dione (5b) – 98% ee

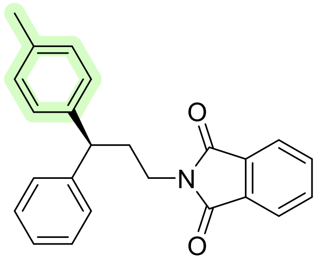

5b

HPLC: Chiralpak IA, *n*-heptane:IPA (97:3), 0.5 mL/min, λ = 210 nm, t_{R1} = 24.5 min, t_{R2} = 26.4 min.

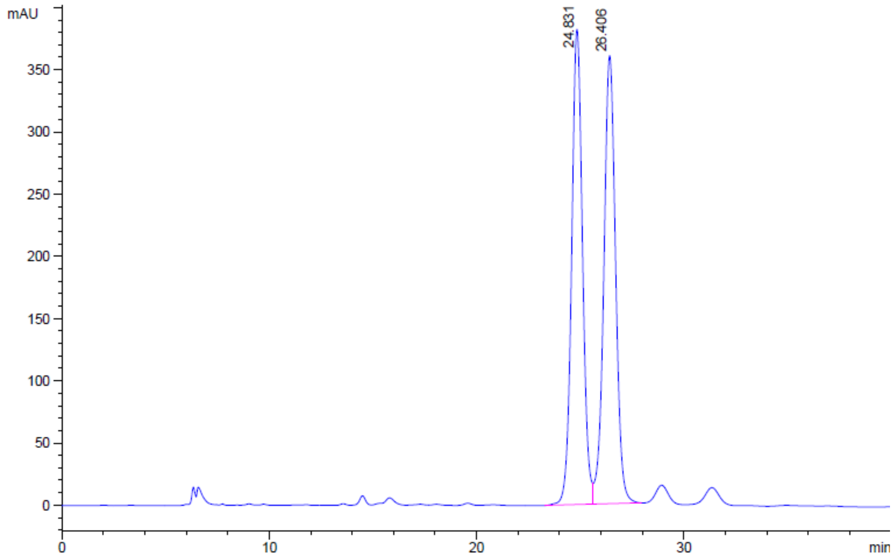

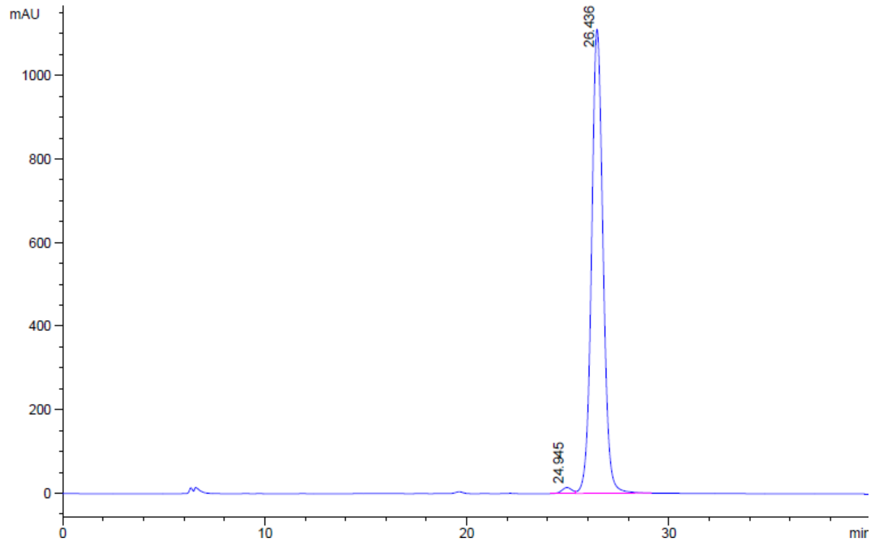

#	Meas. R	Pea	Width	Area	Height	Area %
1	24.945	BV	0.537	506.382	14.451	1.140
2	26.436	VB	0.610	43928.129	1.110e3	98.860

(S)-2-(3-(4-chlorophenyl)-3-phenylpropyl)isoindoline-1,3-dione (5c) – 99% ee

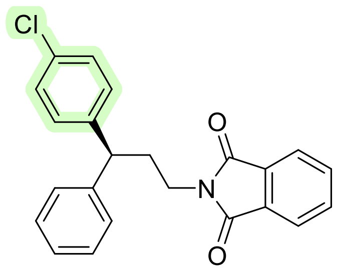

5c

HPLC: Chiralpak IA, *n*-heptane:IPA (92:8), 1 mL/min, λ = 210 nm, t_{R1} = 21.9 min, t_{R2} = 23.0 min.

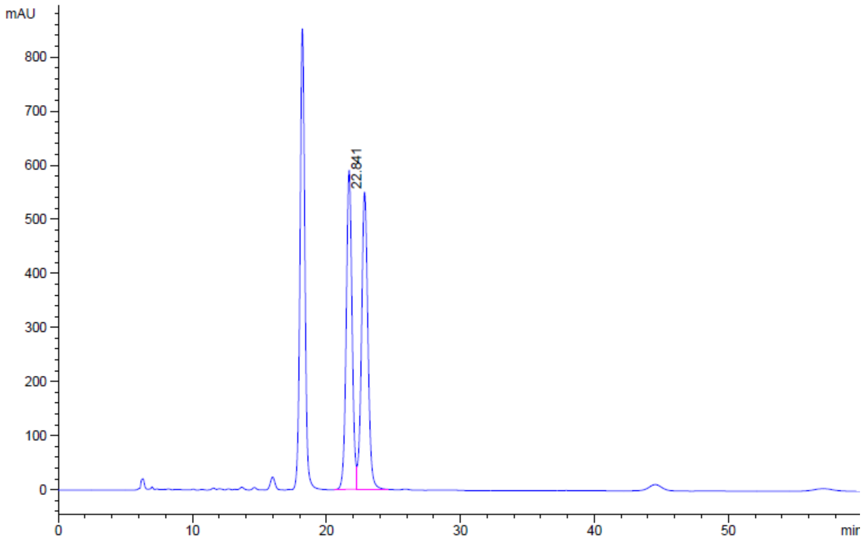

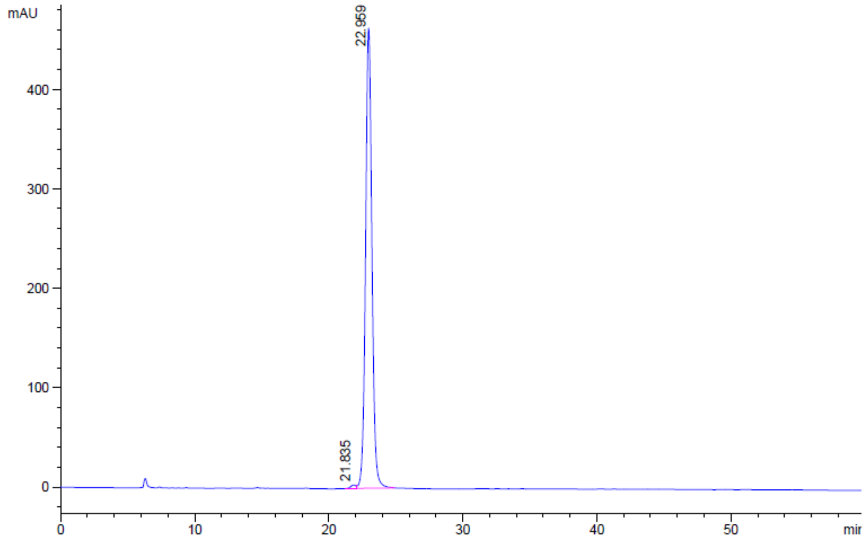

#	Meas. R	Pea	Width	Area	Height	Area %
1	21.835	BV	0.405	97.255	3.590	0.633
2	22.959	VB	0.504	15258.514	462.627	99.367

(S)-2-(3-(4-fluorophenyl)-3-phenylpropyl)isoindoline-1,3-dione (5d) – 99% ee

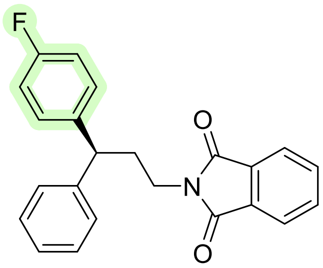

5d

HPLC: Chiralpak IA, *n*-heptane:EtOH (9:1), 0.5 mL/min, λ = 220 nm, t_{R1} = 24.4 min, t_{R2} = 29.6 min.

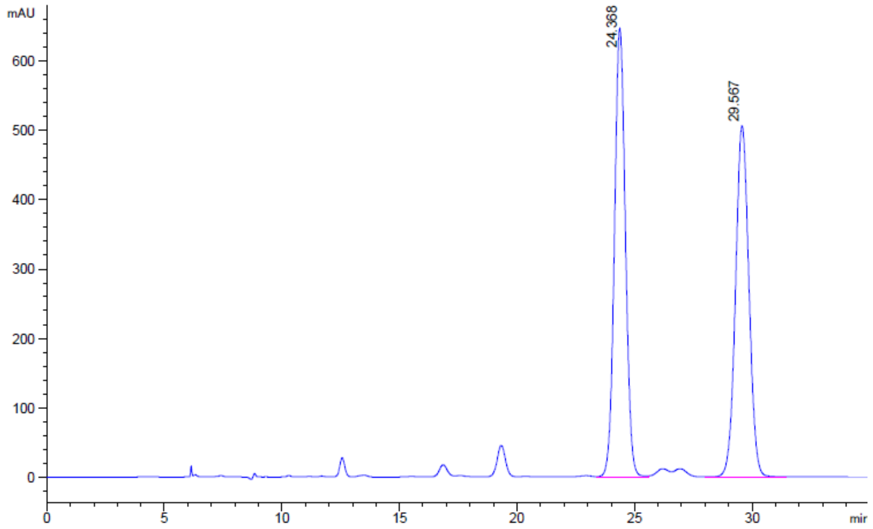

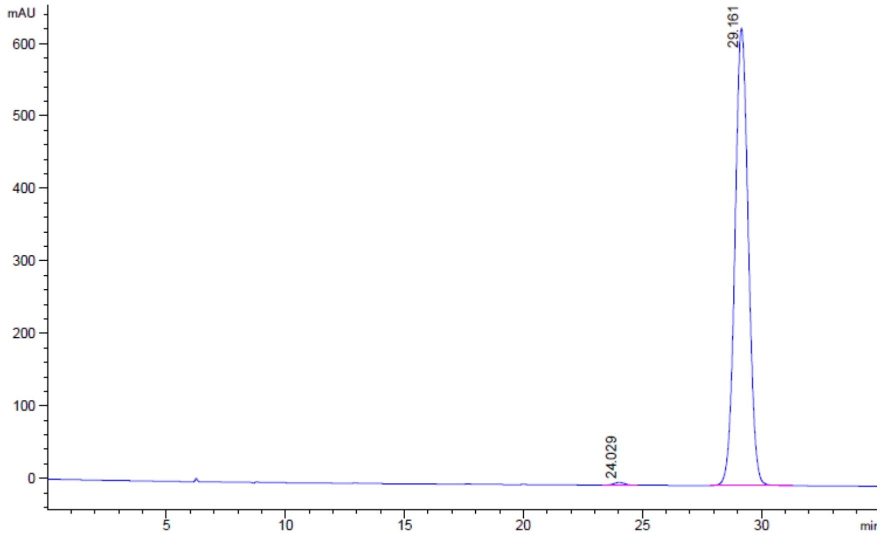

#	Meas. R	Pea	Width	Area	Height	Area %
1	24.029	MF	0.524	116.054	3.695	0.469
2	29.161	BB	0.608	24645.211	631.153	99.531

(S)-2-(3-(3-fluorophenyl)-3-phenylpropyl)isoindoline-1,3-dione (5e) – 99% ee

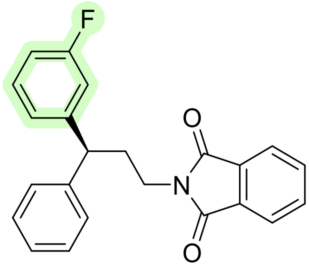

5e

HPLC: Chiralpak IA, *n*-heptane:EtOH (7:3), 0.5 mL/min, λ = 210 nm, t_{R1} = 14.3 min, t_{R2} = 15.6 min.

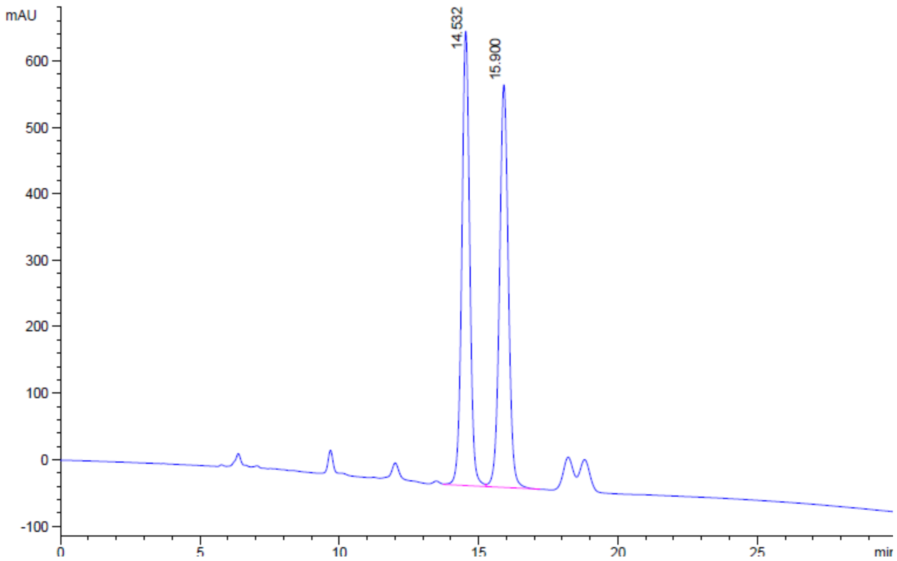

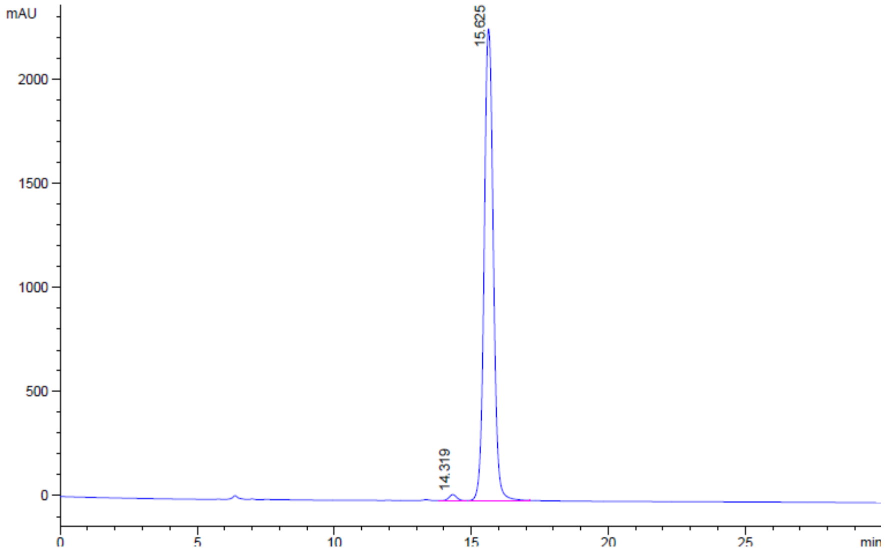

#	Meas. R	Pea	Width	Area	Height	Area %
1	14.319	BV	0.310	598.043	29.434	1.126
2	15.625	MM	0.386	52499.141	2.266e3	98.874

(S)-2-(3-(2-methoxyphenyl)-3-phenylpropyl)isoindoline-1,3-dione (5f) – 99% ee

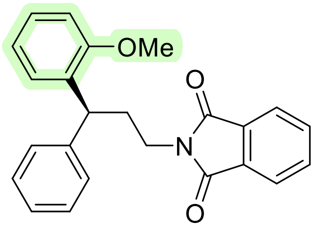

5f

HPLC: Chiralcel ODH, *n*-heptane:IPA (9:1), 0.5 mL/min, λ = 220 nm, t_{R1} = 15.7 min, t_{R2} = 17.0 min.

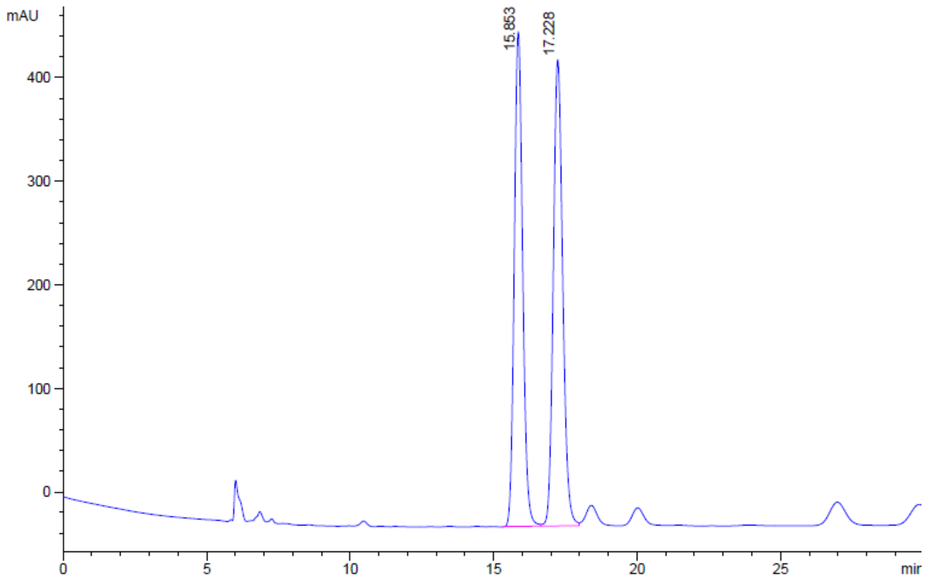

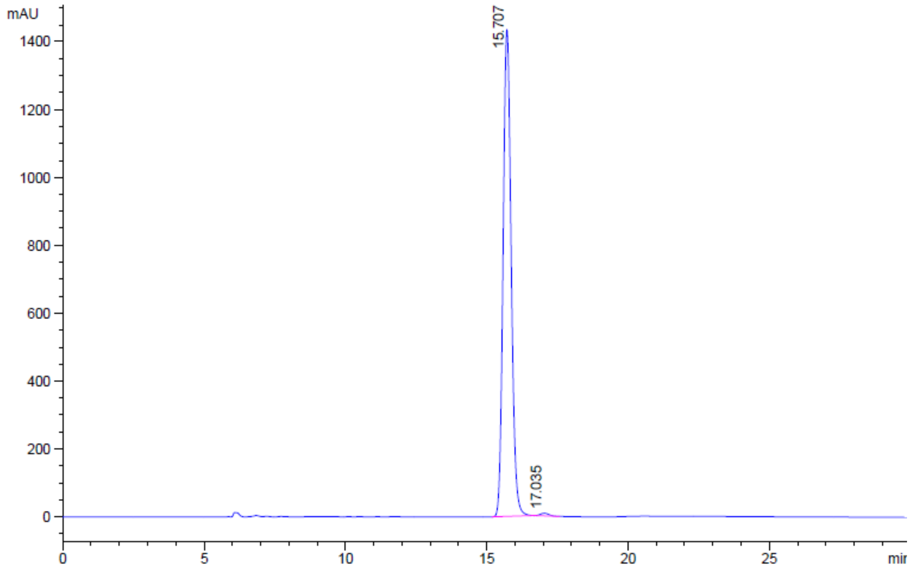

#	Meas. R	Pea	Width	Area	Height	Area %
1	15.707	MM	0.331	28533.123	1.435e3	99.390
2	17.035	MM	0.359	175.066	8.134	0.610

Chromatogram obtained in the ***0,5 mol% of catalyst*** *experiment –* ***98% ee***

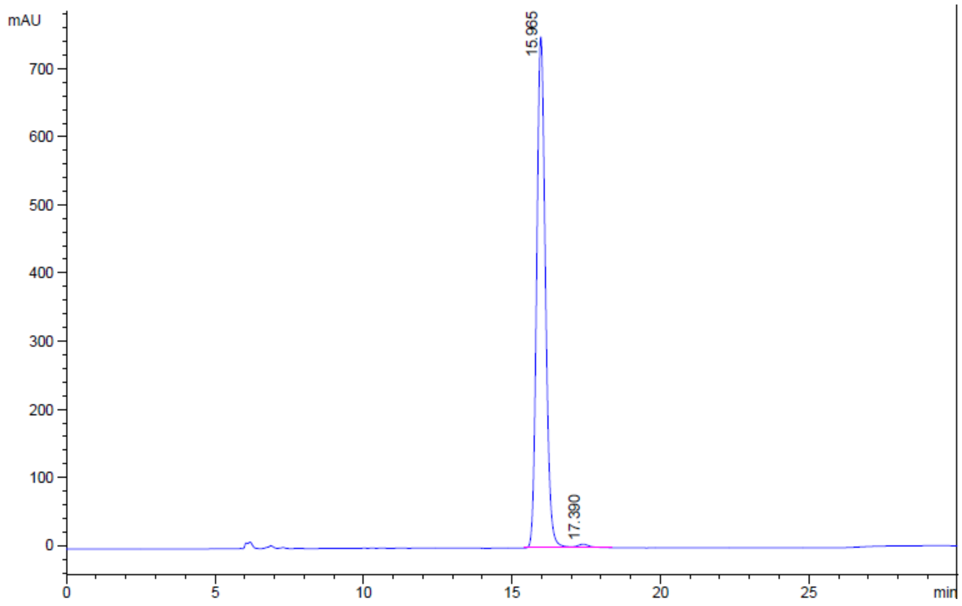

#	Meas. R	Pea	Width	Area	Height	Area %
1	15.965	BB	0.317	15341.616	749.752	98.942
2	17.390	BB	0.441	164.116	5.368	1.058

Preparation of (S)-2-(3-(2-chlorophenyl)-3-phenylpropyl)isoindoline-1,3-dione (5g) – 99% ee

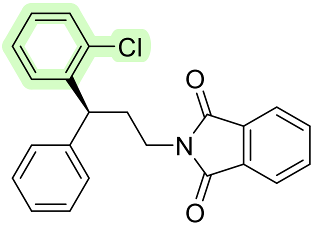

5g

HPLC: Chiralpak IC, *n*-heptane:IPA (99:1), 0.5 mL/min, λ = 210 nm, t_{R1} = 31.0, min, t_{R2} = 36.5 min.

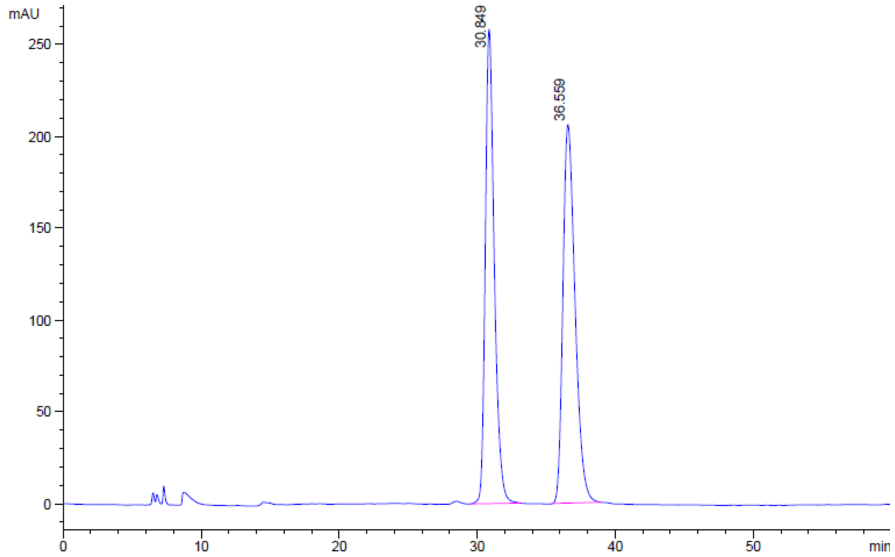

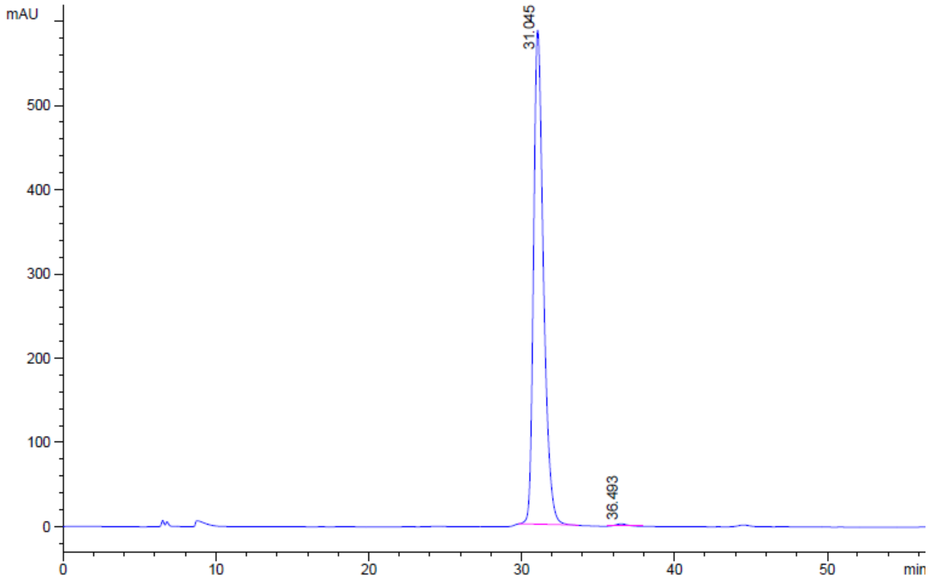

#	Meas. R	Pea	Width	Area	Height	Area %
1	31.045	MM	0.753	26550.543	587.278	99.422
2	36.493	BB	0.738	154.399	2.827	0.578

(S)-2-(3-(2-chloro-4-methoxyphenyl)-3-phenylpropyl)isoindoline-1,3-dione (5h) – 99% ee

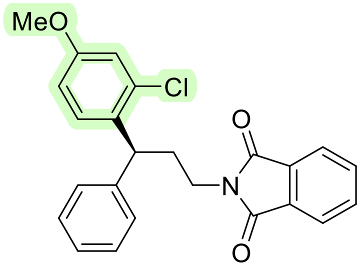

5h

HPLC: Chiralpak IC, *n*-heptane:IPA (9:1), 0.5 mL/min, λ = 210 nm, t_{R1} = 14.7 min, t_{R2} = 18.2 min.

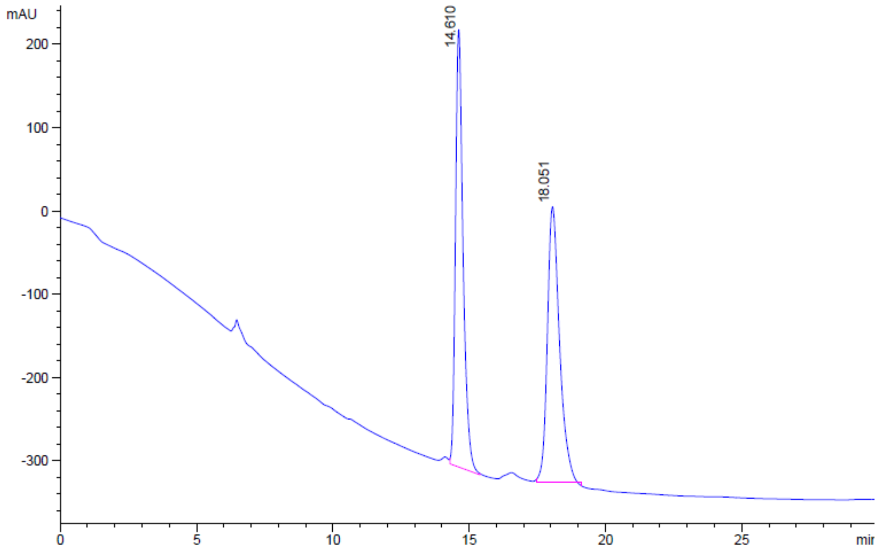

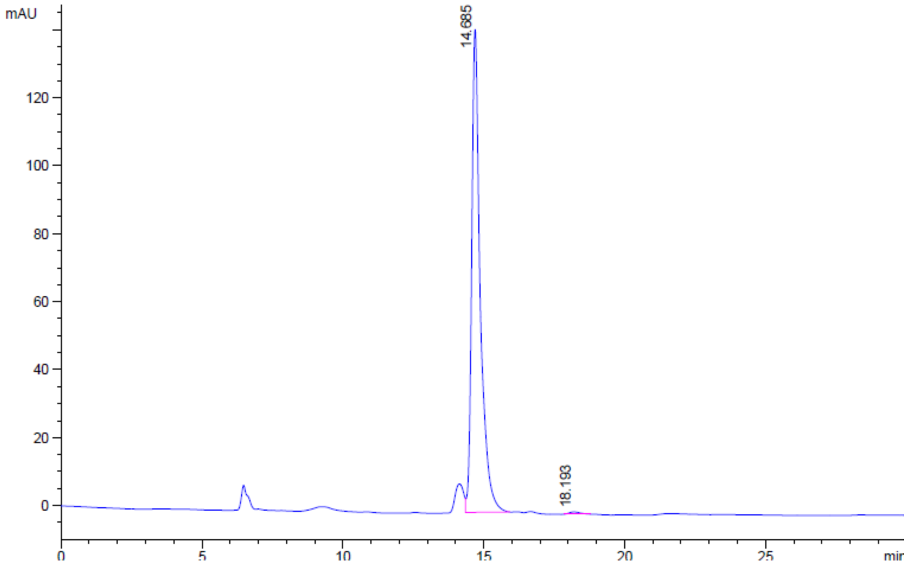

#	Meas. R	Pea	Width	Area	Height	Area %
1	14.685	VB	0.308	3008.850	142.126	99.392
2	18.193	MM	0.451	18.413	0.681	0.608

(S)-2-(3-(2-methoxy-5-methylphenyl)-3-phenylpropyl)isoindoline-1,3-dione (5i) – 99% ee

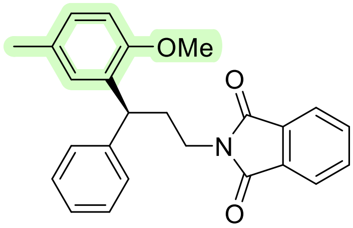

5i

HPLC: Chiralpak IC, *n*-heptane:IPA (98:2), 0.5 mL/min, λ = 220 nm, t_{R1} = 23.9 min, t_{R2} = 25.7 min.

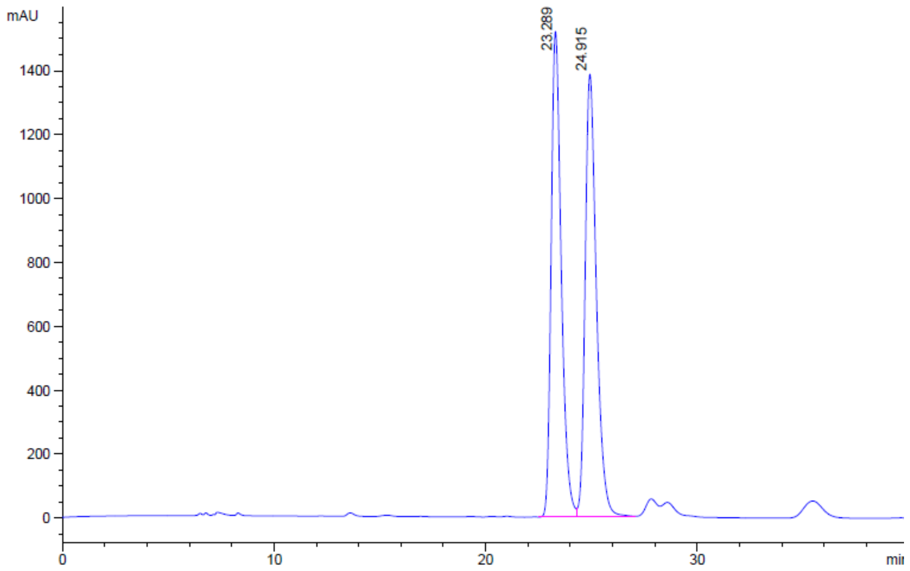

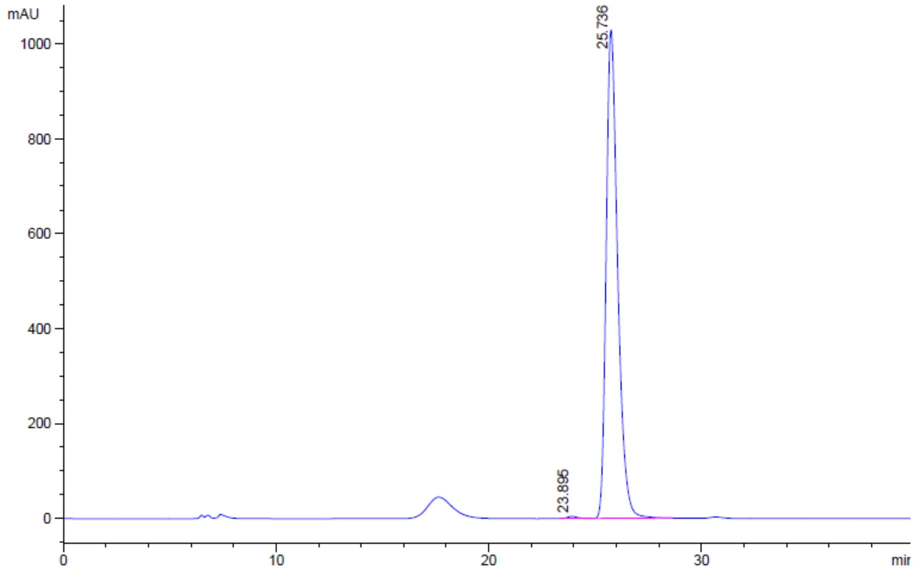

#	Meas. R	Pea	Width	Area	Height	Area %
1	23.895	BB	0.474	154.622	4.881	0.398
2	25.736	BB	0.570	38742.035	1.030e3	99.602

(R)-2-(3-(2-methoxy-5-methylphenyl)-3-phenylpropyl)isoindoline-1,3-dione ((R)-5i) – 99% ee

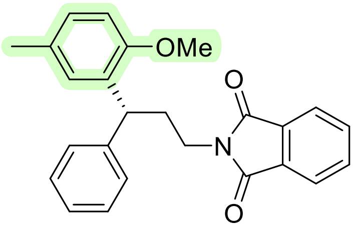

***R*-5i**

HPLC: Chiralpak IC, *n*-heptane:IPA (99:1), 0.5 mL/min, λ = 220 nm, t_{R1} = 35.8 min, t_{R2} = 39.5 min.

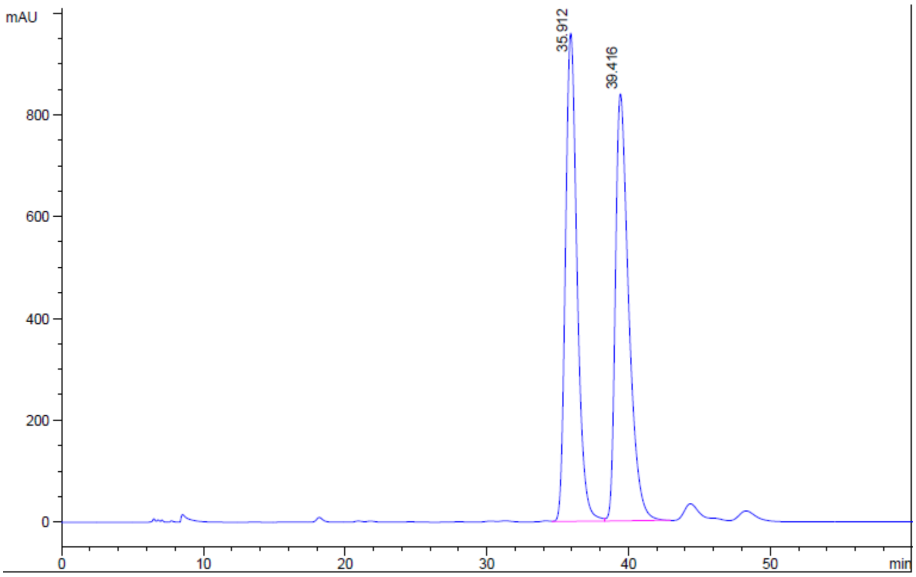

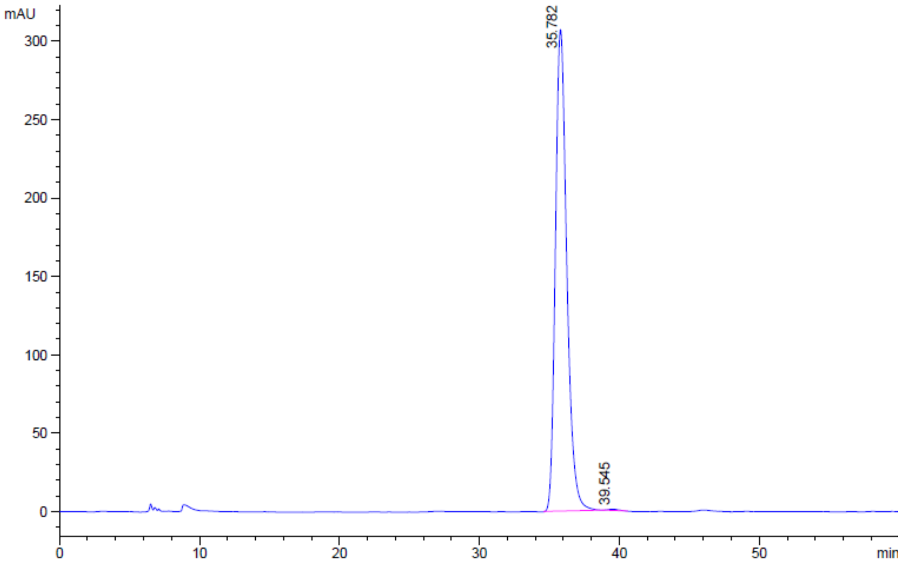

#	Meas. R	Pea	Width	Area	Height	Area %
1	35.782	MM	0.942	17377.959	307.310	99.741
2	39.545	MM	0.863	45.168	0.872	0.259

(S)-2-(3-(naphthalen-1-yl)-3-phenylpropyl)isoindoline-1,3-dione (5j) – 98% ee

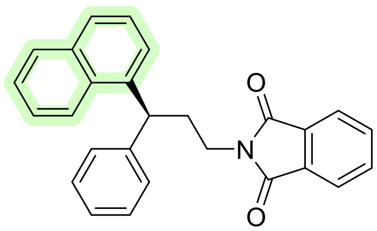

5j

HPLC: Chiralpak IA, *n*-heptane:IPA (98:2), 0.5 mL/min, λ = 210 nm, t_{R1} = 17.7 min, t_{R2} = 22.0 min.

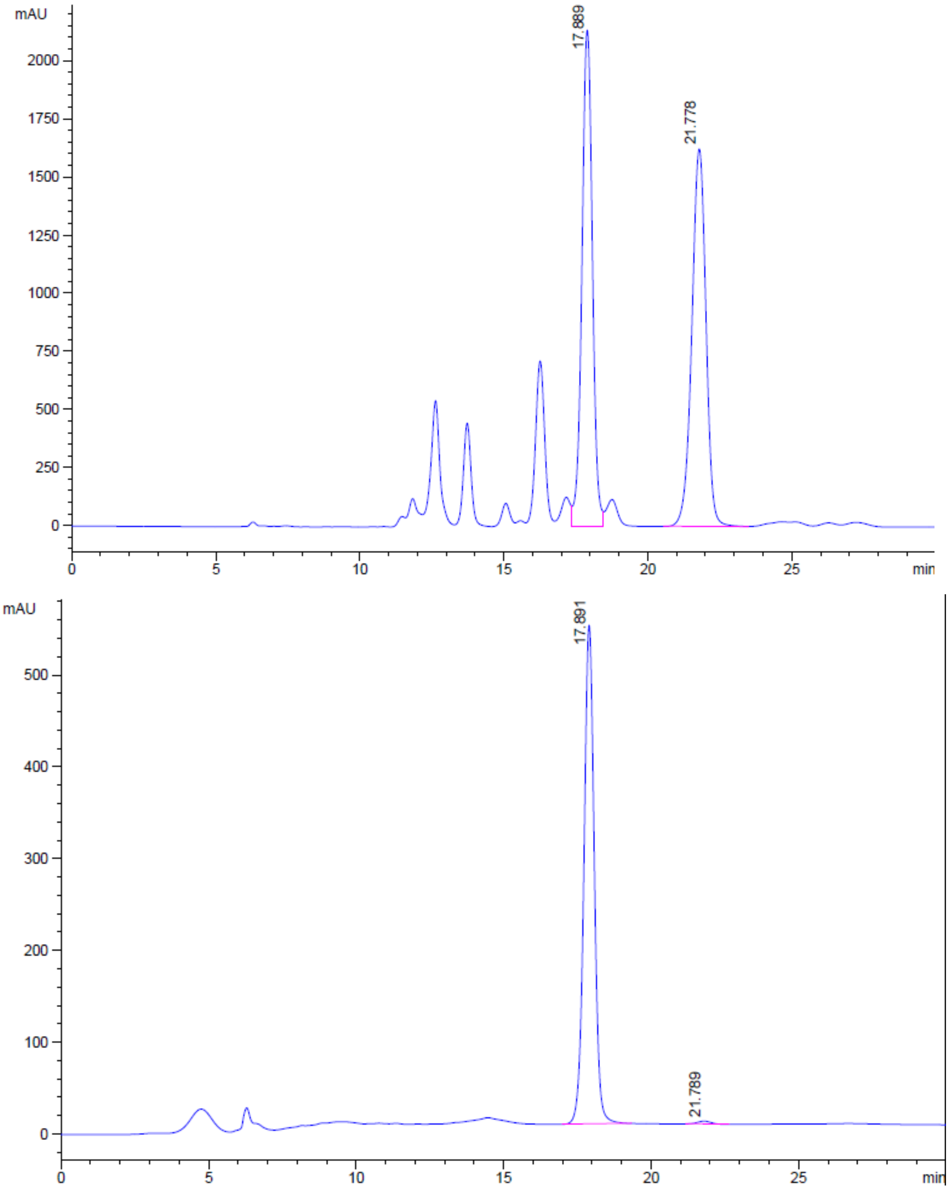

#	Meas. R	Pea	Width	Area	Height	Area %
1	17.891	BB	0.373	13210.716	542.960	99.216
2	21.789	BB	0.495	104.414	3.206	0.784
